# Cobalt‐Catalyzed Intramolecular C─H Silylation of Arenes

**DOI:** 10.1002/anie.5584122

**Published:** 2026-04-14

**Authors:** Yongqiang Xu, Valentin Poirier, Gilles Lemière, Julie Oble, Marc Petit

**Affiliations:** ^1^ CNRS, Institut Parisien De Chimie Moléculaire, IPCM Sorbonne Université Paris France

**Keywords:** C−H activation, cobalt, homogeneous catalysis, silacycles, silylation

## Abstract

Direct C−Si bond formation via C−H silylation offers an efficient atom‐economical route to organosilanes, yet existing strategies remain largely restricted to noble‐metal catalysis. Here, we disclose the first cobalt‐catalyzed intramolecular C−H silylation of arenes, enabled by the well‐defined hydride complex HCo(PMe_3_)_4_. This catalyst sequentially activates Si−H and arene C─H bonds, enabling either an hydrosilylation/cyclization sequence or direct cyclization. The method features broad functional‐group tolerance and delivers diverse heterosilacycles and silafluorenes in high yields and is extended to germacycle. DFT studies support a two‐electron mechanism involving oxidative addition of the Si─H bond, H_2_ elimination facilitated by a hydrogen acceptor, intramolecular C─H activation to form a cobaltacycle, and final reductive elimination to furnish the silacycle. Overall, this work establishes cobalt hydrides as a sustainable and effective alternative to noble metals for the synthesis of silacyclic architectures.

## Graphical abstract

Direct C─Si bond formation via C─H silylation is an atom‐economical route to organosilanes but is largely limited to noble metals. We report the first cobalt‐catalyzed intramolecular arene C–H silylation enabled by HCo(PMe_3_)_4_, which activates Si–H and C–H bonds to form diverse silacycles and silafluorenes in high yields with broad functional‐group tolerance.

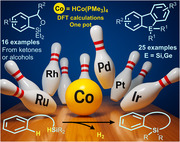

## 1 Introduction

**SCHEME 1:**
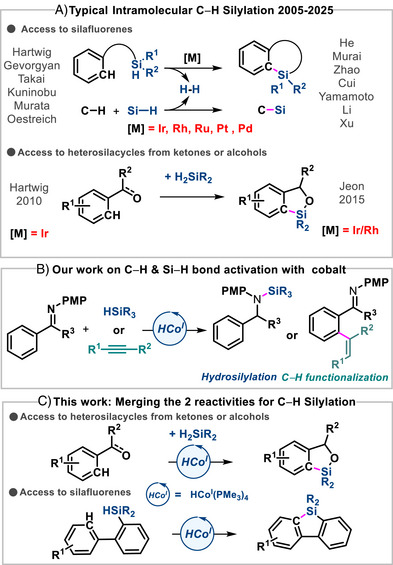
(A) Noble‐metal intramolecular C−H silylation. (B) Our previous work on C−H and Si−H activation with cobalt complexes. (C) C−H silylation catalyzed by well‐defined cobalt hydride complex.

The direct formation of C─Si bonds via C−H silylation [1, 2, 3, 4, 5] has emerged as a powerful strategy for accessing organosilanes, that is, molecules of growing significance in medical science, materials science, and synthetic chemistry [6, 7]. Compared to traditional cross‐coupling methods that require pre‐functionalized substrates, C−H silylation offers a more atom‐economical and streamlined alternative. Among its many applications, the intramolecular C−H silylation leading to silacycle has garnered particular interest due to the relevance of these frameworks in functional materials [8, 9, 10] and bioactive molecules [11, 12]. Despite its potential and the huge developments reported by Hartwig [13], Gevorgyan [14], Takai [15, 16], Murai, [17, 18] Kuninobu [19] and others, current C−H silylation methods still face major limitations. As highlighted in recent reviews by Oestreich [1], Mu [5], Liu and Niu [2], the field remains heavily dependent on noble transition metals, such as iridium, rhodium, and ruthenium, for both inter‐ and intramolecular silylation (Scheme 1A) [20]. While these metals are highly effective in promoting C−H activation and subsequent C−Si bond formation, their high cost, limited availability, and environmental impact significantly hinder broader implementation and large‐scale applications. These issues are particularly pronounced in silacycle synthesis, where intramolecular C−H silylation often requires elaborate substrate architectures, elevated reaction temperatures, and sacrificial acceptors, all of which diminish functional‐group tolerance and increase overall synthetic complexity. Directed C−H silylation strategies, although useful for achieving regioselectivity, require the installation and subsequent removal of auxiliary directing groups. In contrast, undirected C−H silylation, while attractive for late‐stage functionalization, often suffers from poor regioselectivity and limited scope [21]. Taken together, these challenges highlight both potential and limitations of noble‐metal catalysis in C−H silylation. Developing catalytic systems based on Earth‐abundant metals, such as cobalt, which has shown significant promise in C−H functionalization chemistry, represents a crucial step toward more sustainable methodologies [22]. Despite notable advances over the past decades in cobalt‐catalyzed C−H activation [23, 24], and hydrosilylation of unsaturated substrates [25], only a limited number of cobalt complexes are known to activate both Si−H and C−H bonds, a dual capability that is essential for efficient C−H silylation [2, 26]. To date, no cobalt‐based system has emerged as an efficient catalyst for the direct C−H silylation of arenes [27]. This transformation requires two‐electron redox processes, in which oxidative addition and reductive steps play key roles in both C−H and Si−H bonds activations. A survey of the literature reveals that such two‐electron pathways are generally accessible only to low‐valent cobalt complexes, whose electronic‐rich configuration allow then to undergo these transformations [28], thereby excluding high‐valent cobalt complexes, which typically operate through fundamentally different pathways. Over the past decade, our group has focused extensively on the reactivity of well‐defined low‐valent cobalt complexes [26]. We have demonstrated that cobalt hydrides of the type HCoL_4_ (L = simple phosphine ligands) serve as highly versatile catalysts, competent in C−H activation [29, 30, 31, 32], hydrosilylation [33, 34, 35], and related transformations (Scheme 1B). Among these, HCo(PMe_3_)_4_, which features strong‐field phosphine ligands, fulfills all the key criteria necessary for direct C−H silylation of aromatic substrates. Indeed, this catalyst has been shown to activate sp^2^ C−H bonds of arenes and alkenes, as well as alkynes. It also undergoes oxidative addition with silanes, and we have isolated and characterized Co(III) hydride‐silyl complexes, consistent with this mechanism. [34, 35] Furthermore, the catalyst readily promotes the formation of C−Si and N−Si bonds. Importantly, HCo(PMe_3_)_4_ exhibits high thermal stability, tolerating elevated temperatures often required for C−H silylation. Herein, we report the merger of two distinct reactivities of low‐valent cobalt hydride complexes to develop a cobalt‐catalyzed C−H silylation of arenes, providing access to silacycles of the silafluorene type as well as heterosilacycles (Scheme 1C).

## 2 Results and Discussion

**SCHEME 2:**
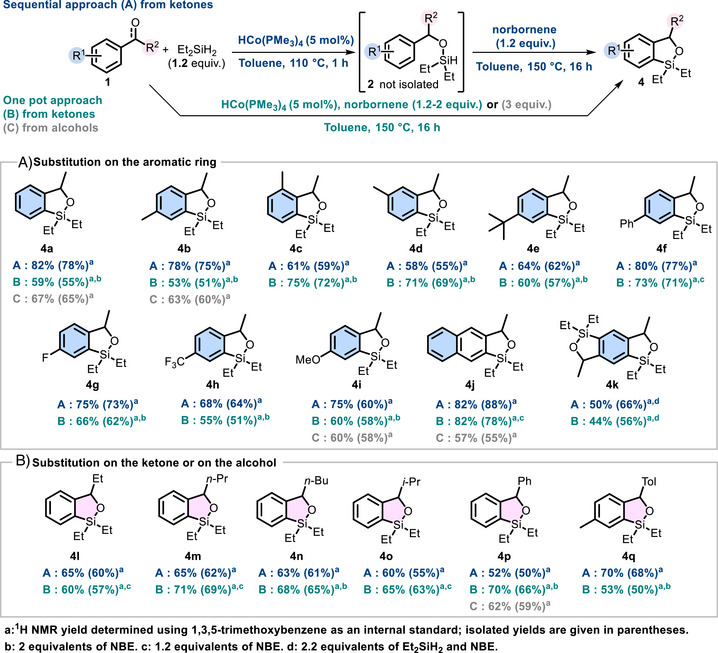
Scope of sequential and one‐pot cascade hydrosilylation of ketones (or alcohols) / C−H silylation of arenes.

Building on our previous studies on imine hydrosilylation [34] and inspired by the pioneering work of Hartwig on the ortho‐silylation of arylketones [36, 37], we initiated our investigation with aryl‐ketone substrates. We anticipated that, similarly to iridium systems, HCo(PMe_3_)_4_ could promote a one‐pot sequence combining ketone hydrosilylation [38] and dehydrogenative cyclization in presence of hydrogen acceptor. To prevent any undesired hydrosilylation of the hydrogen acceptor, we initially evaluated a stepwise protocol. We began by studying the reduction of acetophenone with diethylsilane using HCo(PMe_3_)_4_ as catalyst in toluene. Heating at 110°C for 1 h resulted in full conversion to the silylated derivative **2a** (Table 1a, entry 1). To prevent the formation of dimeric byproduct **3a**, precise control of both temperature and reaction time proved essential. Conducting the reaction at room temperature for 16 h led to a mixture containing ketone **1a**, dimeric species **3a** and desired product **2a** (Table 1a, entry 2). A decrease in conversion was also observed when THF was used instead of toluene (Table 1a, entry 3). Reducing the catalyst loading to 3 mol% required a longer reaction time to achieve full conversion, which in turn promoted the formation of side product **3a** (Table 1a, entry 4). A similar trend was observed at higher temperatures (Table 1a, entry 5) or with prolonged reaction times (Table 1a, entry 6). Finally, using near‐stoichiometric amounts of silane further diminished both conversion and selectivity (Table 1a, entry 7). Using the optimized conditions (Table 1a, entry 1), we then turned our attention to the second step, the C─H silylation. Although no additional catalyst was required, the presence of a hydrogen acceptor, such as norbornene (NBE), proved indispensable, as did the use of an elevated reaction temperature of 150°C for 16 h (Table 1b, entry 1). Under these conditions, the cyclic product **4a** was obtained in 82% ^1^H‐NMR yield (78% isolated yield, Scheme 2), accompanied by only a minor amount of the dimeric compound **3a** (4a/3a ratio = 9:1). In the absence of NBE, no formation of the desired product **4a** was detected, and complete conversion to the dimeric byproduct **3a** was observed instead (Table 1b, entry 2). Among the hydrogen acceptors tested, norbornene provided the highest efficiency, while 3,3‐dimethyl‐1‐butene led to significantly lower conversion and diminished selectivity (Table 1b, entry 3). To confirm the role of norbornene, the reaction was monitored by ^1^H NMR, which revealed the formation of one equivalent of norbornane in the reaction mixture alongside the desired product (Figure S1). Decreasing the reaction temperature to 110°C resulted in reduced conversion (Table 1b, entry 4), whereas higher temperatures of 180°C promoted the formation of dimer **3a**, thereby lowering selectivity (Table 1b, entry 5). Shorter reaction times also had a detrimental impact on both conversion and selectivity (Table 1b, entries 6 and 7).

**TABLE 1:** Optimization of the sequence hydrosilylation/C−H silylation.

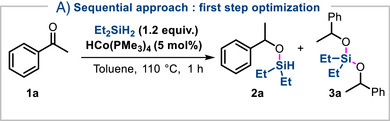		
	Deviation from standard condition	1a/2a/3a^a^
**1**	**none**	**0/100/0**
**2**	**r.t. instead of 110°C**	**34/20/46** ^b^
**3**	**THF instead of toluene at 90°C**	**69/31/0** ^b^
**4**	**3 mol% instead of 5 mol%**	**0/91/9** ^b^
**5**	**150°C instead of 110°C**	**0/79/21**
**6**	**16 h instead of 1 h**	**0/11/89**
**7**	**1.05 equiv. instead of 1.2 equiv.r.t**.	**53/27/20**

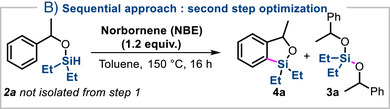			
	Deviation from standard condition	2a/4a/3a^a^	4a^c^
**1**	**none**	**0/90/10**	**82%**
**2**	**no hydrogen acceptor**	**0/0/100**	—
**3**	**(3,3‐dimethyl‐1‐butene) instead of NBE**	**17/18/65**	—
**4**	**110°C instead of 150°C**	**74/6/20**	—
**5**	**180°C instead of 150°C**	**0/36/64**	—
**6**	**1 h instead of 16 h**	**65/19/16**	—
**7**	**6 h instead of 16 h**	**13/74/13**	—

^a^ Ratio measured by ^1^H‐NMR spectroscopy.

^b^ Reaction was run for 16 h.

^c^ ^1^H‐NMR yield of **4a** calculated using 1,3,5‐trimethoxybenzene as internal standard.

We next examined this reactivity using a more sustainable one‐pot approach, in which norbornene was added at the beginning of the reaction. After a brief optimization (Table S3), we successfully obtained the desired compound **4a** after 16 h at 150°C, in 59% NMR yield (55% isolated yield, Scheme 2), which is slightly lower than that obtained with the sequential protocol. For acetophenone, the use of 2 equivalents of NBE proved necessary to minimize the formation of dimeric by‐product; however, this requirement does not apply to all ketones, as dimer formation appears to be influenced by steric hindrance [39]. We also evaluated this transformation starting from benzyl alcohol **1a'**, and the one‐pot sequence proved equally effective, affording **4a** in 65% isolated yield (Scheme 2) [40, 41]. With the alcohols, the addition of 3 equivalents of NBE was required due to the intrinsic formation of two equivalents of H_2_ and to minimize the formation of the dimer. It is noteworthy that other variations of silanes appear unsuccessful or give mixtures of compounds in the sequential approach. With these optimized conditions in hand (Table S4), we then explored the scope of the reaction using all three strategies (Scheme 2): from ketones, the sequential approach (A) and the one‐step protocol (B), as well as, for selected cases, the one‐pot sequence starting from benzyl alcohols (C).

Variation on the aromatic ring was first examined by introducing methyl substituents at the *para*, *meta*, and *ortho* positions of the aryl‐ketone. These modifications had no significant impact on the reaction efficiency, affording the corresponding products **4b**, **4c** and **4d** in 75%, 59%, and 55% isolated yields, respectively, under the sequential conditions. Similar results were obtained in the one‐pot procedure, which gave **4b–4d** in 51%, 72%, and 69% yields. Further *para* substitution with *t*‐Bu, Ph, F, CF_3_, or MeO groups revealed no influence on the reaction outcome. Using approach A, the corresponding products **4e**, **4f**, **4** **g**, **4** **h**, and **4i** were obtained in 62%, 77%, 73%, 64% and 60% yields, respectively. Approach B again provided comparable results, with isolated yields ranging from 51% to 78%. Replacing the phenyl ring with an naphthyl group led to enhanced reactivity, providing **4j** in 88% and 78% yields under the sequential and one‐pot conditions, respectively. Double C─H silylation was also feasible using 1,4‐diacetylbenzene as the starting material, affording **4k** in 66% and 56% yields under the two conditions. Next, variation at the ketone moiety was examined and proved to be well‐tolerated, giving the desired products in 60%, 62%, 61%, and 51% isolated yields with ethyl, *n*‐propyl, *n*‐butyl, and *i*‐propyl substituents, respectively, again with comparable outcomes for both approaches. Bis‐aryl‐ketones were also compatible, delivering **4p** and **4q** in 50% and 68% yields, respectively. Finally, this reaction was also feasible starting from secondary benzylic alcohols following procedure C (one‐pot) with 3 equivalents of NBE, leading to compounds **4b**, **4i**, **4j**, and **4p** in 55%–65% isolated yields.

**SCHEME 3:**
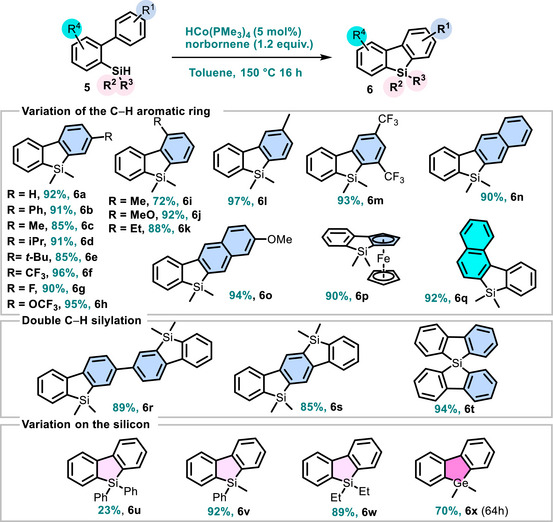
Scope of intramolecular C−H silylation of arene: access to silafluorenes.

Encouraged by the efficiency of the reduction/C─H silylation sequence of aryl‐ketones (and alcohols), we next turned our attention on the synthesis of silafluorenes structures (Scheme 3) that, to the best of our knowledge, have not previously been accessed using non‐noble metal catalysis via C─H silylation [42].

After a short optimization (Table S5), we found that the optimized one‐pot protocol (approach B), provided the most efficient conditions for the synthesis of silafluorenes. We first investigated the influence of substituents on the arene bearing the C─H bond undergoing activation. *Para*‐substitution on the silyl‐arene had no significant electronic or steric effect, as compounds **6a–6 h** (H, Ph, Me, *i*Pr, *t*‐Bu, CF_3_, F, and OCF_3_) were isolated in excellent yields (85%–96%). Likewise, *ortho*‐substitution had only a modest effect, delivering **6i**–**6k** (Me, MeO, and Et) in 72%, 92%, and 88% yields, respectively. Notably, *meta*‐substitution furnished exclusively a single regioisomer **6l** in 97% yield, whereas previous reports describe mixtures [15, 16]. Extended π‐conjugated systems were also well tolerated, providing **6n**, **6o**, **6q** in excellent yields (90%–94%). A ferrocenyl‐substituted silyl‐arene was similarly compatible, affording fluorene **6p** in 90% yield. We next explored double C─H silylation using various substrate designs. Diphenyl precursor underwent efficient double silylation to give **6r** in 89% yield. The more challenging bis‐C─H silylation on a single aryl unit furnished **6s** in 85% yield, while the spiro‐silylfluorene **6t** was obtained in excellent 94% yield. Subsequent variation at the silicon center revealed notable trends. Diphenylsilane afforded the corresponding silafluorene **6u** in a modest 23% yield, even after extended reaction times. In contrast, phenylmethylsilane and diethylsilane proved fully compatible under the standard conditions, delivering **6v** and **6w** in 92% and 89% yields, respectively. Finally, replacing dimethylsilane with dimethylgermane afforded the corresponding germyl analogue **6x** in 70% yield after 64 h of reaction (Scheme 3).

**FIGURE 1:**
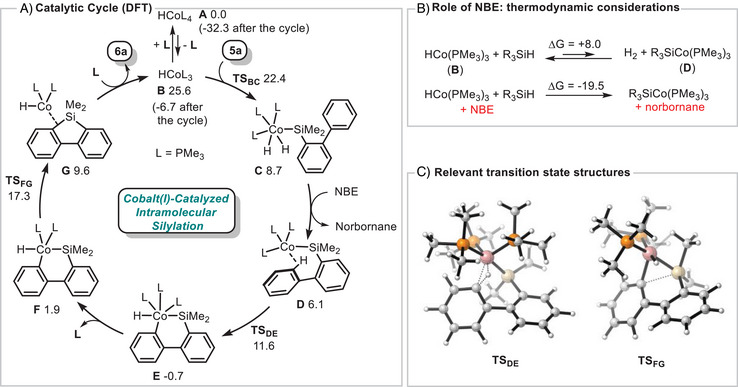
Mechanism insights supported by DFT calculations at PBE0/def2‐TZVP//PBE0/def2‐SVP level of theory. All energies discussed are based on Gibbs free energies at 423.15 K and are given in kcal.mol^−1^. Solvent effects were included using SMD solvation model (Toluene). The structure of **TS_DE_** and **TS_FG_** have been drawn using CylView software.

A plausible mechanism, supported by DFT calculations, for the cobalt‐catalyzed cyclodehydrogenation of silane **5a** into silafluorene **6a** is depicted in Figure 1A. The active species **B** is initially generated from the starting complex **A** through the dissociation of one PMe_3_ ligand. In the presence of silane **5a**, this 16 electron complex undergoes a strongly favored oxidative addition into the Si─H bond to form the silacobalt(III) intermediate **C**, a reactivity previously established for this cobalt system [33, 34]. Intermediate **C** then evolves into the cobalt(I) complex **D** via H_2_ elimination. Experimentally, the use of NBE as a hydrogen acceptor proves beneficial as it strongly facilitates the formation of **D**, with concomitant formation of norbornane (Figures 1B and S3). Thermodynamically, the conversion of complex **B** to **D** in the presence of NBE is associated with a substantial energy gain of ca. 27.5 kcal.mol^−1^ rendering the process highly exergonic (Figure 1B) and significantly driving the reaction. Remarkably, complex **D** features an intramolecular stabilizing agostic interaction with a proximal C–H aromatic bond, which undergoes oxidative addition to furnish cobaltacycle **E**. This step is exergonic by 6.8 kcal.mol^−1^ and proceeds through a low‐lying transition states **TS_DE_** with an activation barrier of only 5.5 kcal.mol^−1^ (Figure 1A,C). The subsequent reductive elimination leading to the formation of the silafluorene cannot occur directly from complex **E**, likely due to the presence of three electron‐rich alkylphosphines around the metal center. However, upon formation of complex **F**, in which one phosphine has dissociated, reductive elimination becomes feasible, proceeding **TS_FG_** with an activation energy of 15.4 kcal.mol^−1^ (Figure 1A,C). Although the formation of complex **G** is slightly endergonic, recoordination of PMe_3_ together with release of silafluorene **6a** allows to regenerate the active catalyst **B**, with an energy gain exceeding 17 kcal.mol^−1^. Overall, catalytic transformation is exergonic by approximatively 32 kcal.mol^−1^. Consistent with these computational data, the reaction can also proceed in the absence of NBE, although at a significantly reduced rate due to less favorable H_2_ elimination step.

## 3 Conclusion

In summary, we describe the first cobalt‐catalyzed intramolecular C−H silylation of arenes, providing a sustainable alternative to noble‐metal systems for synthesis of silacycles. The well‐defined hydride complex HCo(PMe_3_)_4_ promotes both Si−H activation and arene C−H activation, enabling efficient access to silafluorenes and related heterosilacycles under operationally simple conditions. The method displays broad scope, including challenging substitution patterns and double C−H silylation. Mechanistic and computational studies support two‐electron pathway involving Si−H oxidative addition, C−H activation, and reductive elimination. Overall, this work establishes cobalt hydrides as powerful and versatile catalysts for constructing C−Si bonds through intramolecular C−H silylation.

## Conflicts of Interest

The authors declare no conflicts of interest.

## Supporting information



The authors have cited additional references within the Supporting Information [43, 44, 45, 46, 47, 48, 49, 50, 51, 52, 53, 54, 55, 56, 57, 58].
**Supporting File**: anie72205‐sup‐0001‐SuppMat.pdf.

## Data Availability

The data that supports the findings of this study are available in the supplementary material of this article.
