# Supplementary material for: Cobalt‐Catalyzed Intramolecular C─H Silylation of Arenes
Source: Angew Chem Int Ed Engl. 2026 Apr 14;65(21):e5584122. doi: 10.1002/anie.5584122 (PMC13182198; doi:10.1002/anie.5584122)

Supporting Information

Cobalt-Catalyzed Intramolecular C−H Silylation of Arenes

Yongqiang Xu,[a] Valentin Poirier,[a] Gilles Lemière,[a] Julie Oble*[a] and Marc Petit*[a]

Sorbonne Université, CNRS, Institut Parisien de Chimie Moléculaire, IPCM, 4 place Jussieu, 75005 Paris, France.

julie.oble@sorbonne-universite.fr

marc.petit@sorbonne-universite.fr

Table of Contents

1. Materials and Methods 2

2. Optimizations of reaction conditions (ketones and secondary alcohol) 3

2.1. General procedure A (1st step + 2nd step) 3

2.2. General procedure B (one-pot) 4

2.3. General procedure C (secondary alcohol) 5

2.4. ^{1}H-NMR study: role of the NBE 5

2.5. Characterizations of final products 6

3. Optimizations of reaction conditions (biphenyls) 13

3.1. General Procedure D 13

3.2. ^{1}H-NMR study: role of the NBE 14

3.3. Synthesis and characterizations of starting materials 5 14

3.4. Synthesis and characterizations of Final products 6 28

3.5. DFT calculations 34

4. ^{1}H, ^{13}C and ^{19}F NMR Spectra 45

1. Materials and Methods

Silylation reactions were carried out in an N_2-filled glovebox using oven-dried glassware and Teflon-coated magnetic stirring bars. $HCo(PMe_3)_4$ was synthesized according to the reported procedure.[34] (*Caution: Owing to the presence of trimethylphosphine as a ligand, the catalyst is pyrophoric upon exposure to air and must be handled and weighed under an inert atmosphere (glovebox*) Diethylsilane and chlorodimethylsilane were purchased from Sigma-Aldrich and used without further purification. Norbornene (NBE) was obtained from Alfa Aesar and used as received. Toluene (VWR Chemicals BDH) was degassed by argon purging, and THF was dried using a Braun MB SPS-800 solvent purification system. All other reagents and solvents were used as received.

Thin-layer chromatography (TLC) was performed on Merck 60 F254 silica gel plates. Visualization was achieved by fluorescence quenching (λ = 254 nm) or by staining with $KMnO_4$ or *p*-anisaldehyde solutions. Flash column chromatography was carried out on Merck Geduran® SI 60 silica gel (40-63 µm).

NMR spectra (^1H, ^{13}C) were recorded at room temperature in $CDCl_3$ on Bruker AM 300 MHz or Bruker AVANCE 400 MHz spectrometers. Chemical shifts (δ) are reported in ppm relative to the residual solvent signals (δ = 7.26 for ^1H, δ = 77.16 for ^{13}C). ^1H NMR data are reported as chemical shift (multiplicity, coupling constants, integration). The following abbreviations are used: s = singlet, d = doublet, t = triplet, q = quartet, m = multiplet, bs = broad signal.

IR spectra were recorded with a Tensor 27 (ATR diamond) Bruker spectrometer. IR was reported as characteristic bands (cm^{-1}). High-resolution mass spectra (HRMS) were recorded using a mass spectrometer MicroToF from Bruker with an electron spray ion source (ESI) or an atmospheric-pressure chemical ionization (APCI) and a ToF detector at Institut Parisien de Chimie Moléculaire.

2. Optimizations of reaction conditions (ketones and secondary alcohol)

2.1. General procedure A (1st step + 2nd step)

Table S1. Optimizations of reaction conditions (1st step)

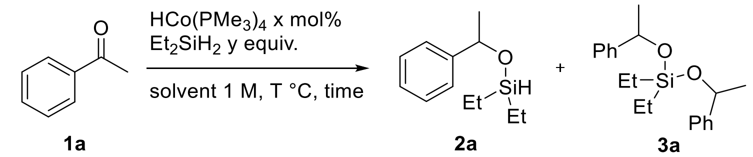

Entry	[Co] x mol%	Time/h	Solvent	T ºC	Et_2SiH_2 y equiv.	1a/2a/3a (%)[a]
**1**	5	16	THF	r.t	1.2	100/0/0
**2**	5	16	THF	90	1.2	69/31/0
**3**	5	16	Tol	r.t	1.2	34/20/46
**4**	5	16	Tol	110	1.2	0/11/89
**5**	**5**	**1**	**Tol**	**110**	**1.2**	**0/100/0**
**6**	5	1	Tol	150	1.2	0/79/21
**7**	3	16	Tol	110	1.2	0/91/9
**8**	5	16	Tol	110	1.05	53/27/20

[a] Ratio was measured by ^1H NMR spectroscopy of the crude mixture.

Table S2. Optimizations of reaction conditions (2nd step)

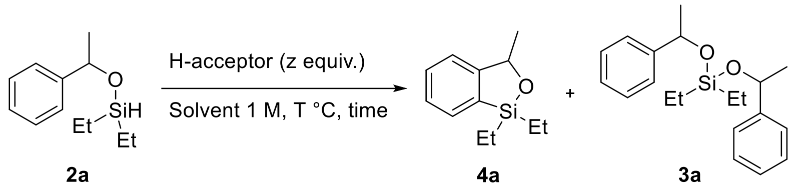

Entry	Time/h	T ºC	H acceptor (z equiv.)	2a/4a/3a (%)[a]	^1H NMR yield 4a (%)[b]
**1**	16	150	1.2 (TBE)	17/18/65	-
**2**	16	110	1.2 (NBE)	74/6/20	-
**3**	16	180	1.2 (NBE)	0/36/64	-
**4**	1	150	1.2 (NBE)	65/19/16	-
**5**	6	150	1.2 (NBE)	13/74/13	-
**6**	**16**	**150**	**1.2 (NBE)**	**0/90/10**	**82**
**7**	16	150	-	0/0/100	-

[a] Ratio measured by ^1H NMR spectroscopy of the crude mixture. [b] ^1H-NMR yield of **4a** calculated using 1,3,5-trimethoxybenzene as internal standard.

General procedure A (GP-A)

1st step. In an argon-filled glovebox, a dried V-shaped Biotage microwave vial equipped with a stirring bar was charged with the substrates (1 equiv), Et_2SiH_2 (1.2 equiv.), $HCo(PMe_3)_4$ (5 mol%), and toluene (1 M). The vial was sealed and the reaction mixture was stirred for 1 h at 110 °C. For the optimization, it was then filtered through a plug of silica/Celite® using AcOEt as eluent. Conversion and product ratios were determined by ^1H NMR analysis of the crude material.

2nd step. After completion of the hydrosilylation (as confirmed by ^1H NMR), the reaction mixture was directly (without isolation of **2a** by filtration) with a freshly prepared solution of norbornene (NBE) (1.2

equiv.) in toluene (1 M), prepared inside an argon-filled glovebox. The mixture was stirred for 16 h at 150 °C and then filtered through a plug of silica/Celite® using AcOEt as eluent. After evaporation of solvents, conversion and product ratios were determined by ^{1}H NMR analysis of the crude material and ^{1}H-NMR yield of **4a** was calculated using 1,3,5-trimethoxybenzene as internal standard.

2.2. General procedure B (one-pot)

Table S3. Optimizations of reaction conditions (one-pot)

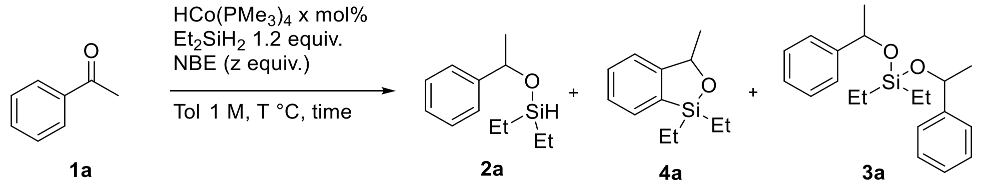

Entry	[Co] x mol%	Time/h	T °C	NBE (z equiv.)	2a/4a/3a (%)[a]	^{1}H NMR yield 4a (%)[b]
1	5.0	1	110	1.2	9/0/91	-
1	5.0	16	110	1.2	49/22/29	-
2	5.0	16	150	1.2	0/45/55	-
3	5.0	16	180	1.2	0/34/66	-
**5**	**5.0**	**16**	**150**	**2.0**	**0/86/14**	**59**
6	5.0	16	110	2.0	47/36/17	-
7	5.0	6	150	2.0	0/79/21	-
8	1.0	16	150	2.0	64/17/19	-

[a] Ratio measured by ^{1}H NMR spectroscopy of the crude mixture. [b] ^{1}H-NMR yield calculated using 1,3,5-trimethoxybenzene as internal standard.

General procedure B (GP-B)

In an argon-filled glovebox, a dried V-shaped Biotage microwave vial equipped with a stirring bar was charged with the substrates (0.5 mmol, 1.0 equiv.), $HCo(PMe_3)_4$ (5 mol%), Et_2SiH_2 (1.2 equiv), and NBE (1.2-2 equiv. depending of the ketone, see characterization section). After dissolution of all components in toluene (1 M), the vial was sealed and the reaction mixture was stirred for 16 h at 150 °C. It was then filtered through a plug of silica/Celite® using AcOEt as eluent. After evaporation of solvents, conversion and ratio were determined by ^{1}H NMR analysis of the crude material and ^{1}H-NMR yield of **4a** was calculated using 1,3,5-trimethoxybenzene as internal standard.

2.3. General procedure C (secondary alcohol)

Table S4. Optimizations of reaction conditions

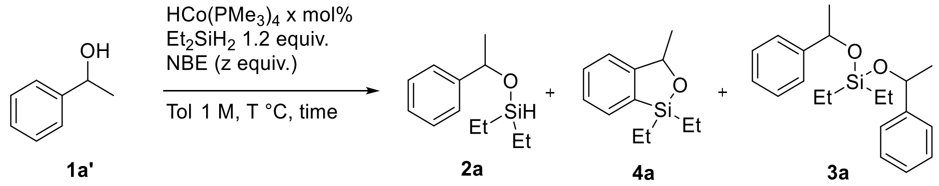

Entry	[Co] x mol%	y (equiv.)	z (equiv.)	T ℃	Time/h	2a/4a/3a (%)[a]	^{1}H NMR yield 4a (%)[a]
1	5	1.2	1.2	150	16	100/0/0	-
2	5	1.2	3.0	110	1	100/0/0	-
**3**	**5**	**1.2**	**3.0**	**150**	**16**	**0/67/33**	**67%**
4	5	1.5	3.0	150	16	0/67/33	67%
5	5	1.2	4.0	150	16	0/52/48	52%
6	5	-	3.0	150	16	n.r.	-
7	5	1.2	-	150	16	n.r.	-
8	-	1.2	3.0	150	16	n.r.	-

[a] Ratio and ^{1}H-NMR yield were calculated using 1,3,5-trimethoxybenzene as internal standard. n.r. no reaction.

General procedure C (GP-C)

In an argon-filled glovebox, a dried V-shaped Biotage microwave vial equipped with a stirring bar was charged with the substrates (0.5 mmol, 1.0 equiv.), $HCo(PMe_3)_4$ (5 mol%), Et_2SiH_2 (1.2 equiv), and NBE (3 equiv.). After dissolution of all components in toluene (1 M), the vial was sealed and the reaction mixture was stirred for 16 h at 150 °C. It was then filtered through a plug of silica/Celite® using AcOEt as eluent. After evaporation of solvents, conversion and ratio were determined by ^{1}H NMR analysis of the crude material and ^{1}H-NMR yield of **4a** was calculated using 1,3,5-trimethoxybenzene as internal standard.

2.4. ^{1}H-NMR study: role of the NBE

Sequential procedure from ketone:

In a glovebox in a J-Young tube was added 4.6 mg of $HCo(PMe_3)_4$ (5 mol%), 30 mg of acetophenone (0.25 mmol , 1 eq.), 39 uL of diethylsilane (0.3 mmol, 1.2 eq.) and 0.5 mL of Tol-d8. The tube was heated at 110°C for 1 hour (1st step). Then in the glove box , was added 23.5 mg of Norbonene (0.25 mmol, 1 eq.) and the tube was heated at 150°C for 16 hours (2nd Step).

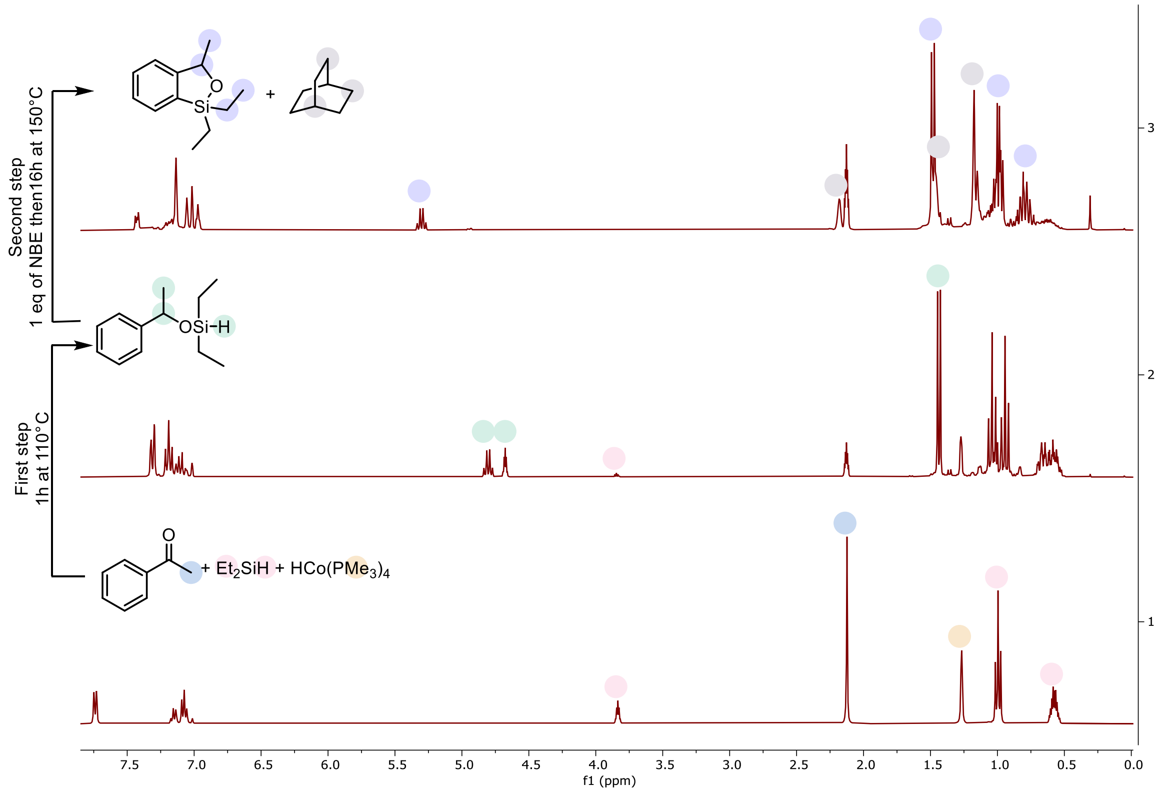

Figure S1 : NMR monitoring of the two steps procedure in a J-Young NMR tube showing the formation of the norbornane.

2.5. Characterizations of final products

2-(Diethyl(1-phenylethoxy)silane) 2a. Following **GP-A-1st step** from acetophenone (60 mg, 0.5 mmol, 1.0 equiv.). After 1 h at 110 °C and filtration on a plug of silica/Celite®, **2a** was obtained without further purification. **^{1}H NMR** (400 MHz, $CDCl_3$) δ 7.36-7.30 (m, 4H), 7.25-7.22 (m, 1H), 4.87 (q, J = 6.4 Hz, 1H), 4.43-4.41 (m, 1H), 1.47 (d, J = 6.3 Hz, 3H), 1.01-0.65 (m, 10H).

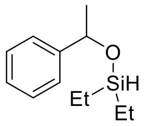

Chemical Formula: $C_{12}H_{20}OSi$
Molecular Weight: 208.38

3-(Diethylbis(1-phenylethoxy)silane) 3a. Following **GP-A-1st step** from acetophenone (60 mg, 0.5 mmol, 1.0 equiv.). ^{1}H NMR of the crude material **3a** was obtained without further purification. **^{1}H NMR** (400 MHz, $CDCl_3$) δ 7.38-7.27 (m, 8H), 7.24-7.19 (m, 2H), 4.93 (q, J = 6.4 Hz, 2H), 1.42 (d, J = 6.4 Hz, 3H), 1.32 (d, J = 6.4 Hz, 3H), 1.00-0.6 (m, 10H).

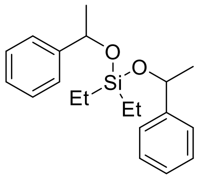

Chemical Formula: $C_{20}H_{28}O_2Si$
Molecular Weight: 328.53

1,1-Diethyl-3-methyl-1,3-dihydrobenzo[c][1,2]oxasilole 4a. Following **GP-A** from acetophenone **1a**

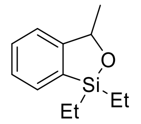

Chemical Formula: $C_{12}H_{18}OSi$
Molecular Weight: 206.36

(60 mg, 0.50 mmol, 1.0 equiv.), the crude material was analyzed by ^{1}H NMR using 1,3,5-trimethoxybenzene as an internal standard, which indicated an 82% NMR yield. Purification by silica gel (99:1 hexanes/EtOAc) afforded **4a** as a clear oil (80 mg, 78%). Following **GP-B** from acetophenone **1a** (60 mg, 0.50 mmol, 1 equiv.) and 2 equivalents of NBE, the NMR yield was 59%, and the isolated yield was 55% (57 mg). Following **GP-C** from alcohol **1a'** (61 mg, 0.50 mmol, 1 equiv.) the NMR yield was 67%, and the isolated yield was 65% (67 mg). **^{1}H NMR** (400 MHz, $CDCl_3$) δ 7.55 (d, *J* = 7.2 Hz, 1H), 7.40 (td, *J* = 7.5, 1.3 Hz, 1H), 7.29 (t, *J* = 7.2 Hz, 1H), 7.22 (d, *J* = 7.7 Hz, 1H), 5.34 (q, *J* = 6.5 Hz, 1H), 1.51 (d, *J* = 6.4 Hz, 3H), 0.99-0.91 (m, 6H), 0.89-0.79 (m, 4H); **^{13}C NMR** (100 MHz, $CDCl_3$) δ 155.1, 133.1, 131.6, 129.7, 126.9, 122.3, 78.2, 25.3, 7.3, 7.2, 6.8, 6.6. This is a known compound, and the spectroscopic data is in agreement with the literature.[36]

1,1-Diethyl-3,6-dimethyl-1,3-dihydrobenzo[c][1,2]oxasilole 4b. Following **GP-A** from 4'-

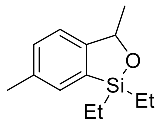

Chemical Formula: $C_{13}H_{20}OSi$
Molecular Weight: 220.39

methylacetophenone **1b** (67 mg, 0.50 mmol, 1.0 equiv.), the crude material was analyzed by ^{1}H NMR using 1,3,5-trimethoxybenzene as an internal standard, which indicated an 78% NMR yield. Purification by silica gel (99:1 hexanes/EtOAc) afforded **4b** as a clear oil (82 mg, 75%). Following **GP-B** from 4'-methylacetophenone **1b** (67 mg, 0.50 mmol, 1 equiv.) and 2 equivalents of NBE, the NMR yield was 53%, and the isolated yield was 51% (56 mg). Following **GP-C** from alcohol **1b'** (68 mg, 0.50 mmol, 1 equiv.) the NMR yield was 63%, and the isolated yield was 60% (66 mg). **^{1}H NMR** (400 MHz, $CDCl_3$) δ 7.35 (s, 1H), 7.21 (d, *J* = 1.7 Hz, 1H), 7.10 (d, *J* = 7.9 Hz, 1H), 5.31 (q, *J* = 6.4 Hz, 1H), 2.38 (s, 3H), 1.49 (d, *J* = 6.5 Hz, 3H), 0.99-0.92 (m, 6H), 0.87-0.78 (m, 4H); **^{13}C NMR** (100 MHz, $CDCl_3$) δ 152.4, 136.3, 133.3, 131.9, 130.8, 122.0, 78.1, 25.4, 21.3, 7.3, 7.2, 6.8, 6.6; **IR (neat, cm^{-1})**: 2957, 2876, 1462, 1317, 1267, 1235, 1084, 1065, 924, 818; **HRMS** (ESI) Calcd for $[C_{13}H_{21}OSi]^+$: *m/z* 221.1356, found 221.1357.

1,1-Diethyl-3,4-dimethyl-1,3-dihydrobenzo[c][1,2]oxasilole 4c. Following **GP-A** from 2'-

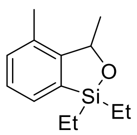

Chemical Formula: $C_{13}H_{20}OSi$
Molecular Weight: 220.39

methylacetophenone **1c** (67 mg, 0.50 mmol, 1.0 equiv.), the crude material was analyzed by ^{1}H NMR using 1,3,5-trimethoxybenzene as an internal standard, which indicated an 61% NMR yield. Purification by silica gel (99:1 hexanes/EtOAc) afforded **4c** as a clear oil (65 mg, 59%). Following **GP-B** from 2'-methylacetophenone **1c** (67 mg, 0.50 mmol, 1 equiv.) and 2 equivalents of NBE, the NMR yield was 75%, and the isolated yield was 72% (79 mg). **^{1}H NMR** ($CDCl_3$, 300 MHz) δ 7.39 (d, *J* = 6.8 Hz, 1H), 7.25–7.21 (m, 1H), 7.20–7.17 (m, 1H), 5.41 (q, *J* = 6.3 Hz, 1H), 2.31 (s, 3H), 1.49 (d, *J* = 6.3 Hz, 3H), 1.03 (t, *J* = 7.6 Hz, 3H), 0.95–0.77 (m, 7H); **^{13}C NMR** ($CDCl_3$, 100 MHz) δ

153.4, 133.1, 131.8, 131.7, 129.2, 127.3, 77.9, 24.3, 19.4, 7.8, 7.1, 7.0, 6.5. This is a known compound, and the spectroscopic data is in agreement with the literature.[37]

1,1-Diethyl-3,5-dimethyl-1,3-dihydrobenzo[c][1,2]oxasilole 4d. Following **GP-A** from 3'-methylacetophenone **1d** (67 mg, 0.50 mmol, 1.0 equiv.), the crude material was analyzed by ^{1}H NMR using 1,3,5-trimethoxybenzene as an internal standard, which indicated an 58% NMR yield. Purification by silica gel (99:1 hexanes/EtOAc) afforded **4d** as a clear oil (61 mg, 55%). Following **GP-B** from 3'-methylacetophenone **1d** (67 mg, 0.50 mmol, 1 equiv.) and 2 equivalents of NBE, the NMR yield was 71%, and the isolated yield was 69% (76 mg). **^{1}H NMR** ($CDCl_3$, 400 MHz) δ 7.46 (d, *J* = 7.4 Hz, 1H), 7.15 (d, *J* = 7.4 Hz, 1H), 7.04 (s, 1H), 5.32 (q, *J* = 6.5 Hz, 1H), 2.40 (s, 3H), 1.51 (d, *J* = 6.5 Hz, 3H), 0.96 (t, *J* = 6.8 Hz, 3H), 0.93 (t, *J* = 6.4 Hz, 3H), 0.87–0.75 (m, 4H); **^{13}C NMR** ($CDCl_3$, 100 MHz) δ 155.6, 139.8, 131.4, 129.6, 128.1, 122.9, 78.1, 25.3, 21.8, 7.4, 7.3, 6.8, 6.6. This is a known compound, and the spectroscopic data is in agreement with the literature.[36]

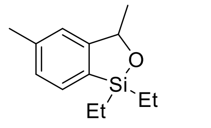

Chemical Formula: $C_{13}H_{20}OSi$
Molecular Weight: 220.39

6-(Tert-butyl)-1,1-diethyl-3-methyl-1,3-dihydrobenzo[c][1,2]oxasilole 4e. Following **GP-A** from 1-(4-(tert-butyl)phenyl)ethan-1-one **1e** (88 mg, 0.50 mmol, 1.0 equiv.), the crude material was analyzed by ^{1}H NMR using 1,3,5-trimethoxybenzene as an internal standard, which indicated an 64% NMR yield. Purification by silica gel (99:1 hexanes/EtOAc) afforded **4e** as a clear oil (82 mg, 62%). Following **GP-B** from 1-(4-(tert-butyl)phenyl)ethan-1-one **1e** (88 mg, 0.50 mmol, 1 equiv.) and 2 equivalents of NBE, the NMR yield was 60%, and the isolated yield was 57% (75 mg). **^{1}H NMR** (300 MHz, $CDCl_3$) δ 7.56 (d, *J* = 2.0 Hz, 1H), 7.47 (dd, *J* = 8.2, 2.0 Hz, 1H), 7.17 (d, *J* = 8.1 Hz), 5.34 (q, *J* = 6.5 Hz, 1H), 1.53 (d, *J* = 6.5 Hz, 3H), 1.38 (s, 9H), 1.02-0.96 (m, 6H), 0.90-0.81 (m, 4H); **^{13}C NMR** (100 MHz, $CDCl_3$) δ 152.3, 149.6, 133.0, 127.8, 127.2, 121.8, 78.0, 34.7, 31.7, 25.2, 7.4, 7.2, 6.9, 6.7; **IR (neat, cm^{-1})**: 3052, 2959, 2875, 1460, 1363, 1259, 1117, 1083, 1063, 1020, 924, 808; **HRMS** (APCI) Calcd for $[C_{16}H_{27}OSi]^+$: *m/z* 263.1826, found 263.1826.

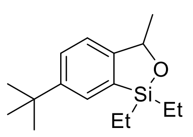

Chemical Formula: $C_{16}H_{26}OSi$
Molecular Weight: 262.47

1,1-Diethyl-3-methyl-6-phenyl-1,3-dihydrobenzo[c][1,2]oxasilole 4f. Following **GP-A** from 4-acetylbiphenyl **1f** (98 mg, 0.50 mmol, 1.0 equiv.), the crude material was analyzed by ^{1}H NMR using 1,3,5-trimethoxybenzene as an internal standard, which indicated an 80% NMR yield. Purification by silica gel (99:1 hexanes/EtOAc) afforded **4f** as a clear oil (77 mg, 62%). Following **GP-B** from 4-acetylbiphenyl **1f** (98 mg, 0.50 mmol, 1 equiv.) and 1.2 equivalents of NBE, the NMR yield was 73%, and the isolated yield was 71% (100 mg). **^{1}H NMR** (400 MHz, $CDCl_3$) δ

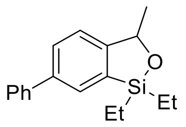

Chemical Formula: $C_{18}H_{22}OSi$
Molecular Weight: 282.46

7.74 (d, J = 1.8 Hz, 1H), 7.64-7.60 (m, 3H), 7.47-7.43 (m, 2H), 7.37-7.33 (m, 1H), 7.29 (d, J = 8.0 Hz, 1H), 5.39 (q, J = 6.5 Hz, 1H), 1.55 (d, J = 6.4 Hz, 3H), 1.02-0.95 (m, 6H), 0.92-0.81 (m, 4H); **^{13}C NMR** (100 MHz, $CDCl_3$) δ 154.3, 141.4, 140.0, 134.0, 130.1, 129.1, 128.9, 127.4, 127.3, 122.6, 78.1, 25.3, 7.3, 7.2, 6.9, 6.6. This is a known compound, and the spectroscopic data is in agreement with the literature. [37]

1,1-Diethyl-6-fluoro-3-methyl-1,3-dihydrobenzo[c][1,2]oxasilole 4g. Following **GP-A** from 4-fluoroacetophenone **1g** (69 mg, 0.50 mmol, 1.0 equiv.), the crude material was analyzed by ^{1}H NMR using 1,3,5-trimethoxybenzene as an internal standard, which indicated an 75% NMR yield. Purification by silica gel (99:1 hexanes/EtOAc) afforded **4g** as a clear oil (82 mg, 73%). Following **GP-B** from 4-fluoroacetophenone **1g** (98 mg, 0.50 mmol, 1 equiv.) and 2 equivalents of NBE, the NMR yield was 66%, and the isolated yield was 62% (69 mg). **^{1}H NMR** (400 MHz, $CDCl_3$) δ 7.19-7.15 (m, 2H), 7.07 (td, J = 8.7, 2.5 Hz, 1H), 5.30 (q, J = 6.4 Hz, 1H), 1.49 (d, J = 6.5 Hz, 3H), 0.98-0.91 (m, 6H), 0.88-0.77 (m, 4H); **^{13}C NMR** (100 MHz, $CDCl_3$) δ 162.3 (d, J_F = 246.2 Hz), 150.5 (d, J_F = 2.4 Hz), 135.9 (d, J_F = 5.3 Hz), 123.8 (d, J_F = 7.5 Hz), 117.2 (d, J_F = 19.5 Hz), 117.1 (d, J_F = 22.8 Hz), 77.9, 25.4, 7.2, 7.1, 6.7, 6.5; **^{19}F NMR** (376 MHz, $CDCl_3$) δ -107.7. This is a known compound, and the spectroscopic data is in agreement with the literature.[37]

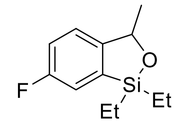

Chemical Formula: $C_{12}H_{17}FOSi$
Molecular Weight: 224.35

1,1-Diethyl-3-methyl-6-(trifluoromethyl)-1,3-dihydrobenzo[c][1,2]oxasilole 4h. Following **GP-A** from 4'-(trifluoromethyl)-acetophenone **1h** (94 mg, 0.50 mmol, 1.0 equiv.), the crude material was analyzed by ^{1}H NMR using 1,3,5-trimethoxybenzene as an internal standard, which indicated an 68% NMR yield. Purification by silica gel (99:1 hexanes/EtOAc) afforded **4h** as a clear oil (88 mg, 64%). Following **GP-B** from 4'-(trifluoromethyl)-acetophenone **1h** (94 mg, 0.50 mmol, 1 equiv.) and 2 equivalents of NBE, the NMR yield was 55%, and the isolated yield was 51% (70 mg). **^{1}H NMR** (400 MHz, $CDCl_3$) δ 7.78 (s, 1H), 7.64 (d, J = 9.9 Hz, 1H), 7.31 (d, J = 8.1 Hz, 1H), 5.37 (q, J = 6.6 Hz, 1H), 1.52 (d, J = 6.6 Hz, 3H), 0.99-0.90 (m, 6H), 0.89-0.80 (m, 4H); **^{13}C NMR** (100 MHz, $CDCl_3$) δ 158.9, 134.4, 129.5 (q, J_F = 32.8 Hz), 128.3 (q, J_F = 3.8 Hz), 126.8 (q, J_F = 3.6 Hz), 124.6 (q, J_F = 272.6 Hz), 122.6, 78.1, 25.0, 7.2, 7.1, 6.7, 6.5; **^{19}F NMR** (376 MHz, $CDCl_3$) δ -63.3. This is a known compound, and the spectroscopic data is in agreement with the literature. [37]

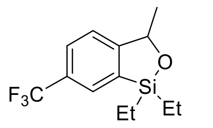

Chemical Formula: $C_{13}H_{17}F_3OSi$
Molecular Weight: 274.36

1,1-Diethyl-6-methoxy-3-methyl-1,3-dihydrobenzo[c][1,2]oxasilole 4i. Following **GP-A** from 4'-methoxyacetophenone **1i** (75 mg, 0.50 mmol, 1.0 equiv.), the crude material was analyzed by ^{1}H NMR using 1,3,5-trimethoxybenzene as an internal standard, which indicated an 75% NMR yield. Purification by silica gel (99:1 hexanes/EtOAc) afforded **4i** as a clear oil (85 mg, 72%). Following **GP-B** from 4' methoxyacetophenone **1i** (75 mg, 0.50 mmol, 1 equiv.) and 2 equivalents of NBE, the NMR yield was 60%, and the isolated yield was 58% (68 mg). Following **GP-C** from alcohol **1i'** (76 mg, 0.50 mmol, 1 equiv.) the NMR yield was 60%, and the isolated yield was 58% (68mg). **^{1}H NMR** (400 MHz, $CDCl_3$) δ 7.13 (d, *J* = 8.4 Hz, 1H), 7.02 (d, *J* = 2.5 Hz, 1H), 6.96 (dd, *J* = 8.4, 2.6 Hz, 1H), 5.29 (q, *J* = 6.4 Hz, 1H), 3.83 (s, 3H), 1.48 (d, *J* = 6.4 Hz, 3H), 0.99-0.92 (m, 6H), 0.87-0.78 (m, 4H); **^{13}C NMR** (75 MHz, $CDCl_3$) δ 158.8, 147.4, 134.8, 123.2, 116.5, 115.3, 77.9, 55.5, 25.5, 7.3, 7.2, 6.8, 6.6; **HRMS** (APCI) Calcd for $[C_{13}H_{21}O_2Si]^+$: *m/z* 237.1305, found 237.1306. This is a known compound, and the spectroscopic data is in agreement with the literature. [37]

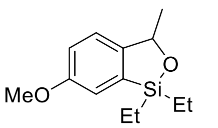

Chemical Formula: $C_{13}H_{20}O_2Si$
Molecular Weight: 236.39

1,1-Diethyl-3-methyl-1,3-dihydronaphtho[2,3-c][1,2]oxasilole 4j. Following **GP-A** from 2-acetonaphthone **1j** (85 mg, 0.50 mmol, 1.0 equiv.), the crude material was analyzed by ^{1}H NMR using 1,3,5-trimethoxybenzene as an internal standard, which indicated an 82% NMR yield. Purification by silica gel (99:1 hexanes/EtOAc) afforded **4j** as a clear oil (100 mg, 88%). Following **GP-B** from 2-acetonaphthone **1j** (85 mg, 0.50 mmol, 1.0 equiv.) and 1.2 equivalents of NBE, the NMR yield was 82%, and the isolated yield was 78% (100 mg). Following **GP-C** from alcohol **1j'** (86 mg, 0.50 mmol, 1 equiv.) the NMR yield was 57%, and the isolated yield was 55% (70mg). **^{1}H NMR** (400 MHz, $CDCl_3$) δ 8.08 (s, 1H), 7.89-7.82 (m, 2H), 7.65 (s, 1H), 7.52-7.43 (m, 2H), 5.51 (dd, *J* = 6.5, 1.2 Hz, 1H), 1.62 (d, *J* = 6.5 Hz, 3H), 1.00-0.95 (m, 6H), 0.92-0.85 (m, 4H); **^{13}C NMR** (100 MHz, $CDCl_3$) δ 151.4, 134.5, 132.9, 132.3, 132.2, 128.3, 128.1, 126.7, 125.6, 120.2, 77.9, 25.7, 7.34, 7.28, 6.8, 6.6; **IR (neat, cm^{-1})**: 3053, 2957, 2875, 1676, 1459, 1367, 1294, 1087, 1039, 1004, 923, 881; **HRMS** (APCI) Calcd for $[C_{16}H_{21}OSi]^+$: *m/z* 257.1356, found 257.1357.

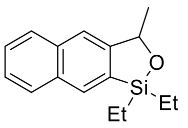

Chemical Formula: $C_{16}H_{20}OSi$
Molecular Weight: 256.42

1,1,5,5-Tetraethyl-3,7-dimethyl-5,7-dihydro-1H,3H-benzo[1,2-c:4,5-c']bis([1,2]oxasilole) 4k. Following **GP-A** from 1,4-diacetylbenzene **1k** (81 mg, 0.50 mmol, 1.0 equiv.) and 2.2 equivalents of silane and NBE, the crude material was analyzed by ^{1}H NMR using 1,3,5-trimethoxybenzene as an internal standard, which indicated an 50% NMR yield. Purification by silica gel (99:1 hexanes/EtOAc) afforded **4k** as a clear oil (100 mg, 88%). Following **GP-B** from 1,4-diacetylbenzene **1k** (85 mg, 0.50 mmol, 1.0 equiv.) and 2.2 equivalents of NBE and silane, the NMR

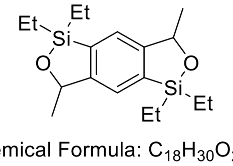

Chemical Formula: $C_{18}H_{30}O_2Si_2$
Molecular Weight: 334.61

yield was 60%, and the isolated yield was 56% (94mg). **^{1}H NMR** (300 MHz, $CDCl_3$) δ 7.34 (s, 2H), 5.35 (q, *J* = 6.4 Hz, 1H), 1.53 (d, *J* = 6.5 Hz, 3H), 0.99-0.93 (m, 12H), 0.90-0.82 (m, 8H); **^{13}C NMR** (75 MHz, $CDCl_3$) δ 153.54, 153.5, 135.5, 124.8, 78.1, 78.07, 25.4, 7.3, 7.1, 6.9, 6.8, 6.64, 6.6; **IR (neat, cm^{-1})**: 2957, 2876, 1678, 1461, 1368, 1239, 1087, 1007, 925; **HRMS** (APCI) Calcd for $[C_{18}H_{31}O_2Si_2]^+$: *m/z* 335.1857, found 335.1853.

1,1,3-Triethyl-1,3-dihydrobenzo[c][1,2]oxasilole 4l. Following **GP-A** from propiophenone **1l** (67 mg, 0.50 mmol, 1.0 equiv.), the crude material was analyzed by ^{1}H NMR using 1,3,5-trimethoxybenzene as an internal standard, which indicated an 65% NMR yield. Purification by silica gel (99:1 hexanes/EtOAc) afforded **4l** as a clear oil (66 mg, 60%). Following **GP-B** from propiophenone **1l** (67 mg, 0.50 mmol, 1.0 equiv.) and 1.2 equivalents of NBE, the NMR yield was 60%, and the isolated yield was 57% (63 mg). **^{1}H NMR** (300 MHz, $CDCl_3$) δ 7.55 (d, *J* = 7.1 Hz, 1H), 7.39 (t, *J* = 7.1 Hz, 1H), 7.29 (t, *J* = 7.2 Hz, 1H), 7.21 (d, *J* = 7.7 Hz, 1H), 5.19 (dd, *J* = 7.6, 3.5 Hz, 1H), 2.03-1.95 (m, 1H), 1.70-1.58 (m, 1H), 1.03-0.96 (m, 6H), 0.92-0.89 (m, 3H), 0.86-0.79 (m, 4H); **^{13}C NMR** (75 MHz, $CDCl_3$) δ 153.8, 133.9, 131.6, 129.6, 126.8, 122.3, 83.1, 31.8, 9.7, 7.3, 7.1, 6.9, 6.6. This is a known compound, and the spectroscopic data is in agreement with the literature.[36]

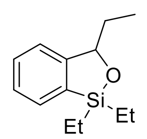

Chemical Formula: $C_{13}H_{20}OSi$
Molecular Weight: 220.39

1,1-Diethyl-3-propyl-1,3-dihydrobenzo[c][1,2]oxasilole 4m. Following **GP-A** from butyrophenone **1m** (74 mg, 0.50 mmol, 1.0 equiv.), the crude material was analyzed by ^{1}H NMR using 1,3,5-trimethoxybenzene as an internal standard, which indicated an 65% NMR yield. Purification by silica gel (99:1 hexanes/EtOAc) afforded **4m** as a clear oil (73 mg, 62%). Following **GP-B** from butyrophenone **1m** (74 mg, 0.50 mmol, 1.0 equiv.) and 1.2 equivalents of NBE, the NMR yield was 71%, and the isolated yield was 69% (81mg). **^{1}H NMR** (300 MHz, $CDCl_3$) δ 7.55 (d, *J* = 7.1 Hz, 1H), 7.39 (t, *J* = 7.4 Hz, 1H), 7.30 (t, *J* = 7.2 Hz, 1H), 7.22 (d, *J* = 7.7 Hz, 1H), 5.25-5.22 (m, 1H), 1.95-1.84 (m, 1H), 1.65-1.44 (m, 3H), 1.03-0.91 (m, 9H), 0.89-0.79 (m, 4H); **^{13}C NMR** (100 MHz, $CDCl_3$) δ 154.2, 133.7, 131.6, 129.6, 126.8, 122.3, 81.9, 41.6, 18.7, 14.3, 7.4, 7.2, 6.9, 6.6. This is a known compound, and the spectroscopic data is in agreement with the literature.[36]

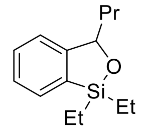

Chemical Formula: $C_{14}H_{22}OSi$
Molecular Weight: 234.41

3-butyl-1,1-diethyl-1,3-dihydrobenzo[c][1,2]oxasilole 4n. Following **GP-A** from valerophenone **1n** (81 mg, 0.50 mmol, 1.0 equiv.), the crude material was analyzed by ^{1}H NMR using 1,3,5-trimethoxybenzene as an internal standard, which indicated an 63% NMR yield. Purification by silica gel (99:1 hexanes/EtOAc) afforded **4n** as a clear oil (76 mg, 61%). Following **GP-B** from valerophenone **1n** (81 mg, 0.50 mmol, 1.0 equiv.) and 2 equivalents of NBE, the NMR yield was 68%, and the isolated yield was 65% (81 mg). **^{1}H NMR** (400 MHz, $CDCl_3$) δ 7.55 (d, *J* = 7.2 Hz, 1H), 7.39 (td, *J* = 7.5, 1.3 Hz, 1H), 7.29 (t, *J* = 7.3 Hz, 1H), 7.22 (d, *J* = 7.7 Hz, 1H), 5.22 (dd, *J* = 8.0, 3.4 Hz, 1H), 1.95-1.88 (m, 1H), 1.64-1.56 (m, 1H), 1.53-1.31 (m, 4H), 1.02-0.98 (m, 3H), 0.95-0.89 (m, 6H), 0.88-0.80 (m, 4H); **^{13}C NMR** (100 MHz, $CDCl_3$) δ 154.2, 133.7, 131.6, 129.6, 126.8, 122.3, 82.1, 39.1, 27.7, 22.9, 14.2, 7.4, 7.2, 6.9, 6.6; **IR (neat, cm^{-1})**: 3058, 2957, 2875, 1683, 1459, 1262, 1080, 1052, 1013, 916; **HRMS** (ESI) Calcd for $[C_{15}H_{25}OSi]^+$: *m/z* 249.1669, found 249.1668.

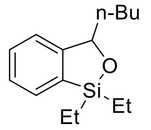

Chemical Formula: $C_{15}H_{24}OSi$
Molecular Weight: 248.44

1,1-Diethyl-3-isopropyl-1,3-dihydrobenzo[c][1,2]oxasilole 4o. Following **GP-A** from isobutyrophenone **1o** (74 mg, 0.50 mmol, 1.0 equiv.), the crude material was analyzed by ^{1}H NMR using 1,3,5-trimethoxybenzene as an internal standard, which indicated an 60% NMR yield. Purification by silica gel (99:1 hexanes/EtOAc) afforded **4o** as a clear oil (74 mg, 55%). Following **GP-B** from isobutyrophenone **1o** (74 mg, 0.50 mmol, 1.0 equiv.) and 1.2 equivalents of NBE, the NMR yield was 65%, and the isolated yield was 63% (74 mg). **^{1}H NMR** (300 MHz, $CDCl_3$) δ 7.56 (d, *J* = 7.2 Hz, 1H), 7.39 (td, *J* = 7.5, 1.3 Hz, 1H), 7.29 (t, *J* = 7.2 Hz, 1H), 7.22 (d, *J* = 7.7 Hz, 1H), 5.15 (d, *J* = 2.7 Hz, 1H), 2.17-2.10 (m, 1H), 1.19 (d, *J* = 6.9 Hz, 3H), 1.08 (t, *J* = 7.9 Hz, 3H), 0.93-0.81 (m, 7H), 0.63 (d, *J* = 6.8 Hz, 3H); **^{13}C NMR** (75 MHz, $CDCl_3$) δ 153.0, 134.7, 131.5, 129.6, 126.8, 122.4, 86.6, 34.6, 20.4, 15.1, 7.2, 7.0, 6.9, 6.6. This is a known compound, and the spectroscopic data is in agreement with the literature. [37]

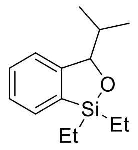

Chemical Formula: $C_{14}H_{22}OSi$
Molecular Weight: 234.41

1,1-Diethyl-3-phenyl-1,3-dihydrobenzo[c][1,2]oxasilole 4p. Following **GP-A** from benzophenone **1p** (91 mg, 0.50 mmol, 1.0 equiv.), the crude material was analyzed by ^{1}H NMR using 1,3,5-trimethoxybenzene as an internal standard, which indicated an 52% NMR yield. Purification by silica gel (99:1 hexanes/EtOAc) afforded **4p** as a clear oil (67 mg, 50%). Following **GP-B** from isobutyrophenone **1p** (74 mg, 0.50 mmol, 1.0 equiv.) and 2 equivalents of NBE, the NMR yield was 70%, and the isolated yield was 66% (88 mg). Following **GP-C** from alcohol **1p'** (92 mg, 0.50 mmol, 1 equiv.) the NMR yield was 62%, and the isolated yield was 59% (79 mg). **^{1}H NMR** (300 MHz, $CDCl_3$) δ 7.63-7.60 (m, 1H), 7.36-7.28 (m, 7H), 7.03-7.01 (m, 1H), 6.16 (s, 1H), 1.11-0.99 (m, 6H), 0.96-0.86 (m, 4H); **^{13}C NMR** (75 MHz, $CDCl_3$)

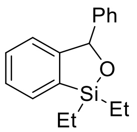

Chemical Formula: $C_{17}H_{20}OSi$
Molecular Weight: 268.43

δ 153.2, 144.0, 133.7, 131.4, 129.9, 128.6, 128.0, 127.5, 127.1, 123.9, 84.4, 7.4, 7.1, 7.0, 6.6. This is a known compound, and the spectroscopic data is in agreement with the literature.[36]

1,1-diethyl-6-methyl-3-(p-tolyl)-1,3-dihydrobenzo[c][1,2]oxasilole 4q. Following **GP-A** from 4,4'-dimethylbenzophenone **1q** (105 mg, 0.50 mmol, 1.0 equiv.), the crude material was analyzed by ^{1}H NMR using 1,3,5-trimethoxybenzene as an internal standard, which indicated an 70% NMR yield. Purification by silica gel (99:1 hexanes/EtOAc) afforded **4q** as a clear oil (101mg, 68%). Following **GP-B** from 4,4'-dimethylbenzophenone **1q** (105 mg, 0.50 mmol, 1.0 equiv.) and 2 equivalents of NBE, the NMR yield was 53%, and the isolated yield was 50% (74 mg). **^{1}H NMR** (300 MHz, $CDCl_3$) δ 7.40 (s, 1H), 7.18-7.12 (m, 5H), 6.90 (d, *J* = 7.9 Hz, 1H), 6.10 (s, 1H), 2.38 (s, 3H), 2.33 (s, 3H), 1.10-0.96 (m, 6H), 0.93-0.82 (m, 4H); **^{13}C NMR** (75 MHz, $CDCl_3$) δ 150.7, 141.3, 137.5, 136.5, 133.9, 131.6, 130.9, 129.3, 127.5, 123.7, 84.1, 21.3, 7.4, 7.1, 7.0, 6.7; **IR (neat, cm^{-1})**: 3024, 2955, 2875, 1903, 1725, 1643, 1608, 1513, 1462, 1412, 1268, 1181, 1140, 1016, 826; **HRMS** (ESI) Calcd for $[C_{19}H_{25}OSi]^+$: *m/z* 297.1669, found 297.1668.

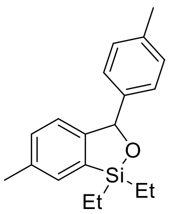

Chemical Formula: $C_{19}H_{24}OSi$
Molecular Weight: 296.49

3. Optimizations of reaction conditions (biphenyls)

3.1. General Procedure D

Table S5. Optimizations of reaction conditions

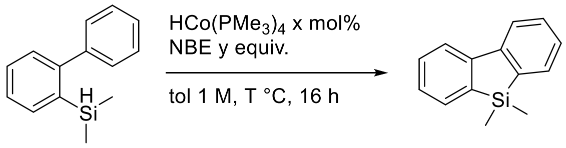

5a **6a**

Entry	[Co] x mol%	y equiv.	T ℃	^{1}H NMR yield -a (%)[a]
**1**	5	1.2	110	89%
**2**	**5**	**1.2**	**150**	**100%**
**3**	1	1.2	150	38%
**4**	5	-	150	59%
**5**	-	1.2	150	-

[a] ^{1}H-NMR yield calculated using 1,3,5-trimethoxybenzene as internal standard.

General procedure D (GP-D)

In an argon-filled glovebox, a dried V-shaped Biotage microwave vial equipped with a stirring bar was charged with $HCo(PMe_3)_4$ (5 mol%) and NBE (1.2 equiv.). A separately prepared, degassed solution of the corresponding biphenyl derivative **5** (1.0 equiv.) in toluene (1M) was then transferred to this mixture. The sealed reaction vessel was stirred for 16 h at 150°C. The reaction was quenched with petroleum ether

and the resulting mixture was filtered through a plug of silica/Celite® using AcOEt as eluent. After evaporation of solvents, the crude product was purified by flash chromatography leading to silacycle **6**.

3.2. ^{1}H-NMR study: role of the NBE

In a glovebox in a J-Young tube was added 1.5 mg of $HCo(PMe_3)_4$ (5 mol%), 17.5 mg of silane **5a** (0.082 mmol , 1 eq.), 9.5 mg of Norbonene (0.098 mmol, 1.2 eq.) and 0.5 mL of Tol-d8. The tube was heated at 150°C for 24 hours.

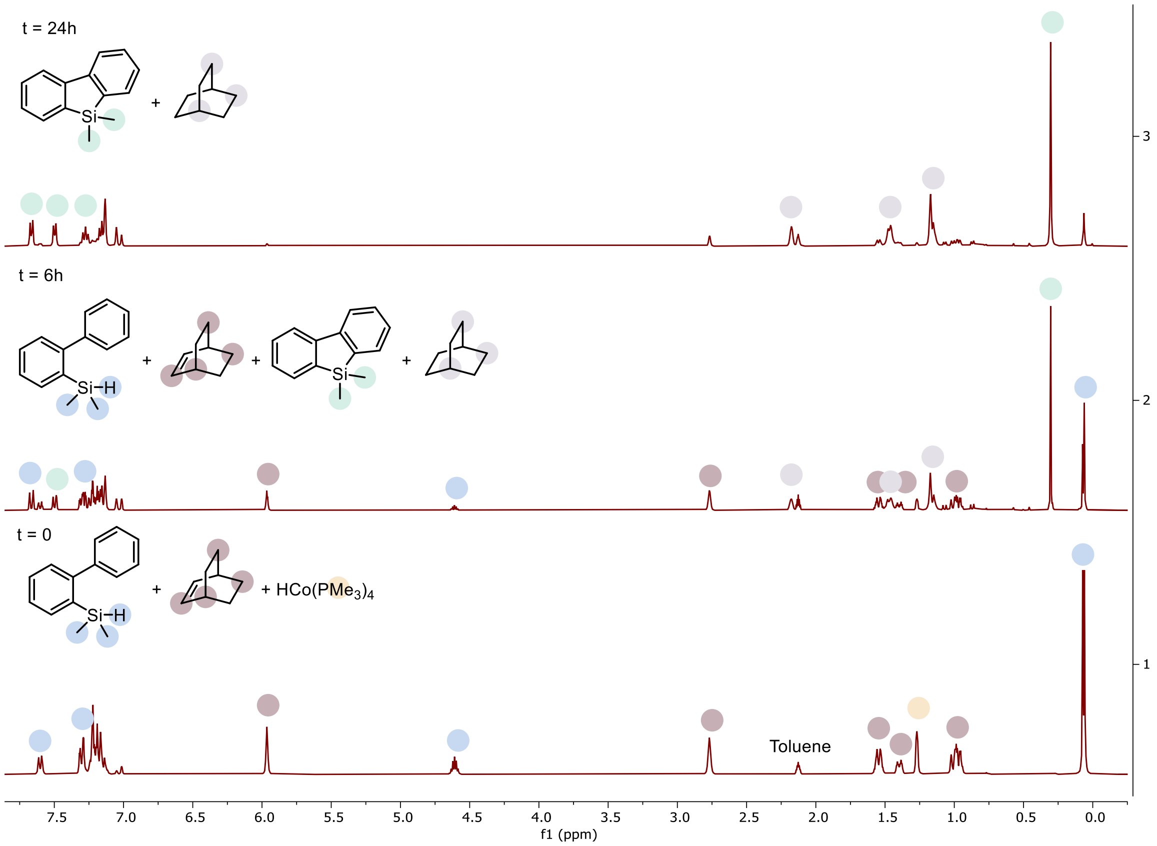

Figure S2 : NMR monitoring of the one step procedure in a J-Young NMR tube showing the formation of the norbornane starting from silane **5a**.

3.3. Synthesis and characterizations of starting materials 5

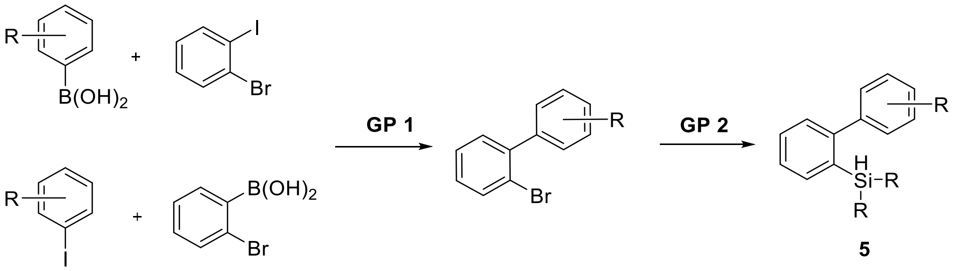

General Procedure 1 (GP 1)

To a 50 mL round-bottomed flask equipped with a magnetic stir bar were successively added the iodoarene derivative (10.0 mmol, 1.0 equiv.), the corresponding phenylboronic acid (12.0 mmol, 1.2 equiv.), $PdCl_2(PPh_3)_2$ (0.2 mmol, 2.0 mol%), K_2CO_3 (25.0 mmol, 2.5 equiv.), 1,2-dimethoxyethane (30.0 mL), and water (4.0 mL). The mixture was degassed for 30 min and then stirred at 80 °C for 16 h. After completion, the reaction was quenched with saturated NH_4Cl aqueous solution, and the product was extracted with ethyl acetate and water, then dried over $MgSO_4$. The crude product was purified by flash chromatography using the indicated solvents as eluent to afford the corresponding compound 2-bromo-biphenyl.

General Procedure 2 (GP 2)

In a dried 25 mL flask, a solution of the corresponding 2-bromobiphenyl derivative (1.0 equiv) in THF was cooled to -78 °C. n-BuLi (2.5 M in hexanes, 1.5 equiv.) was added dropwise. After stirring for 0.5 h at -78 °C, chlorosilane (1.5 equiv.) was added, and the reaction mixture was allowed to warm to room temperature and stirred overnight. The reaction was quenched with water, and the aqueous phase was extracted with ethyl acetate. The combined organic layers were dried over $MgSO_4$, and the solvents were removed under reduced pressure. The crude product was purified by flash chromatography (using the indicated solvents as eluent) to afford the corresponding compound 5.

2-Bromo-1,1′:4′,1″-terphenyl. Prepared from 1-bromo-2-iodobenzene (1.41 g, 5.0 mmol, 1.0 equiv.), 4-biphenylboronic acid (1.19 g, 6.0 mmol, 1.2 equiv.), K_2CO_3 (1.73 g, 12.5 mmol, 2.5 equiv.), and $PdCl_2(PPh_3)_2$ (70.2 mg, 0.1 mmol, 2.0 mol%) according to **GP1**. Purification by flash column chromatography on silica gel using cyclohexane as eluent afforded 1.42 g of the desired compound (92%) as a white solid. **^{1}H NMR** (400 MHz, $CDCl_3$) δ 7.73-7.67 (m, 5H), 7.53-7.47 (m, 4H), 7.41-7.37 (m, 3H), 7.24-7.21 (m, 1H); **^{13}C NMR** (100 MHz, $CDCl_3$) δ 142.3, 140.8, 140.6, 140.2, 133.4, 131.5, 130.0, 128.94, 128.91, 127.6, 127.5, 127.3, 126.8, 122.8. This is a known compound, and the spectroscopic data is in agreement with the literature.[43]

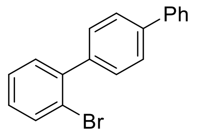

Chemical Formula: $C_{18}H_{13}Br$
Molecular Weight: 309.21

2-Bromo-4′-methyl-1,1′-biphenyl. Prepared from 1-bromo-2-iodobenzene (1.41 g, 5.0 mmol, 1.0 equiv.), *p*-tolylboronic acid (816 mg, 6.0 mmol, 1.2 equiv.), K_2CO_3 (1.73 g, 12.5 mmol, 2.5 equiv.), and $PdCl_2(PPh_3)_2$ (70.2 mg, 0.1 mmol, 2.0 mol%) according to **GP1**. Purification by flash column chromatography on silica gel using cyclohexane as eluent afforded 984 mg of the desired compound (80%) as a colorless oil. **^{1}H NMR** (400 MHz, $CDCl_3$) δ 7.70 (d, *J* = 8.0 Hz, 1H), 7.40-

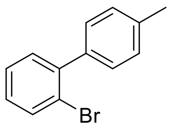

Chemical Formula: $C_{13}H_{11}Br$
Molecular Weight: 247.14

7.34 (m, 4H), 7.28 (d, *J* = 7.9 Hz, 2H), 7.21 (ddd, *J* = 8.4, 6.3, 2.7 Hz, 1H), 2.45 (s, 3H); **^{13}C NMR** (100 MHz, $CDCl_3$) δ 142.7, 138.4, 137.5, 133.2, 131.4, 129.4, 128.8, 128.6, 127.5, 122.9, 21.4. This is a known compound, and the spectroscopic data is in agreement with the literature.[44]

2-Bromo-4'-isopropyl-1,1'-biphenyl. Prepared from 1-iodo-4-isopropylbenzene (2.46 g, 10.0 mmol, 1.0 equiv.), 2-bromophenylboronic acid (2.41 g, 12.0 mmol, 1.2 equiv.), K_2CO_3 (3.46 g, 25.0 mmol, 2.5 equiv.), and $PdCl_2(PPh_3)_2$ (140.4 mg, 0.2 mmol, 2.0 mol%) according to **GP1**. Purification by flash column chromatography on silica gel using cyclohexane as eluent afforded 2.35 g of the desired compound (86%) as a colorless oil. **^{1}H NMR** (300 MHz, $CDCl_3$) δ 7.67 (dd, *J* = 7.8, 1.0 Hz, 1H), 7.43-7.29 (m, 6H), 7.23-7.15 (m, 1H), 2.99 (sept, *J* = 6.9 Hz, 1H), 1.32 (d, *J* = 7.0 Hz, 6H); **^{13}C NMR** (100 MHz, $CDCl_3$) δ 148.3, 142.7, 138.6, 133.3, 131.5, 129.4, 128.6, 127.5, 126.2, 122.9, 34.0, 24.1; **IR (neat, cm^{-1})**: 3054, 2959, 1911, 1613, 1589, 1466, 1435, 1403, 1363, 1246, 1160, 1120, 1067, 1052, 1024, 1003, 832, 756, 741, 650; **HRMS** (APCI): Calcd for $[C_{15}H_{14}Br]^+$: *m/z* 273.0273, Found 273.0274.

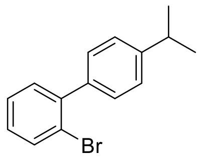

Chemical Formula: $C_{15}H_{15}Br$
Molecular Weight: 275.19

2-Bromo-4'-(*tert*-butyl)-1,1'-biphenyl. Prepared from 1-bromo-2-iodobenzene (1.41 g, 5.0 mmol, 1.0 equiv.), 4-(*tert*-butyl)phenyl boronic acid (1.07 g, 6.0 mmol, 1.2 equiv.), K_2CO_3 (1.73 g, 12.5 mmol, 2.5 equiv.), and $PdCl_2(PPh_3)_2$ (70.2 mg, 0.1 mmol, 2.0 mol%) according to **GP1**. Purification by flash column chromatography on silica gel using cyclohexane as eluent afforded 1.22 g of the desired compound (85%) as a colorless oil. **^{1}H NMR** (300 MHz, $CDCl_3$) δ 7.68-7.65 (m, 1H), 7.46-7.44 (m, 2H), 7.38-7.33 (m, 4H), 7.21-7.15 (m, 1H), 1.38 (s, 9H); **^{13}C NMR** (100 MHz, $CDCl_3$) δ 150.6, 142.7, 138.2, 133.3, 131.6, 129.2, 128.6, 127.5, 125.0, 122.8, 34.8, 31.5. This is a known compound, and the spectroscopic data is in agreement with the literature.[45]

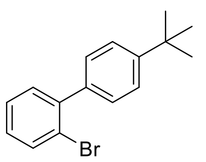

Chemical Formula: $C_{16}H_{17}Br$
Molecular Weight: 289.22

2-Bromo-4′-trifluoromethyl-1,1′-biphenyl. Prepared from 4-iodotriflouromethylbenzene (1.36 g, 5.0 mmol, 1.0 equiv.), 2-bromo phenyl boronic acid (1.20 g, 6.0 mmol, 1.2 equiv.), K_2CO_3 (1.73 g, 12.5 mmol, 2.5 equiv.), and $PdCl_2(PPh_3)_2$ (70.2 mg, 0.1 mmol, 2.0 mol%) according to **GP1**. Purification by flash column chromatography on silica gel using cyclohexane as eluent afforded 1.13 g of the desired compound (75%) as a colorless oil. **^{1}H NMR** (300 MHz, $CDCl_3$) δ 7.70 (d, *J* = 8.0 Hz, 3H), 7.54 (d, *J* = 8.1 Hz, 2H), 7.42-7.37 (m, 1H), 7.32 (dd, *J* = 7.6, 1.8 Hz, 1H), 7.25 (td, *J* = 7.5, 1.9 Hz, 1H); **^{13}C NMR** (100 MHz, $CDCl_3$) δ 144.7, 141.4, 133.5, 131.2, 130.0, 129.8 (q, J_F = 32,1 Hz), 129.6, 127.7, 125.2 (q, J_F = 3.8 Hz), 124.3 (q, J_F = 272.1 Hz), 122.4. This is a known compound, and

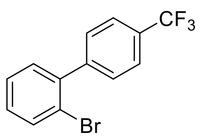

Chemical Formula: $C_{13}H_8BrF_3$
Molecular Weight: 301.11

the spectroscopic data is in agreement with the literature.[44]

2-Bromo-4′-fluoro -1,1′-biphenyl. Prepared from 1-bromo-2-iodobenzene (1.41 g, 5.0 mmol, 1.0 equiv.), 4-fluorobenzeneboronic acid (840 mg, 6.0 mmol, 1.2 equiv.), K_2CO_3 (1.73 g, 12.5 mmol, 2.5 equiv.), and $PdCl_2(PPh_3)_2$ (70.2 mg, 0.1 mmol, 2.0 mol%) according to **GP1**. Purification by flash column chromatography on silica gel using cyclohexane as eluent afforded 1.10 g of the desired compound (88%) as a colorless oil. **^{1}H NMR** (300 MHz, $CDCl_3$) δ 7.67 (d, *J* = 7.9 Hz, 1H), 7.41-7.29 (m, 4H), 7.24-7.18 (m, 1H), 7.15-7.09 (m, 2H); **^{13}C NMR** (100 MHz, $CDCl_3$) δ 162.5 (d, J_F = 246.9 Hz), 141.7, 137.2 (d, J_F = 3.3 Hz), 133.3, 131.4, 131.2 (d, J_F = 8.2 Hz), 129.0, 127.6, 122.9, 115.1 (d, J_F = 21.4 Hz). This is a known compound, and the spectroscopic data is in agreement with the literature.[46]

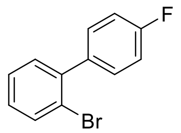

Chemical Formula: $C_{12}H_8BrF$
Molecular Weight: 251.10

2-Bromo-4'-(trifluoromethoxy)-1,1'-biphenyl. Prepared from 1-bromo-2-iodobenzene (2.82 g, 10.0 mmol, 1.0 equiv.), 4-trifluoromethoxyphenylboronic acid (2.47 g, 12.0 mmol, 1.2 equiv.), K_2CO_3 (3.46 g, 25.0 mmol, 2.5 equiv.), and $PdCl_2(PPh_3)_2$ (140.4 mg, 0.2 mmol, 2.0 mol%) according to **GP1**. Purification by flash column chromatography on silica gel using petroleum ether as eluent afforded 2.65 g of the desired compound (84%) as a colorless oil. **^{1}H NMR** (400 MHz, $CDCl_3$) δ 7.69 (dd, *J* = 8.0, 1.2 Hz, 1H), 7.46-7.43 (m, 2H), 7.38 (td, *J* = 7.4, 1.2 Hz, 1H), 7.33-7.27 (m, 3H), 7.23 (td, *J* = 7.7, 1.9 Hz, 1H); **^{13}C NMR** (100 MHz, $CDCl_3$) δ 148.9, 141.4, 139.8, 133.4, 131.3, 131.0, 129.3, 127.6, 122.7, 120.6 (q, J_F = 253.7 Hz), 120.5. This is a known compound, and the spectroscopic data is in agreement with the literature.[47]

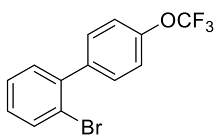

Chemical Formula: $C_{13}H_8BrF_3O$
Molecular Weight: 317.11

2-Bromo-2′-methyl-1,1′-biphenyl. Prepared from 1-bromo-2-iodobenzene (1.41 g, 5.0 mmol, 1.0 equiv.), *o*-tolylboronic acid (816 mg, 6.0 mmol, 1.2 equiv.), K_2CO_3 (1.73 g, 12.5 mmol, 2.5 equiv.), and $PdCl_2(PPh_3)_2$ (70.2 mg, 0.1 mmol, 2.0 mol%) according to **GP1**. Purification by flash column chromatography on silica gel using cyclohexane as eluent afforded 1.20 g of the desired compound (98%) as a colorless oil. **^{1}H NMR** (300 MHz, $CDCl_3$) δ 7.67 (d, *J* = 8.1 Hz, 1H), 7.39-7.28 (m, 3H), 7.25-7.19 (m, 3H), 7.13 (d, *J* = 6.0 Hz, 1H), 2.12 (s, 3H); **^{13}C NMR** (100 MHz, $CDCl_3$) δ 142.8, 141.3, 136.1, 132.7, 131.0, 129.9, 129.4, 128.8, 128.0, 127.3, 125.6, 123.9, 20.0. This is a known compound, and the spectroscopic data is in agreement with the literature.[44]

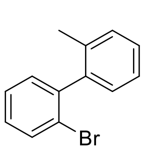

Chemical Formula: $C_{13}H_{11}Br$
Molecular Weight: 247.14

2-Bromo-2′-methoxyl-1,1′-biphenyl. Prepared from 1-bromo-2-iodobenzene (1.41 g, 5.0 mmol, 1.0 equiv.), 2-methoxyphenylboronic acid (912 mg, 6.0 mmol, 1.2 equiv.), K_2CO_3 (1.73 g, 12.5 mmol, 2.5 equiv.), and $PdCl_2(PPh_3)_2$ (70.2 mg, 0.1 mmol, 2.0 mol%) according to **GP1**. Purification by flash column chromatography on silica gel using cyclohexane as eluent afforded 1.15 g of the desired compound (88%) as a white solid. **^{1}H NMR** (400 MHz, $CDCl_3$) δ 7.67 (dd, *J* = 8.0, 1.2 Hz, 1H), 7.41 (dd, *J* = 7.3, 1.8 Hz, 1H), 7.39-7.35 (m, 1H), 7.31 (dd, *J* = 7.6, 1.9 Hz, 1H), 7.23 (dd, *J* = 7.9, 2.0 Hz, 1H), 7.19 (dd, *J* = 7.3, 1.8 Hz, 1H), 7.05 (td, *J* = 7.4, 1.1 Hz, 1H), 7.00 (dd, *J* = 8.3, 1.0 Hz, 1H), 3.80 (s, 3H); **^{13}C NMR** (100 MHz, $CDCl_3$) δ 156.7, 140.0, 132.6, 131.7, 131.0, 130.4, 129.5, 128.8, 127.2, 124.4, 120.4, 111.1, 55.7. This is a known compound, and the spectroscopic data is in agreement with the literature.[48]

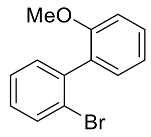

Chemical Formula: $C_{13}H_{11}BrO$
Molecular Weight: 263.13

2-Bromo-2'-ethyl-1,1'-biphenyl. Prepared from 1-iodo-2- ethylbenzene (2.32 g, 10.0 mmol, 1.0 equiv.), 2-bromophenylboronic acid (2.41 g, 12.0 mmol, 1.2 equiv.), K_2CO_3 (3.46 g, 25.0 mmol, 2.5 equiv.), and $PdCl_2(PPh_3)_2$ (140.4 mg, 0.2 mmol, 2.0 mol%) according to **GP1**. Purification by flash column chromatography on silica gel using cyclohexane as eluent afforded 2.25 g of the desired compound (87%) as a colorless oil. **^{1}H NMR** (300 MHz, $CDCl_3$) δ 7.68 (dd, *J* = 8.0, 1.3 Hz, 1H), 7.41-7.33 (m, 3H), 7.30-7.26 (m, 2H), 7.25-7.21 (m, 1H),7.13 (dd, *J* = 7.4, 1.3 Hz, 1H), 2.58-2.35 (m, 2H), 1.10 (t, *J* = 7.6 Hz, 3H); **^{13}C NMR** (100 MHz, $CDCl_3$) δ 142.6, 142.0, 140.7, 132.7, 131.3, 129.7, 128.8, 128.3, 128.3, 127.1, 125.5, 124.1, 26.3, 15.1; **IR (neat, cm^{-1})**: 3056, 2965, 1919, 1560, 1464, 1423, 1373, 1247, 1159, 1119, 1064, 1025, 1004, 868, 749, 729, 659; **HRMS** (APCI): Calcd for $[C_{14}H_{13}Br]^+$: *m/z* 260.0195, Found 260.0197.

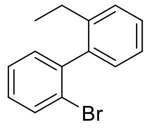

Chemical Formula: $C_{14}H_{13}Br$
Molecular Weight: 261.16

2-Bromo-3′-methyl-1,1′-biphenyl. Prepared from 1-bromo-2-iodobenzene (1.41 g, 5.0 mmol, 1.0 equiv.), *m*-tolylboronic acid (816 mg, 6.0 mmol, 1.2 equiv.), K_2CO_3 (1.73 g, 12.5 mmol, 2.5 equiv.), and $PdCl_2(PPh_3)_2$ (70.2 mg, 0.1 mmol, 2.0 mol%) according to **GP1**. Purification by flash column chromatography on silica gel using cyclohexane as eluent afforded 1.01 g of the desired compound (82%) as a colorless oil. **^{1}H NMR** (300 MHz, $CDCl_3$) δ 7.67 (dd, *J* = 7.7, 1.0 Hz, 1H), 7.39-7.31 (m, 3H), 7.24-7.19 (m, 4H), 2.43 (s, 3H); **^{13}C NMR** (100 MHz, $CDCl_3$) δ 142.9, 141.2, 137.7, 133.2, 131.4, 130.2, 128.7, 128.5, 128.0, 127.4, 126.6, 122.8, 21.6. This is a known compound, and the spectroscopic data is in agreement with the literature.[44]

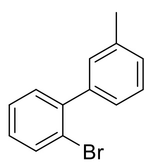

Chemical Formula: $C_{13}H_{11}Br$
Molecular Weight: 247.14

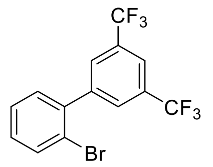

Chemical Formula: $C_{14}H_7BrF_6$
Molecular Weight: 369.10

2-Bromo-3′,5′-bis(trifluoromethyl)-1,1′-biphenyl. Prepared from 1-bromo-2-iodobenzene (2.82 g, 10.0 mmol, 1.0 equiv.), 3,5-bis(trifluoromethyl)benzeneboronic acid (3.10 g, 12.0 mmol, 1.2 equiv.), K_2CO_3 (3.46 g, 25.0 mmol, 2.5 equiv.), and $PdCl_2(PPh_3)_2$ (140.4 mg, 0.2 mmol, 2.0 mol%) according to **GP1**. Purification by flash column chromatography on silica gel using cyclohexane as eluent afforded 3.31 g of the desired compound (90%) as a yellow oil. **^{1}H NMR** (300 MHz, $CDCl_3$) δ 7.92 (s, 1H), 7.90 (s, 2H), 7.73 (dd, *J* = 7.9, 1.3 Hz, 1H), 7.42 (td, *J* = 7.3, 1.3 Hz, 1H), 7.35 (dd, *J* = 7.8, 2.0 Hz, 1H), 7.33-7.28 (m, 1H); **^{13}C NMR** (100 MHz, $CDCl_3$) δ 143.0, 139.7, 133.7, 131.6 (q, J_F = 32.1 Hz), 131.2, 130.3, 129.9 (q, J_F = 3.7 Hz), 128.0, 123.3 (q, J_F = 270.9 Hz), 122.4, 121.8-121.6 (m). This is a known compound, and the spectroscopic data is in agreement with the literature.[49]

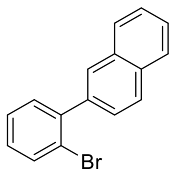

Chemical Formula: $C_{16}H_{11}Br$
Molecular Weight: 283.17

2-(2′-Bromophenyl) naphthalene. Prepared from 1-bromo-2-iodobenzene (1.41 g, 5.0 mmol, 1.0 equiv.), 2-naphthaleneboronic acid (1.03 g, 6.0 mmol, 1.2 equiv.), K_2CO_3 (1.73 g, 12.5 mmol, 2.5 equiv.), and $PdCl_2(PPh_3)_2$ (70.2 mg, 0.1 mmol, 2.0 mol%) according to **GP1**. Purification by flash column chromatography on silica gel using cyclohexane as eluent afforded 1.20 g of the desired compound (85%) as a white soild. **^{1}H NMR** (400 MHz, $CDCl_3$) δ 7.93-7.88 (m, 4H), 7.73 (dd, *J* = 8.0, 1.2 Hz, 2H), 7.59 (dd, *J* = 8.5, 1.8 Hz, 1H), 7.54 (dt, *J* = 6.2, 3.4 Hz, 1H), 7.46-7.39 (m, 2H), 7.28-7.23 (m, 1H); **^{13}C NMR** (100 MHz, $CDCl_3$) δ 142.7, 138.8, 133.3, 133.2, 132.8, 131.7, 129.0, 128.4, 128.3, 127.9, 127.8, 127.6, 127.5, 126.39, 126.38, 123.0. This is a known compound, and the spectroscopic data is in agreement with the literature.[50]

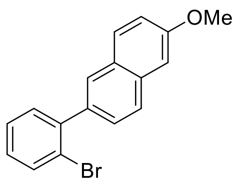

Chemical Formula: $C_{17}H_{13}BrO$
Molecular Weight: 313.19

2-(2-Bromophenyl)-6-methoxynaphthalene. Prepared from 1-bromo-2-iodobenzene (2.82 g, 10.0 mmol, 1.0 equiv.), 6-methoxy-2-naphthaleneboronic acid (2.42 g, 12.0 mmol, 1.2 equiv.), K_2CO_3 (3.46 g, 25.0 mmol, 2.5 equiv.), and $PdCl_2(PPh_3)_2$ (140.4 mg, 0.2 mmol, 2.0 mol%) according to **GP1**. Purification by flash column chromatography on silica gel using dichloromethane/petroleum ether (10:90–50:50) as eluent afforded 2.70 g of the desired compound (87%) as a white soild. **^{1}H NMR** (400 MHz, $CDCl_3$) δ 7.82-7.79 (m, 3H), 7.73 (dd, *J* = 8.0, 1.2 Hz, 1H), 7.57 (dd, *J* = 8.5, 1.7 Hz, 1H), 7.45 (dd, *J* = 7.6, 2.0 Hz, 1H), 7.40 (td, *J* = 7.4, 1.2 Hz, 1H), 7.24-7.21 (m, 3H), 3.96 (s, 3H); **^{13}C NMR** (100 MHz, $CDCl_3$) δ 158.1, 142.7, 136.6, 133.9, 133.3, 131.7, 129.8, 128.8, 128.7, 128.2, 128.2, 127.5, 126.3, 123.0, 119.2, 105.8, 55.5. This is a known compound, and the spectroscopic data is in agreement with the literature. [51]

(2-Bromophenyl)ferrocene. A mixture containing ferrocene (10 mmol) and t-BuOK (13 mol %, 146 mg) in THF (30 mL) is combined with t-BuLi (12 mmol, 1.7 M, 7.1 mL) under an inert atmosphere at low temperature, giving rise to a lithiated ferrocene species. Introduction of a $ZnCl_2$ solution in THF (20 mmol, 0.5 M, 40 mL) then affords the corresponding organozinc intermediate upon gradual warming to room temperature. The system is subsequently treated with $Pd(PPh_3)_4$ (5 mol %, 0.5 mmol, 578 mg) and 2-bromoiodobenzene (12 mmol), enabling the palladium-catalyzed coupling. After completion, the mixture undergoes an acidic aqueous workup with 2 N HCl (25 mL), followed by organic extraction, washing with water (20 mL) and brine (20 mL), and drying over $MgSO_4$. Concentration and purification by silica-gel chromatography using a dichloromethane/petroleum ether gradient (0:100 to 10:90) yield the desired compound as an orange oil (2.04 g, 60%). **^{1}H NMR** (400 MHz, $CDCl_3$) δ 7.82-7.79 (m, 3H), 7.73 (dd, *J* = 8.0, 1.2 Hz, 1H), 7.57 (dd, *J* = 8.5, 1.7 Hz, 1H), 7.45 (dd, *J* = 7.6, 2.0 Hz, 1H), 7.40 (td, *J* = 7.4, 1.2 Hz, 1H), 7.24-7.21 (m, 3H), 3.96 (s, 3H); **^{13}C NMR** (100 MHz, $CDCl_3$) δ 158.1, 142.7, 136.6, 133.9, 133.3, 131.7, 129.8, 128.8, 128.7, 128.2, 127.5, 126.3, 123.0, 119.2, 105.8, 55.5. This is a known compound, and the spectroscopic data is in agreement with the literature.[18]

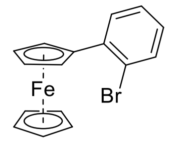

Chemical Formula: $C_{16}H_{13}BrFe$
Molecular Weight: 341.03

2,2'''-Dibromo-1,1':4',1'':4'',1'''-quaterphenyl. Prepared from 4,4'-diiodo-1,1'-biphenyl (2.03 g, 5.0 mmol, 1.0 equiv.), 2-bromophenylboronic acid (2.41 g, 12.0 mmol, 2.4 equiv.), K_2CO_3 (1.73 g, 12.5 mmol, 2.5 equiv.), and $PdCl_2(PPh_3)_2$ (140.4 mg, 0.2 mmol, 4.0 mol%) according to **GP1**. Purification by flash column chromatography on silica gel and then crystallization from ethanol/acetone afforded 1.51 gof the desired compound (65%) as a white soild. **^{1}H NMR** (400 MHz, $CDCl_3$) δ 7.75-7.70 (m, 6H), 7.54-7.52 (m, 4H), 7.41-7.36 (m, 4H), 7.25-7.21 (m, 2H); **^{13}C NMR** (100 MHz, $CDCl_3$) δ 142.3, 140.3, 140.0, 133.4, 131.5, 130.0, 128.9, 127.6, 126.8, 122.8. This is a known compound, and the spectroscopic data is in agreement with the literature.[52]

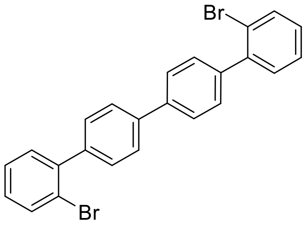

Chemical Formula: $C_{24}H_{16}Br_2$
Molecular Weight: 464.20

2,2''-Dibromo-1,1':4',1''-terphenyl. Prepared from 1,4-diiodobenzene (1.65 g, 5.0 mmol, 1.0 equiv.), 2-bromophenylboronic acid (2.41 g, 12.0 mmol, 2.4 equiv.), K_2CO_3 (1.73 g, 12.5 mmol, 2.5 equiv.), and $PdCl_2(PPh_3)_2$ (140.4 mg, 0.2 mmol, 4.0 mol%) according to **GP1**. Purification by flash column chromatography on silica gel using cyclohexane as eluent afforded 1.35 g of the desired compound (70%) as a white solid. **^{1}H NMR** (300 MHz, $CDCl_3$) δ 7.71-7.69 (m, 2H), 7.49 (s, 4H), 7.41-7.35 (m, 4H), 7.25-7.19 (m, 2H); **^{13}C NMR** (100 MHz, $CDCl_3$) δ 142.3,

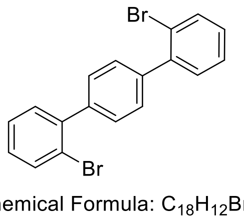

Chemical Formula: $C_{18}H_{12}Br_2$
Molecular Weight: 388.10

140.4, 133.4, 131.6, 129.2, 128.9, 127.6, 122.7. This is a known compound, and the spectroscopic data is in agreement with the literature.[44]

2-(Dimethylsilyl) biphenyl (5a). Following **GP2** from 2-iodobiphenyl (1.16 g, 5 mmol, 1.0 equiv.). Purification by silica gel (cyclohexane) afforded **5a** as a colorless oil (848 mg, 80%). **^{1}H NMR** (400 MHz, $CDCl_3$) δ 7.68 (dd, *J* = 1.8, 1.2 Hz, 1H), 7.46-7.32 (m, 8H), 4.38 (sept, *J* = 3.9 Hz, 1H), 0.10 (d, *J* = 3.8 Hz, 6H); **^{13}C NMR** (100 MHz, $CDCl_3$) δ 149.5, 143.9, 136.1, 135.3, 129.38, 129.36, 129.2, 128.0, 127.3, 126.5, -2.8. This is a known compound, and the spectroscopic data is in agreement with the literature.[15]

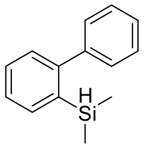

Chemical Formula: $C_{14}H_{16}Si$
Molecular Weight: 212.37

[1,1':4',1''-Terphenyl]-2-yldimethylsilane (5b). Following **GP2** from 2-bromo-1,1′:4′,1′′-terphenyl (308 mg, 1 mmol, 1.0 equiv.). Purification by silica gel (cyclohexane) afforded **5b** as a white solid (219 mg, 76%). **^{1}H NMR** (400 MHz, $CDCl_3$) δ 7.70-7.65 (m, 5H), 7.50-7.43 (m, 5H), 7.41-7.34 (m, 3H), 4.41 (sept, *J* = 3.8 Hz, 1H), 0.13 (d, *J* = 3.8 Hz, 6H); **^{13}C NMR** (100 MHz, $CDCl_3$) δ 149.0, 142.9, 140.9, 140.1, 136.2, 135.3, 129.8, 129.4, 129.3, 128.9, 127.5, 127.2, 126.7, 126.6, -2.8. This is a known compound, and the spectroscopic data is in agreement with the literature.[43]

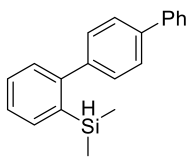

Chemical Formula: $C_{20}H_{20}Si$
Molecular Weight: 288.47

2-(Dimethylsilyl)-4′-methylbiphenyl (5c). Following **GP2** from 2-bromo-4′-methyl-1,1′-biphenyl (492 mg, 2 mmol, 1.0 equiv.). Purification by silica gel (cyclohexane) afforded **5c** as a white solid (271 mg, 60%). **^{1}H NMR** (400 MHz, $CDCl_3$) δ 7.64 (dd, *J* = 7.3, 1.5 Hz, 1H), 7.43 (td, *J* = 7.5, 1.6 Hz, 1H), 7.36 (td, *J* = 7.5, 1.4 Hz, 1H), 7.31-7.27 (m, 1H), 7.25-7.20 (m, 4H), 4.37 (sept, *J* = 3.8 Hz, 1H), 2.43 (s, 3H), 0.10 (d, *J* = 3.8 Hz, 6H); **^{13}C NMR** (100 MHz, $CDCl_3$) δ 149.5, 141.0, 136.9, 136.2, 135.2, 129.8, 129.5, 129.2, 128.7, 126.4, 21.4, -2.8. This is a known compound, and the spectroscopic data is in agreement with the literature.[43]

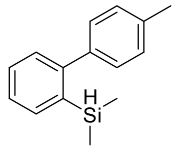

Chemical Formula: $C_{15}H_{18}Si$
Molecular Weight: 226.39

2-(Dimethylsilyl)-4′-isopropylbiphenyl (5d). Following **GP2** from 2-bromo-4'-isopropyl-1,1'-biphenyl (274 mg, 1 mmol, 1.0 equiv.). Purification by silica gel (cyclohexane) afforded **5d** as a colorless oil (183 mg, 72%). **^{1}H NMR** (300 MHz, $CDCl_3$) δ 7.65 (dd, *J* = 7.2, 1.5 Hz, 1H), 7.46-7.28 (m, 7H), 4.38 (sept, *J* = 3.7 Hz, 1H), 2.99 (sept, *J* = 6.9 Hz, 1H), 1.33 (d, *J* = 7.2 Hz, 6H), 0.08 (d, *J* = 3.8 Hz, 6H); **^{13}C NMR** (100 MHz, $CDCl_3$) δ 149.5, 148.0, 141.3, 136.2, 135.3, 129.4, 129.24, 129.23, 126.3, 126.0, 34.0, 24.2, -2.9; **IR (neat, cm^{-1})**: 3051, 2959, 2116, 1586, 1464, 1249, 1124, 1085, 1055, 1004,

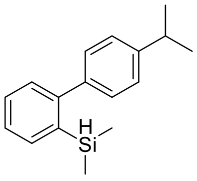

Chemical Formula: $C_{17}H_{22}Si$
Molecular Weight: 254.45

881, 833, 766, 742, 708; **HRMS** (APCI): Calcd for $[C_{17}H_{21}Si]^+$: *m/z* 253.1407, Found 253.1407.

2-(Dimethylsilyl)-4'-*tert*-butylbiphenyl (5e). Following **GP2** from 2-bromo-4'-(*tert*-butyl)-1,1'-biphenyl (1.44 g, 5 mmol, 1.0 equiv.). Purification by silica gel (cyclohexane) afforded **5e** as a colorless oil (912 mg, 68%). **^{1}H NMR** (300 MHz, $CDCl_3$) δ 7.65-7.59 (m, 1H), 7.44-7.27 (m, 7H), 4.37 (sept, *J* = 3.7 Hz, 1H), 1.38 (s, 9H), 0.06 (d, *J* = 3.8 Hz, 6H); **^{13}C NMR** (100 MHz, $CDCl_3$) δ 150.3, 149.4, 136.2, 135.3, 129.4, 129.2, 129.1, 129.0, 126.3, 124.9, 34.7, 31.6, -2.9. This is a known compound, and the spectroscopic data is in agreement with the literature.[15]

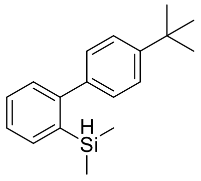

Chemical Formula: $C_{18}H_{24}Si$
Molecular Weight: 268.48

2-(Dimethylsilyl)-4'-trifluoromethylbiphenyl (5f). Following **GP2** from 2-bromo-4'-trifluoromethyl-1,1'-biphenyl (1.5 g, 5 mmol, 1.0 equiv.). Purification by silica gel (cyclohexane) afforded **5f** as a colorless oil (910 mg, 65%). **^{1}H NMR** (300 MHz, $CDCl_3$) δ 7.70-7.63 (m, 3H), 7.50-7.40 (m, 4H), 7.28 (d, *J* = 1.7, 1H), 4.32 (sept, *J* = 3.8 Hz, 1H), 0.10 (d, *J* = 3.8 Hz, 6H); **^{13}C NMR** (100 MHz, $CDCl_3$) δ 147.9, 147.5, 136.1, 135.4, 129.7, 129.4 (q, *J* = 27.8 Hz), 129.3, 127.4, , 127.2, 125.0 (q, *J* = 3.8 Hz), 124.5 (q, *J* = 275.0 Hz), -2.9; **^{19}F NMR** (376 MHz, $CDCl_3$) δ -63.5. This is a known compound, and the spectroscopic data is in agreement with the literature.[15]

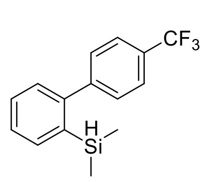

Chemical Formula: $C_{15}H_{15}F_3Si$
Molecular Weight: 280.37

2-(Dimethylsilyl)-4'-fluorobiphenyl (5g) Following **GP2** from 2-bromo-4'-fluoro -1,1'-biphenyl (250 mg, 1 mmol, 1.0 equiv.). Purification by silica gel (cyclohexane) afforded **5g** as a colorless oil (161 mg, 70%). **^{1}H NMR** (400 MHz, $CDCl_3$) δ 7.64 (dd, *J* = 7.2, 1.0 Hz, 1H), 7.43 (td, *J* = 7.4, 1.6 Hz, 1H), 7.37 (td, *J* = 7.4, 1.5 Hz, 1H), 7.34-7.26 (m, 3H), 7.09 (t, *J* =8.7 Hz, 2H), 4.34 (sept, *J* = 3.8 Hz, 1H), 0.10 (d, *J* = 3.7 Hz, 6H); **^{13}C NMR** (100 MHz, $CDCl_3$) δ 162.4 (d, J_F = 245.9 Hz), 148.3, 139.8 (d, J_F = 3.3 Hz), 136.2, 135.3, 130.9 (d, J_F = 8.1 Hz), 129.5, 129.3, 126.7, 114.9 (d, J_F = 21.3 Hz), -2.9; **^{19}F NMR** (376 MHz, $CDCl_3$) δ -116.6. This is a known compound, and the spectroscopic data is in agreement with the literature.[15]

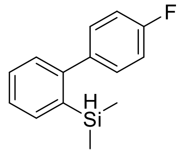

Chemical Formula: $C_{14}H_{15}FSi$
Molecular Weight: 230.36

Dimethyl(4'-(trifluoromethoxy)-[1,1'-biphenyl]-2-yl)silane (5h). Following **GP2** from 2-bromo-4'-(trifluoromethoxy)-1,1'-biphenyl (316 mg, 1 mmol, 1.0 equiv.). Purification by silica gel (cyclohexane) afforded **5h** as a colorless oil (243 mg, 82%). **^{1}H NMR** (400 MHz, $CDCl_3$) δ 7.64 (dd, *J* = 7.2, 1.6 Hz, 1H), 7.44 (td, *J* = 7.5, 1.6 Hz, 1H), 7.40-7.36 (m, 3H), 7.29-7.24 (m, 3H), 4.34 (sept, *J* = 3.8 Hz, 1H), 0.09 (d, *J* = 3.8 Hz, 6H); **^{13}C NMR** (100 MHz, $CDCl_3$) δ 148.7,

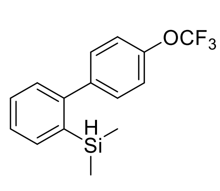

Chemical Formula: $C_{15}H_{15}F_3OSi$
Molecular Weight: 296.36

147.9, 142.5, 136.2, 135.3, 130.7, 129.4, 127.0, 120.5, 119.9 (q, J_F = 256.0 Hz), -2.9; **^{19}F NMR** (376 MHz, $CDCl_3$) δ -58.8; **IR (neat, cm^{-1})**: 3054, 2961, 2123, 1588, 1466, 1430, 1250, 1203, 1158, 1125, 1086, 1006, 881, 836, 767, 732, 709; **HRMS** (APCI): Calcd for $[C_{15}H_{14}F_3OSi]^+$: *m/z* 295.0761, Found 295.0760.

2-(Dimethylsilyl)-2′-methylbiphenyl (5i). Following **GP2** from 2-bromo-2′-methyl-1,1′-biphenyl (492 mg, 2 mmol, 1.0 equiv.). Purification by silica gel (cyclohexane) afforded **5i** as a colorless oil (338 mg, 75%). **^{1}H NMR** (300 MHz, $CDCl_3$) δ 7.65 (dd, *J* = 7.3, 1.6 Hz, 1H), 7.42 (td, *J* = 7.4, 1.7 Hz, 1H), 7.38-7.31 (m, 1H), 7.29-7.11 (m, 5H), 4.13 (sept, *J* = 4.0 Hz, 1H), 2.08 (s, 3H), 0.06 (d, *J* = 3.8 Hz, 3H), 0.01 (d, *J* = 3.8 Hz, 3H); **^{13}C NMR** (100 MHz, $CDCl_3$) δ 148.6, 142.9, 136.3, 136.2, 135.1, 130.1, 129.8, 129.2, 129.1, 127.6, 126.4, 125.1, 20.5, -3.3. This is a known compound, and the spectroscopic data is in agreement with the literature.[43]

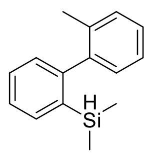

Chemical Formula: $C_{15}H_{18}Si$
Molecular Weight: 226.39

2-(Dimethylsilyl)-2′-methoxylbiphenyl (5j). Following **GP2** from 2-bromo-2′-methoxyl-1,1′-biphenyl (262 mg, 1 mmol, 1.0 equiv.). Purification by silica gel (cyclohexane) afforded **5j** as a colorless oil (171 mg, 73%). **^{1}H NMR** (400 MHz, $CDCl_3$) δ 7.63 (d, *J* = 7.5 Hz, 1H), 7.42 (t, *J* = 7.2 Hz, 1H), 7.36 (t, *J* = 7.4 Hz, 2H), 7.25 (d, *J* = 7.2 Hz, 1H), 7.17 (d, *J* = 7.3 Hz, 1H), 7.00 (d, *J* = 7.4 Hz, 1H), 6.94 (d, *J* = 8.3 Hz, 1H), 4.15 (sept, *J* = 3.9 Hz, 1H), 3.74 (s, 3H), 0.07 (d, *J* = 3.8 Hz, 6H); **^{13}C NMR** (100 MHz, $CDCl_3$) δ 156.9, 145.3, 137.3, 134.8, 132.3, 131.5, 130.0, 128.98, 128.95, 126.6, 120.1, 110.5, 55.4, -3.1. This is a known compound, and the spectroscopic data is in agreement with the literature.[15]

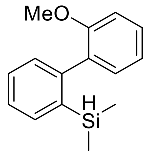

Chemical Formula: $C_{15}H_{18}OSi$
Molecular Weight: 242.39

2-(Dimethylsilyl)-2′-methoxylbiphenyl (5k). Following **GP2** from 2-bromo-2'-ethyl-1,1'-biphenyl (260 mg, 1 mmol, 1.0 equiv.). Purification by silica gel (cyclohexane) afforded **5k** as a colorless oil (168 mg, 70%). **^{1}H NMR** (300 MHz, $CDCl_3$) δ 7.64 (dd, *J* = 7.3, 1.6 Hz, 1H), 7.45-7.30 (m, 4H), 7.24-7.19 (m, 2H), 7.14 (dd, *J* = 7.5, 1.7 Hz, 1H), 4.11 (sept, *J* = 3.8 Hz, 1H), 2.42 (qd, *J* = 7.4, 4.4 Hz, 2H), 1.09 (t, *J* = 7.6 Hz, 3H), 0.07 (d, *J* = 3.8 Hz, 3H), 0.01 (d, *J* = 3.8 Hz, 3H); **^{13}C NMR** (100 MHz, $CDCl_3$) δ 148.4, 142.4, 142.1, 136.5, 135.0, 130.3, 129.4, 128.9, 128.0, 127.8, 126.4, 125.0, 26.5, 15.2, -3.0, -3.1; **IR (neat, cm^{-1})**: 3055, 2965, 2116, 1585, 1462, 1426, 1248, 1125, 1085, 1044, 1005, 880, 836, 774, 753, 709; **HRMS** (APCI): Calcd for $[C_{16}H_{19}Si]^+$: *m/z* 239.1251, Found 239.1251.

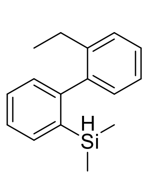

Chemical Formula: $C_{16}H_{20}Si$
Molecular Weight: 240.42

2-(Dimethylsilyl)-3′-methylbiphenyl (5l). Following **GP2** from 2-bromo-3′-methyl-1,1′-biphenyl (492 mg, 2 mmol, 1.0 equiv.). Purification by silica gel (cyclohexane) afforded **5l** as a colorless oil (326 mg, 72%). **^{1}H NMR** (300 MHz, $CDCl_3$) δ 7.65-7.62 (m, 1H), 7.42 (td, *J* = 7.4, 1.6 Hz, 1H), 7.36 (dd, *J* = 7.3, 1.5 Hz, 1H), 7.33-7.28 (m, 2H), 7.19-7.14 (m, 3H), 4.33 (sept, *J* = 3.7 Hz, 1H), 2.40 (s, 3H), 0.08 (d, *J* = 3.8 Hz, 6H); **^{13}C NMR** (100 MHz, $CDCl_3$) δ 149.6, 143.8, 137.6, 136.1, 135.3, 130.2, 129.3, 129.2, 127.9, 127.8, 126.44, 126.38, 21.6, -2.8. This is a known compound, and the spectroscopic data is in agreement with the literature.[15]

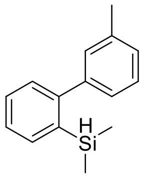

Chemical Formula: $C_{15}H_{18}Si$
Molecular Weight: 226.39

2-(Dimethylsilyl)-3',5'-bis(trifluoromethyl)biphenyl (5m). Following **GP2** from 2-bromo-3′,5′-bis(trifluoromethyl)-1,1′-biphenyl (368 mg, 1 mmol, 1.0 equiv.). Purification by silica gel (cyclohexane) afforded **5m** as a colorless oil (285 mg, 82%). **^{1}H NMR** (400 MHz, $CDCl_3$) δ 7.90 (s, 1H), 7.83 (s, 2H), 7.68 (dd, *J* = 7.3, 1.4 Hz, 1H), 7.51-7.42 (m, 2H), 7.30 (dd, *J* = 7.4, 1.5 Hz, 1H), 4.26 (sept, *J* = 3.7 Hz, 1H), 0.13 (d, *J* = 3.8 Hz, 6H); **^{13}C NMR** (100 MHz, $CDCl_3$) δ 146.0, 145.7, 136.4, 135.6, 131.9 (q, J_F = 131.3), 129.7, 129.6 (q, J_F = 3.8 Hz), 129.3, 127.9, 123.4 (q, J_F = 272.6 Hz), 121.1-121.0 (m), -3.0; **^{19}F NMR** (376 MHz, $CDCl_3$) δ -63.9; **IR (neat, cm^{-1})**: 3059, 2962, 2132, 1619, 1378, 1276, 1172, 1127, 1090, 1052, 1033, 883, 835, 765, 709, 681; **HRMS** (ESI): Calcd for $[C_{16}H_{13}F_6Si_2]^+$: *m/z* 347.0685, Found 347.0682

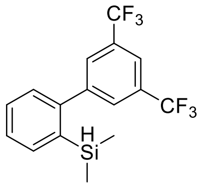

Chemical Formula: $C_{16}H_{14}F_6Si$
Molecular Weight: 348.36

2-(2-Dimethylsilylphenyl)naphthalene (5n). Following **GP2** from 2-(2′-bromophenyl) naphthalene (282 mg, 1 mmol, 1.0 equiv.). Purification by silica gel (cyclohexane) afforded **5n** as a colorless oil (157 mg, 60%). **^{1}H NMR** (400 MHz, $CDCl_3$) δ 7.92-7.84 (m, 4H), 7.71-7.69 (m, 1H), 7.56-7.52 (m, 3H), 7.50-7.46 (m, 1H), 7.43-7.40 (m, 2H), 4.39 (sept, *J* = 3.7 Hz, 1H), 0.09 (d, *J* = 3.8 Hz, 6H); **^{13}C NMR** (100 MHz, $CDCl_3$) δ 149.3, 141.3, 136.4, 135.4, 133.1, 132.6, 129.6, 129.3, 128.2, 128.1, 127.9, 127.6, 126.6, 126.4, 126.1, -2.8. This is a known compound, and the spectroscopic data is in agreement with the literature.[15]

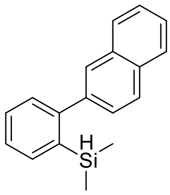

Chemical Formula: $C_{18}H_{18}Si$
Molecular Weight: 262.43

(2-(6-Methoxynaphthalen-2-yl)phenyl)dimethylsilane (5o). Following **GP2** from 2-(2-bromophenyl)-6-methoxynaphthalene (312 mg, 1 mmol, 1.0 equiv.). Purification by silica gel (dichloromethane/petroleum ether (10:90–20:80)) afforded **5o** as a colorless oil (254 mg, 87%). **^{1}H NMR** (400 MHz, $CDCl_3$) δ 7.78-7.72 (m, 3H), 7.72-7.70 (m, 1H), 7.52-7.47 (m, 2H), 7.43-7.40 (m, 2H), 7.24-7.22 (m, 2H), 4.42 (sept, *J* = 3.5 Hz, 1H), 3.97 (s, 3H), 0.10 (d, *J* = 3.8 Hz, 6H); **^{13}C NMR** (100 MHz, $CDCl_3$) δ 157.9, 149.4, 139.2, 136.4, 135.3, 133.7, 129.69, 129.66, 129.3, 128.6, 128.4, 127.9, 126.5, 126.4, 119.3, 105.8, 55.5, -2.8; **IR (neat, cm^{-1})**: 3055, 3007, 2964, 2122, 1607, 1390, 1247, 1203, 1122, 1085, 1027, 880, 854, 836, 812, 754, 710; **HRMS** (APCI): Calcd for $[C_{19}H_{21}OSi]^+$: *m/z* 293.1356, Found 293.1354.

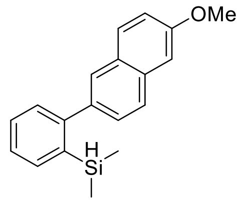

Chemical Formula: $C_{19}H_{20}OSi$
Molecular Weight: 292.45

2-(Dimethylsilyl)phenyl]ferrocene (5p). Following **GP2** from (2-bromophenyl)ferrocene (340 mg, 1 mmol, 1.0 equiv.). Purification by silica gel (petroleum ether) afforded **5q** as an orange solid (278 mg, 87%). **^{1}H NMR** (400 MHz, $CDCl_3$) δ 7.91 (d, *J* = 7.2 Hz, 1H), 7.47 (dd, *J* = 7.4, 1.5 Hz, 1H), 7.40 (td, *J* = 7.5, 1.5 Hz, 1H), 7.27-7.23 (m, 1H), 4.49 (t, *J* = 1.6 Hz, 2H), 4.48-4.46 (m, 1H), 4.27 (t, *J* = 1.9 Hz, 2H), 4.18 (s, 5H), 0.11 (d, *J* = 3.7 Hz, 6H); **^{13}C NMR** (100 MHz, $CDCl_3$) δ 145.2, 136.8, 134.8, 131.2, 129.0, 125.9, 91.6, 70.7, 69.6, 67.9, -2.8. This is a known compound, and the spectroscopic data is in agreement with the literature.[18]

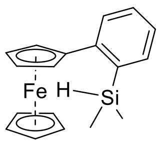

Chemical Formula: $C_{18}H_{20}FeSi$
Molecular Weight: 320.29

Dimethyl(1-phenylnaphthalen-2-yl)silane (5q). Following **GP2** from 2-bromo-1-phenylnaphthalene (282 mg, 1 mmol, 1.0 equiv.). Purification by silica gel (petroleum ether) afforded **5p** as a white solid (236 mg, 90%). **^{1}H NMR** (400 MHz, $CDCl_3$) δ 7.92-7.86 (m, 2H), 7.76-7.70 (m, 1H), 7.55-7.45 (m, 5H), 7.42-7.33 (m, 3H), 4.28-4.19 (m, 1H), 0.14-0.09 (m, 6H); **^{13}C NMR** (100 MHz, $CDCl_3$) δ 147.6, 141.1, 134.0, 132.5, 130.8, 128.1, 127.6, 126.8, 126.7, 126.4, 126.0, -2.7; **IR (neat, cm^{-1})**: 3051, 2955, 2117, 1552, 1490, 1440, 1378, 1365, 1249, 1101, 1072, 1027, 882, 855, 813, 761, 701, 671; **HRMS** (APCI): Calcd for $[C_{18}H_{17}Si]^+$: *m/z* 261.1094, Found 261.1093.

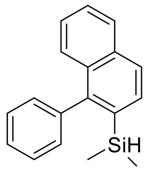

Chemical Formula: $C_{18}H_{18}Si$
Molecular Weight: 262.43

2,2'''-Bis(dimethylsilyl)-1,1':4',1'':4'',1'''-quaterphenyl (5r). Following **GP2** from (2,2'''-dibromo-1,1':4',1'':4'',1'''-quaterphenyl (462 mg, 1 mmol, 1.0 equiv.). Purification by silica gel (ethanol/acetone) afforded **5r** as a white solid (249 mg, 59%). **^{1}H NMR** (400 MHz, $CDCl_3$) δ 7.74-7.71 (m, 4H), 7.67 (dd, *J* = 7.2, 1.2 Hz, 2H), 7.48-7.44 (m, 6H), 7.38 (qd, *J* = 7.4, 1.4 Hz, 4H), 4.42 (sept, *J* = 3.7 Hz, 2H), 0.14 (d, *J* = 3.7 Hz, 12H); **^{13}C NMR** (100 MHz, $CDCl_3$) δ 149.0, 143.0, 139.6, 136.2, 135.3, 129.8, 129.4, 129.3, 126.6, -2.7; **IR (neat, cm^{-1})**: 3054, 2951, 2900, 2144, 1584, 1562, 1462, 1427, 1246, 1124, 1083, 1045, 1004, 874, 830, 754, 710; **HRMS** (ESI): Calcd for $[C_{28}H_{29}Si_2]^+$: *m/z* 421.1802, Found 421.1805.

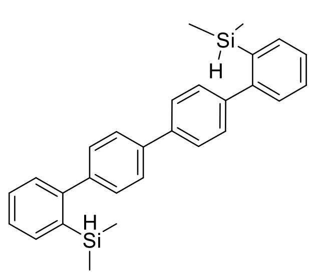

Chemical Formula: $C_{28}H_{30}Si_2$
Molecular Weight: 422.72

2,2''-Bis(dimethylsilyl)-*p*-terphenyl (5s). Following **GP2** from 2,2″-dibromo-1,1′:4′,1″-terphenyl (386 mg, 1 mmol, 1.0 equiv.). Purification by silica gel (cyclohexane) afforded **5s** as a white solid (97 mg, 28%). **^{1}H NMR** (300 MHz, $CDCl_3$) δ 7.67-7.64 (m, 2H), 7.47-7.33 (m, 10H), 4.41 (sept, *J* = 3.8 Hz, 2H), 0.15 (d, *J* = 3.9 Hz, 12H); **^{13}C NMR** (100 MHz, $CDCl_3$) δ 149.2, 142.7, 136.2, 135.3, 129.6, 129.3, 128.9, 126.6, -2.7. This is a known compound, and the spectroscopic data is in agreement with the literature.[15]

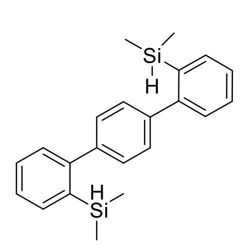

Chemical Formula: $C_{22}H_{26}Si_2$
Molecular Weight: 346.62

Bis(biphenyl-2-yl)silane (5t). A solution of the 2-bromobiphenyl (2.33 g, 10.0 mmol, 1.0 equiv.) in Et_2O (30 mL) was cooled to -78 °C. *t*-BuLi (1.7 M in pentane, 2.2 equiv.) was added dropwise under an argon atmosphere, and the mixture was slowly warmed to room temperature and stirred for 1 h. The resulting solution was then added dropwise to a solution of tetrachlorosilane (0.570 mL, 5.0 mmol, 0.5 equiv.) in Et_2O (5.0 mL) at -78 °C, and the mixture was stirred at room temperature for an additional 6 h. The reaction mixture was added to a suspension of $LiAlH_4$ (1.52 g, 40.0 mmol, 4.0 eq) in Et_2O (20 mL) at 0 °C, and the mixture was stirred at 40 °C for 16 h. The reaction mixture was quenched with H_2O and the aqueous phase was extracted with dichloromethane. The combined organic layers were dried over $MgSO_4$, and the solvents were evaporated under reduced pressure. Purification of the crude product by silica gel column chromatography (petroleum ether) afforded **5t** (840 mg, 50%) as a colorless oil. **^{1}H NMR** (400 MHz, $CDCl_3$) d 7.40 (td , *J* = 7.5, 1.5 Hz, 2H), 7.34 (dd , *J* = 7.4, 1.4 Hz, 2H), 7.28-7.26 (m, 5H), 7.25-7.19 (m, 5H), 7.17-7.15 (m, 4H), 4.59 (s, 2H); **^{13}C NMR** (100 MHz, $CDCl_3$) d 149.7, 143.3, 137.5, 131.4, 129.8, 129.1, 129.1, 128.0, 127.2, 126.4. This is a known compound, and the spectroscopic data is in agreement with the literature.[19]

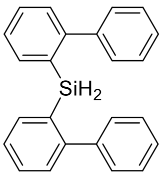

Chemical Formula: $C_{24}H_{20}Si$
Molecular Weight: 336.51

1,1′-Biphenyl-2-yl-diphenylsilane (5u). Following **GP2** from 2-iodobiphenyl (232 mg, 1 mmol, 1.0 equiv.). Purification by silica gel (cyclohexane/ethyl acetate = 4/1) afforded **5u** as a white solid (215 mg, 64%).**^{1}H NMR** (300 MHz, $CDCl_3$) δ 7.54 (d, *J* = 7.3, 1H), 7.51-7.43 (m, 5H), 7.41-7.29 (m, 8H), 7.25-7.16 (m, 5H), 5.18 (s, 1H); **^{13}C NMR** (100 MHz, $CDCl_3$) δ 150.4, 143.2, 137.2, 135.8, 134.5, 132.4, 129.9, 129.7, 129.6, 129.5, 128.0, 127.8, 127.2, 126.5. This is a known compound, and the spectroscopic data is in agreement with the literature.[53]

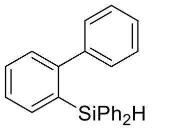

Chemical Formula: $C_{24}H_{20}Si$
Molecular Weight: 336.51

(Biphenyl-2-yl)methylphenylsilane (5v). To a solution of Pd(P(*t*-Bu)$_3$)$_2$ (127.8 mg, 0.25 mmol) in THF (10 mL) were added 2-iodobiphenyl (0.88 mL, 5.0 mmol), triethylamine (1.40 mL, 10.0 mmol), and methylphenylsilane (1.04 mL, 7.5 mmol). After stirring for 2 days at room temperature, the reaction mixture was quenched with water, extracted three times with CH_2Cl_2, and dried over Na_2SO_4. The solvent was evaporated under reduced pressure, and silica gel column chromatography (petroleum ether) produced **5v** (1.17 g, 85%) as a colorless oil. **^{1}H NMR** ($CDCl_3$, 400 MHz) δ 7.64 (dd, *J* = 7.4, 1.5 Hz, 1H), 7.49-7.30 (m, 12H), 7.28-7.25 (m, 1H), 4.83 (q, *J* = 3.9 Hz, 1H), 0.29 (d, *J* = 3.9 Hz, 3H); **^{13}C NMR** (100 MHz, $CDCl_3$) 149.8, 143.6, 136.5, 136.4, 134.9, 134.3, 129.6, 129.5, 129.4, 129.3, 128.0, 127.9, 127.3, 126.5, -4.5. This is a known compound, and the spectroscopic data is in agreement with the literature.[43]

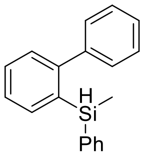

Chemical Formula: $C_{19}H_{18}Si$
Molecular Weight: 274.44

2-(Diethylsilyl)biphenyl (5w). To a solution of Pd(P(*t*-Bu)$_3$)$_2$ (127.8 mg, 0.25 mmol) in THF (10 mL) were added 2-iodobiphenyl (0.88 mL, 5.0 mmol), triethylamine (1.40 mL, 10.0 mmol), and diethylsilane (0.97 mL, 7.5 mmol). After stirring for 2 days at room temperature, the reaction mixture was quenched with water, extracted three times with CH_2Cl_2, and dried over Na_2SO_4. The solvent was evaporated under reduced pressure, and silica gel column chromatography (cyclohexane) produced **5w** (520 mg, 43%) as a colorless oil. **^{1}H NMR** (300 MHz, $CDCl_3$) δ 7.63 (dd, *J* = 7.1, 1.6 Hz, 1H), 7.46-7.29 (m, 8H), 4.07 (quin, *J* = 3.5 Hz, 1H), 0.89 (t, *J* = 7.8 Hz, 6H), 0.61-0.52 (m, 4H); **^{13}C NMR** (100 MHz, $CDCl_3$) δ 149.7, 144.0, 136.1, 134.3, 129.4, 129.3, 129.1, 127.9, 127.2, 126.4, 8.5, 4.2.This is a known compound, and the spectroscopic data is in agreement with the literature.[54]

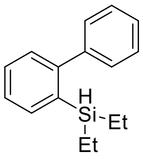

Chemical Formula: $C_{16}H_{20}Si$
Molecular Weight: 240.42

2-(Dimethylgermyl)biphenyl (5x). Following **GP2** from 2-iodobiphenyl (232 mg, 1 mmol, 1.0 equiv.).

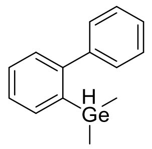

Chemical Formula: $C_{14}H_{16}Ge$
Molecular Weight: 256.91

Purification by silica gel (cyclohexane) afforded **5x** as a colorless oil (218 mg, 85%). **^{1}H NMR** (300 MHz, $CDCl_3$) δ 7.59 (dd, *J* = 7.0, 1.8 Hz, 1H), 7.43-7.31 (m, 8H), 4.38 (sept, *J* = 3.5 Hz, 1H), 0.19 (d, *J* = 3.4 Hz, 6H); **^{13}C NMR** (100 MHz, $CDCl_3$) δ 148.9, 143.9, 138.6, 134.7, 129.34, 129.30, 128.7, 128.1, 127.2, 126.7, -3.1. This is a known compound, and the spectroscopic data is in agreement with the literature.[55]

3.4. Synthesis and characterizations of Final products 6

General procedure D (GP-D)

In an argon-filled glovebox, a dried V-shaped Biotage microwave vial equipped with a stirring bar was charged with $HCo(PMe_3)_4$ (5 mol%) and NBE (1.2 equiv.). A separately prepared, degassed solution of the corresponding biphenyl derivative **5** (1.0 equiv.) in toluene (1M) was then transferred to this mixture. The sealed reaction vessel was stirred for 16 h at 150°C. The reaction was quenched with petroleum ether and the resulting mixture was filtered through a plug of silica/Celite® using AcOEt as eluent. After evaporation of solvents, the crude product was purified by flash chromatography leading to silacycle **6**.

9,9-Dimethyl-9-silafluorene (6a). Following **GP-D** from **5a** (212 mg, 1 mmol, 1.0 equiv.). Purification by silica gel (cyclohexane) afforded **6a** as a white solid (193 mg, 92%). **^{1}H NMR** (400 MHz, $CDCl_3$) δ 7.85 (d, *J* = 7.9 Hz, 2H), 7.65 (d, *J* = 7.1 Hz, 2H), 7.45 (t, *J* = 7.6 Hz, 2H), 7.30 (t, *J* = 7.2 Hz, 2H), 0.45 (s, 6H); **^{13}C NMR** (100 MHz, $CDCl_3$) δ 148.0, 139.1, 132.9, 130.3, 127.5, 121.0, -3.1. This is a known compound, and the spectroscopic data is in agreement with the literature.[15]

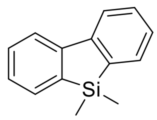

Chemical Formula: $C_{14}H_{14}Si$
Molecular Weight: 210.35

5,5-Dimethyl-3-phenyl-*5H*-dibenzo[*b,d*]silole (6b). Following **GP-D** from **5b** (144 mg, 0.5 mmol, 1.0 equiv.). Purification by silica gel (cyclohexane: ethyl acetate = 100:1) afforded **6b** as a white solid (130 mg, 91%). **^{1}H NMR** (300 MHz, $CDCl_3$) δ 7.92-7.86 (m, 3H), 7.71-7.68 (m, 4H), 7.52-7.45 (m, 3H), 7.41-7.30 (m, 2H), 0.50 (s, 6H); **^{13}C NMR** (100 MHz, $CDCl_3$) δ147.6, 147.1, 141.4, 140.3, 139.8, 139.2, 132.9, 131.6, 130.4, 129.3, 128.9, 127.5, 127.3, 127.2, 121.3, 121.1, -3.0. This is a known compound, and the spectroscopic data is in agreement with the literature.[43]

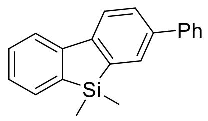

Chemical Formula: $C_{20}H_{18}Si$
Molecular Weight: 286.45

9,9-Dimethyl-2-methyl-9-silafluorene (6c). Following **GP-D** from **5c** (226 mg, 1 mmol, 1.0 equiv.).

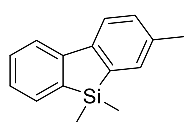

Chemical Formula: $C_{15}H_{16}Si$
Molecular Weight: 224.38

Purification by silica gel (cyclohexane) afforded **6c** as a yellow oil (190 mg, 85%). **^{1}H NMR** (400 MHz, $CDCl_3$) δ 7.79 (d, *J* = 7.8 Hz, 1H), 7.72 (d, *J* = 7.9 Hz, 1H), 7.61 (d, *J* = 8.1 Hz, 1H), 7.45 (s, 1H), 7.42 (td, *J* = 7.6, 1.3 Hz, 1H), 7.27-7.18 (m, 2H), 2.39 (s, 3H), 0.42 (s, 6H); **^{13}C NMR** (100 MHz, $CDCl_3$) δ 148.0, 145.3, 139.1, 138.8, 137.1, 133.6, 132.8, 131.1, 130.3, 127.1, 120.8, 120.6, 21.4, -3.1. This is a known compound, and the spectroscopic data is in agreement with the literature.[43]

9,9-Dimethyl-2- isopropyl -9-silafluorene (6d). Following **GP-D** from **5d** (127 mg, 0.5 mmol, 1.0 equiv.).

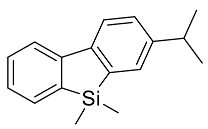

Chemical Formula: $C_{17}H_{20}Si$
Molecular Weight: 252.43

Purification by silica gel (cyclohexane) afforded **6d** as a white solid (115 mg, 91%). **^{1}H NMR** (300 MHz, $CDCl_3$) δ 7.79 (dd, *J* = 10.6, 7.9 Hz, 2H), 7.63 (ddd, *J* = 7.0, 1.4, 0.7 Hz, 1H), 7.51 (d, *J* = 1.9 Hz, 1H), 7.43 (td, *J* = 7.6, 1.4 Hz, 1H), 7.34-7.24 (m, 2H), 2.97 (sept, *J* = 6.9 Hz, 1H), 1.33 (d, *J* = 7.0 Hz, 6H), 0.45 (s, 6H); **^{13}C NMR** (100 MHz, $CDCl_3$) δ 148.10, 148.05, 145.8, 139.2, 139.0, 132.8, 131.0, 130.2, 128.5, 127.0, 120.9, 120.7, 34.2, 24.2, -3.0; **IR (neat, cm^{-1})**: 3046, 2957, 2866, 1589, 1459, 1435, 1248, 1129, 1072, 1057, 841, 774, 747, 715, 652; **HRMS** (APCI): Calcd for $[C_{17}H_{21}Si]^+$: *m/z* 253.1407, Found 253.1406.

9,9-Dimethyl-2-*tert*-butyl-9-silafluorene (6e). Following **GP-D** from **5e** (268 mg, 1 mmol, 1.0 equiv.).

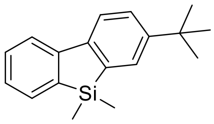

Chemical Formula: $C_{18}H_{22}Si$
Molecular Weight: 266.46

Purification by silica gel (cyclohexane) afforded **6e** as a white solid (226 mg, 85%). **^{1}H NMR** (400 MHz, $CDCl_3$) δ 7.83 (dd, *J* = 14.1, 8.0 Hz, 2H), 7.72 (d, *J* = 2.1 Hz, 1H), 7.67 (d, *J* = 7.0 Hz, 1H), 7.53 (dd, *J* = 8.2, 2.1 Hz, 1H), 7.46 (td, *J* = 7.5, 1.3 Hz, 1H), 7.32-7.29 (m, 1H), 1.44 (s, 9H), 0.50 (s, 6H); **^{13}C NMR** (100 MHz, $CDCl_3$) δ 150.3, 148.0, 145.4, 139.1, 138.8, 132.7, 130.2, 129.5, 127.5, 127.1, 120.7, 120.6, 34.9, 31.6, -2.9. This is a known compound, and the spectroscopic data is in agreement with the literature.[15]

9,9-Dimethyl-2-trifluoromethyl-9-silafluorene (6f). Following **GP-D** from **5f** (280 mg, 1 mmol, 1.0 equiv.).

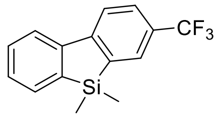

Chemical Formula: $C_{15}H_{13}F_3Si$
Molecular Weight: 278.35

Purification by silica gel (cyclohexane) afforded **6f** as a white solid (267 mg, 96%). **^{1}H NMR** (400 MHz, $CDCl_3$) δ 7.91-7.86 (m, 3H), 7.70-7.66 (m, 2H), 7.48 (td, *J* = 7.6, 1.4 Hz, 1H), 7.35 (td, *J* = 7.3, 1.0 Hz, 1H), 0.46 (s, 6H); **^{13}C NMR** (100 MHz, $CDCl_3$) δ 151.3, 146.5, 140.0, 139.6, 133.1, 130.6, 129.5 (q, *J* = 3.7 Hz), 129.1 (q, *J* = 31.6 Hz), 128.5, 127.4 (q, *J* = 3.7 Hz), 124.6 (q, *J* = 271.4 Hz), 121.7, 120.9, -3.3; **^{19}F NMR** (376 MHz, $CDCl_3$) δ -63.5. This is a known compound, and the spectroscopic data is in agreement with the literature.[15]

9,9-Dimethyl-2-fluoro-9-silafluorene (6g). Following **GP-D** from **5g** (115 mg, 0.5 mmol, 1.0 equiv.). Purification by silica gel (cyclohexane) afforded **6g** as a white solid (103 mg, 90%). **^{1}H NMR** (400 MHz, $CDCl_3$) δ 7.86-7.76 (m, 2H), 7.67-7.63 (m, 1H), 7.47-7.43 (m, 1H), 7.33-7.27 (m, 2H), 7.12 (td, *J* = 8.8, 2.7 Hz, 1H), 0.45 (s, 6H); **^{13}C NMR** (100 MHz, $CDCl_3$) δ 162.9 (d, J_F = 248.3 Hz), 147.1, 143.8, 142.1, 142.0, 138.6, 132.9, 130.5, 127.2, 122.4 (d, J_F = 7.4 Hz), 120.7, 119.2 (d, J_F = 19.7 Hz), 117.1 (d, J_F = 22.2 Hz), -3.2; **^{19}F NMR** (376 MHz, $CDCl_3$) δ -116.6. This is a known compound, and the spectroscopic data is in agreement with the literature.[15]

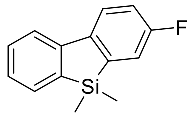

Chemical Formula: $C_{14}H_{13}FSi$
Molecular Weight: 228.34

5,5-Dimethyl-3-(trifluoromethoxy)-*5H*-dibenzo[*b,d*]silole (6h). Following **GP-D** from **5h** (148 mg, 0.5 mmol, 1.0 equiv.). Purification by silica gel (petroleum ether) afforded **6h** as a white solid (140 mg, 95%). **^{1}H NMR** (400 MHz, $CDCl_3$) δ 7.83-7.78 (m, 2H), 7.64 (dd, *J* = 7.1, 1.3 Hz, 1H), 7.47-7.44 (m, 2H), 7.32-7.26 (m, 2H), 0.45 (s, 6H); **^{13}C NMR** (100 MHz, $CDCl_3$) δ 149.0, 146.7, 146.4, 141.7, 139.0, 133.0, 130.6, 127.7, 125.0, 122.9, 122.1, 121.1, 120.8 (q, J_F = 256.7 Hz), -3.3; **^{19}F NMR** (376 MHz, $CDCl_3$) δ -58.6; **IR (neat, cm^{-1})**: 3069, 2956, 2870, 1592, 1470, 1435, 1246, 1217, 1156, 1131, 1060, 1004, 946, 857, 801, 775, 747, 719, 650; **HRMS** (APCI): Calcd for $[C_{15}H_{14}F_3OSi]^+$: *m/z* 295.0761, Found 295.0757.

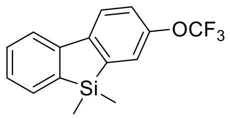

Chemical Formula: $C_{15}H_{13}F_3OSi$
Molecular Weight: 294.35

9,9-Dimethyl-4-methyl-9-silafluorene (6i). Following **GP-D** from **5i** (226 mg, 1 mmol, 1.0 equiv.). Purification by silica gel (cyclohexane) afforded **6i** as a colorless oil (161 mg, 72%). **^{1}H NMR** (300 MHz, $CDCl_3$) δ 8.11 (d, *J* = 8.1 Hz, 1H), 7.71 (dd, *J* = 7.4, 1.2 Hz, 1H), 7.55 (dd, *J* = 6.4, 1.9 Hz, 1H), 7.49 (td, *J* = 7.7, 1.5 Hz, 1H), 7.32 (t, *J* = 7.5 Hz, 1H), 7.25-7.21 (m, 2H), 2.80 (s, 3H), 0.45 (s, 6H); **^{13}C NMR** (100 MHz, $CDCl_3$) δ 149.8, 146.2, 140.6, 140.5, 134.5, 134.4, 132.9, 130.5, 130.1, 127.0, 126.6, 125.8, 24.4, -3.1. This is a known compound, and the spectroscopic data is in agreement with the literature.[56]

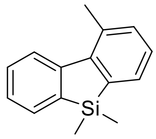

Chemical Formula: $C_{15}H_{16}Si$
Molecular Weight: 224.38

9,9-Dimethyl-4-methoxyl-9-silafluorene (6j). Following **GP-D** from **5j** (121mg, 0.5 mmol, 1.0 equiv.). Purification by silica gel (cyclohexane) afforded **6j** as a colorless oil (110 mg, 92%). **^{1}H NMR** (400 MHz, $CDCl_3$) δ 8.60 (d, *J* = 8.0 Hz, 1H), 7.65 (d, *J* = 6.9 Hz, 1H), 7.46 (td, *J* = 7.6, 1.4 Hz, 1H), 7.33-7.26 (m, 3H), 7.04 (dd, *J* = 7.8, 1.4 Hz, 1H), 4.01 (s, 3H), 0.45 (s, 6H); **^{13}C NMR** (100 MHz, $CDCl_3$) δ 157.5, 147.9, 142.0, 139.1, 135.5, 132.2, 130.3, 128.6, 126.9, 126.5, 124.7, 113.6, 55.3, -3.1. This is a known compound, and the spectroscopic data is in agreement with the literature.[15]

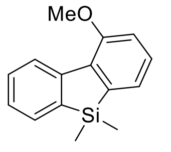

Chemical Formula: $C_{15}H_{16}OSi$
Molecular Weight: 240.38

9,9-Dimethyl-4- ethyl -9-silafluorene (6k). Following **GP-D** from **5k** (120 mg, 0.5 mmol, 1.0 equiv.). Purification by silica gel (cyclohexane) afforded **6k** as a colorless oil (105 mg, 88%). **^{1}H NMR** (300 MHz, $CDCl_3$) δ 8.03 (d, *J* = 8.1 Hz, 1H), 7.67 (dd, *J* = 6.8, 1.1 Hz, 1H), 7.52-7.43 (m, 2H), 7.31-7.26 (m, 2H), 7.24-7.21 (m, 1H), 3.14 (q, *J* = 7.5 Hz, 2H), 1.41 (t, *J* = 7.5 Hz, 3H), 0.41 (s, 6H); **^{13}C NMR** (100 MHz, $CDCl_3$) δ 149.4, 145.5, 141.1, 140.70, 140.67, 132.9, 132.8, 130.5, 130.2, 127.3, 126.6, 125.8, 29.1, 14.8, -3.0; **IR (neat, cm^{-1})**: 3053, 2966, 2885, 1588, 1465, 1441, 1404, 1246, 1133, 1060, 858, 803, 772, 745, 732, 670, 643; **HRMS** (APCI): Calcd for $[C_{16}H_{19}Si]^+$: *m/z* 239.1251, Found 239.1250.

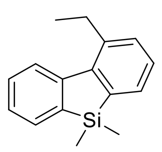

Chemical Formula: $C_{16}H_{18}Si$
Molecular Weight: 238.41

9,9-Dimethyl-3-methyl-9-silafluorene (6l). Following **GP-D** from **5l** (226 mg, 1 mmol, 1.0 equiv.). Purification by silica gel (cyclohexane) afforded **6l** as a colorless oil (217 mg, 97%). **^{1}H NMR** (400 MHz, $CDCl_3$) δ 7.85 (d, *J* = 7.7 Hz, 1H), 7.69 (s, 1H), 7.65 (d, *J* = 7.0 Hz, 1H), 7.56 (d, *J* = 7.2 Hz, 1H), 7.45 (td, *J* = 7.6, 1.3 Hz, 1H), 7.30 (t, *J* = 7.2 Hz, 1H), 7.15 (d, *J* = 7.3 Hz, 1H), 2.46 (s, 3H), 0.44 (s, 6H); **^{13}C NMR** (100 MHz, $CDCl_3$) δ 148.3, 147.9, 140.2, 139.5, 135.5, 132.81, 132.77, 130.2, 128.4, 127.4, 121.8, 120.8, 22.0, -3.0. This is a known compound, and the spectroscopic data is in agreement with the literature.[15]

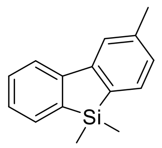

Chemical Formula: $C_{15}H_{16}Si$
Molecular Weight: 224.38

5,5-Dimethyl-2,4-bis(trifluoromethyl)-*5H*-dibenzo[*b,d*]silole (6m). Following **GP-D** from **5m** (174 mg, 0.5 mmol, 1.0 equiv.). Purification by silica gel (cyclohexane) afforded **6m** as a white solid (161 mg, 93%) with traces of starting material. **^{1}H NMR** (400 MHz, $CDCl_3$) δ 8.20 (s, 1H), 7.90 (d, *J* = 7.8 Hz, 1H), 7.78 (s, 1H), 7.68 (dt, *J* = 7.0, 1.1 Hz, 1H), 7.51 (td, *J* = 7.6, 1.4 Hz, 1H), 7.40 (td, *J* = 7.3, 1.0 Hz, 1H), 0.51 (d, *J* = 1.2 Hz, 6H); **^{13}C NMR** (100 MHz, $CDCl_3$) δ 150.6, 144.8, 139.3, 135.9 (q, J_F = 31 Hz), 133.1 (q, J_F = 32.1 Hz), 132.9, 130.8, 129.3, 128.4 (q, J_F = 204 Hz), 123.5 (q, J_F = 271 Hz), 121.6, 121.0-120.9 (m), 120.4 (q, J_F = 4.0 Hz), -3.3 (q, J_F = 2.4 Hz); **^{19}F NMR** (376 MHz, $CDCl_3$) δ -62.0, -64.1; **IR (neat, cm^{-1})**: 3063, 2957, 2872, 1618, 1455, 1370, 1275, 1187, 1123, 1077, 895, 860, 808, 777, 757, 727, 711, 640; **HRMS** (APCI): Calcd for $[C_{16}H_{12}F_6Si]^+$: *m/z* 346.0607, Found 346.0607.

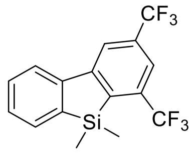

Chemical Formula: $C_{16}H_{12}F_6Si$
Molecular Weight: 346.35

8-Methoxy-5,5-dimethyl-*5H*-benzo[*b*]naphtho[*2,3-d*]silole (6o). Following **GP-D** from **5o** (146 mg, 0.5 mmol, 1.0 equiv.). Purification by silica gel (dichloromethane/petroleum ether (10:90–30:70)) afforded **6o** as a white solid (136 mg, 94%). **^{1}H NMR** (400 MHz, $CDCl_3$) δ 8.17 (s, 1H), 8.03 (s, 1H), 7.98 (d, *J* = 7.8 Hz, 1H), 7.79-7.77 (m, 1H), 7.67 (dt, *J* = 7.0, 1.0 Hz, 1H), 7.48 (td, *J* = 7.6, 1.3 Hz, 1H), 7.30 (td, *J* = 7.2, 0.9 Hz, 1H), 7.18-7.15 (m, 2H), 3.94 (s, 3H), 0.49 (s, 6H); **^{13}C NMR** (100 MHz, $CDCl_3$) δ 157.7, 147.9, 142.6, 139.2, 137.9, 134.3, 132.8, 132.2, 130.34, 130.25, 129.8, 127.3, 121.0, 119.2, 118.8, 106.3, 55.3, -2.7; **IR (neat, cm^{-1})**: 3059, 3006, 2958, 1621, 1595, 1387, 1230, 1164, 1127, 1057, 1028, 890, 837, 810, 773, 750, 719, 658; **HRMS** (APCI): Calcd for $[C_{19}H_{19}OSi]^+$: *m/z* 291.1200, Found 291.1198.

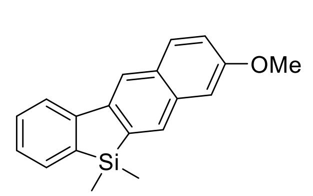

Chemical Formula: $C_{19}H_{18}OSi$
Molecular Weight: 290.44

8,8-Dimethylbenzosilolo[*2,3-a*]ferrocene (6p). Following **GP-D** from **5p** (160 mg, 0.5 mmol, 1.0 equiv.). Purification by silica gel (petroleum ether) afforded **6p** as an orange solid, (143 mg, 90%). **^{1}H NMR** (400 MHz, $CDCl_3$) δ 7.48 (dt, *J* = 7.0, 1.0 Hz, 1H), 7.39 (dt, *J* = 7.6, 0.9 Hz, 1H), 7.31 (td, *J* = 7.5, 1.3 Hz, 1H), 7.18 (td, *J* = 7.2, 1.1 Hz, 1H), 4.83-4.82 (m, 1H), 4.57 (t, *J* = 2.3 Hz, 1H), 4.41 (dd, *J* = 2.2, 0.8 Hz, 1H), 4.03 (s, 5H), 0.72 (s, 3H), 0.37 (s, 3H); **^{13}C NMR** (100 MHz, $CDCl_3$) δ 147.9, 142.2, 132.5, 129.6, 125.4, 121.0, 97.1, 73.3, 71.8, 70.6, 69.8, 64.2, -0.6, -1.5. This is a known compound, and the spectroscopic data is in agreement with the literature.[18]

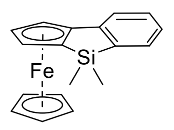

Chemical Formula: $C_{18}H_{18}FeSi$
Molecular Weight: 318.27

7,7-Dimethyl- *7H*-benzo[*b*]naphtho[*1,2-d*]silole (6q). Following **GP-D** from **5q** (131 mg, 0.5 mmol, 1.0 equiv.). Purification by silica gel (petroleum ether) afforded **6q** as a white solid (120 mg, 92%). **^{1}H NMR** (400 MHz, $CDCl_3$) δ 8.93 (d, *J* = 8.6 Hz, 1H), 8.50 (d, *J* = 8.0 Hz, 1H), 7.95 (dd, *J* = 8.6, 1.3 Hz, 1H), 7.82 (d, *J* = 7.8 Hz, 1H), 7.78-7.73 (m, 2H), 7.62 (ddd, *J* = 8.6, 6.7, 1.6 Hz, 1H), 7.55 (td, *J* = 7.5, 1.4 Hz, 2H), 7.36 (t, *J* = 7.2 Hz, 1H), 0.51 (s, 6H); **^{13}C NMR** (100 MHz, $CDCl_3$) δ 149.6, 145.1, 140.8, 139.3, 136.1, 133.1, 130.2, 130.0, 129.4, 128.3, 128.0, 126.8, 126.5, 126.0, 125.9, 124.9, -3.4; **IR (neat, cm^{-1})**: 3049, 2951, 2868, 1577, 1440, 1343, 1244, 1128, 1063, 1026, 965, 871, 835, 799, 773, 746, 714, 660; **HRMS** (APCI): Calcd for $[C_{18}H_{17}Si]^+$: *m/z* 261.1094, Found 261.1093.

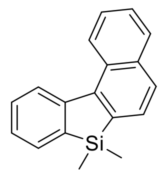

Chemical Formula: $C_{18}H_{16}Si$
Molecular Weight: 260.41

5,5,5',5'-Tetramethyl-*5H,5'H*-3,3'-bidibenzo[*b,d*]silole (6r). Following **GP-D** from **5r** (211 mg, 0.5 mmol, 1.0 equiv.). Purification by silica gel (dichloromethane : cyclohexane = 1:9) afforded **6r** as a white solid, (186 mg, 89%). **^{1}H NMR** (400 MHz, $CDCl_3$) δ 7.93-7.87 (m, 6H), 7.74 (dd, *J* = 8.0, 2.0 Hz, 2H), 7.68 (d, *J* = 7.1 Hz, 2H), 7.47 (td, *J* = 7.5, 1.3 Hz, 2H), 7.31 (t, *J* = 7.2 Hz, 2H), 0.50 (s, 12H); **^{13}C NMR** (100 MHz, $CDCl_3$) δ 147.6, 147.0, 140.2, 139.7, 139.1, 132.8, 131.3, 130.3, 129.1, 127.4, 121.2, 120.9, -3.1; **IR (neat, cm^{-1})**: 2917, 2849, 1587, 1463, 1430, 1245, 1130, 1062, 862, 828, 771, 747, 714, 651; **HRMS** (ESI): Calcd for $[C_{28}H_{27}Si_2]^+$: *m/z* 419.1646, Found 419.1648.

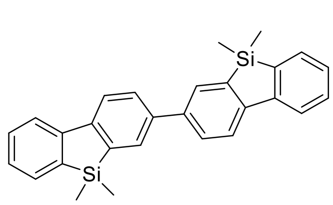

Chemical Formula: $C_{28}H_{26}Si_2$
Molecular Weight: 418.69

6,6,12,12-Tetramethyl-6,12-disilaindeno[*1,2-b*]fluorene (6s). Following **GP-D** from **5s** (173 mg, 0.5 mmol, 1.0 equiv.). Purification by silica gel (cyclohexane: ethyl acetate = 100:1) afforded **6s** as a white solid, (145 mg, 85%). **^{1}H NMR** (400 MHz, $CDCl_3$) δ 8.12 (s, 2H), 7.91 (d, *J* = 7.8 Hz, 2H), 7.65 (d, *J* = 7.0 Hz, 2H), 7.45 (td, *J* = 7.6, 1.4 Hz, 2H), 7.28 (td, *J* = 7.2, 0.9 Hz, 2H), 0.49 (s, 12H) ; **^{13}C NMR** (100 MHz, $CDCl_3$) δ 147.9, 147.0, 141.6, 139.1, 132.9, 130.3, 127.3, 125.0, 120.9, -3.0. This is a known compound, and the spectroscopic data is in agreement with the literature.[15]

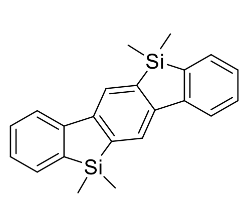

Chemical Formula: $C_{22}H_{22}Si_2$
Molecular Weight: 342.59

9,9′-Spiro-9-silabifluorene (6t). Following **GP-D** from **5t** (336 mg, 1 mmol, 1.0 equiv.). Purification by silica gel (petroleum ether) afforded **6t** as a white solid, (312 mg, 94%). **^{1}H NMR** (400 MHz, $CDCl_3$) d 7.97 (d , *J* = 7.8 Hz, 4H), 7.53 (td , *J* = 7.6, 1.3 Hz, 4H), 7.45 (dd , *J* = 7.2, 1.3 Hz, 4H), 7.26-7.22 (m, 4H); **^{13}C NMR** (100 MHz, $CDCl_3$) d 150.1, 134.5, 132.8, 131.5, 128.0, 121.2. This is a known compound, and the spectroscopic data is in agreement with the literature.[19]

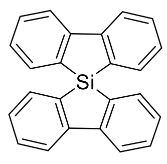

Chemical Formula: $C_{24}H_{16}Si$
Molecular Weight: 332.48

5,5-Diphenyl-*5H*-dibenzo[*b,d*]silole (6u). Following **GP-D** from **5u** (168 mg, 0.5 mmol, 1.0 equiv.). Purification by silica gel (cyclohexane) afforded **6u** as a white solid, (38 mg, 23%). **^{1}H NMR** (300 MHz, $CDCl_3$) δ 7.91 (d, *J* = 7.7 Hz, 2H), 7.80 (d, *J* = 7.1 Hz, 2H), 7.69-7.66 (m, 4H), 7.50 (td, *J* = 7.4, 1.6 Hz, 2H), 7.46-7.31 (m, 8H); **^{13}C NMR** (100 MHz, $CDCl_3$) δ 148.9, 136.1, 135.6, 134.1, 132.9, 130.9, 130.2, 128.2, 127.9, 121.3. This is a known compound, and the spectroscopic data is in agreement with the literature.[57]

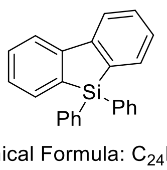

Chemical Formula: $C_{24}H_{18}Si$
Molecular Weight: 334.49

9-Methyl-9-phenyl-9-silafluorene (6v). Following **GP-D** from **5v** (274 mg, 1 mmol, 1.0 equiv.).

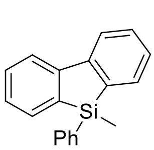

Chemical Formula: $C_{19}H_{16}Si$
Molecular Weight: 272.42

Purification by silica gel (dichloromethane/petroleum ether = 10:90) afforded **6v** as a white solid, (250 mg, 92%). **^{1}H NMR** (400 MHz, $CDCl_3$) δ 7.97 (d, *J* = 7.8 Hz, 2H), 7.77 (d, *J* = 8.5 Hz, 2H), 7.70-7.67 (m, 2H), 7.56 (td, *J* = 7.6, 1.4 Hz, 2H), 7.49-7.36 (m, 5H), 0.86 (s, 3H); **^{13}C NMR** (100 MHz, $CDCl_3$) δ 148.5, 137.5, 134.7, 134.6, 133.5, 130.6, 130.0, 128.2, 127.7, 121.1, -4.9. This is a known compound, and the spectroscopic data is in agreement with the literature.[15]

9,9-Diethyl-9-silafluorene (6w). Following **GP-D** from **5w** (240 mg, 1 mmol, 1.0 equiv.). Purification by

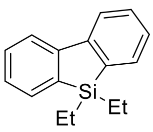

Chemical Formula: $C_{16}H_{18}Si$
Molecular Weight: 238.41

silica gel (cyclohexane) afforded **6w** as a white solid, (212 mg, 89%). **^{1}H NMR** (400 MHz, $CDCl_3$) δ 7.85 (d, *J* = 7.8 Hz, 2H), 7.64 (dd, *J* = 7.0, 1.3 Hz, 2H), 7.45 (td, *J* = 7.6, 1.4 Hz, 2H), 7.28 (t, *J* = 7.2 Hz, 2H), 1.02 (t, *J* = 7.0 Hz, 6H), 0.99-0.92 (m, 4H); **^{13}C NMR** (100 MHz, $CDCl_3$) δ 148.7, 137.4, 133.4, 130.2, 127.3, 120.9, 7.7, 3.9. This is a known compound, and the spectroscopic data is in agreement with the literature.[15]

9,9-Dimethyl-9-germafluorene (6x). Following **GP-D** from **5x** (129 mg, 0.5 mmol, 1.0 equiv.). The reaction mixture was stirred for 64 h at 150°C. Purification by silica gel (cyclohexane) afforded **6x** as a

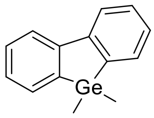

Chemical Formula: $C_{14}H_{14}Ge$
Molecular Weight: 254.90

white solid, (90 mg, 70%). **^{1}H NMR** (300 MHz, $CDCl_3$) δ 7.88 (d, *J* = 7.8 Hz, 2H), 7.64 (dd, *J* = 7.1, 1.4 Hz, 2H), 7.42 (td, *J* = 7.6, 1.4 Hz, 2H), 7.29 (t, *J* = 7.2 Hz, 2H), 0.60 (s, 6H); **^{13}C NMR** (100 MHz, $CDCl_3$) δ 146.5, 141.3, 133.0, 129.5, 127.6, 121.5, -2.6. This is a known compound, and the spectroscopic data is in agreement with the literature.[55]

3.5. DFT calculations

All DFT calculations were carried out using the Gaussian 16[58] software package. Optimization of geometries and frequencies were performed using the PBE0-D3(BJ) functional with the Def2-SVP basis set for all atoms. Thermal corrections to Gibbs Free energy at 423.15 K were also obtained at this level of theory. Single points were recorded using the PBE0-D3(BJ) functional with the triple-ζ valence def2-TZVP basis set for all atoms. Solvation effect (Toluene) using the SMD model was obtained at the same level of theory for the single-point energies calculations. Standard state energy correction (+ 0.00475363 Hartree at 423.15 K) from gas-phase (1 atm) to condensed-phase (1 M) was also made for all the given structures.

Figure S3. Full Mechanism for the transformation of **5a** into **6a**

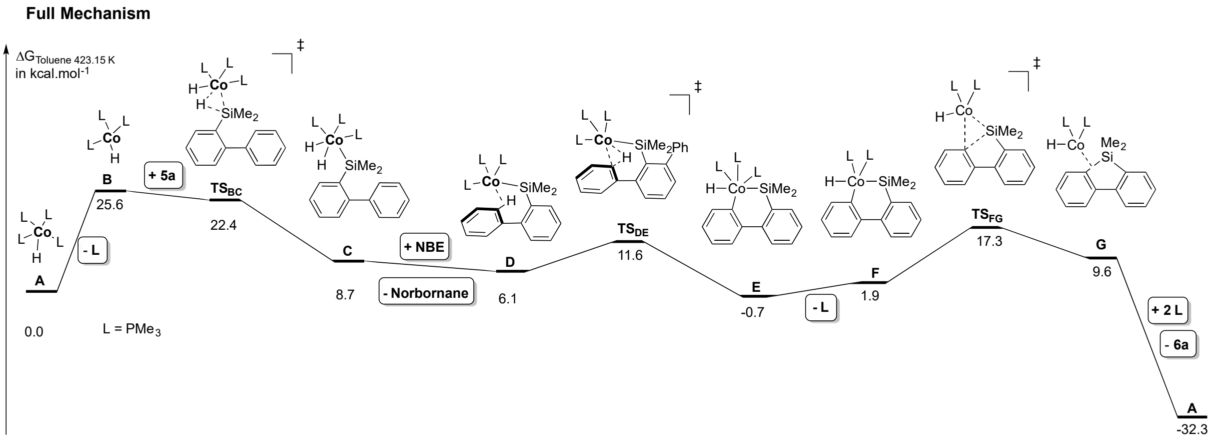

Table S5. Geometries (CylView), Electronic Energies (in Hartrees), and Cartesian coordinates (x,y,z) of the computed species

**A**	**PMe₃**
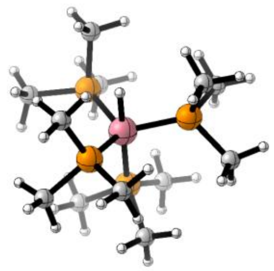<br>E(PBE0-D3) = -3226.807902<br>Thermal correction for Gibbs Energy = 0.366603<br>$N_{imag} = 0$	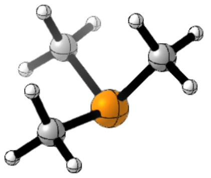<br>E(PBE0-D3) = -3226.807902<br>Thermal correction for Gibbs Energy = 0.06687<br>$N_{imag} = 0$
P -1.89225800 -0.25848100 -0.29310200	P -2.23178800 -0.20711800 -0.00051700
P 0.99853000 0.85949500 1.53759100	C -1.35313700 -1.83528200 0.00003500
H 0.43289600 -0.03671000 -0.56205400	H -1.65295000 -2.41440600 0.88681800
C -2.80370200 1.25484900 0.26568600	H -1.64968300 -2.41324600 -0.88860100
H -2.13105300 2.12052500 0.17070600	H -0.25590300 -1.72457700 0.00212600
H -3.72228800 1.44732900 -0.31353200	C -1.35576100 0.60594200 1.41175600
H -3.06317900 1.15196900 1.32963800	H -1.65393900 1.66399500 1.46898900
C -3.31925900 -1.44235800 -0.33723800	H -1.65606300 0.12591600 2.35571500
H -3.66354300 -1.66506900 0.68358000	H -0.25831400 0.55049800 1.31829200
H -4.17202800 -1.04951700 -0.91460600	C -1.35061000 0.60776200 -1.40853200
H -2.98335300 -2.38610300 -0.79294900	H -1.64746500 0.12896200 -2.35420200
C -1.80457000 0.11435000 -2.09760800	H -1.64856700 1.66589200 -1.46548200
H -2.78863500 0.35993100 -2.52861700	H -0.25351300 0.55218000 -1.31113200
H -1.11495500 0.95712600 -2.24973900	
H -1.38532900 -0.75667200 -2.62275700	
C 2.72304700 0.54764000 2.13891800	
H 2.70260200 -0.23320300 2.91326900	
H 3.32157600 0.16552900 1.29827700	
H 3.20990700 1.44914100 2.54705400	
C 1.33466000 2.31581100 0.45630200	
H 1.90267000 1.97829800 -0.42258900	
H 0.37778900 2.72284000 0.09705800	
H 1.89540400 3.10959700 0.97591100	
C 0.34910100 1.77236900 3.01537000	

H	-0.64964000	2.16750700	2.77582100
H	0.24501400	1.08932100	3.87136400
H	1.00321000	2.60791700	3.31356000
Co	-0.05949200	-0.73120400	0.64087700
P	1.08564400	-2.27293700	-0.23510900
C	2.16010600	-3.40397000	0.76751800
H	1.55012900	-3.98121000	1.47803800
H	2.72592000	-4.11102300	0.13914600
H	2.86926000	-2.79535100	1.34875800
C	2.34728400	-1.73898600	-1.47027200
H	2.90705600	-2.58620200	-1.89848800
H	1.84064700	-1.18745500	-2.27533300
H	3.05128300	-1.04654000	-0.98538900
C	0.21161300	-3.54921400	-1.25499200
H	0.89567200	-4.28385100	-1.71185400
H	-0.51957500	-4.07900100	-0.62700800
H	-0.34691500	-3.03463600	-2.05127600
P	-0.77380800	-1.73536200	2.38155000
C	-2.15301500	-0.96395100	3.33796800
H	-2.40285200	-1.53799600	4.24426100
H	-1.87201400	0.05903000	3.62335100
H	-3.04732400	-0.90033300	2.70162400
C	-1.46231000	-3.43976100	2.19698200
H	-1.85489400	-3.83377500	3.14773900
H	-2.26710800	-3.43065200	1.44918500
H	-0.67449400	-4.11505900	1.83336400
C	0.40694800	-2.04645400	3.76769800
H	-0.05282000	-2.62756500	4.58270500
H	1.28279000	-2.58857200	3.38570000
H	0.75743200	-1.08628000	4.17273800

B

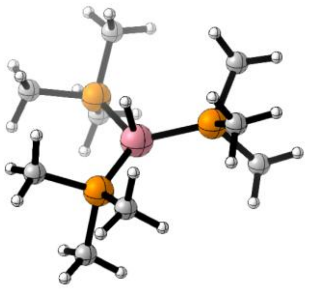

E(PBE0-D3) = -2765.848057
Thermal correction for Gibbs Energy = 0.257583
$N_{imag} = 0$

P	-1.92549500	-0.35197800	-0.36236200
P	0.91918000	0.94659600	1.55754600
H	0.50208400	0.06529200	-0.73697900
C	-2.63920500	1.34942300	-0.48772100
H	-1.93656600	1.98831600	-1.04285500
H	-3.61962200	1.36633100	-0.99220600
H	-2.74737300	1.76883500	0.52365000
C	-3.33881900	-1.21258900	0.46506800
H	-3.41612200	-0.87161400	1.50881100
H	-4.29878300	-1.02282000	-0.04197000
H	-3.14718800	-2.29612300	0.47496500
C	-2.19231600	-0.89823100	-2.10453700

Silane 5a

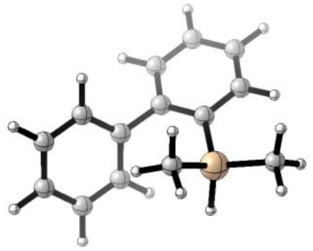

E(PBE0-D3) = -832.114436
Thermal correction for Gibbs Energy = 0.18722
$N_{imag} = 0$

Si	0.30695300	1.57181900	1.68963400
C	0.32576400	0.80152200	3.40787900
H	1.17024900	0.10810900	3.54212300
H	-0.60118100	0.22707200	3.56241300
H	0.38055400	1.57016900	4.19487000
C	-1.22703000	2.64339000	1.50576800
H	-1.17754000	3.50288800	2.19282100
H	-2.12619500	2.05781000	1.75488100
H	-1.33991900	3.02940100	0.48264200
C	2.09827900	3.47581800	0.37329300
C	1.89402800	2.58908000	1.45595500
C	2.87994400	2.53677000	2.45357900

H	-3.20982100	-0.67736600	-2.46681600	C	4.02598200	3.32844200	2.40128100
H	-1.46031000	-0.38865600	-2.74830500	C	4.20490800	4.21003200	1.33923500
H	-2.00658800	-1.97914800	-2.17663600	C	3.24309500	4.28154800	0.33518400
C	2.76111100	1.10792100	1.57871000	H	2.74846300	1.86691400	3.30711100
H	3.19465300	0.20870600	2.04117800	H	4.77431500	3.26030700	3.19464300
H	3.12401100	1.16086400	0.54162600	H	5.09632700	4.83999700	1.28729800
H	3.10621200	1.99912600	2.12883400	H	3.38390700	4.96030500	-0.50999800
C	0.49147400	2.67718200	1.08059000	C	1.11384000	3.58649900	-0.73278200
H	0.72021900	2.81426500	0.01343500	C	0.76490400	2.46769300	-1.50028400
H	-0.58829700	2.83289700	1.21446300	C	-0.18219700	2.57069800	-2.51673200
H	1.04429700	3.42769100	1.66923000	C	-0.79123100	3.79540800	-2.78620400
C	0.58818400	1.02266300	3.37622800	C	-0.43769000	4.92004800	-2.04092000
H	-0.49640400	1.10197800	3.54423900	C	0.51021600	4.81681000	-1.02576500
H	0.93885300	0.09403800	3.85194600	H	-0.44293700	1.68774900	-3.10522600
H	1.08902600	1.87855400	3.85713200	H	-1.53758000	3.87510100	-3.58012600
Co	0.01468000	-0.62217400	0.45243000	H	-0.90925400	5.88398800	-2.24718800
P	1.19609300	-2.26558700	-0.18469300	H	0.77306700	5.69391200	-0.42907500
C	1.40357600	-3.68325100	0.98580700	H	1.24835300	1.50964600	-1.29879500
H	0.41770000	-4.11223900	1.22170100	H	0.24889100	0.44795000	0.69948000
H	2.04567500	-4.47733900	0.57136300				
H	1.84762900	-3.31634600	1.92337900				
C	2.96276400	-1.92851300	-0.59730400				
H	3.48874100	-2.82376400	-0.96772800				
H	3.00033900	-1.14278100	-1.36614500				
H	3.47725800	-1.54791500	0.29621700				
C	0.72629500	-3.20031500	-1.71006300				
H	1.45343800	-3.98737500	-1.97048600				
H	-0.26273000	-3.65867900	-1.56069900				
H	0.64560800	-2.49241800	-2.54821100				

Norbornene

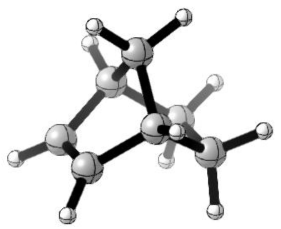

E(PBE0-D3) = -272.516636
Thermal correction for Gibbs Energy = 0.109575
$N_{imag} = 0$

C	-1.17794500	0.35399000	0.02480900
C	0.33324600	0.26013800	0.02871700
C	-0.20029900	2.43783600	0.02874600
C	-1.49695600	1.65616900	0.02488400
C	0.68395900	1.50026400	0.86470900
H	1.75425000	1.76249000	0.83289700
H	0.35150100	1.41880700	1.91008900
C	0.79506600	0.73017800	-1.38129400
H	1.88017600	0.58327000	-1.49757900
H	0.29935200	0.16426700	-2.18317100
C	0.42650600	2.23447500	-1.38130100
H	1.32081100	2.86634000	-1.49763000
H	-0.27468600	2.50707900	-2.18317900
H	0.75970400	-0.70028400	0.34865400

Norbornane

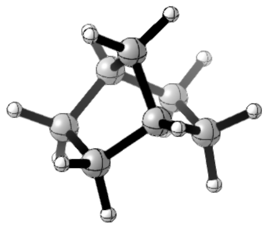

E(PBE0-D3) = -273.754311
Thermal correction for Gibbs Energy = 0.13253
$N_{imag} = 0$

C	-1.19021700	0.24195400	0.00416500
C	0.34596000	0.25896000	-0.00780500
C	-0.18965800	2.44476000	-0.00763200
C	-1.55960500	1.74951800	0.00370300
H	-1.60125200	-0.30156000	-0.86054000
H	-1.56751400	-0.26042900	0.90844200
H	-2.17468700	2.04112200	-0.86157900
H	-2.12707800	2.02102700	0.90742700
C	0.64538000	1.49079300	0.85664300
H	1.71515700	1.75296300	0.88650100
H	0.27317800	1.39947200	1.88967600
C	0.81553200	0.73360500	-1.39148400
H	1.90112700	0.58897300	-1.50556200
H	0.32947000	0.17232500	-2.20437600

H -0.26598800 3.48663600 0.34865600<br>H -2.49212400 2.09183200 -0.08638300<br>H -1.85909100 -0.49230800 -0.08638800	C 0.44662400 2.24131600 -1.39122100<br>H 1.34286600 2.87087000 -1.50461900<br>H -0.24332800 2.51507600 -2.20430400<br>H 0.80360300 -0.68700300 0.31670300<br>H -0.22129900 3.49507300 0.31706400
**H_2**<br>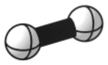<br>E(PBE0-D3) = -1.167696<br>Thermal correction for Gibbs Energy = 0.008022<br>$N_{imag} = 0$	**Silafluorene 6a**<br>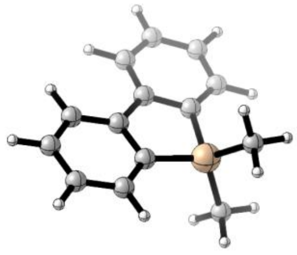<br>E(PBE0-D3) = -830.935015<br>Thermal correction for Gibbs Energy = 0.171082<br>$N_{imag} = 0$
H 0.10028200 1.29834300 0.00000000<br>H -0.66050300 1.29834300 0.00000000	Si 1.49566200 -2.37000900 -1.10528700<br>C 0.57987600 -0.86336700 -1.75077100<br>H 0.92746900 0.04694500 -1.24010200<br>H -0.50456400 -0.96035500 -1.58329400<br>H 0.74404600 -0.73636900 -2.83260800<br>C 0.90761100 -3.92740000 -1.97366500<br>H 1.07901700 -3.86119200 -3.05979600<br>H -0.17037200 -4.08560900 -1.81129500<br>H 1.44637700 -4.80769100 -1.59272300<br>C 3.84409300 -2.21399600 0.21492900<br>C 3.36599700 -2.16924000 -1.11622600<br>C 4.26782000 -1.99700300 -2.16558000<br>C 5.63449500 -1.86891300 -1.91086700<br>C 6.10122000 -1.91351000 -0.59692900<br>C 5.21374800 -2.08509200 0.46393300<br>H 3.90966200 -1.96085800 -3.19904200<br>H 6.33756800 -1.73412100 -2.73646600<br>H 7.17070600 -1.81335300 -0.39560500<br>H 5.59775400 -2.11759300 1.48620200<br>C 2.80265000 -2.40074900 1.25549900<br>C 1.47749800 -2.50792900 0.77068500<br>C 0.42790700 -2.68561200 1.67116700<br>C 0.67649200 -2.75800200 3.04305000<br>C 1.98450600 -2.65174300 3.51639600<br>C 3.04553200 -2.47390500 2.63035300<br>H -0.60096400 -2.76974800 1.30787300<br>H -0.14929900 -2.89735500 3.74500700<br>H 2.18102700 -2.70814900 4.58997000<br>H 4.06312800 -2.39279200 3.01954200
**TS_{BC}**	**C**

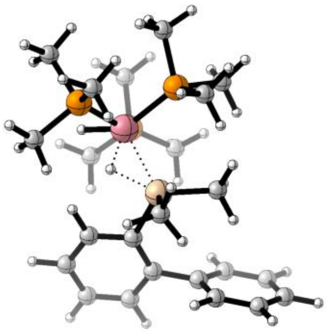	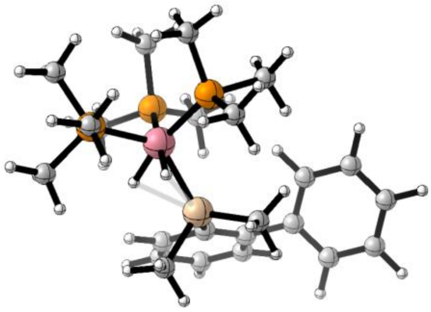
E(PBE0-D3) = -3598.009207<br>Thermal correction for Gibbs Energy = 0.491107<br>N_{imag} = 1 (-173.18 cm^{-1})	E(PBE0-D3) = -3598.035371<br>Thermal correction for Gibbs Energy = 0.495497<br>N_{imag} = 0

Co	1.32374200	10.41846800	6.43140000	Co	1.44025500	10.46142200	6.50101500
P	1.71351900	11.19075200	8.38063600	P	1.44121700	11.79175100	8.20245700
P	2.66039800	11.71929400	5.37132500	P	2.48896000	11.58026900	4.98465000
P	-0.35767500	10.46476400	5.12066100	P	-0.58338000	10.61499600	5.75064600
Si	2.24106600	8.39081100	5.71707300	Si	2.12004300	8.34267900	6.29621000
C	3.41976300	11.59253500	8.96770700	C	3.06509600	12.53221500	8.65962100
H	4.06248200	10.71535800	8.80081600	H	3.79863400	11.72090200	8.77248900
H	3.42572600	11.84384700	10.03978400	H	3.00797200	13.10671800	9.59778300
H	3.84204300	12.43939900	8.40885300	H	3.41848200	13.19859800	7.85920700
C	0.81006900	12.70038000	8.92966700	C	0.37377900	13.29356600	8.35526800
H	1.05398800	13.54733500	8.27399700	H	0.59851500	13.99333900	7.53668600
H	1.04649100	12.96938300	9.97154700	H	0.52601100	13.81067000	9.31623600
H	-0.26837900	12.50604800	8.83842900	H	-0.68582800	13.00875000	8.27535500
C	1.23237200	10.02487300	9.71878300	C	1.03052300	10.94846500	9.78322500
H	1.81018300	9.09591200	9.60667200	H	1.70786800	10.09035500	9.90116800
H	0.16695400	9.77617700	9.61446500	H	0.00511200	10.55541800	9.72719100
H	1.41573400	10.45110500	10.71714700	H	1.12119800	11.61672200	10.65451100
C	4.48542800	11.48057700	5.53374900	C	4.32026000	11.51423400	5.08088900
H	4.76854100	10.50589200	5.11387900	H	4.64679100	10.47385000	4.95555500
H	4.76034800	11.47385300	6.59626600	H	4.63878000	11.85107500	6.07741600
H	5.05022900	12.27350200	5.01862900	H	4.79208200	12.14487900	4.31161100
C	2.56932900	13.51359800	5.82054800	C	2.25293800	13.41206700	4.92909000
H	1.53594100	13.86634500	5.68988200	H	1.19368200	13.65452600	4.77057200
H	3.24187700	14.12373900	5.19673400	H	2.84988500	13.87435500	4.12731800
H	2.84469800	13.65847100	6.87416900	H	2.55814600	13.84923500	5.89081400
C	2.54251500	11.93821600	3.54233400	C	2.23217000	11.18441100	3.20690800
H	1.56080000	12.36040800	3.28472500	H	1.16894300	11.29552000	2.94826200
H	2.65229700	10.97517700	3.02997400	H	2.51750800	10.13802600	3.03603900
H	3.32297300	12.62763900	3.18533000	H	2.82961300	11.83615000	2.55030000
C	-1.86220200	9.56384200	5.67398700	C	-1.86617800	10.25489900	7.01612700
H	-1.62216000	8.49888600	5.79796300	H	-1.75419900	9.20809700	7.33086100
H	-2.68068500	9.67118200	4.94527600	H	-2.88288600	10.40841800	6.62289000
H	-2.18307500	9.95771900	6.64878200	H	-1.71513000	10.89760900	7.89506300
C	-1.09277300	12.13870300	4.86127700	C	-1.17797600	12.24903300	5.13040400
H	-1.31281800	12.57527900	5.84635600	H	-0.97327100	13.03526600	5.86939700
H	-2.01760500	12.09926600	4.26319700	H	-2.25807200	12.22630600	4.91807000
H	-0.36967800	12.79578600	4.35672100	H	-0.64872000	12.50499700	4.20064700
C	-0.31849800	9.84160400	3.38380600	C	-1.19832400	9.56214100	4.37567600
H	-0.10574600	8.76257400	3.41163000	H	-1.02643100	8.50396600	4.61401300
H	0.47004200	10.33216900	2.80026600	H	-0.65224900	9.80294500	3.45261700
H	-1.28821800	10.00313900	2.88785900	H	-2.27291000	9.72909000	4.20314000
C	0.94303400	6.95675800	5.82499700	C	0.70841300	7.09059800	5.85823800

C	0.75580600	5.88045100	4.92753600	C	0.54729000	6.29467300	4.70224300
C	-0.28645800	4.96127800	5.12110300	C	-0.51108600	5.37328500	4.61719000
H	-0.41102100	4.15011700	4.39854800	H	-0.61419400	4.77632900	3.70687100
C	-1.13223700	5.04811800	6.22189600	C	-1.43344600	5.22910900	5.64672400
H	-1.93114000	4.31534200	6.36075900	H	-2.25516100	4.51480100	5.55301200
C	-0.93752300	6.07283500	7.14405200	C	-1.29504100	6.00931700	6.79337000
H	-1.58326900	6.15771500	8.02215100	H	-2.00452000	5.90978800	7.61935900
C	0.07058300	7.00878100	6.92788800	C	-0.23959600	6.91116300	6.88312000
H	0.17183000	7.84296500	7.62756800	H	-0.13858700	7.50472500	7.79771100
H	1.61775000	9.12279300	7.12851100	H	2.78507600	10.10579700	7.00770600
H	0.08169400	10.37930400	7.24184900	H	0.84695900	9.45107700	7.39767900
C	3.77024500	7.75655100	6.66785100	C	2.74094900	7.57493600	7.93688900
H	4.51422100	8.56449100	6.77027800	H	3.63156800	8.12245000	8.28914300
H	3.49402900	7.43120700	7.68375600	H	1.99002100	7.61985000	8.74138000
H	4.25961400	6.91127200	6.15789800	H	3.02378600	6.51839600	7.79905400
C	2.91750800	8.50053300	3.93591900	C	3.63318800	7.98752100	5.18821700
H	2.15316500	8.70347300	3.17501500	H	3.60647000	8.44113800	4.18820200
H	3.67298400	9.29715500	3.88057200	H	4.52203300	8.36996100	5.71773600
H	3.42180500	7.56432800	3.66026100	H	3.77067300	6.90321900	5.05135000
C	1.65557500	5.62874400	3.76960500	C	1.43758000	6.38766000	3.51541300
C	1.23534800	5.87818300	2.45882400	C	1.53562800	7.57404800	2.78084400
C	2.93305500	5.09406600	3.97606700	C	2.15001600	5.26747200	3.06822200
C	2.08390100	5.63204700	1.38082700	C	2.32519100	7.64451500	1.63581500
H	0.23529900	6.28585300	2.28887900	H	0.98066800	8.44786100	3.12266900
C	3.78023100	4.84083100	2.89967600	C	2.95213700	5.33821200	1.93103200
H	3.26023100	4.88465800	4.99718600	H	2.08368600	4.33540200	3.63522000
C	3.36057300	5.11643200	1.59833700	C	3.04193800	6.52742400	1.20903400
H	1.74617600	5.84593500	0.36363700	H	2.38148000	8.57889000	1.07089000
H	4.77592300	4.42704300	3.07783600	H	3.51231300	4.45759400	1.60678200
H	4.02661300	4.92408900	0.75377200	H	3.66659800	6.58236000	0.31425400

**D**	**TS$_{DE}$**
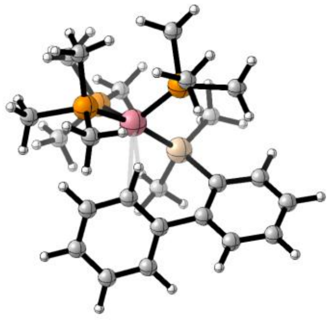	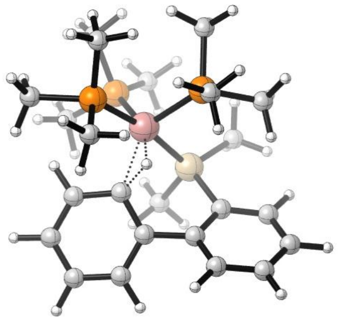
E(PBE0-D3) = -3596.809052<br>Thermal correction for Gibbs Energy = 0.479782<br>N_{imag} = 0	E(PBE0-D3) = -3596.800038<br>Thermal correction for Gibbs Energy = 0.479456<br>N_{imag} = 1 (-537.48 cm^{-1})

P	-1.86384500	0.09533800	-0.06401200	P	-1.83084700	0.04763600	0.01186800
P	1.77065000	-0.74800300	0.50135000	P	1.62380400	-0.76556800	0.60950300
Si	0.20966000	1.85852700	1.42151900	Si	0.17086500	1.98437500	1.44019500
C	-0.04078100	1.19584000	3.21063600	C	-0.08294700	1.31800400	3.22987600
H	-0.03548300	0.09732200	3.27384500	H	-0.23219300	0.23143000	3.28990400
H	-1.01171900	1.53765600	3.60000100	H	-0.97074600	1.79390000	3.67519600
H	0.72163700	1.57226400	3.90990600	H	0.76746400	1.56674500	3.88256400
C	-1.04197700	3.31192800	1.34935700	C	-1.11376000	3.40059600	1.39600100
H	-0.77781700	4.05632800	2.11909300	H	-0.90229400	4.10072000	2.22124600
H	-2.07616600	2.99094400	1.54918700	H	-2.14007200	3.02640300	1.54492200

H	-1.03581600	3.82077200	0.37481700	H	-1.09325900	3.96620100	0.45392000
C	2.27330300	3.57576800	0.31768500	C	2.27087200	3.46917800	0.22694700
C	1.88889700	2.81341500	1.44377600	C	1.87181900	2.85743100	1.43446200
C	2.75833700	2.83363400	2.54480200	C	2.76536500	2.90392000	2.51248800
C	3.96432200	3.53213200	2.53488600	C	4.02668100	3.48901300	2.40189800
C	4.34059000	4.24940200	1.40137800	C	4.42484400	4.04804600	1.18913300
C	3.48679000	4.27824100	0.30389600	C	3.54378100	4.04519400	0.11161400
H	2.49153800	2.28637900	3.45196300	H	2.47667500	2.47292000	3.47509200
H	4.61048900	3.51667500	3.41666500	H	4.69981200	3.50501400	3.26318500
H	5.28913200	4.79146100	1.37519500	H	5.41803900	4.49191400	1.08386500
H	3.76163300	4.84765300	-0.58823800	H	3.84752500	4.48763500	-0.84088300
C	1.34459900	3.72431900	-0.82351100	C	1.29490800	3.55796700	-0.87968800
C	0.74192200	2.61814000	-1.44790400	C	0.44793500	2.45874400	-1.20821300
C	-0.18860900	2.83468800	-2.46778700	C	-0.49546300	2.71367800	-2.23002700
C	-0.54988600	4.12103400	-2.85745700	C	-0.60951500	3.93526600	-2.88534200
C	0.05787600	5.21957500	-2.25162200	C	0.24277400	4.98653300	-2.55566000
C	1.00117100	5.01582500	-1.25044100	C	1.18128000	4.78385600	-1.55252500
H	1.20425000	1.60390700	-1.24303500	H	1.31925400	1.40920700	-0.99544200
H	-0.64813700	1.98516300	-2.96464000	H	-1.20448000	1.93718900	-2.49995400
H	-1.29948200	4.26361700	-3.63950300	H	-1.38281300	4.06644300	-3.64768500
H	-0.21020800	6.23540500	-2.55174000	H	0.16444800	5.95439800	-3.05604200
H	1.46032800	5.87192000	-0.75043100	H	1.83213400	5.60686300	-1.24730300
C	2.74295800	-0.35971000	2.02139600	C	2.43898600	-0.48162200	2.23898000
H	3.41587600	0.48447700	1.82431800	H	2.98538800	0.47000900	2.21675500
H	2.08454100	-0.08593800	2.85357700	H	1.70214400	-0.43867900	3.04841600
H	3.34024100	-1.23769900	2.31303000	H	3.14652100	-1.30012400	2.44310000
C	3.25295400	-1.31429700	-0.46611200	C	3.19888300	-1.08956000	-0.30644600
H	3.86852200	-0.42675700	-0.68004600	H	3.74983000	-0.13742800	-0.34605500
H	3.85945600	-2.02822900	0.11349700	H	3.81960000	-1.84017400	0.20797100
H	2.98855500	-1.77834500	-1.42383600	H	3.02735900	-1.42019200	-1.33769500
C	1.07633200	-2.36575100	1.07419200	C	1.03121600	-2.48466400	0.95892900
H	0.37017200	-2.15083600	1.89023900	H	0.24635400	-2.41747800	1.72821800
H	0.51573400	-2.85835500	0.27013700	H	0.58805400	-2.94460000	0.06720000
H	1.85624100	-3.04905500	1.44847500	H	1.83771800	-3.13300700	1.33744400
C	-2.64749500	0.12036800	1.60945300	C	-2.55936400	-0.03892100	1.70416500
H	-2.12000000	-0.58215000	2.26959000	H	-1.98293800	-0.74205000	2.32105300
H	-2.59246000	1.11733700	2.06019300	H	-2.53138800	0.94279800	2.19211300
H	-3.70430700	-0.17989800	1.53817200	H	-3.60372700	-0.38251700	1.65108000
C	-3.08065700	1.15331200	-0.96677600	C	-3.12540700	1.08131300	-0.80001300
H	-4.12454800	0.88684400	-0.73597600	H	-4.13656700	0.78813100	-0.47801300
H	-2.89890200	2.20167000	-0.68980000	H	-2.94863400	2.13642800	-0.54827400
H	-2.91705200	1.06541500	-2.04978300	H	-3.06075200	0.98342600	-1.89255100
C	-2.49859300	-1.59314400	-0.50370000	C	-2.38304500	-1.63607000	-0.52721800
H	-1.97970700	-2.33260700	0.12413000	H	-1.80381600	-2.39415500	0.01934800
H	-3.58115400	-1.67015900	-0.31616500	H	-3.45296800	-1.78889000	-0.31648900
H	-2.31053500	-1.84765700	-1.55313700	H	-2.21613600	-1.78734500	-1.60107400
Co	0.20865700	0.42937100	-0.38629400	Co	0.23466200	0.59526700	-0.37433500
P	0.28413800	-0.62004200	-2.33551800	P	0.34346600	-0.42067600	-2.37407200
C	-1.06246500	-0.50193100	-3.61326600	C	-0.99319600	-0.30293500	-3.65862100
H	-1.22293900	0.53554600	-3.93346100	H	-1.06426400	0.72471400	-4.03592700
H	-0.78625700	-1.09082200	-4.50135300	H	-0.75499200	-0.96416600	-4.50566300
H	-2.01584400	-0.88400900	-3.22637800	H	-1.97289000	-0.59257100	-3.25507800
C	1.68247300	-0.08136900	-3.41293000	C	1.73061100	0.22701700	-3.39458100
H	1.72595600	-0.66956900	-4.34251500	H	1.77177500	-0.26267500	-4.37948900
H	1.54911500	0.98010300	-3.66623900	H	1.58180700	1.30833200	-3.52886200
H	2.63317900	-0.17686100	-2.87387400	H	2.68437900	0.08137200	-2.87086900
C	0.46997100	-2.45779200	-2.41570600	C	0.62171200	-2.24194400	-2.52509400

H -0.42129400 -2.93514700 -1.98542300<br>H 0.57742800 -2.78776300 -3.46032100<br>H 1.34189900 -2.79962900 -1.84596600	H -0.25539400 -2.78402100 -2.14531000<br>H 0.77395300 -2.51707800 -3.57989000<br>H 1.49635700 -2.56652400 -1.94920600
**E**<br>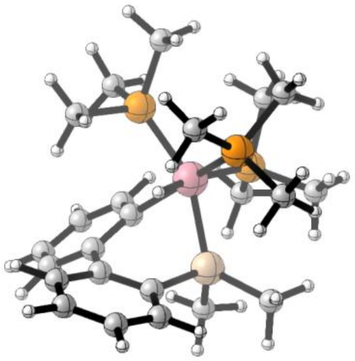<br>E(PBE0-D3) = -3596.821561<br>Thermal correction for Gibbs Energy = 0.481404<br>$N_{imag} = 0$	**F**<br>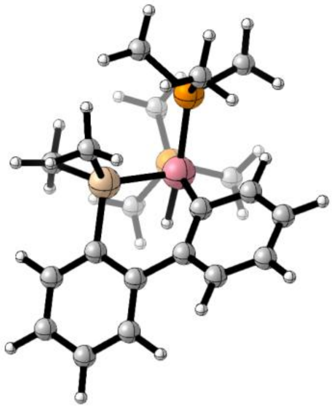<br>E(PBE0-D3) = -3135.898392<br>Thermal correction for Gibbs Energy = 0.372407<br>$N_{imag} = 0$
P -1.81607900 -0.02314100 0.05045800<br>P 1.28727300 -1.00878100 0.78999900<br>Si 0.22986600 2.15569100 1.42689800<br>C 0.17636300 1.77980800 3.31138200<br>H -0.65396100 1.11770300 3.60013800<br>H 0.00321500 2.74680100 3.81302800<br>H 1.09494000 1.35722300 3.73592400<br>C -1.14659800 3.48358200 1.40193700<br>H -0.81623100 4.33605100 2.01816500<br>H -2.06587100 3.09181300 1.87048100<br>H -1.39518800 3.86127600 0.40162400<br>C 2.20012900 3.42215000 -0.15091000<br>C 1.91702200 2.99719700 1.16592800<br>C 2.93346400 3.06804100 2.12193700<br>C 4.21460600 3.51995600 1.79063900<br>C 4.49005500 3.91510100 0.48463000<br>C 3.48106300 3.87506900 -0.47909200<br>H 2.73122400 2.76390300 3.15328000<br>H 4.99655200 3.56050800 2.55381900<br>H 5.49158700 4.25793000 0.21197800<br>H 3.69793200 4.18562700 -1.50507000<br>C 1.08384600 3.43048000 -1.12802000<br>C 0.11290000 2.38650300 -1.17104500<br>C -0.98612500 2.63733000 -2.01381800<br>C -1.13144700 3.78371900 -2.79403000<br>C -0.14462200 4.76428200 -2.76625600<br>C 0.94768500 4.57829800 -1.92701300<br>H 1.67156200 0.99817400 -0.21703300<br>H -1.76919700 1.88483300 -2.09098600<br>H -2.01646100 3.90344100 -3.42585200<br>H -0.23456200 5.67267800 -3.36678100<br>H 1.70217100 5.36504200 -1.84563200<br>C 1.63043400 -0.95044700 2.59327200	P -1.78589800 0.16865700 -0.21785700<br>P 1.26906200 -1.30010400 -0.05747700<br>Si 0.44218300 1.67274200 1.63720300<br>C 0.77263500 0.50366500 3.10451400<br>H 1.80041700 0.11205400 3.08953800<br>H 0.08083700 -0.35353000 3.09668100<br>H 0.62985400 1.03383800 4.06121600<br>C -1.07902300 2.68681100 2.16878800<br>H -0.81093900 3.23202600 3.08901800<br>H -1.97491300 2.08776900 2.39376300<br>H -1.33513400 3.43214500 1.40106400<br>C 1.96154400 3.75911400 0.44109400<br>C 1.83166300 2.93954300 1.58448500<br>C 2.74422300 3.08294900 2.63703600<br>C 3.78261600 4.00998600 2.58411700<br>C 3.92357000 4.80471000 1.44833200<br>C 3.02678200 4.67543800 0.39308700<br>H 2.64282000 2.45441200 3.52675100<br>H 4.48318300 4.10483500 3.41782200<br>H 4.74557400 5.52152400 1.37511400<br>H 3.18148100 5.28012800 -0.50246500<br>C 1.03394200 3.62971800 -0.71228500<br>C 0.32150900 2.42873200 -0.98160500<br>C -0.38489600 2.37015100 -2.20044900<br>C -0.43780800 3.42488000 -3.10956400<br>C 0.18176000 4.62802200 -2.78181000<br>C 0.90225000 4.71641300 -1.59672700<br>H 1.80417100 0.96693300 -0.18664600<br>H -0.91876600 1.44964600 -2.47139900<br>H -0.98854000 3.31715900 -4.04825600<br>H 0.10445600 5.49465800 -3.44278300<br>H 1.38018000 5.66607000 -1.34702900<br>C 2.88955700 -1.41918300 0.78841200

H	2.24258500	-0.06548300	2.80914200	H	3.53908400	-0.61121100	0.42554100
H	0.70189400	-0.87811500	3.17281000	H	2.75114000	-1.28386700	1.86960700
H	2.18182600	-1.85360500	2.89661500	H	3.36300200	-2.39635700	0.60678900
C	3.03299900	-1.23529000	0.24666100	C	1.71461800	-1.79174200	-1.77523800
H	3.57438800	-0.30814700	0.48600700	H	2.33334800	-1.00017700	-2.22242700
H	3.50606200	-2.08193100	0.76741800	H	2.27074500	-2.74194300	-1.79553100
H	3.10763900	-1.39487000	-0.83579300	H	0.80301700	-1.89717000	-2.38195700
C	0.66500500	-2.75114700	0.71506200	C	0.48105700	-2.84882500	0.56127200
H	-0.28000100	-2.82706100	1.27260600	H	0.26125300	-2.73931800	1.63385400
H	0.47521600	-3.05901500	-0.32015500	H	-0.46412800	-3.04162600	0.03387300
H	1.38865600	-3.44677300	1.16720500	H	1.14388700	-3.71777800	0.42597500
C	-2.23400800	-0.49856300	1.77993700	C	-2.40433700	-0.71084200	1.26995100
H	-1.47124600	-1.17042000	2.19596400	H	-1.83350100	-1.63595200	1.42128600
H	-2.25276200	0.40936700	2.39804300	H	-2.26247300	-0.07517300	2.15449400
H	-3.21799500	-0.98917100	1.83435200	H	-3.47269100	-0.95635100	1.17061100
C	-3.26709600	1.06484500	-0.28577600	C	-3.06139400	1.47195600	-0.41846900
H	-4.15729700	0.66978200	0.22699900	H	-4.06795700	1.03171100	-0.48389000
H	-3.06260200	2.08511300	0.06358300	H	-3.01667900	2.15902600	0.43548700
H	-3.47887400	1.10643600	-1.36273200	H	-2.85127000	2.05577700	-1.32474000
C	-2.39167200	-1.53746200	-0.83791500	C	-2.29720100	-1.00100900	-1.55094300
H	-1.73825100	-2.38753300	-0.60095000	H	-1.70656800	-1.92629400	-1.48952600
H	-3.42685200	-1.79706100	-0.56647900	H	-3.36632400	-1.25518400	-1.48070800
H	-2.34603100	-1.36361200	-1.92261500	H	-2.10902300	-0.54382800	-2.53396000
Co	0.27708500	0.61665400	-0.26838000	Co	0.36909900	0.67083800	-0.27971000
P	0.73720300	-0.14589900	-2.31139500				
C	-0.38424400	0.17818900	-3.73960300				
H	-0.43511700	1.25511800	-3.93775200				
H	-0.00190500	-0.33866500	-4.63277200				
H	-1.40134400	-0.18273000	-3.52968800				
C	2.28276000	0.59362200	-2.96522300				
H	2.49617100	0.25581900	-3.99088900				
H	2.16273400	1.68648700	-2.94810100				
H	3.12569500	0.34286700	-2.30728400				
C	1.01984400	-1.94037700	-2.67298300				
H	0.07697900	-2.49001700	-2.53461900				
H	1.34983600	-2.07415500	-3.71467700				
H	1.77516100	-2.38304400	-2.01194100				

TS_{FG}

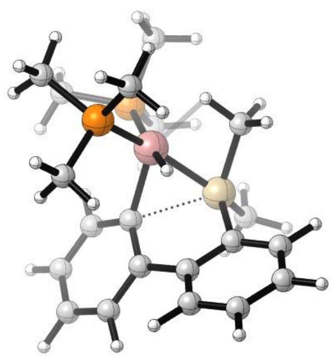

E(PBE0-D3) = -3135.875991
Thermal correction for Gibbs Energy = 0.374468
N_{imag} = 1 (-53.39 cm^{-1})

G

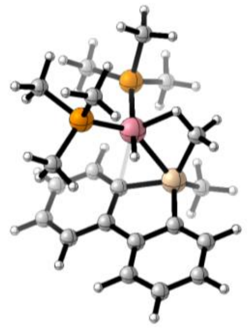

E(PBE0-D3) = -3135.885558
Thermal correction for Gibbs Energy = 0.371785
N_{imag} = 0

P	-1.67558100	-0.82285800	0.24238500	P	-1.70883700	-0.62787600	0.11062200
P	0.87853100	1.19452100	1.40309900	P	0.84004200	1.04149000	1.49190900
Si	1.32648100	-1.94150100	-1.21096200	Si	1.44921400	-2.25261400	-1.05526900
C	0.69096100	-0.42823100	-2.18662600	C	0.66437300	-0.83669500	-2.08215000
H	0.27477900	0.36703900	-1.47801300	H	0.26624900	0.12229100	-1.55974600
H	-0.12258100	-0.67056900	-2.88619200	H	-0.14424600	-1.22023000	-2.72060200
H	1.49912000	0.09775900	-2.71482600	H	1.47360200	-0.44063300	-2.71112900
C	0.70231300	-3.58650400	-1.87311800	C	0.77979300	-3.86788400	-1.75180200
H	1.25358400	-3.85763000	-2.78769800	H	0.98531000	-3.95279100	-2.83054200
H	-0.37140000	-3.57649700	-2.10655500	H	-0.31018100	-3.93380000	-1.61057300
H	0.88587900	-4.37372500	-1.12561000	H	1.24403100	-4.72542500	-1.24187700
C	3.64417800	-2.04955600	0.27828200	C	3.81996800	-2.15570600	0.24271800
C	3.19610100	-1.94374600	-1.05791200	C	3.32352300	-2.14813900	-1.08196900
C	4.11719500	-1.70866900	-2.07923300	C	4.20905600	-1.99862900	-2.14776200
C	5.47611500	-1.55375500	-1.79666800	C	5.57819600	-1.85157700	-1.91693400
C	5.91345300	-1.62742200	-0.47584200	C	6.06276900	-1.84392200	-0.60822500
C	5.00610800	-1.87603200	0.55361900	C	5.19128000	-1.99370700	0.46878600
H	3.77457000	-1.63392800	-3.11641600	H	3.83440400	-1.99171600	-3.17638400
H	6.18940900	-1.36626400	-2.60323700	H	6.26894500	-1.73813300	-2.75625900
H	6.97194200	-1.48746800	-0.24145800	H	7.13286100	-1.71835400	-0.42444600
H	5.36412400	-1.91979900	1.58525800	H	5.58736500	-1.97709800	1.48709900
C	2.64306500	-2.40057000	1.31686800	C	2.80238100	-2.34865500	1.29545100
C	1.26310200	-2.04890500	1.16281000	C	1.44609800	-2.33491000	0.85348800
C	0.38536400	-2.66465000	2.08440800	C	0.44536300	-2.65953500	1.78291900
C	0.81183000	-3.43722200	3.16007700	C	0.74762200	-2.86209700	3.13034000
C	2.17137000	-3.68779500	3.33859500	C	2.07558500	-2.82573000	3.55740000
C	3.06487100	-3.18935600	2.39972500	C	3.09894400	-2.59905000	2.63653100
H	1.92663500	-0.14485900	0.07009200	H	1.87465700	0.04773600	-0.12024400
H	-0.68978200	-2.51230800	1.98297400	H	-0.58253300	-2.79937500	1.44532400
H	0.07609900	-3.85074900	3.85585500	H	-0.05123800	-3.08394600	3.84294200
H	2.52599000	-4.30055100	4.17058200	H	2.31934400	-3.01188700	4.60629700
H	4.12235200	-3.45260200	2.47988500	H	4.13858400	-2.63476300	2.97072500
C	-2.57057200	0.36728100	-0.83900900	C	-2.56085200	0.84757300	-0.58748100
H	-2.21092200	0.26782400	-1.87306800	H	-2.19611900	1.01772400	-1.61122600
H	-3.65880400	0.20136300	-0.81902500	H	-3.65615200	0.73069200	-0.60634200
H	-2.35361100	1.39176800	-0.50284200	H	-2.30117300	1.73302200	0.01116600
C	-2.61820500	-0.58973500	1.80657200	C	-2.64905900	-0.83860900	1.67961300
H	-2.51550000	0.45049900	2.14061700	H	-2.45274500	0.00584800	2.35116200
H	-3.68594200	-0.81360600	1.65937900	H	-3.73082200	-0.89816300	1.48420600
H	-2.21967600	-1.24007700	2.59758300	H	-2.32756300	-1.75607500	2.19188500
C	-2.38952800	-2.41146900	-0.35212100	C	-2.49627800	-1.96481200	-0.88737200
H	-3.48213200	-2.44793600	-0.22145900	H	-3.59419700	-1.88094200	-0.89319300
H	-2.15426500	-2.52645800	-1.41955700	H	-2.12977200	-1.92221200	-1.92284900
H	-1.92713700	-3.25187900	0.18547400	H	-2.21759800	-2.94436200	-0.47072900
C	2.13749900	0.82913600	2.68564400	C	2.35220700	0.80691600	2.50355100
H	1.76580100	0.01737300	3.32832900	H	2.22645400	-0.08615500	3.13133400
H	3.04816300	0.46300800	2.19059600	H	3.20684900	0.62919800	1.83609000
H	2.37131500	1.71372300	3.29811400	H	2.54893100	1.68188500	3.14274700
C	1.63219300	2.62589900	0.52648600	C	1.14714900	2.70512600	0.76451500
H	2.51467500	2.27645500	-0.02821500	H	1.96971700	2.62481700	0.04022800
H	0.91142800	3.03047000	-0.20014800	H	0.24944400	3.03638500	0.22176300
H	1.92882200	3.42744600	1.22102500	H	1.40240800	3.45228000	1.53338500
C	-0.37055600	2.08889600	2.43182300	C	-0.33018800	1.52156000	2.84348000
H	-1.18931600	2.46107100	1.79734900	H	-1.27596800	1.89152900	2.41987200
H	-0.79266400	1.39955900	3.17731700	H	-0.54513300	0.64131400	3.46731500
H	0.08160000	2.94293400	2.96081100	H	0.09752000	2.31153100	3.48093700
Co	0.51688700	-0.52476800	0.17779800	Co	0.46614900	-0.42833600	0.02292400

4. ^{1}H, ^{13}C and ^{19}F NMR Spectra

2-(Diethyl(1-phenylethoxy)silane) 2a.

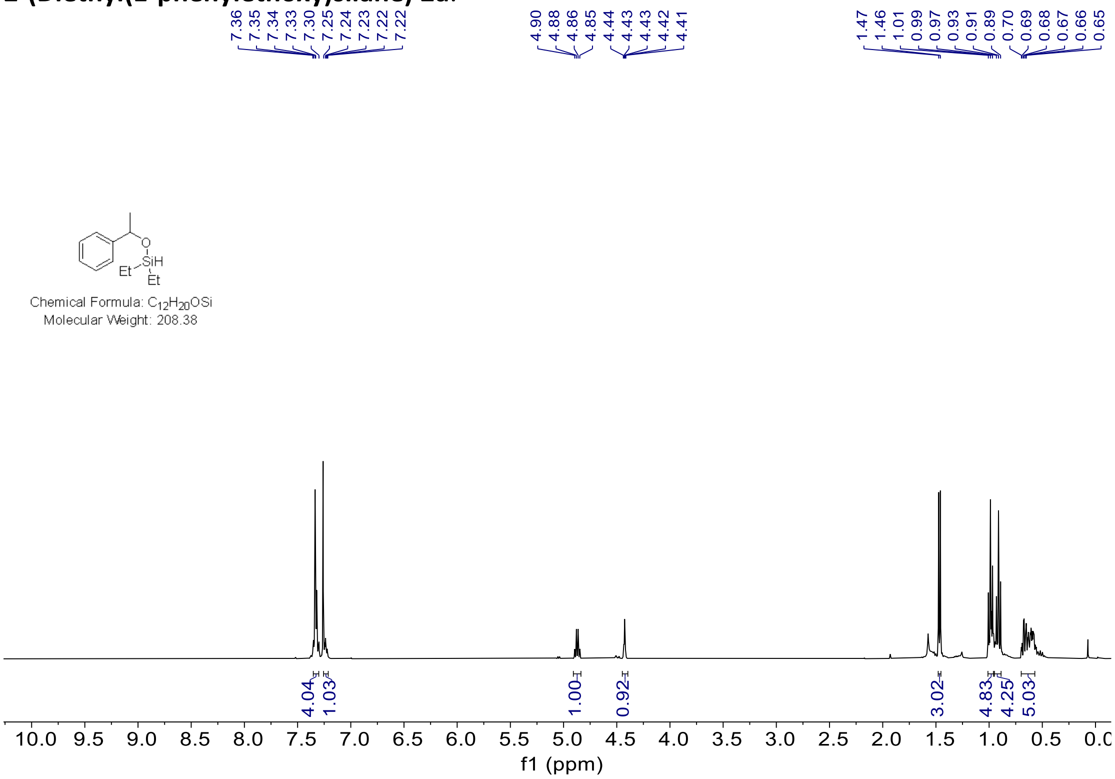

3-(diethylbis(1-phenylethoxy)silane) 3a.

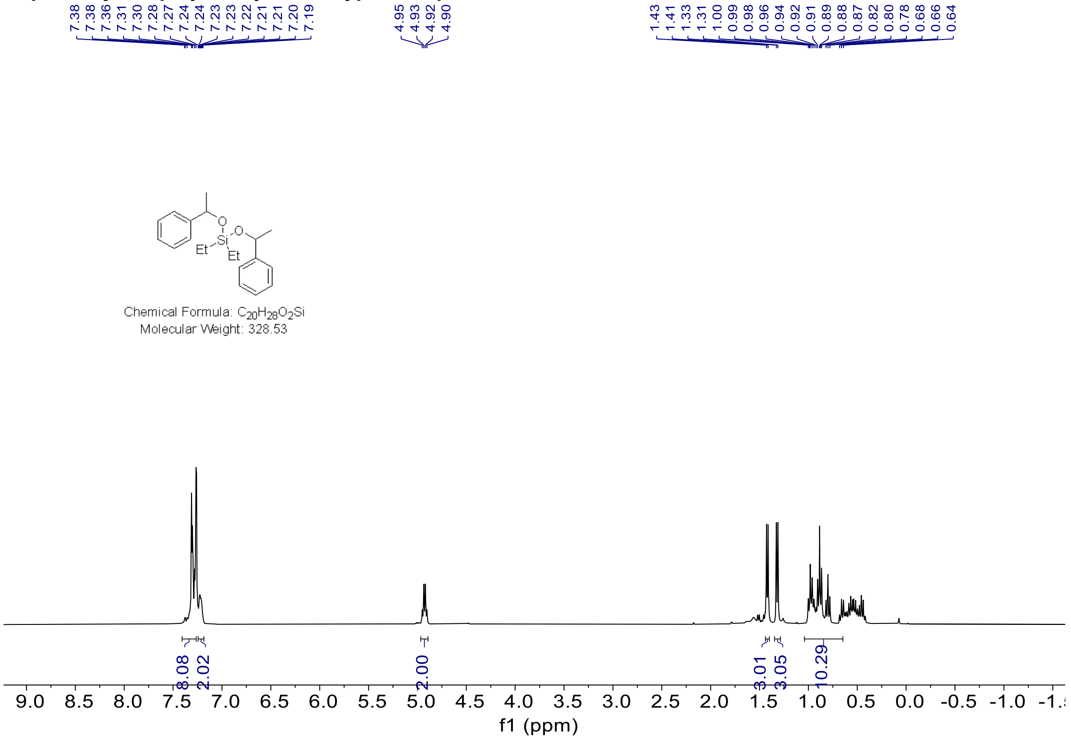

1,1-Diethyl-3-methyl-1,3-dihydrobenzo[c][1,2]oxasilole 4a.

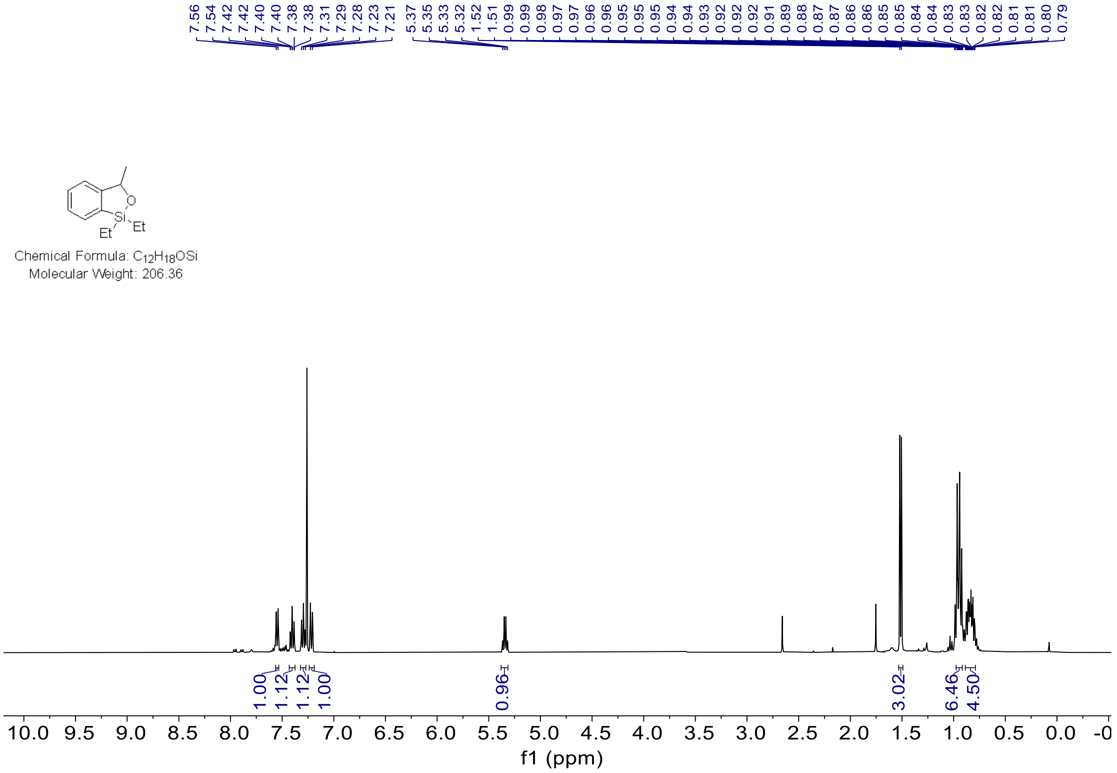

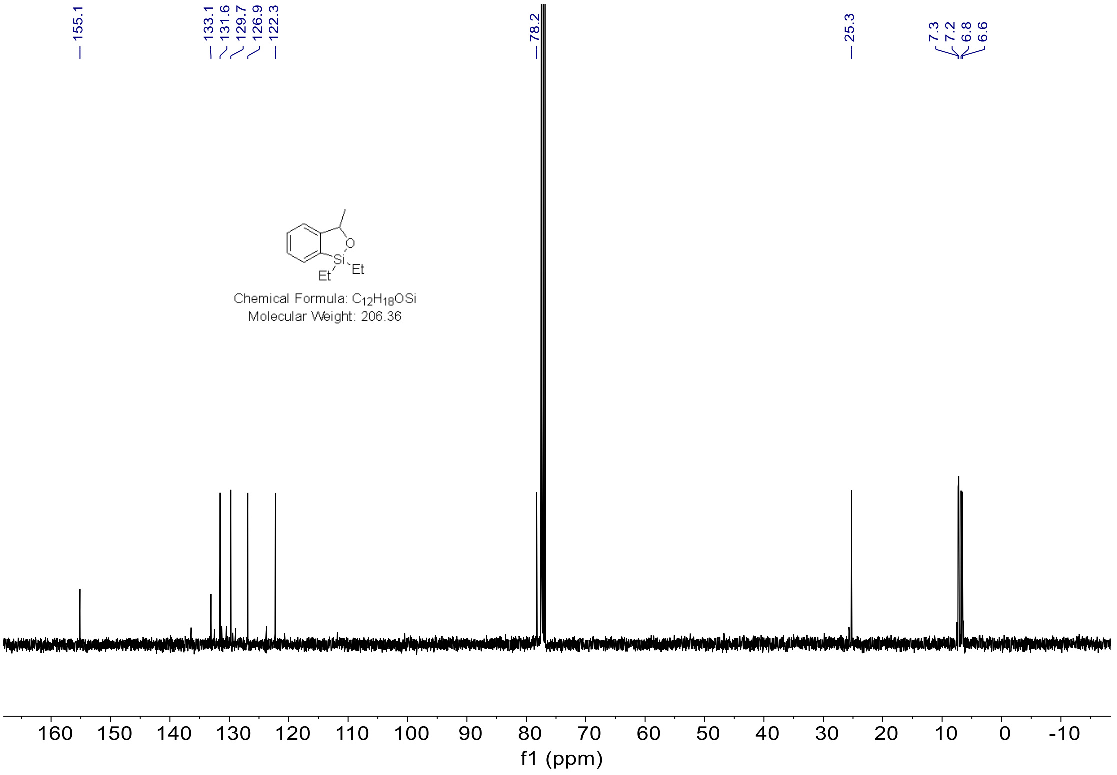

1,1-diethyl-3,6-dimethyl-1,3-dihydrobenzo[c][1,2]oxasilole 4b.

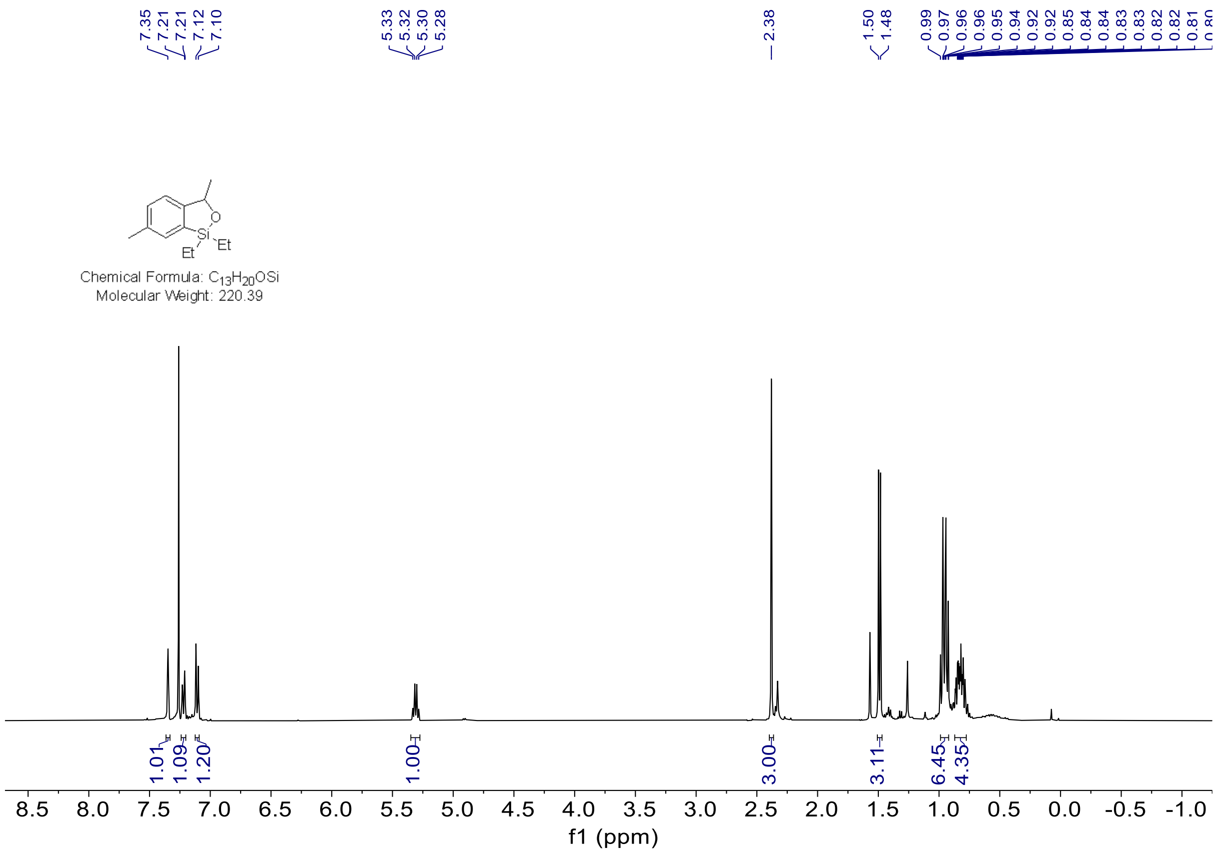

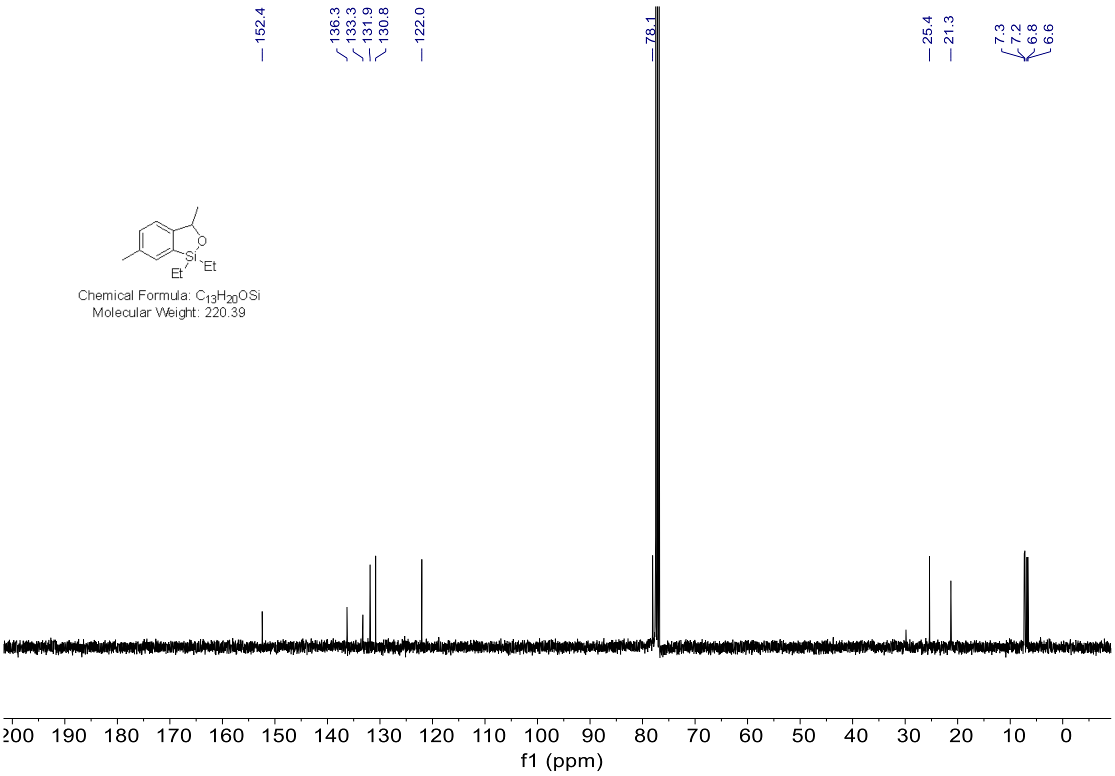

1,1-diethyl-3,4-dimethyl-1,3-dihydrobenzo[c][1,2]oxasilole 4c.

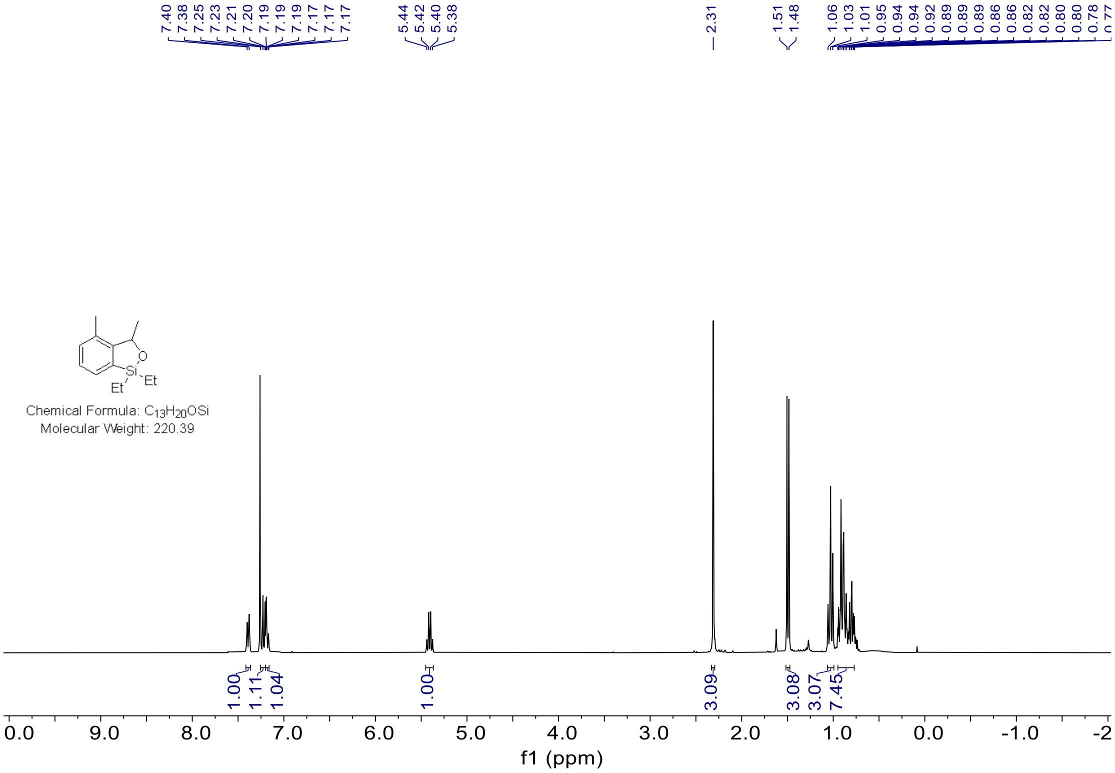

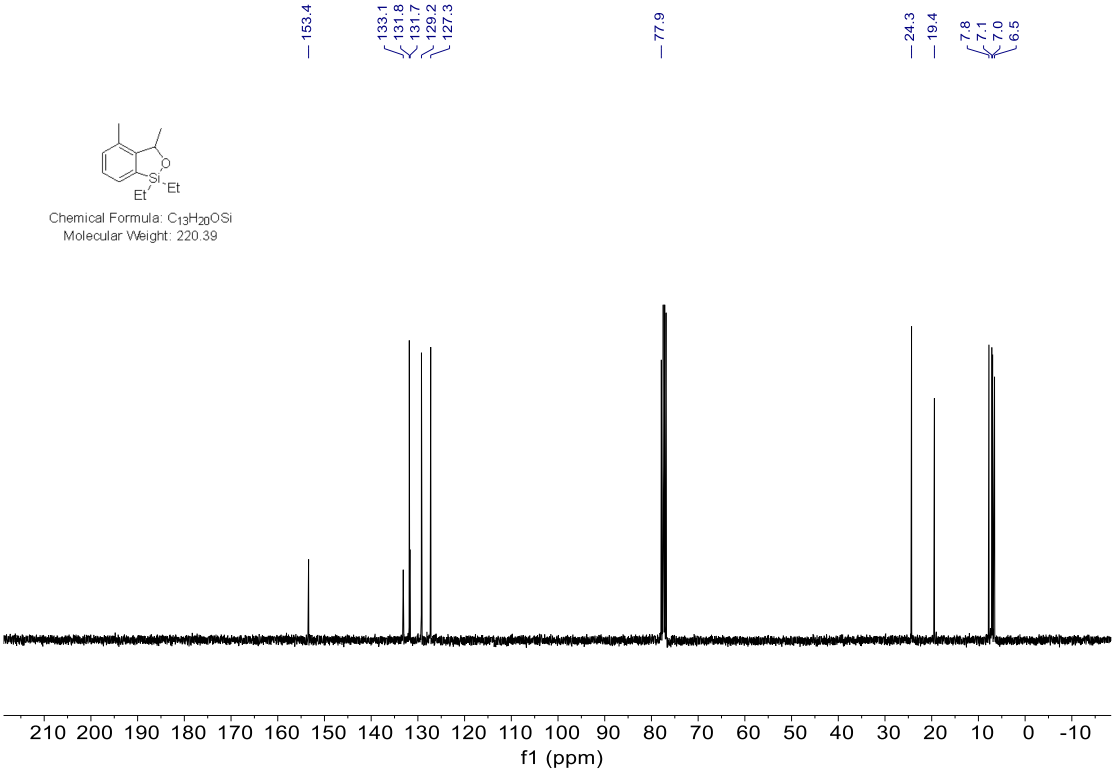

1,1-Diethyl-3,5-dimethyl-1,3-dihydrobenzo[c][1,2]oxasilole 4d.

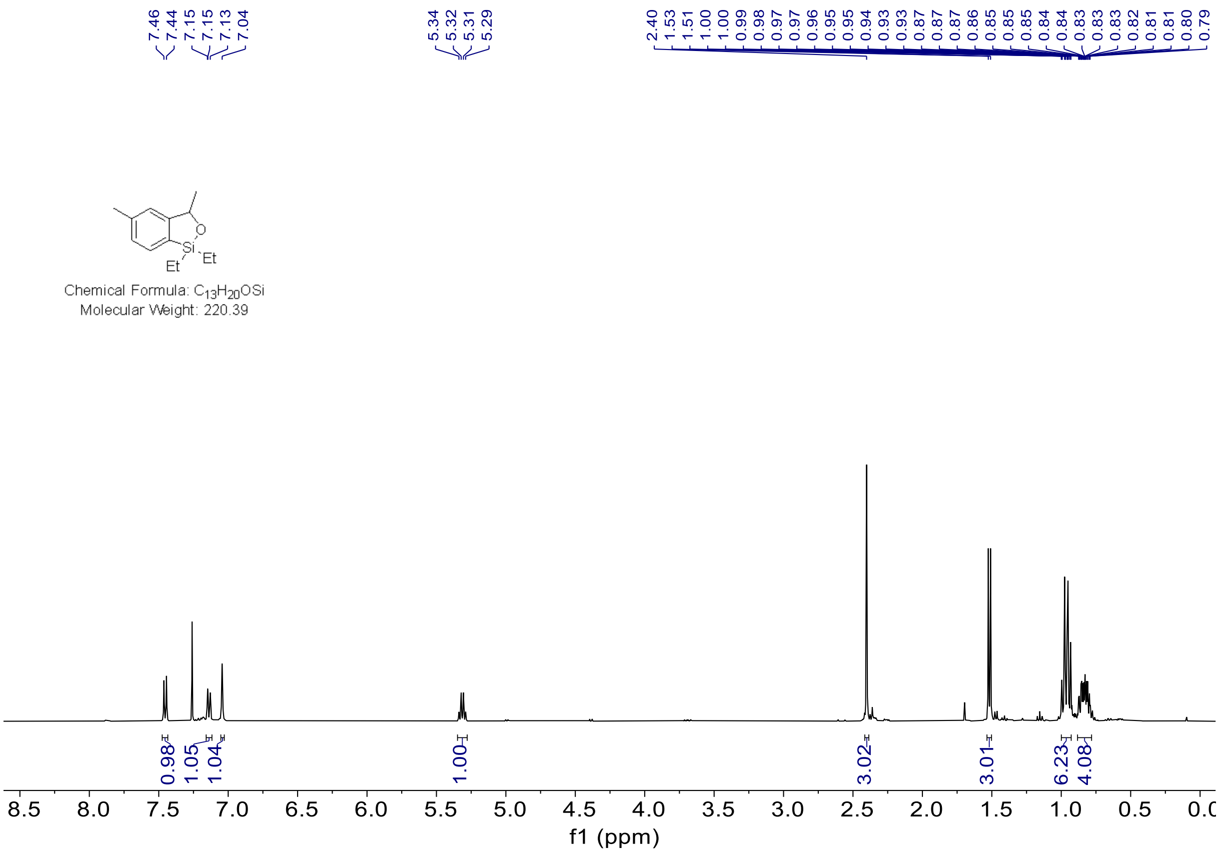

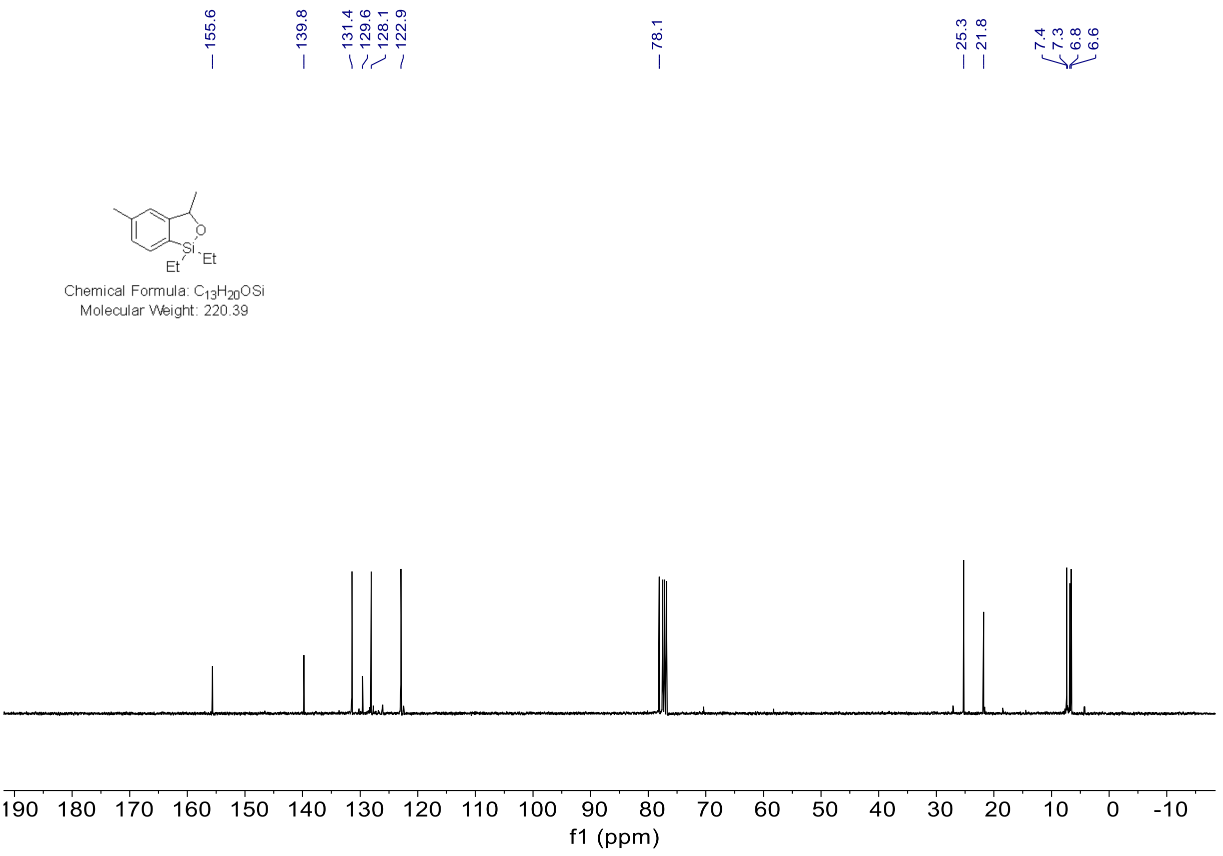

6-(Tert-butyl)-1,1-diethyl-3-methyl-1,3-dihydrobenzo[c][1,2]oxasilole 4e.

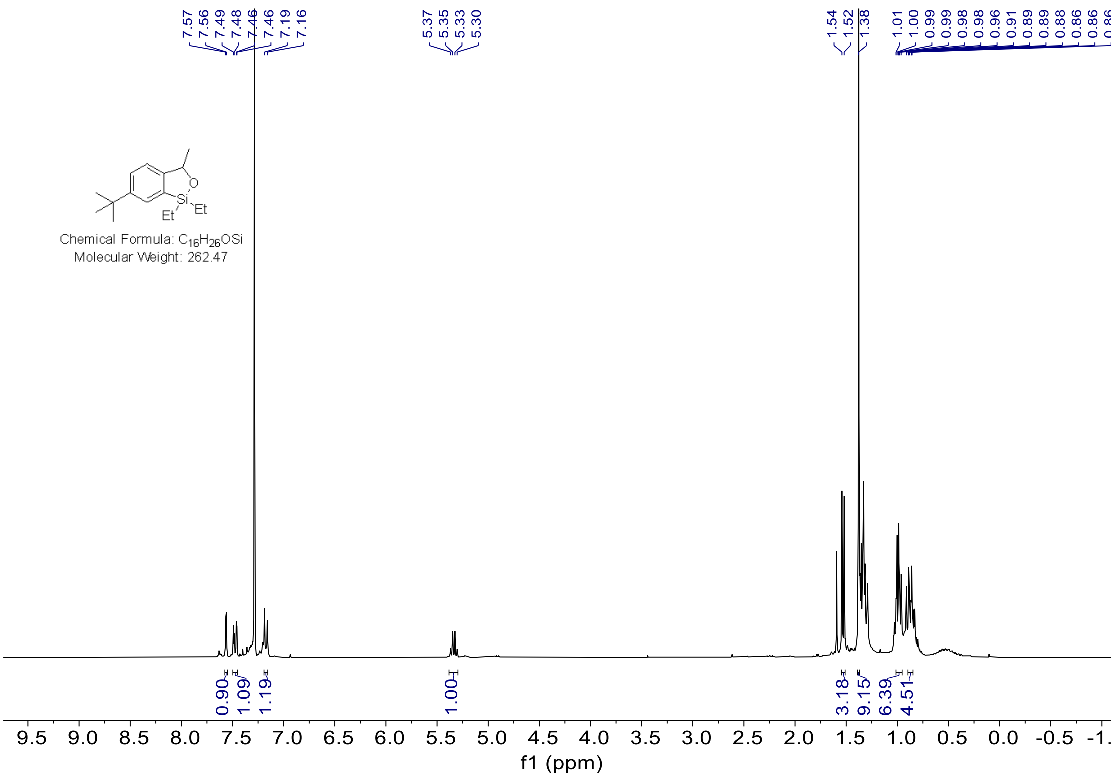

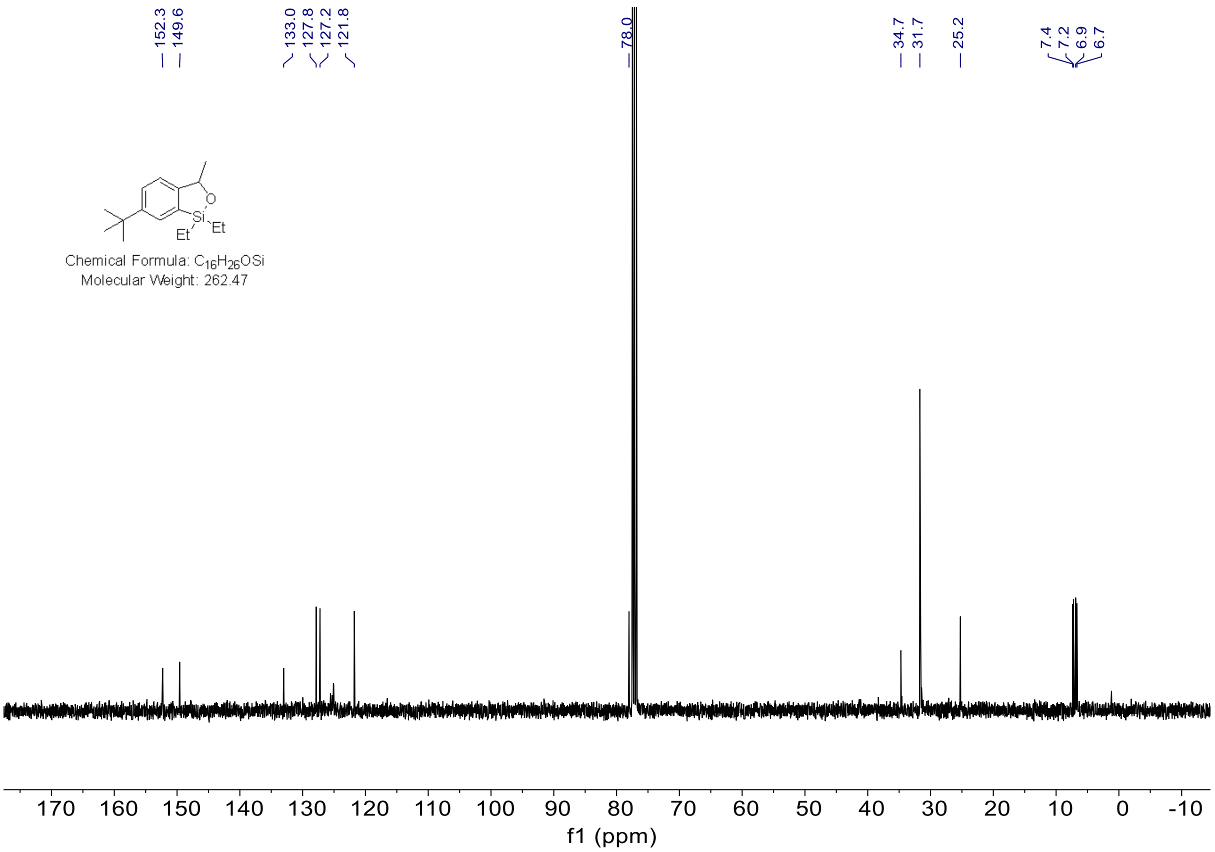

1,1-Diethyl-3-methyl-6-phenyl-1,3-dihydrobenzo[c][1,2]oxasilole 4f.

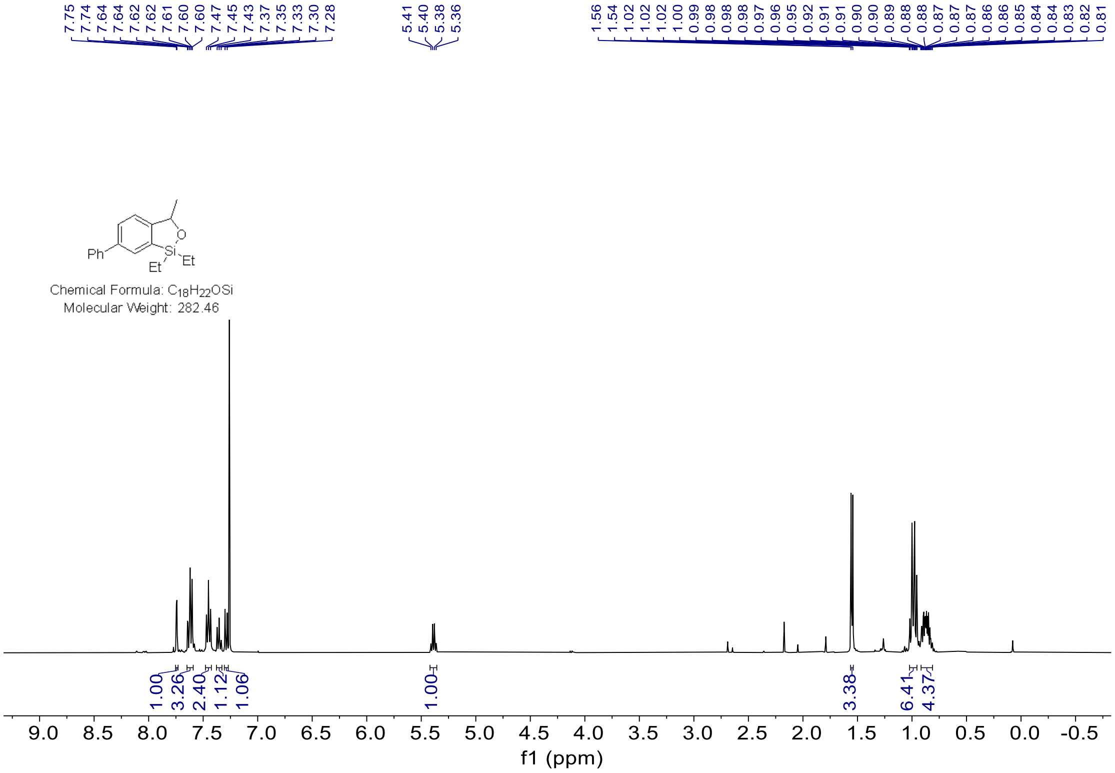

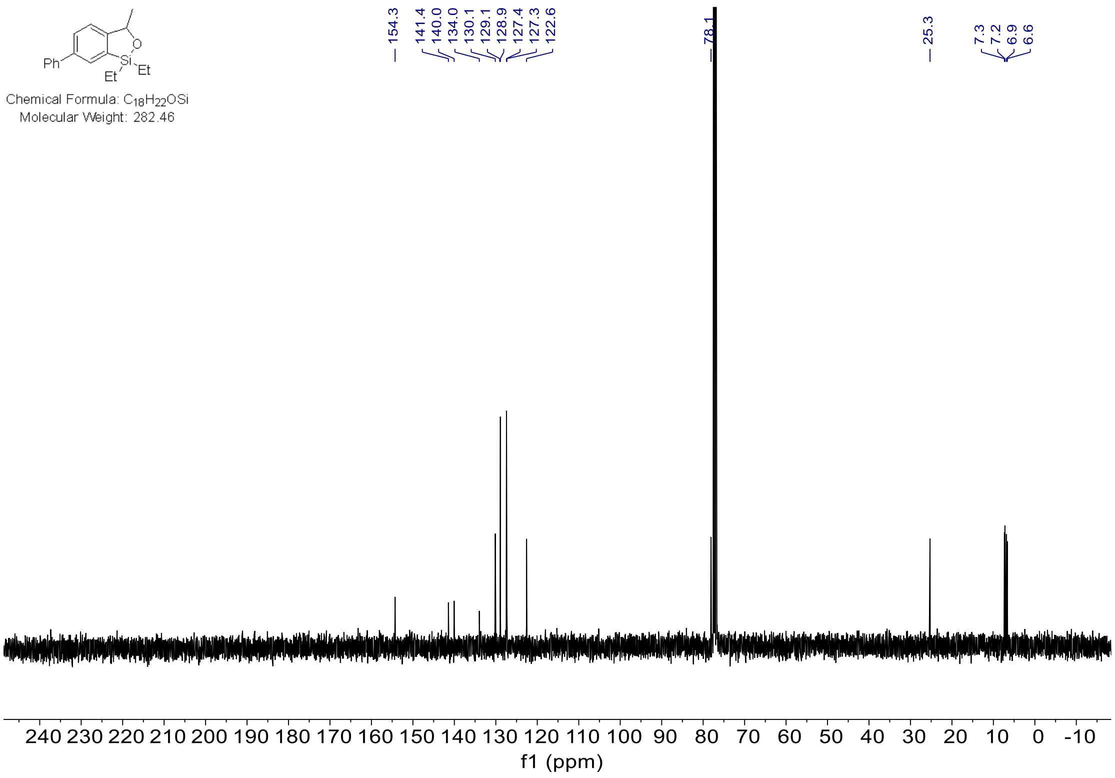

1,1-Diethyl-6-fluoro-3-methyl-1,3-dihydrobenzo[c][1,2]oxasilole 4g.

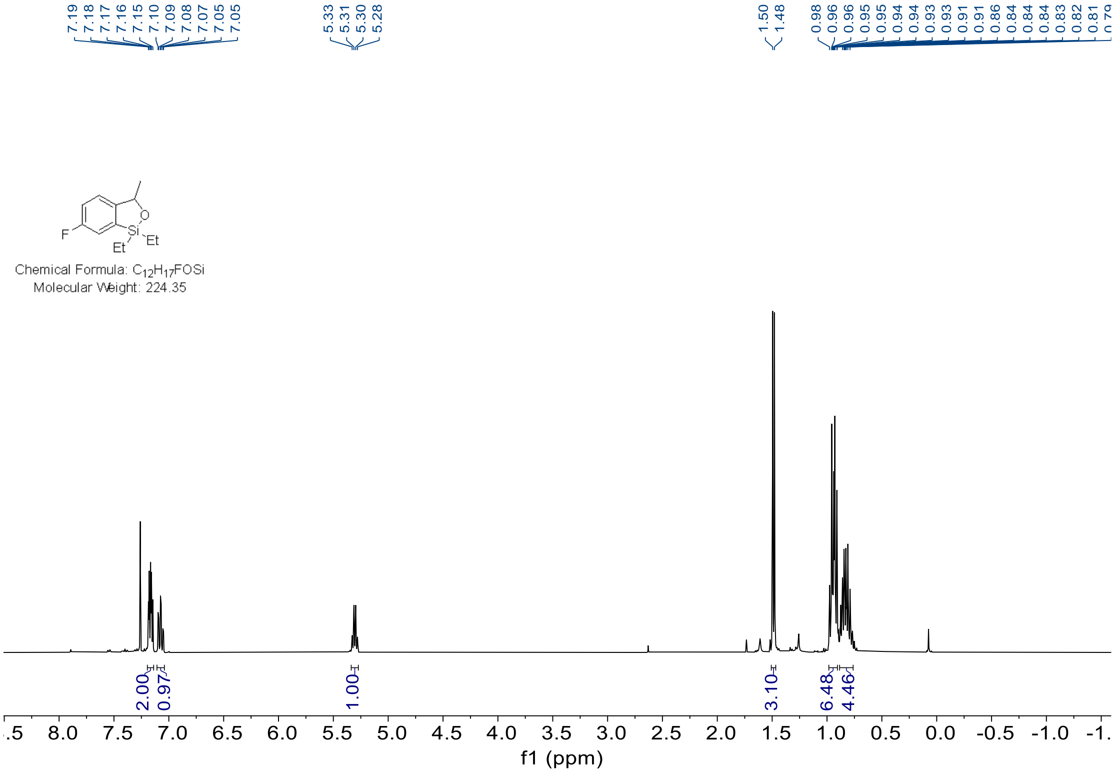

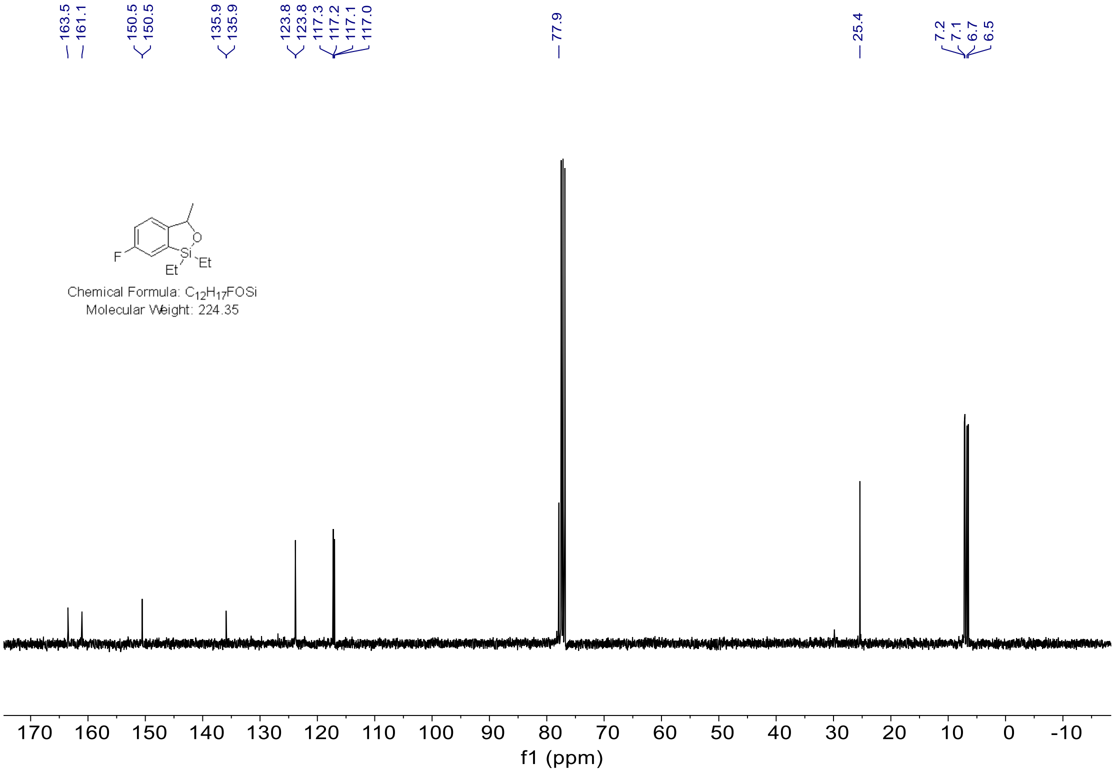

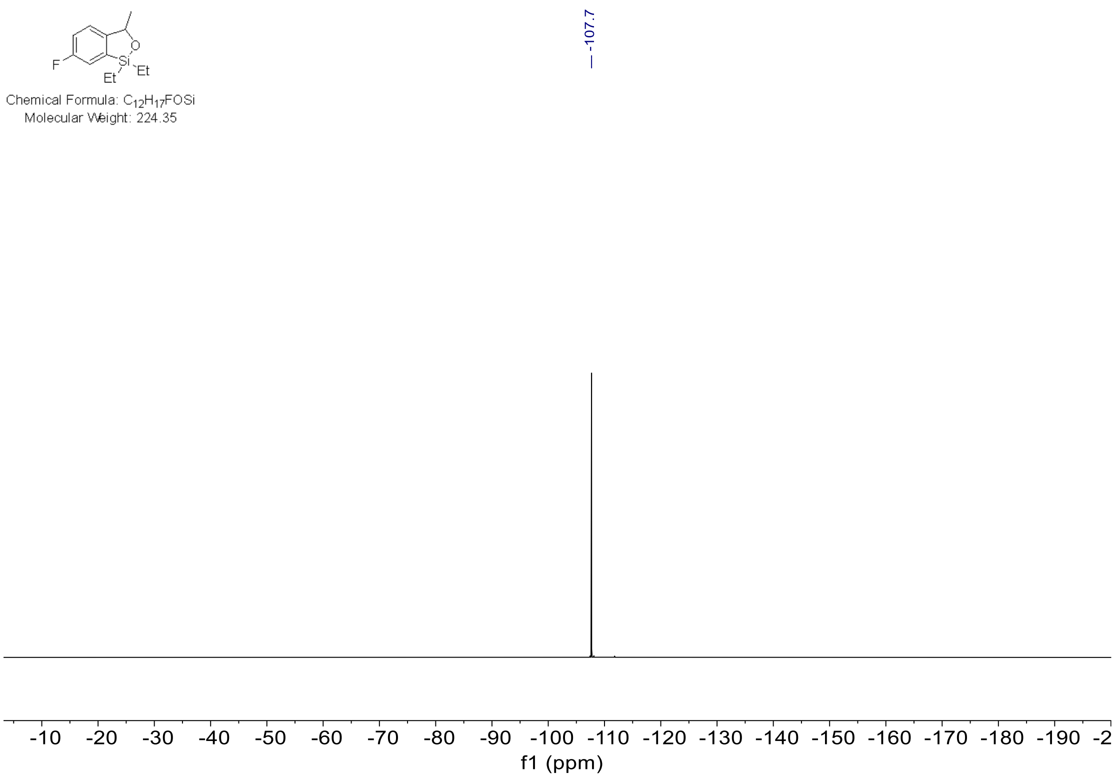

1,1-Diethyl-3-methyl-6-(trifluoromethyl)-1,3-dihydrobenzo[c][1,2]oxasilole 4h.

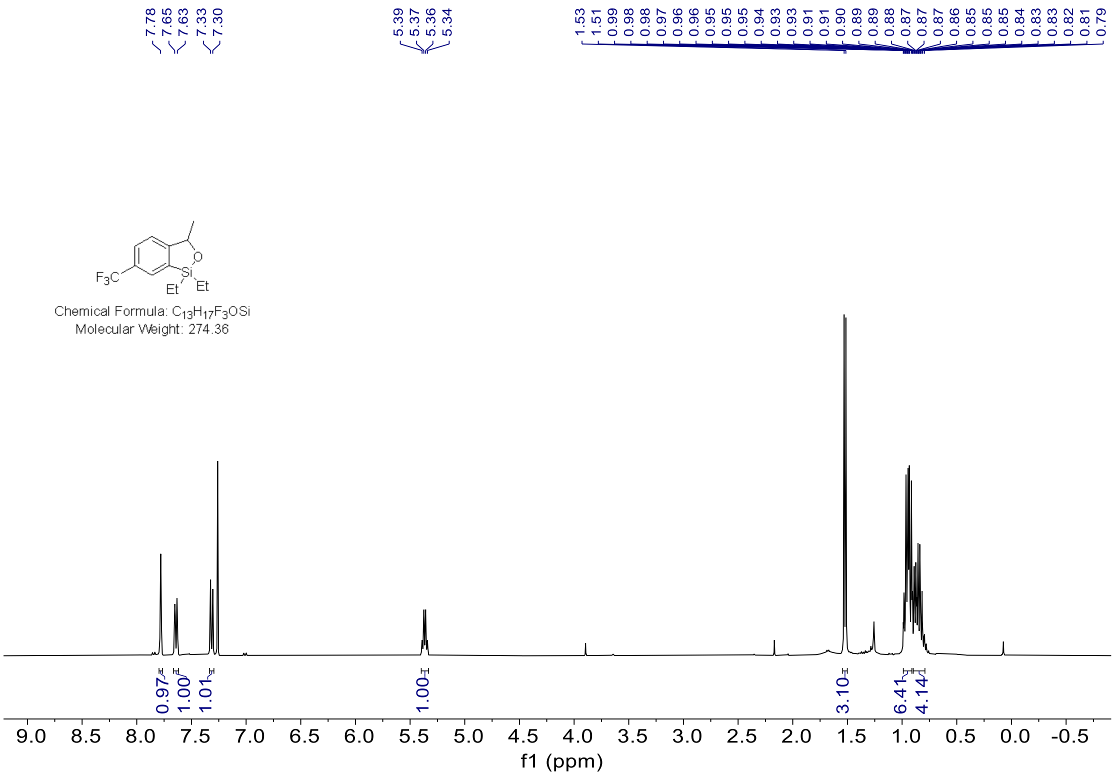

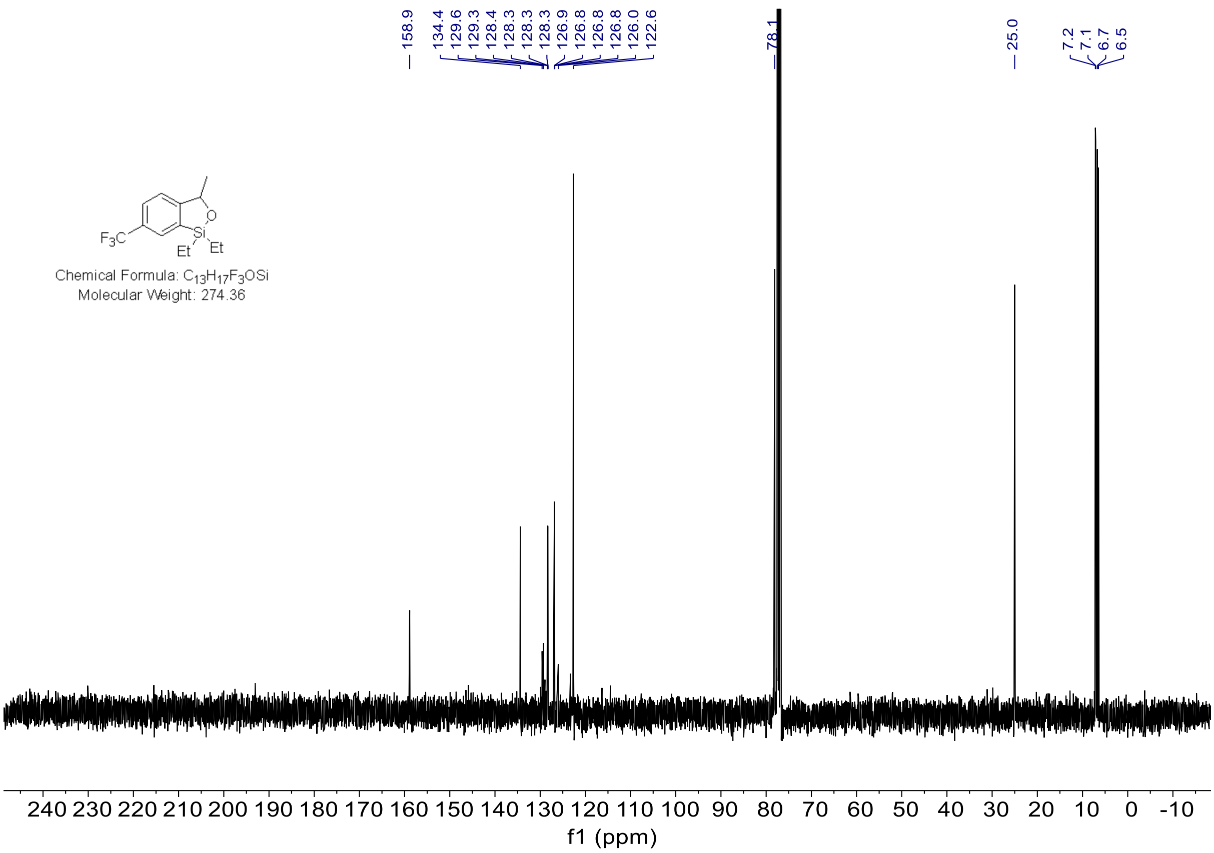

158.9
134.4
129.6
129.3
128.4
128.3
128.3
128.3
126.9
126.8
126.8
126.0
122.6
78.1
25.0
7.2
7.1
6.7
6.5
F3C
Si
O
Et
Et
Chemical Formula: C13H17F3OSi
Molecular Weight: 274.36
240 230 220 210 200 190 180 170 160 150 140 130 120 110 100 90 80 70 60 50 40 30 20 10 0 -10
f1 (ppm)

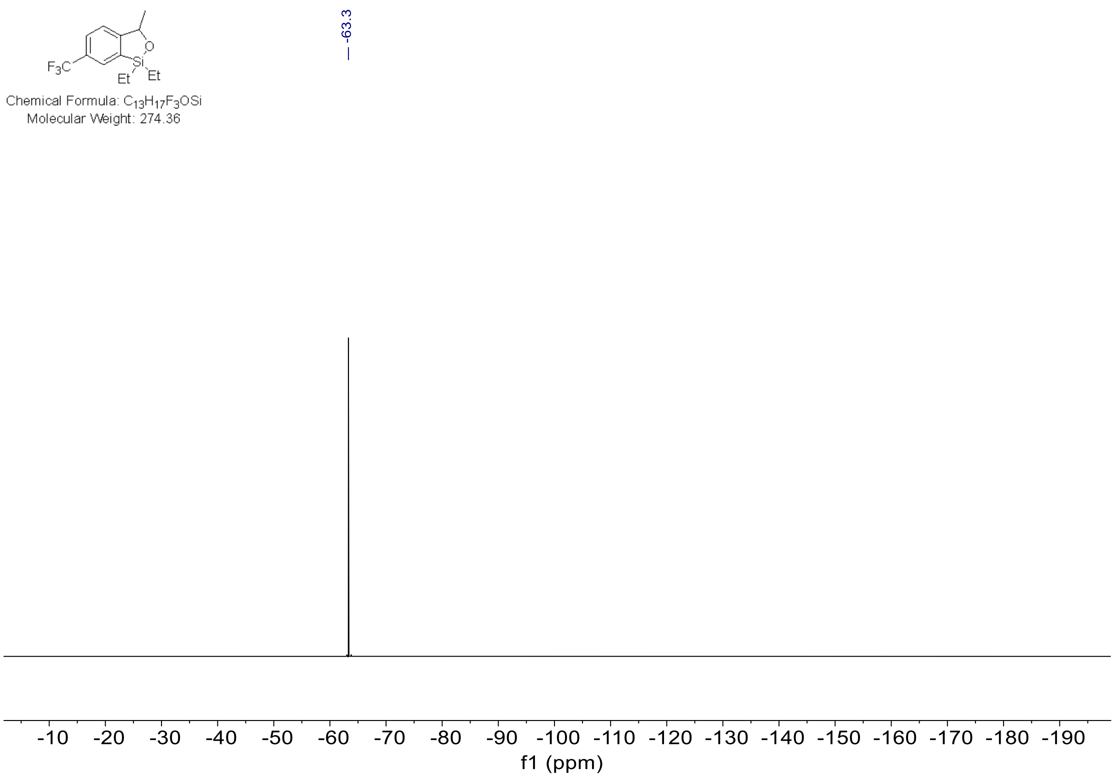

-63.3
F3C
Si
O
Et
Et
Chemical Formula: C13H17F3OSi
Molecular Weight: 274.36
-10 -20 -30 -40 -50 -60 -70 -80 -90 -100 -110 -120 -130 -140 -150 -160 -170 -180 -190
f1 (ppm)

1,1-Diethyl-6-methoxy-3-methyl-1,3-dihydrobenzo[c][1,2]oxasilole 4i.

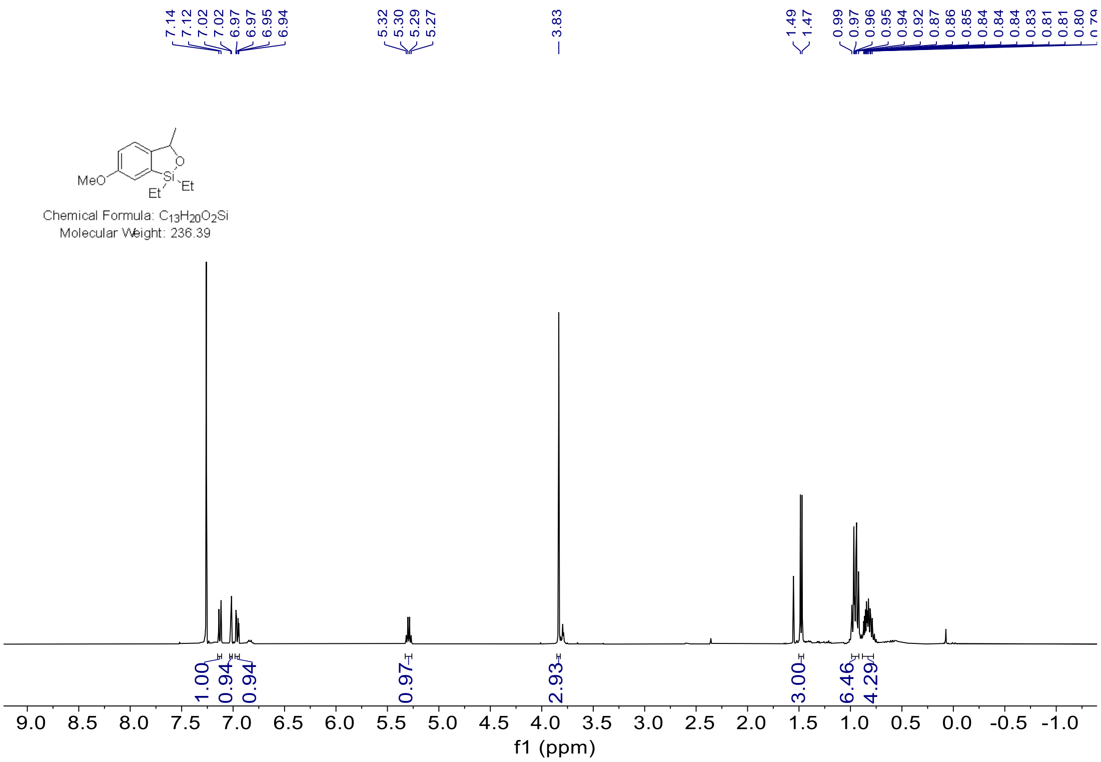

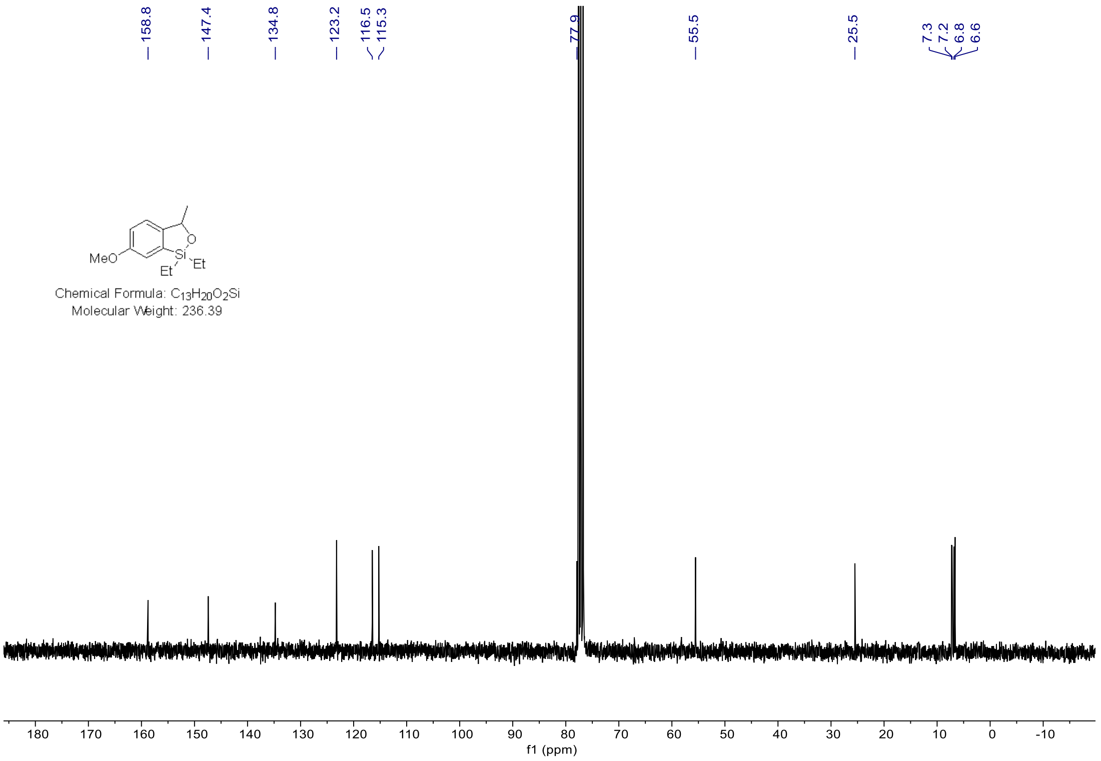

1,1-Diethyl-3-methyl-1,3-dihydronaphtho[2,3-c][1,2]oxasilole 4j.

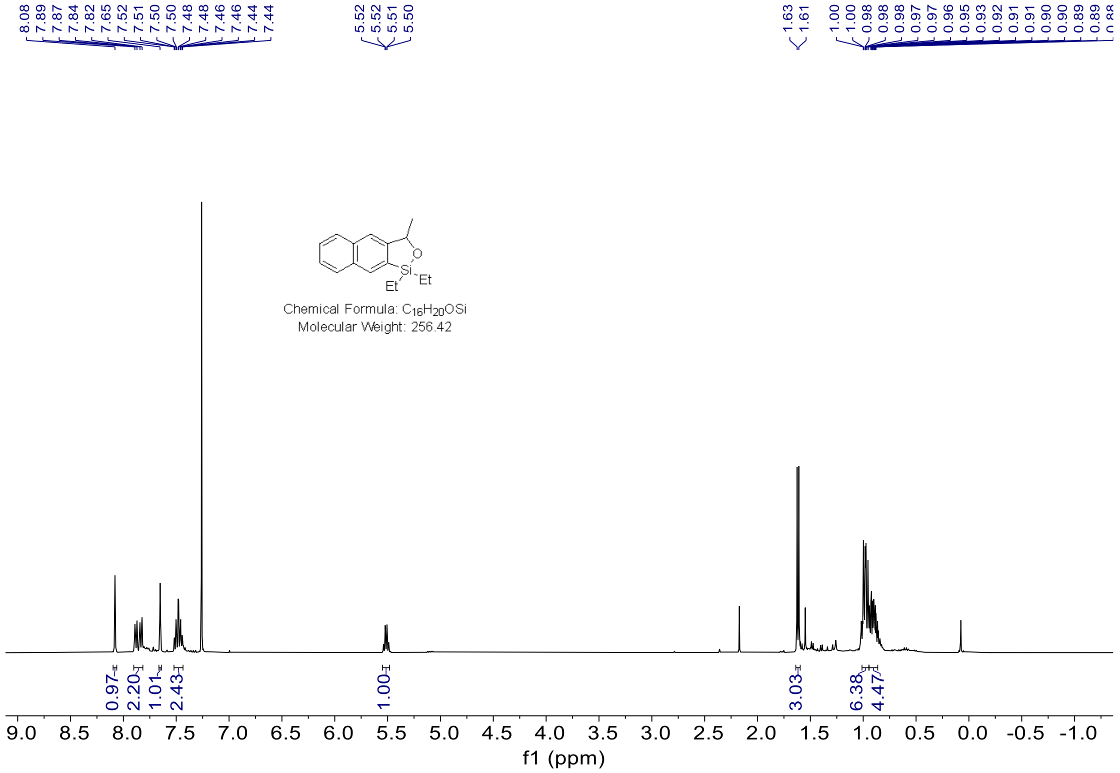

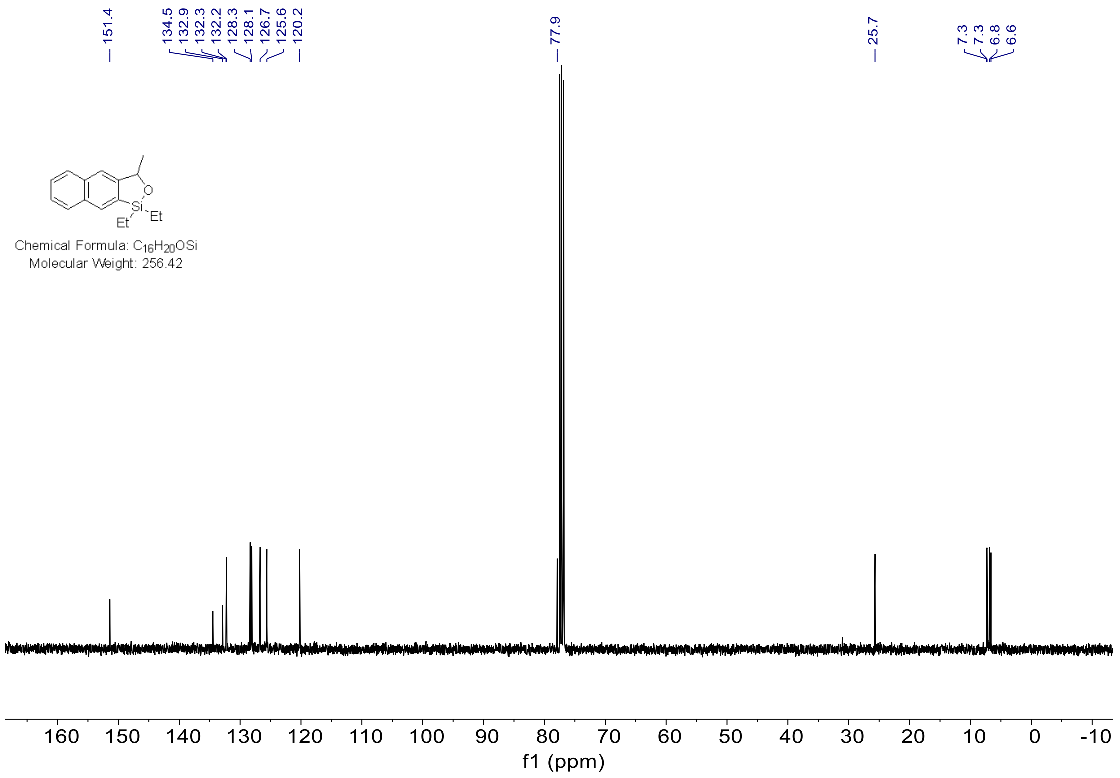

1,1,5,5-Tetraethyl-3,7-dimethyl-5,7-dihydro-1H,3H-benzo[1,2-c:4,5-c']bis([1,2]oxasilole) 4k.

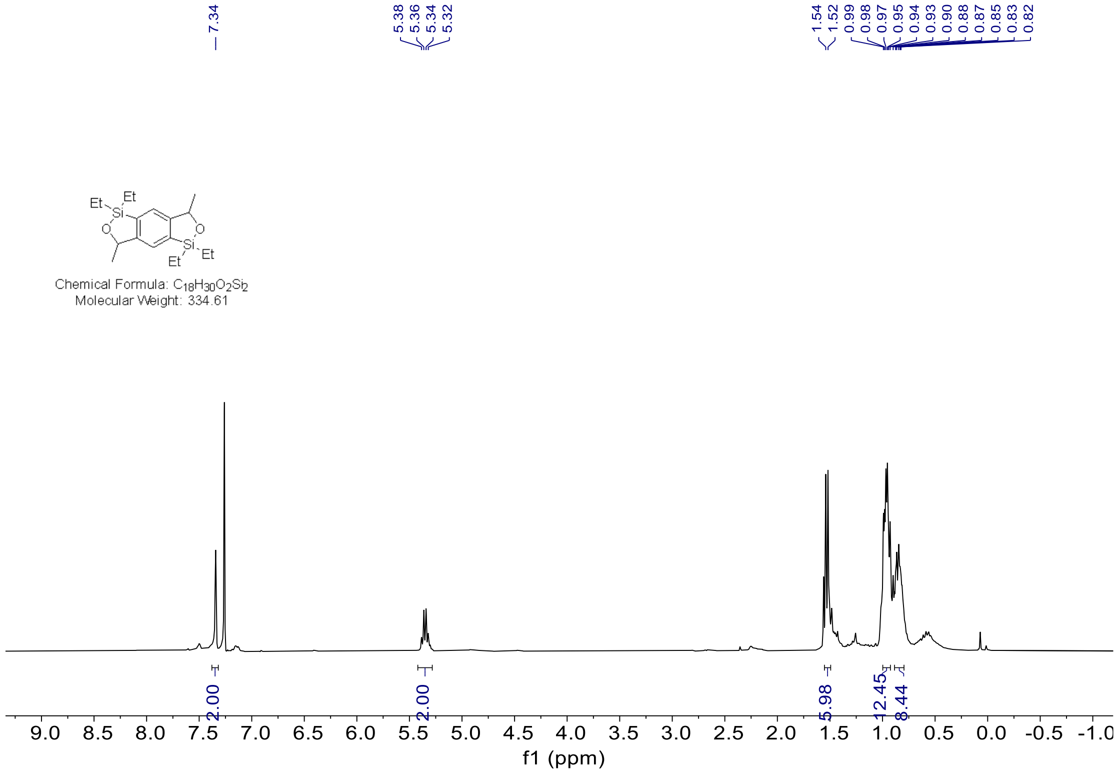

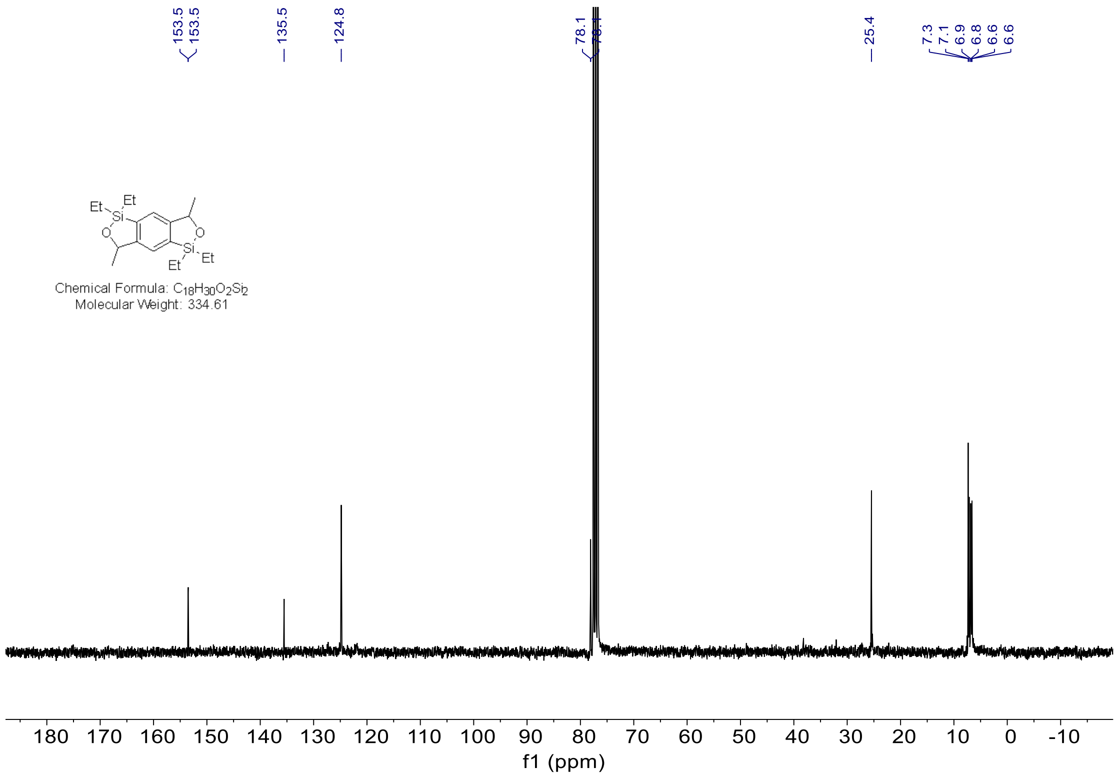

1,1,3-Triethyl-1,3-dihydrobenzo[c][1,2]oxasilole 4l.

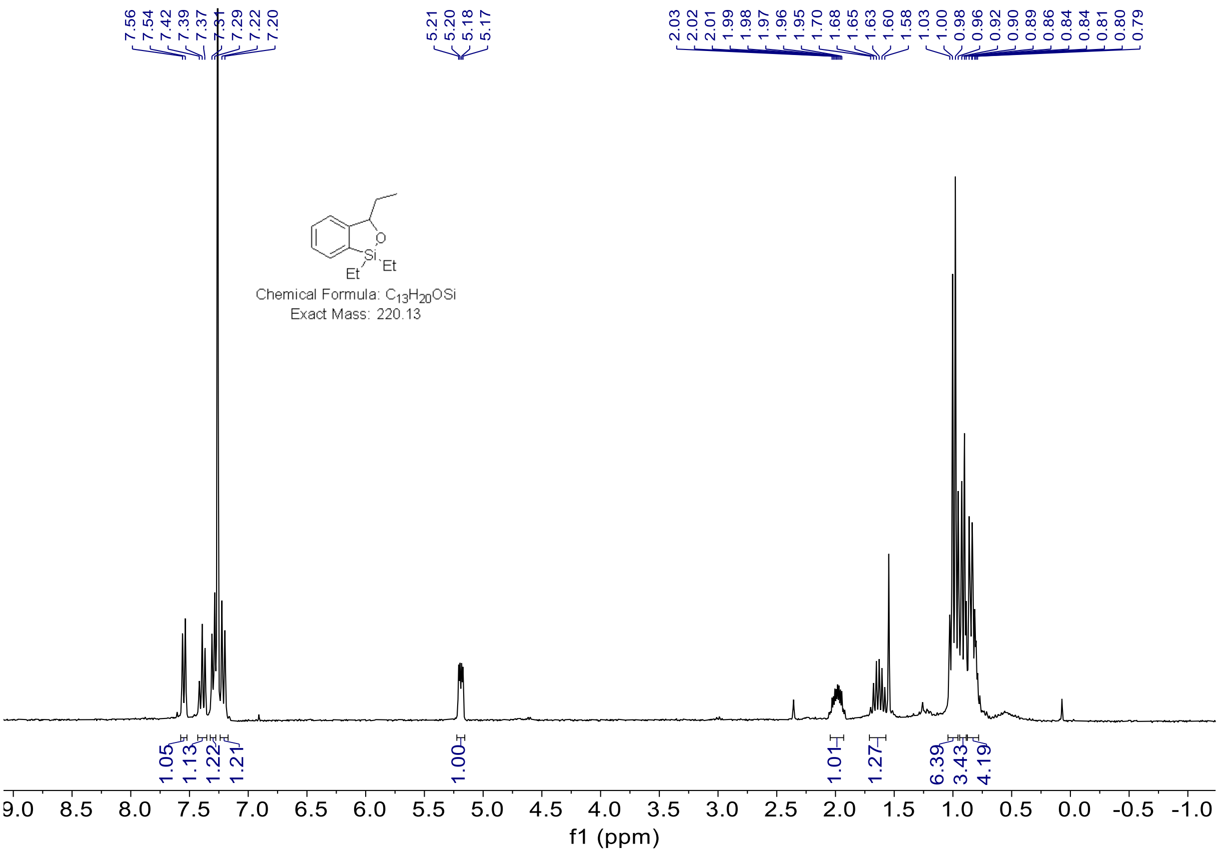

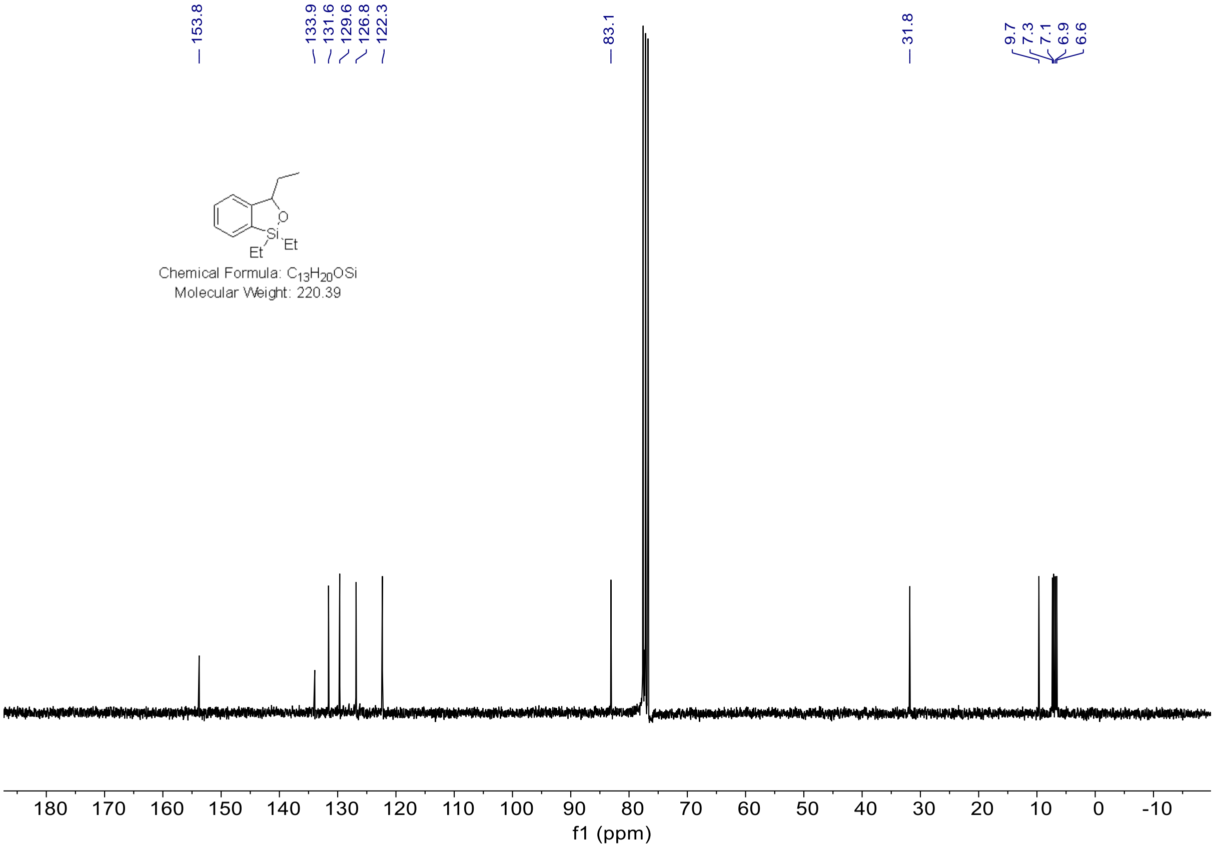

1,1-Diethyl-3-propyl-1,3-dihydrobenzo[c][1,2]oxasilole 4m.

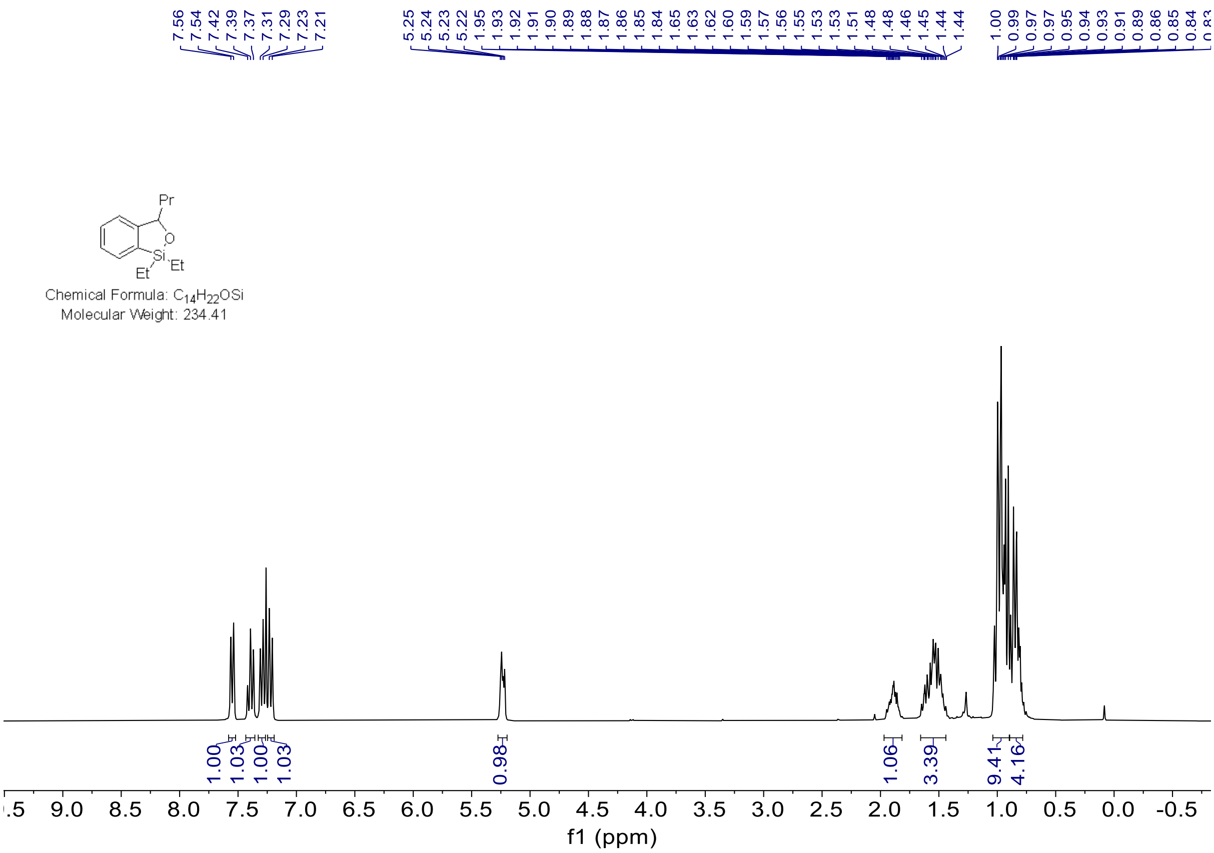

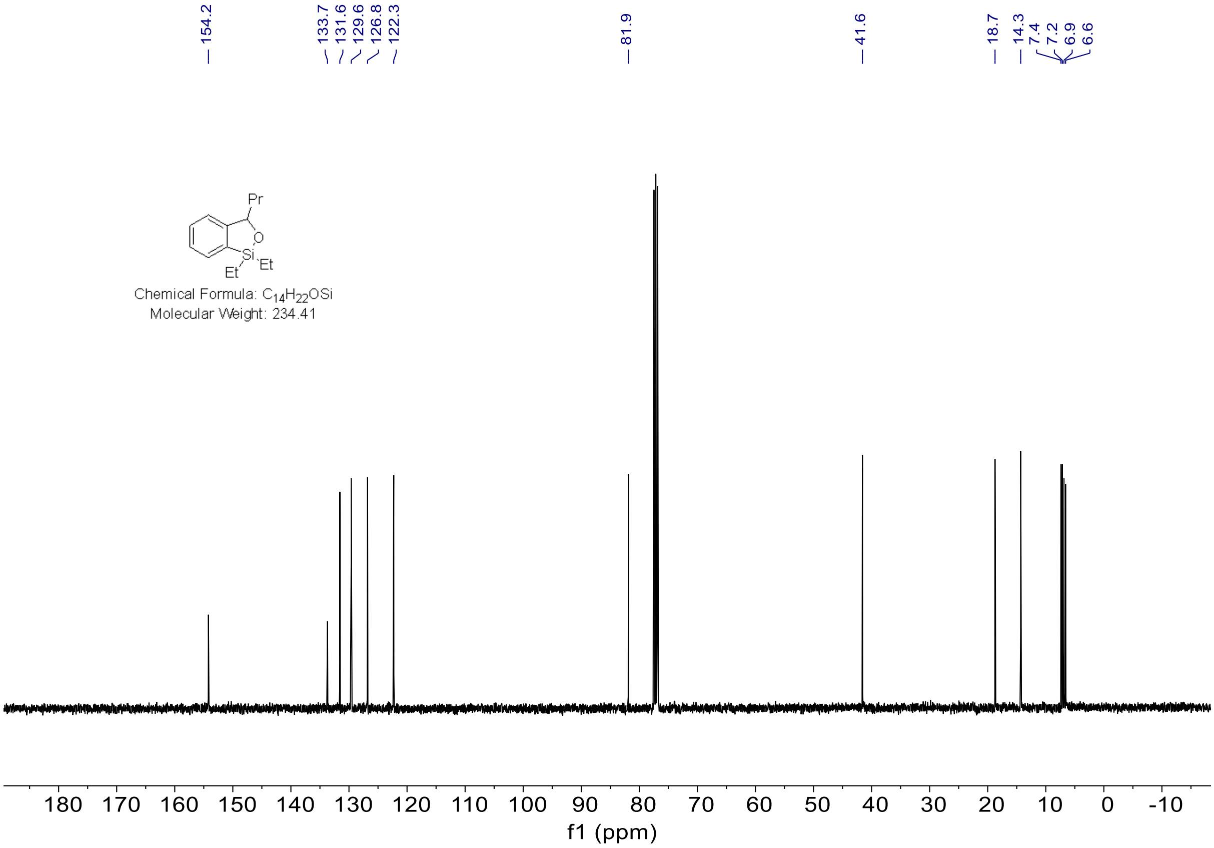

3-Butyl-1,1-diethyl-1,3-dihydrobenzo[c][1,2]oxasilole 4n.

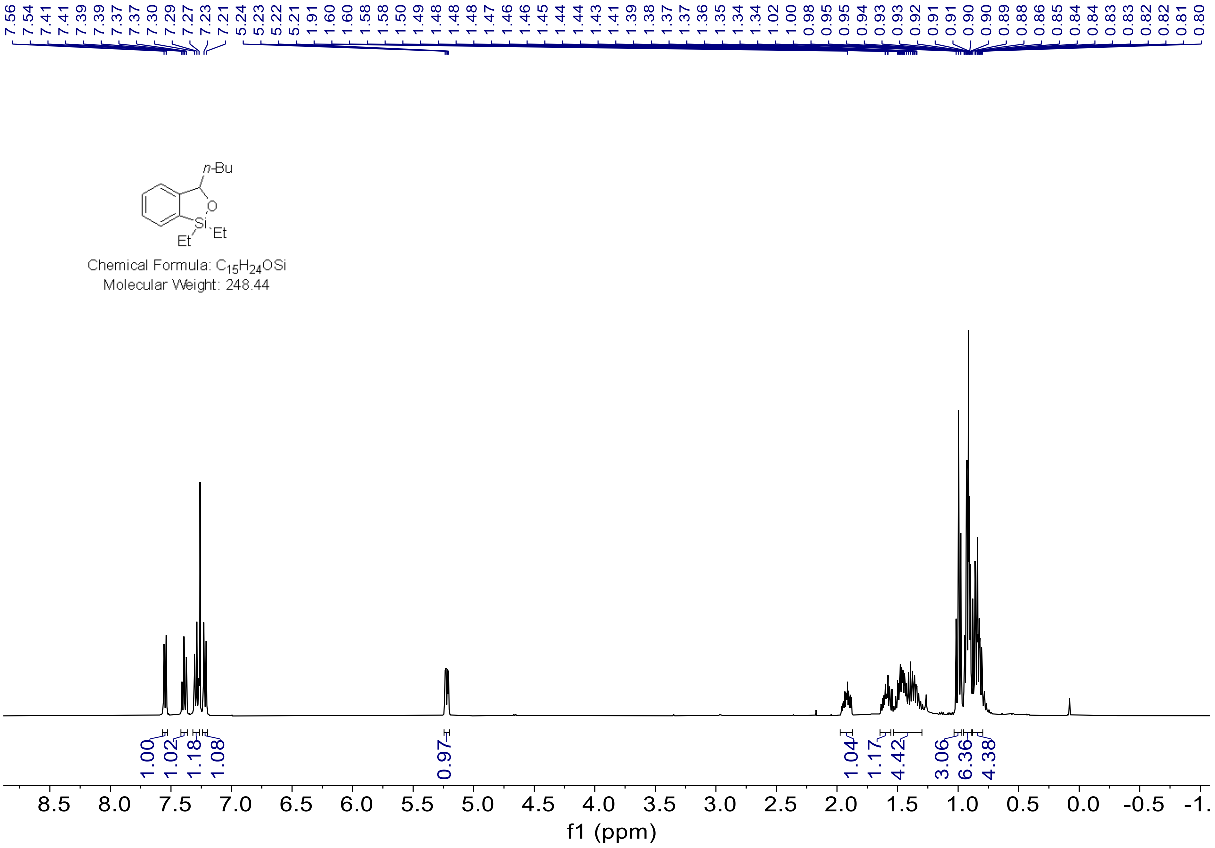

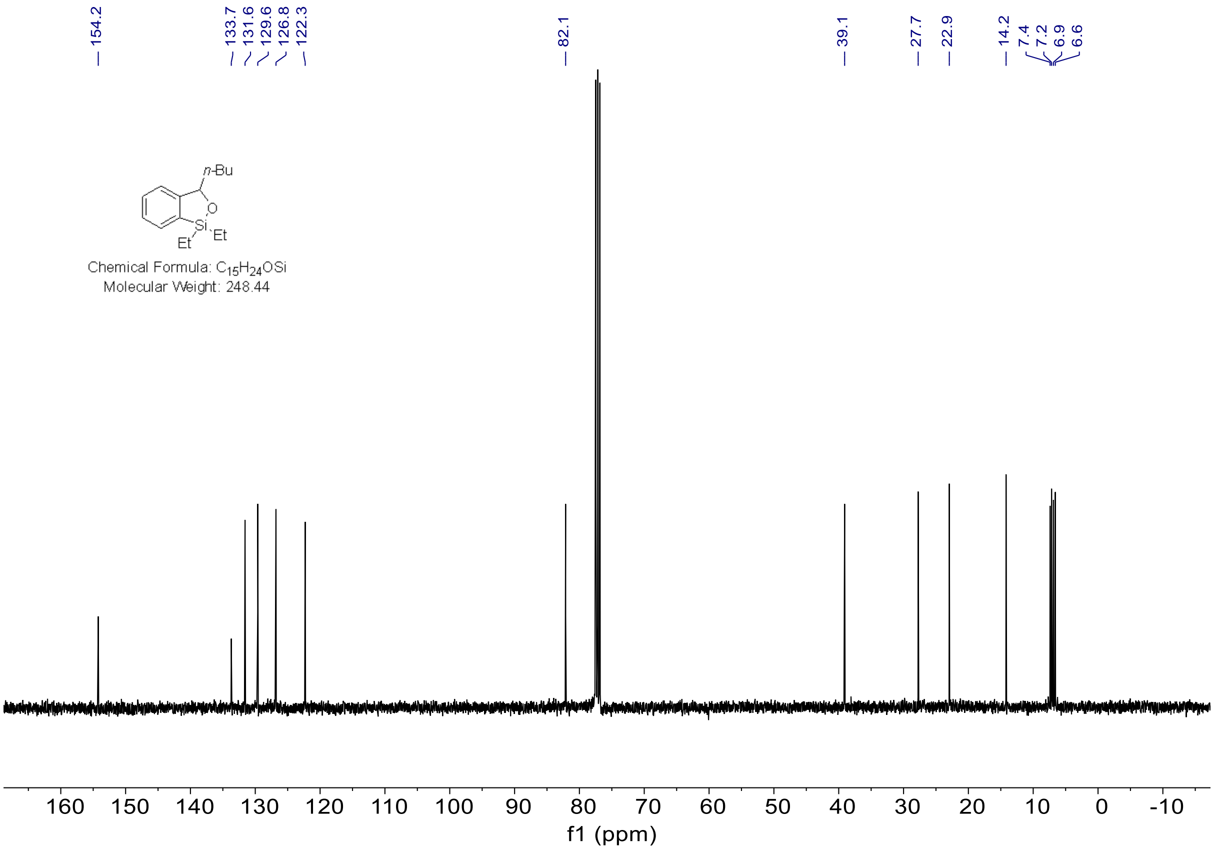

1,1-diethyl-3-isopropyl-1,3-dihydrobenzo[c][1,2]oxasilole 4o.

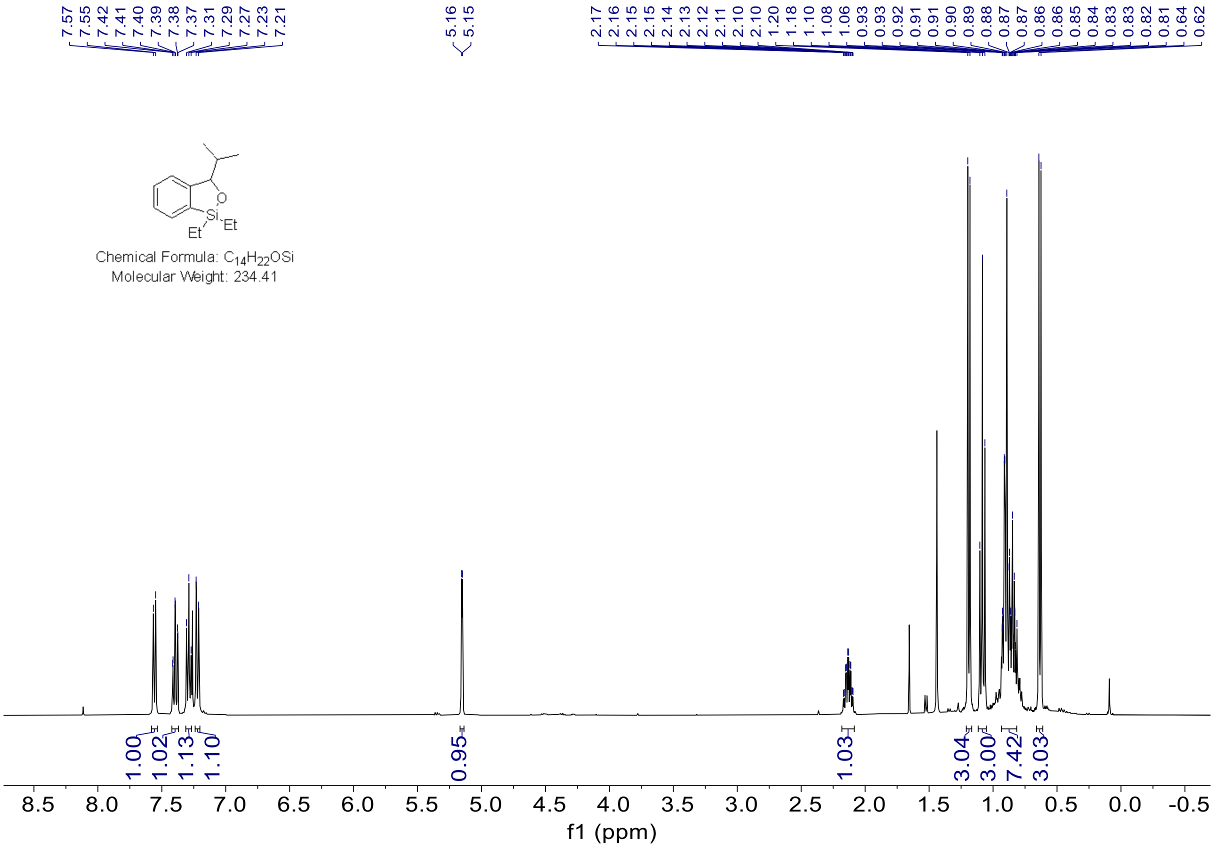

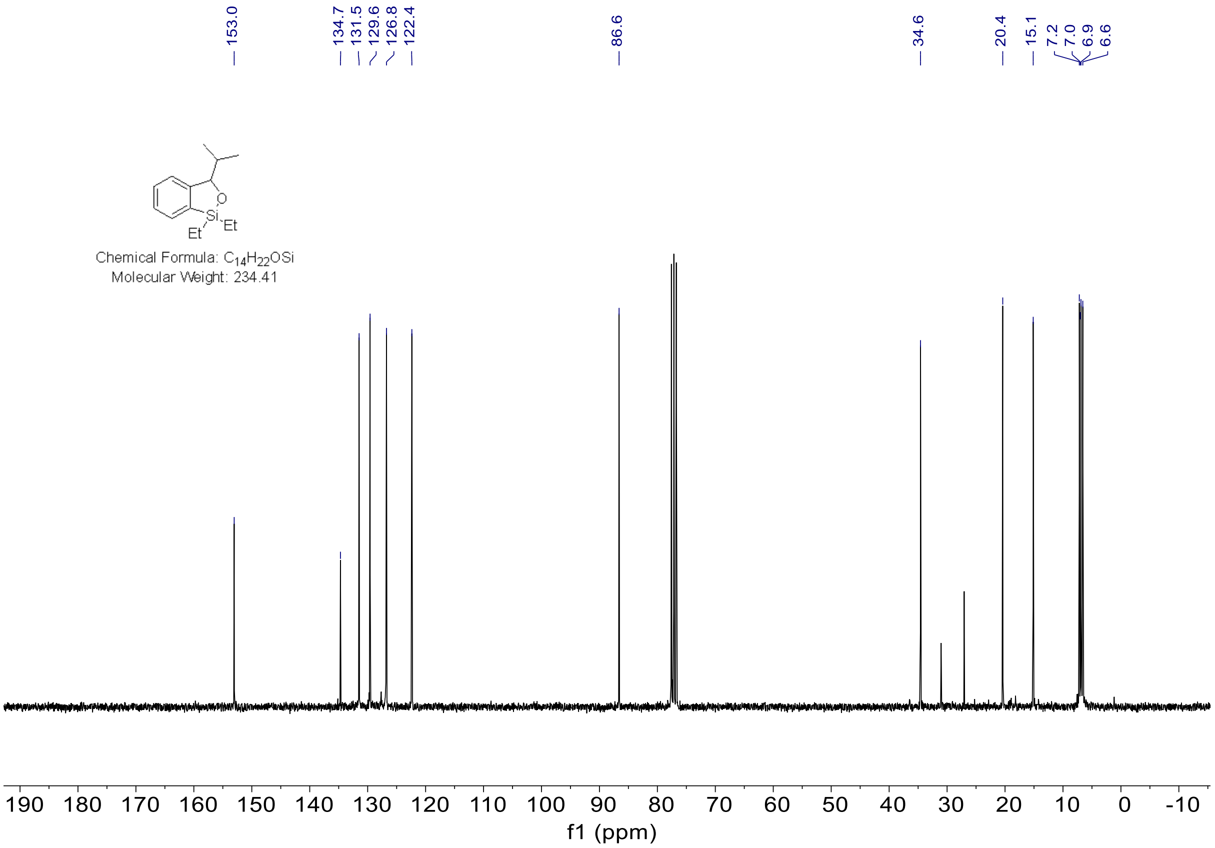

1,1-Diethyl-3-phenyl-1,3-dihydrobenzo[c][1,2]oxasilole 4p.

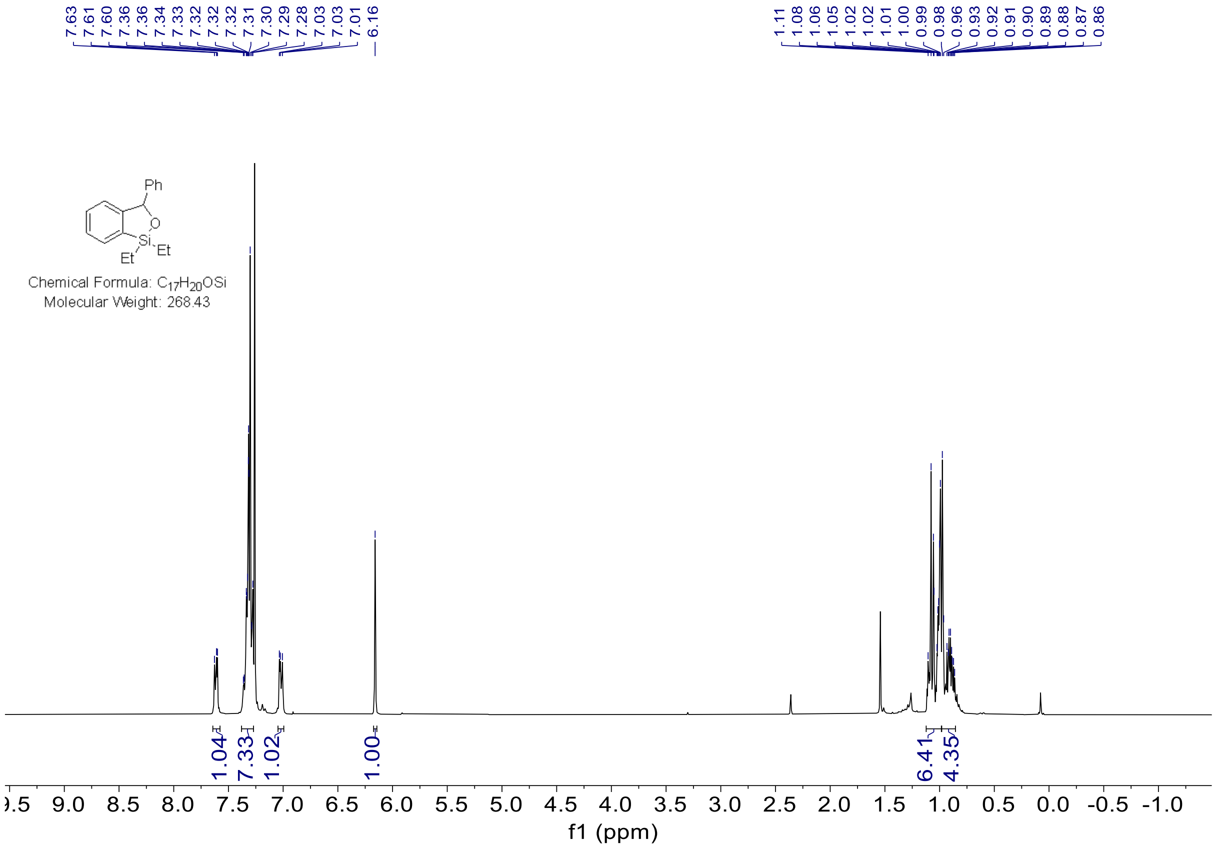

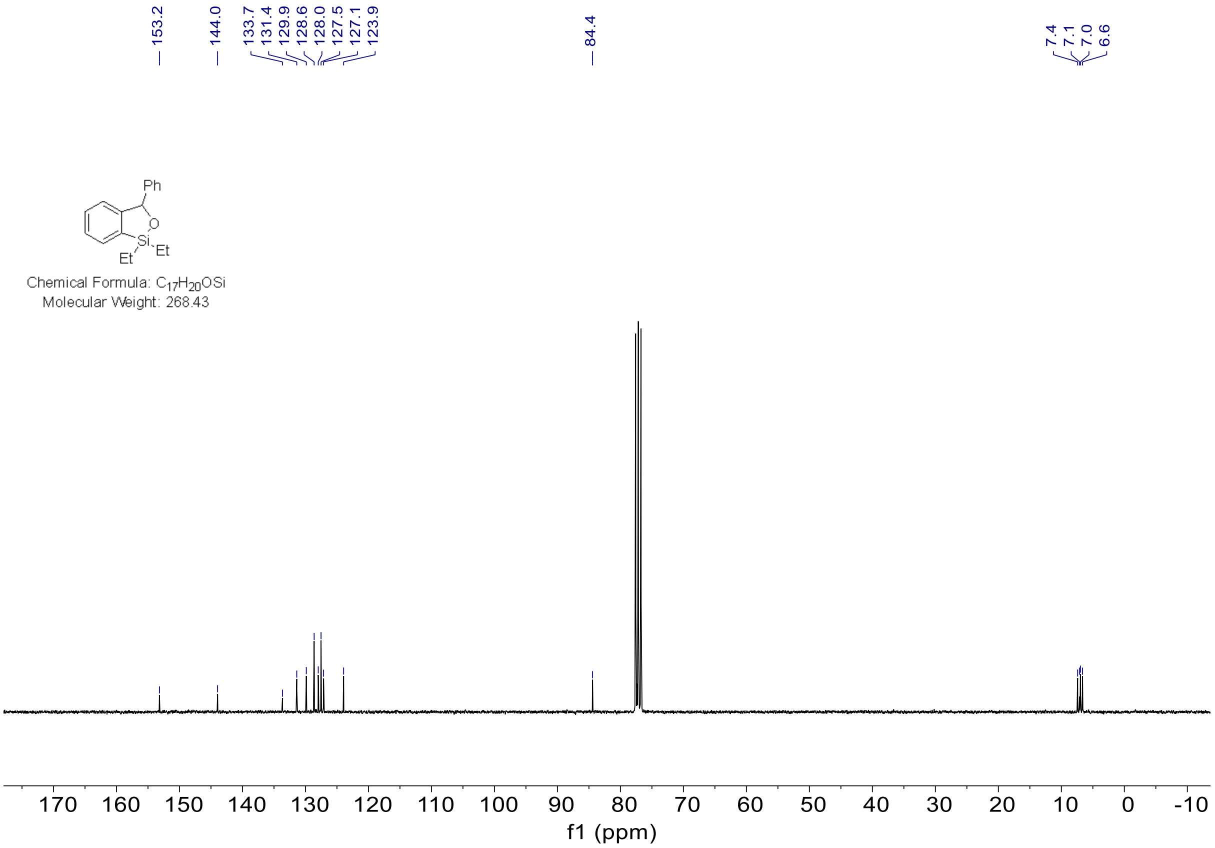

1,1-diethyl-6-methyl-3-(p-tolyl)-1,3-dihydrobenzo[c][1,2]oxasilole 4q.

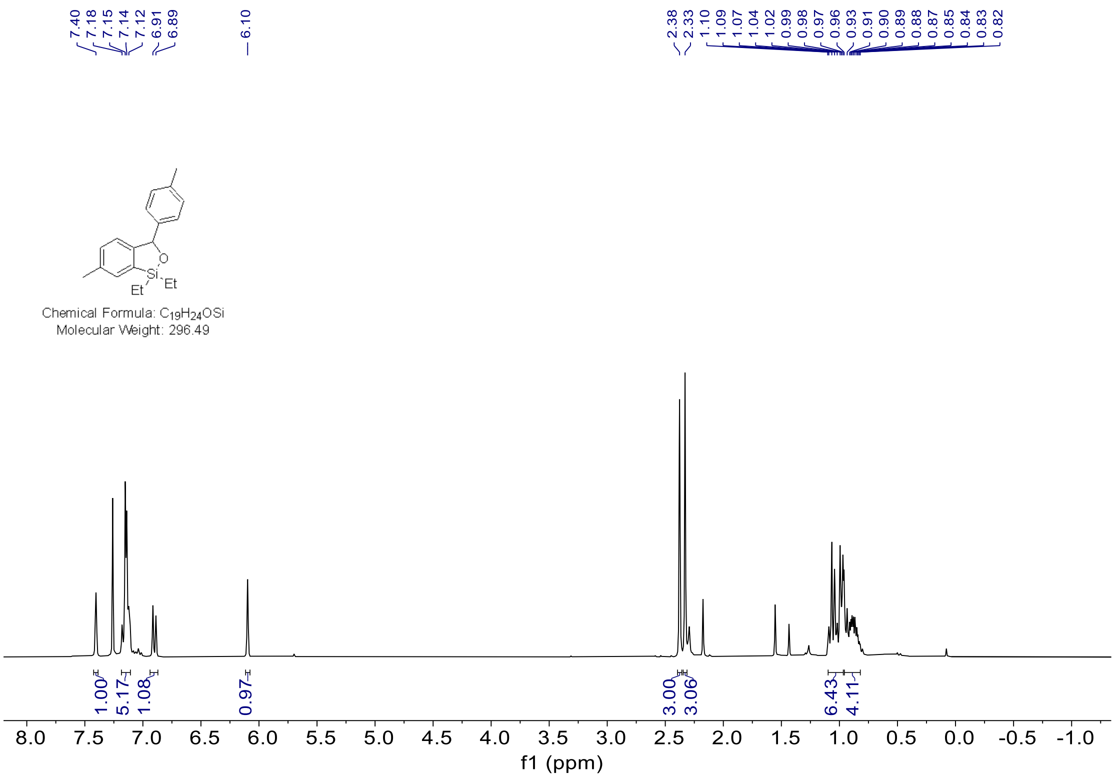
7.40
7.18
7.15
7.14
7.12
6.91
6.89
6.10
2.38
2.33
1.10
1.09
1.07
1.04
1.02
0.99
0.98
0.97
0.96
0.93
0.91
0.90
0.89
0.88
0.87
0.85
0.84
0.83
0.82
O
Si
Et
Et
Chemical Formula: $C_{19}H_{24}OSi$
Molecular Weight: 296.49
1.00
5.17
1.08
0.97
3.00
3.06
6.43
4.11
8.0 7.5 7.0 6.5 6.0 5.5 5.0 4.5 4.0 3.5 3.0 2.5 2.0 1.5 1.0 0.5 0.0 -0.5 -1.0
f1 (ppm)

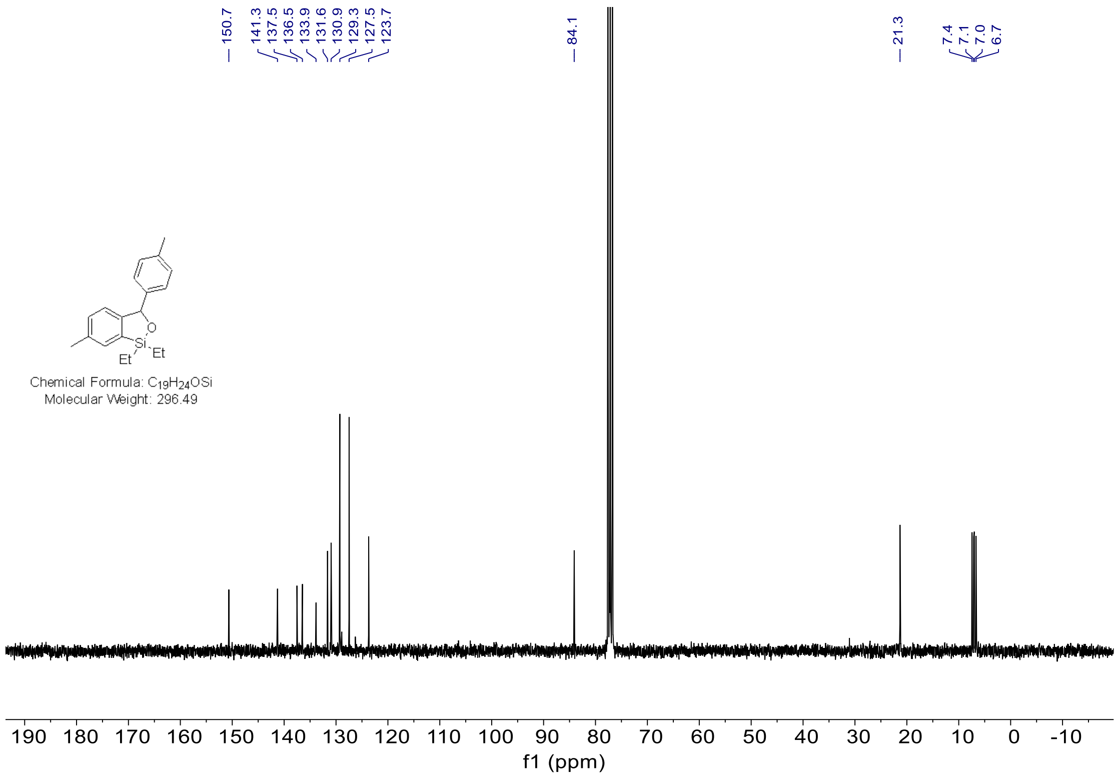
150.7
141.3
137.5
136.5
133.9
131.6
130.9
129.3
127.5
123.7
84.1
21.3
7.4
7.1
7.0
6.7
O
Si
Et
Et
Chemical Formula: $C_{19}H_{24}OSi$
Molecular Weight: 296.49
190 180 170 160 150 140 130 120 110 100 90 80 70 60 50 40 30 20 10 0 -10
f1 (ppm)

2-Bromo-1,1′:4′,1″-terphenyl.

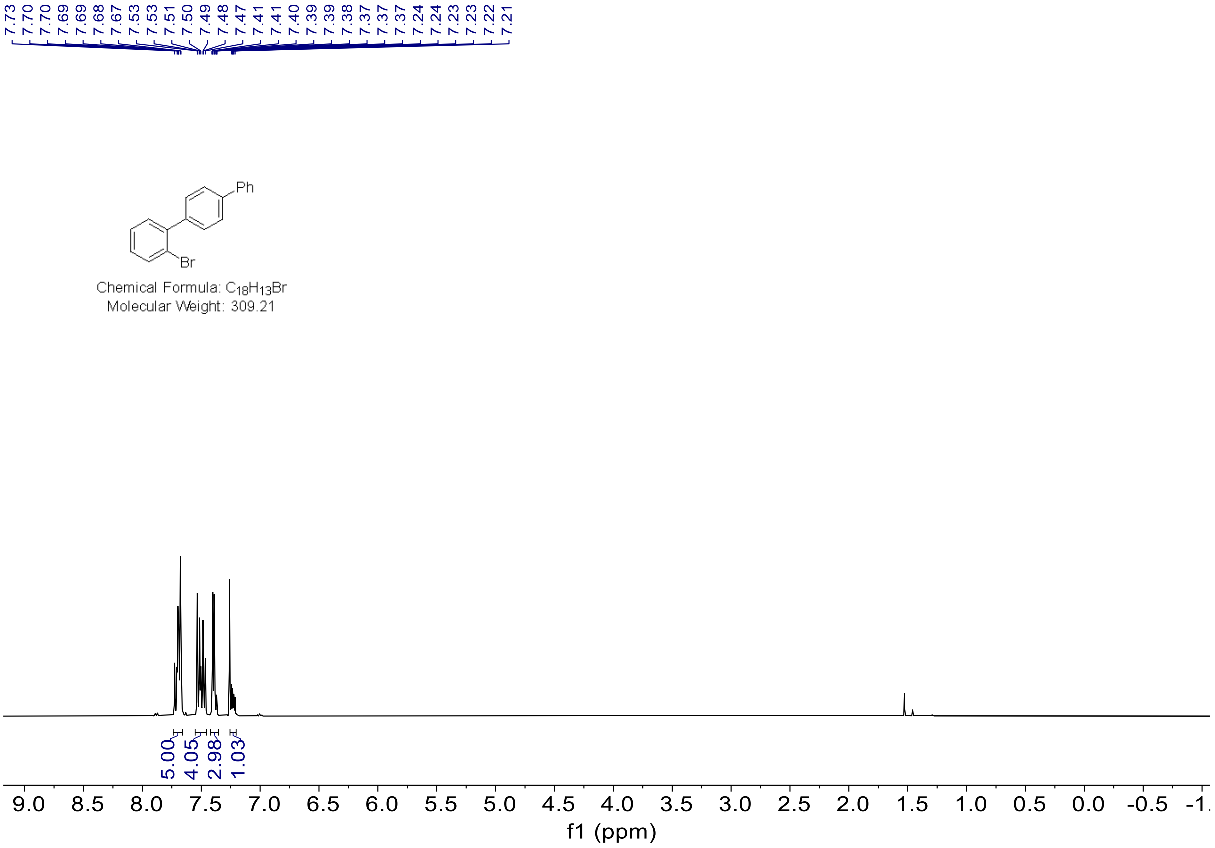

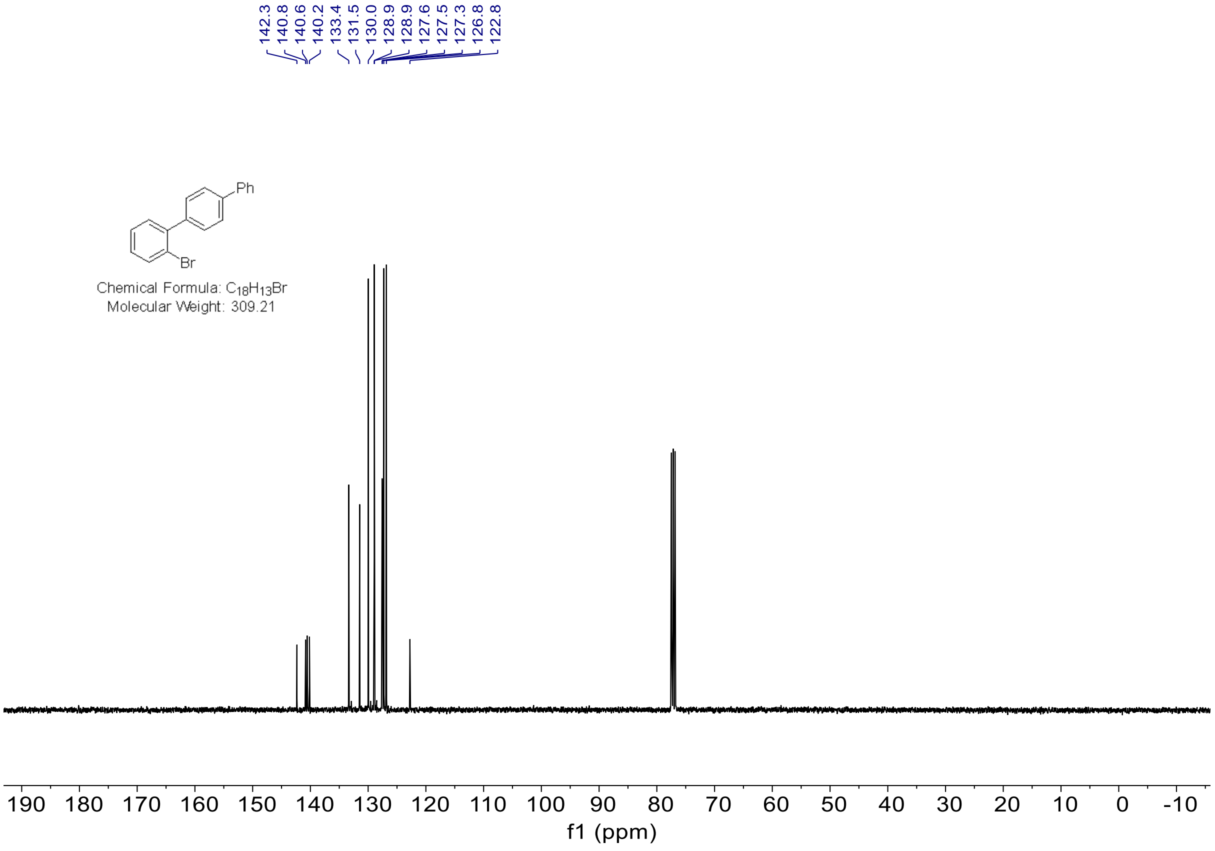

2-Bromo-4′-methyl-1,1′-biphenyl

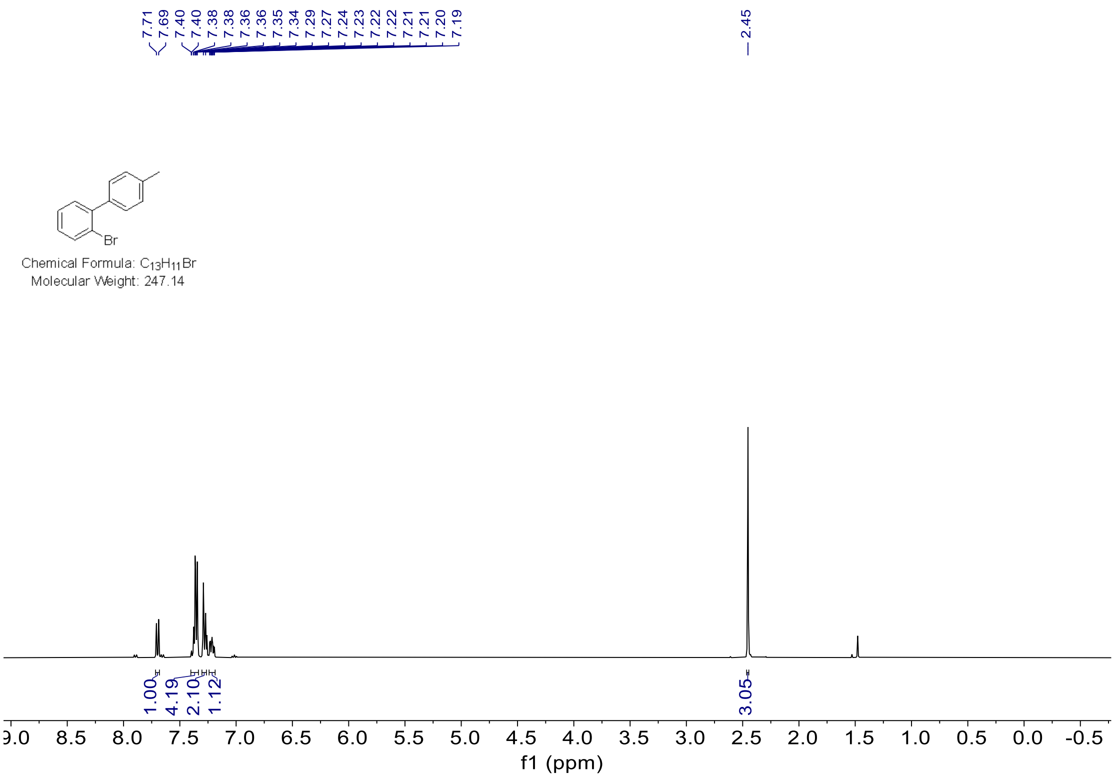

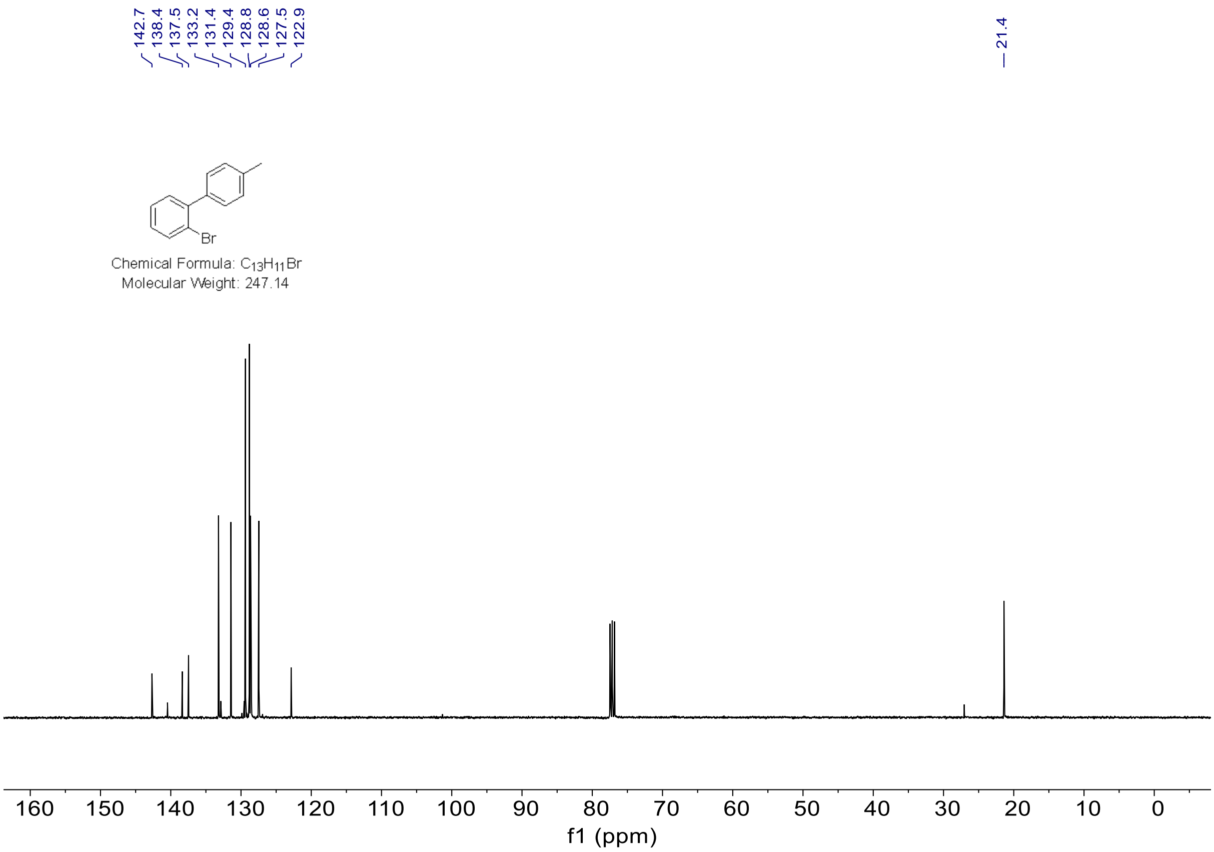

2-Bromo-4'-isopropyl-1,1'-biphenyl

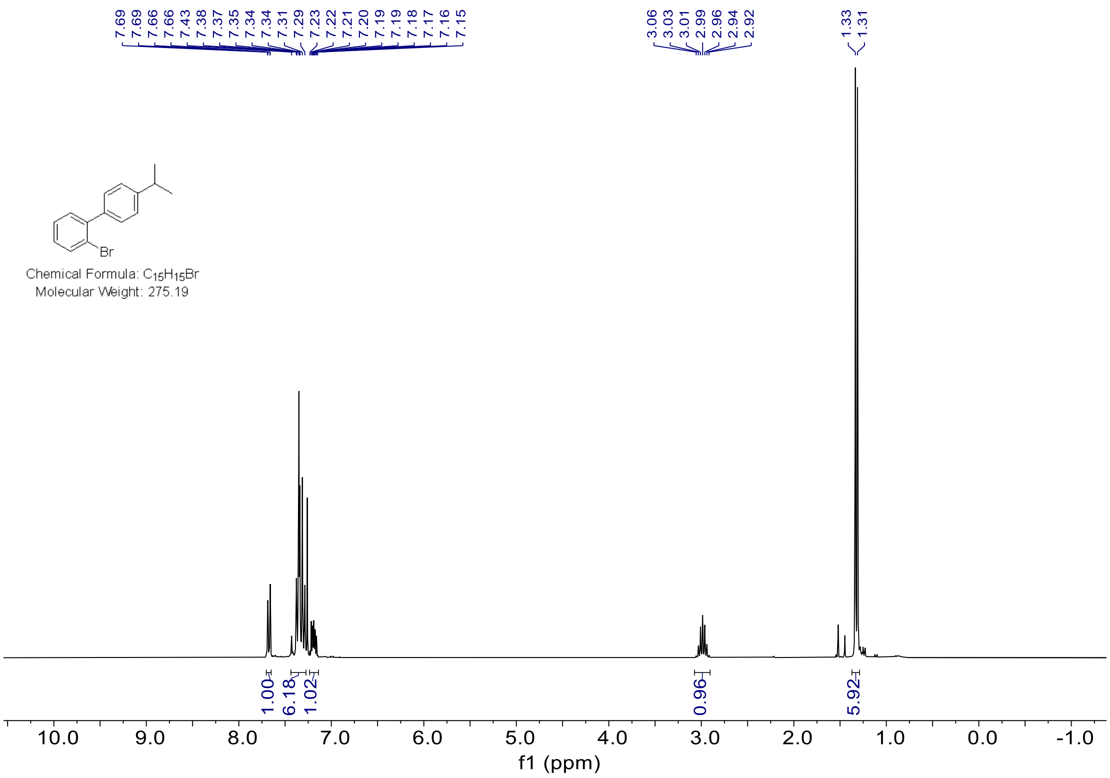
7.69
7.69
7.66
7.66
7.43
7.38
7.37
7.35
7.34
7.31
7.29
7.23
7.22
7.21
7.20
7.19
7.19
7.18
7.17
7.16
7.15
3.06
3.03
3.01
2.99
2.96
2.94
2.92
1.33
1.31
Br
Chemical Formula: C15H15Br
Molecular Weight: 275.19
1.00
6.18
1.02
0.96
5.92
10.0
9.0
8.0
7.0
6.0
5.0
4.0
3.0
2.0
1.0
0.0
-1.0
f1 (ppm)

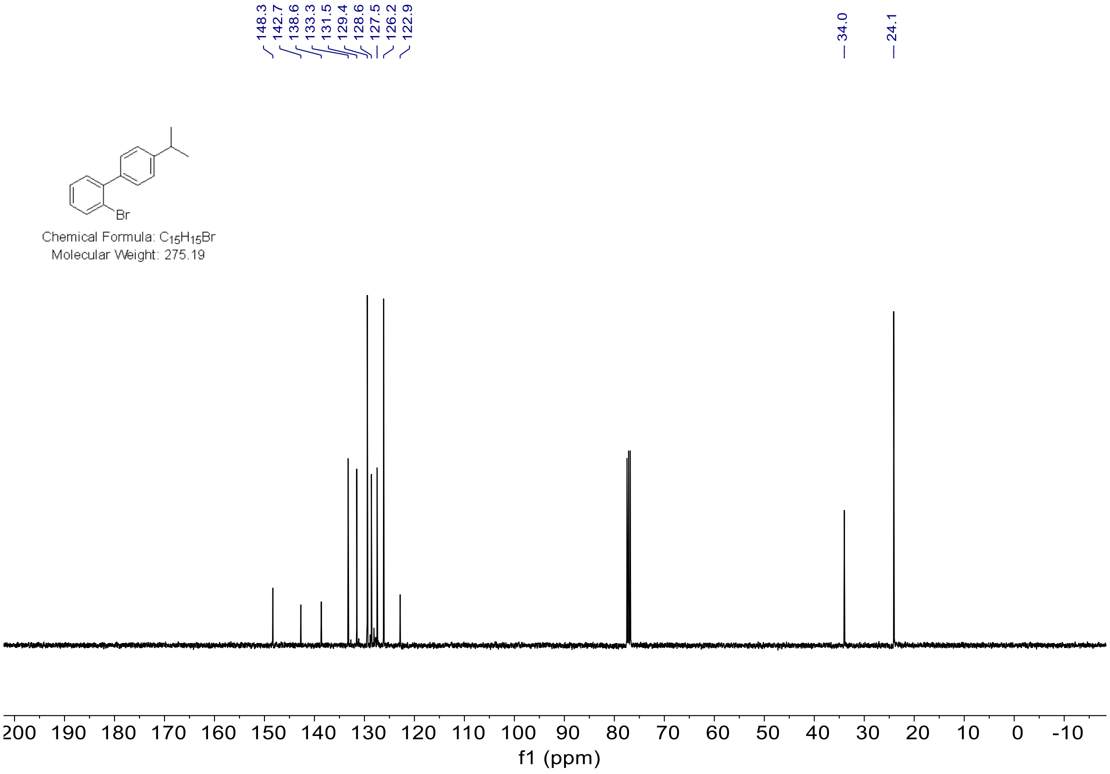
148.3
142.7
138.6
133.3
131.5
129.4
128.6
127.5
126.2
122.9
34.0
24.1
Br
Chemical Formula: C15H15Br
Molecular Weight: 275.19
200
190
180
170
160
150
140
130
120
110
100
90
80
70
60
50
40
30
20
10
0
-10
f1 (ppm)

2-Bromo-4'-(*tert*-butyl)-1,1'-biphenyl

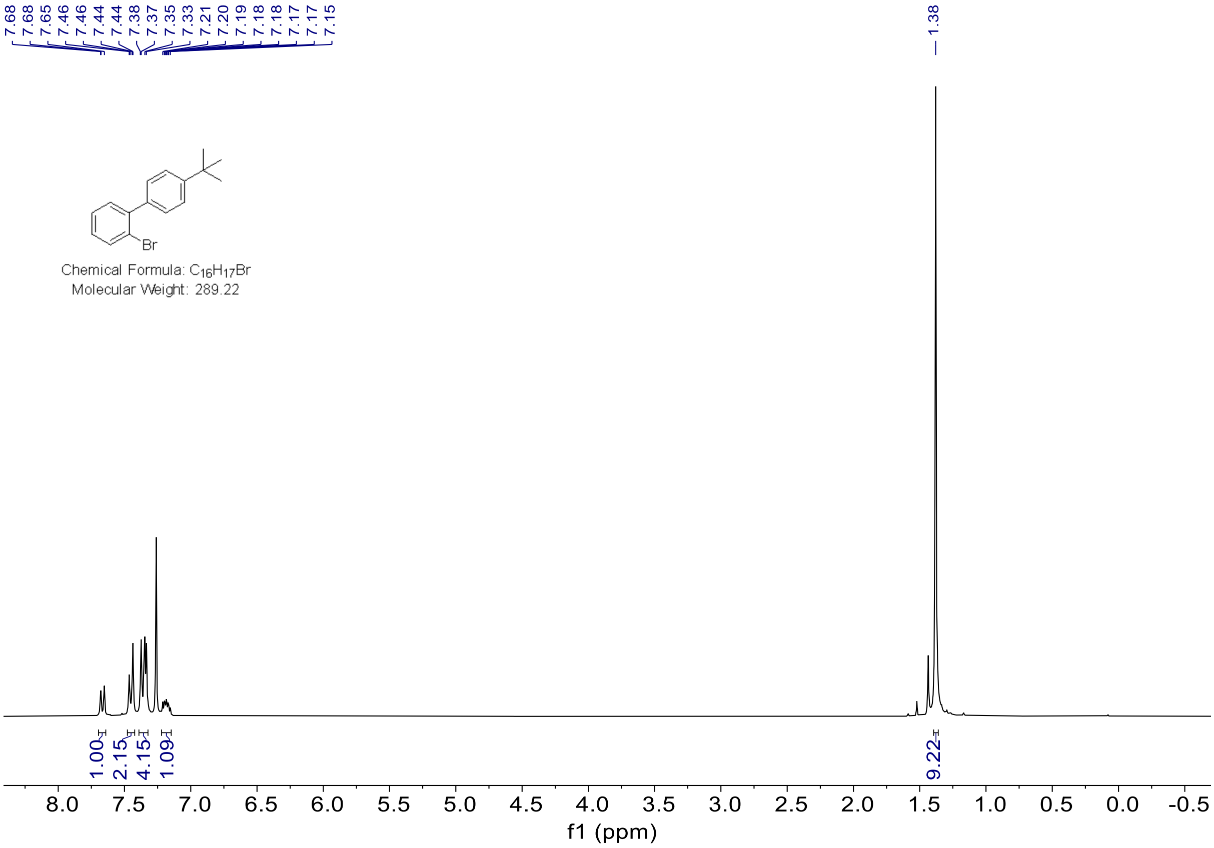

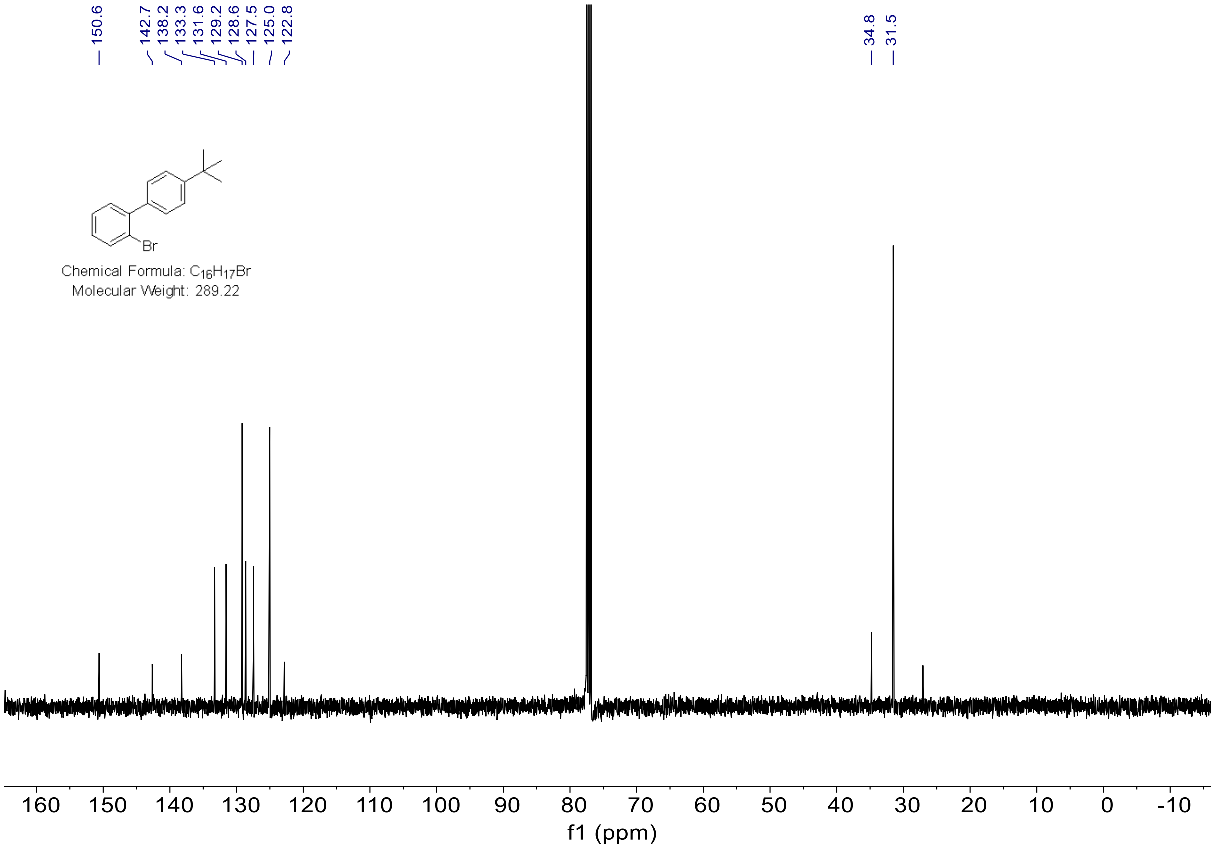

2-Bromo-4′-trifluoromethyl-1,1′-biphenyl

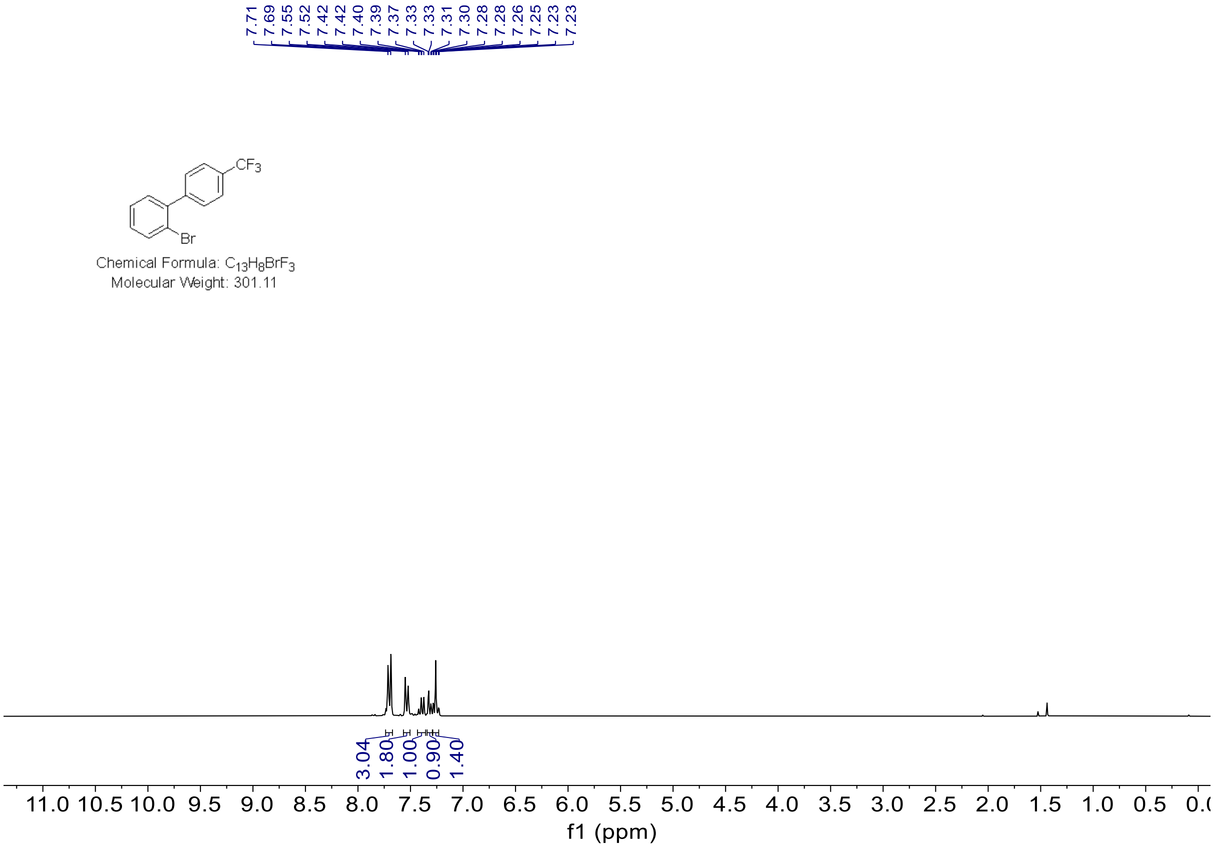

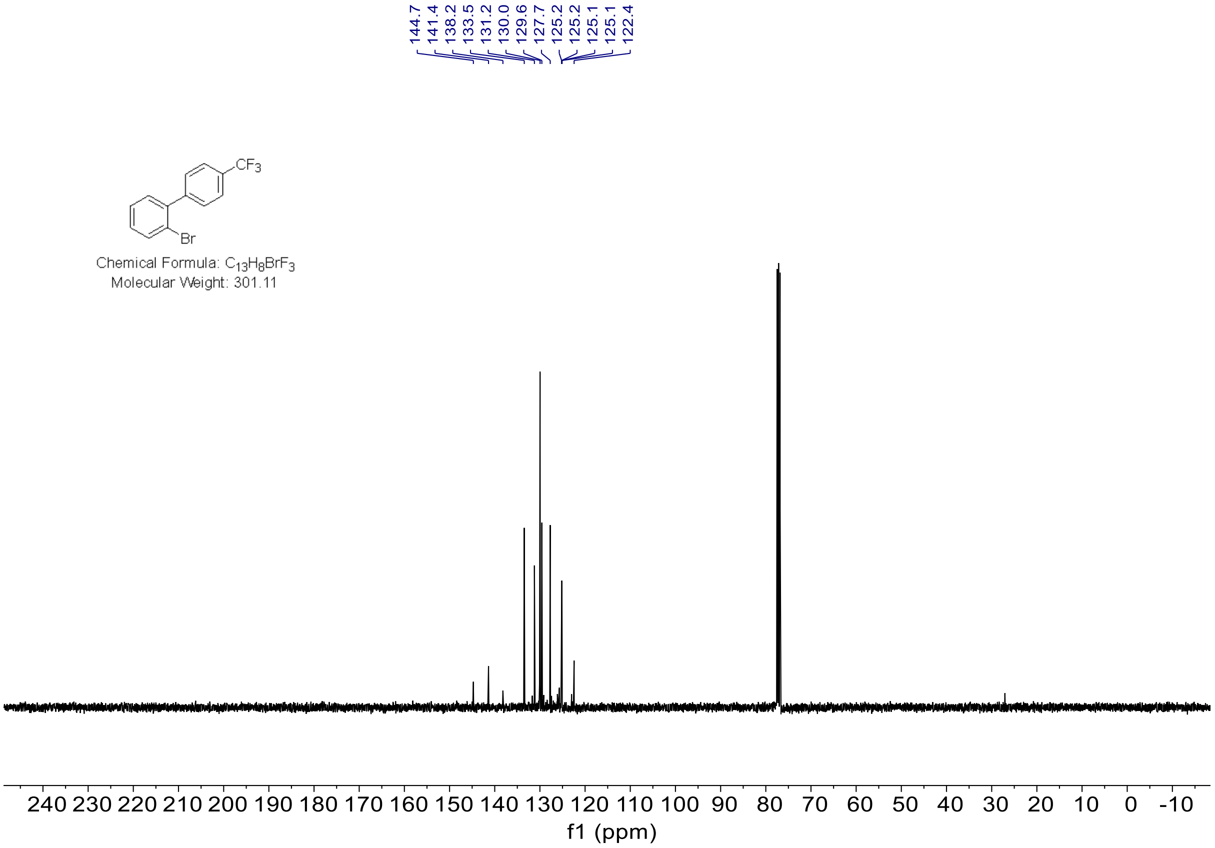

2-Bromo-4′-fluoro -1,1′-biphenyl

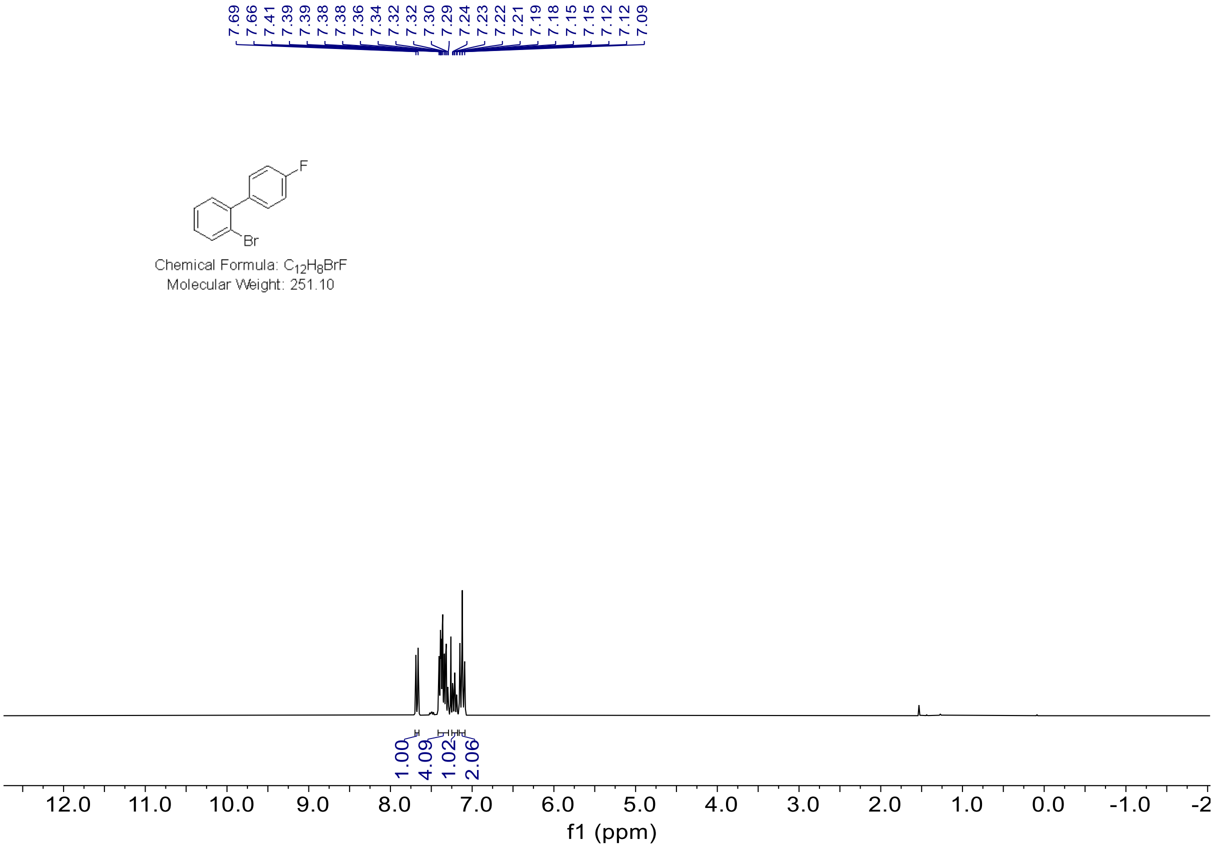
7.69 7.66 7.41 7.39 7.39 7.38 7.38 7.36 7.34 7.32 7.32 7.30 7.29 7.24 7.23 7.22 7.21 7.19 7.18 7.15 7.15 7.12 7.12 7.09
F
Br
Chemical Formula: $C_{12}H_8BrF$
Molecular Weight: 251.10
1.00 4.09 1.02 2.06
12.0 11.0 10.0 9.0 8.0 7.0 6.0 5.0 4.0 3.0 2.0 1.0 0.0 -1.0 -2
f1 (ppm)

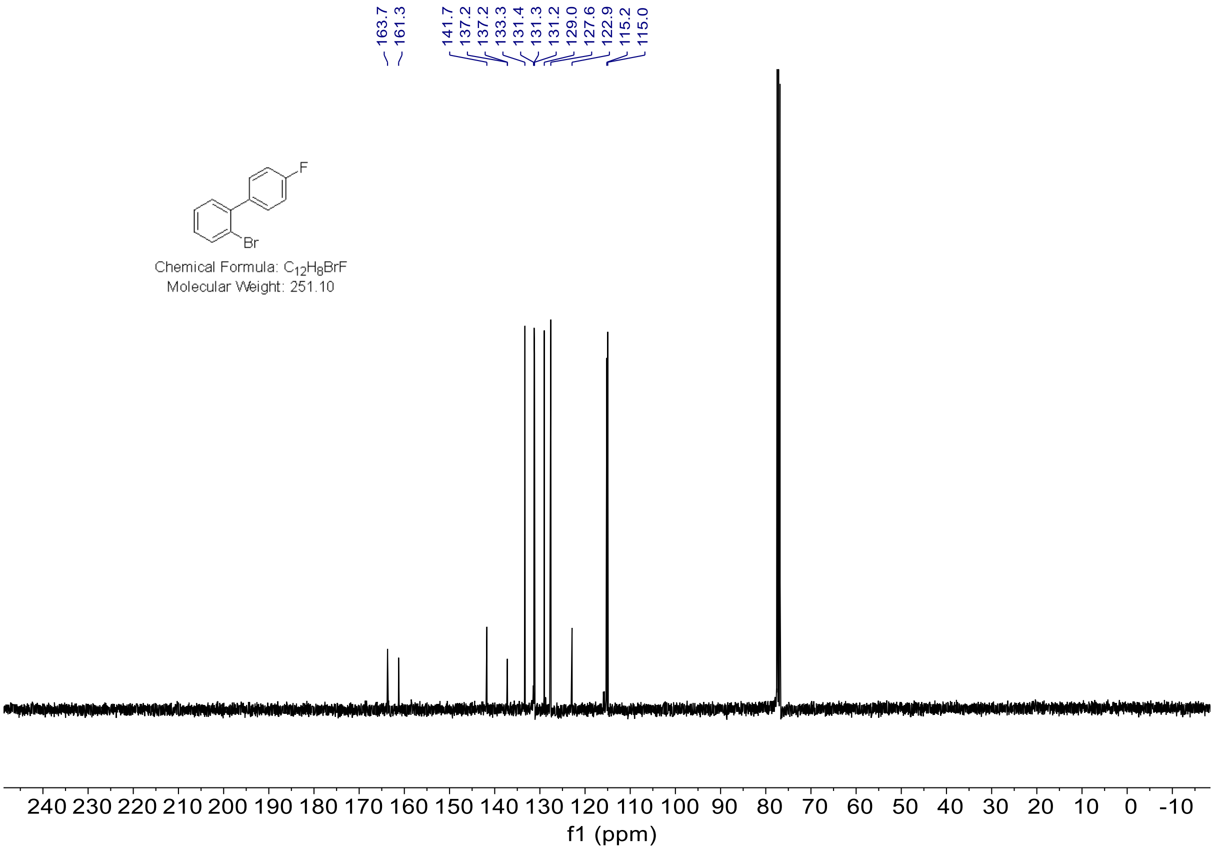
163.7 161.3
141.7 137.2 137.2 133.3 131.4 131.3 131.2 129.0 127.6 122.9 115.2 115.0
F
Br
Chemical Formula: $C_{12}H_8BrF$
Molecular Weight: 251.10
240 230 220 210 200 190 180 170 160 150 140 130 120 110 100 90 80 70 60 50 40 30 20 10 0 -10
f1 (ppm)

2-Bromo-4'-(trifluoromethoxy)-1,1'-biphenyl

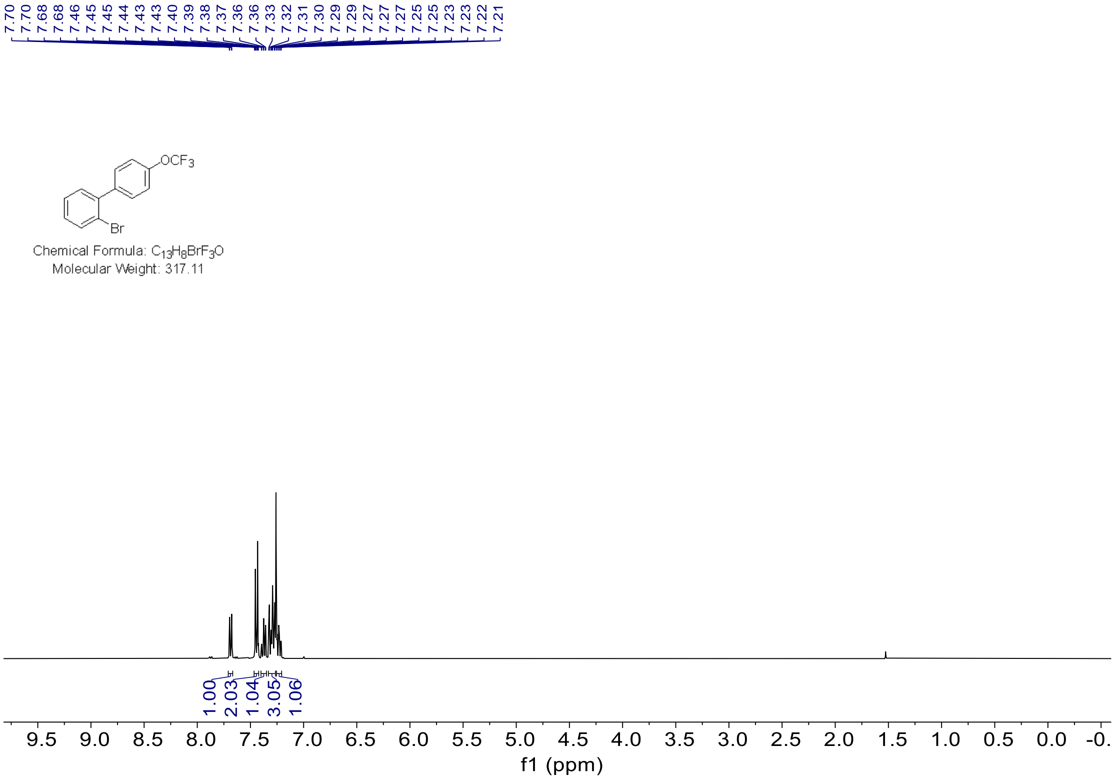

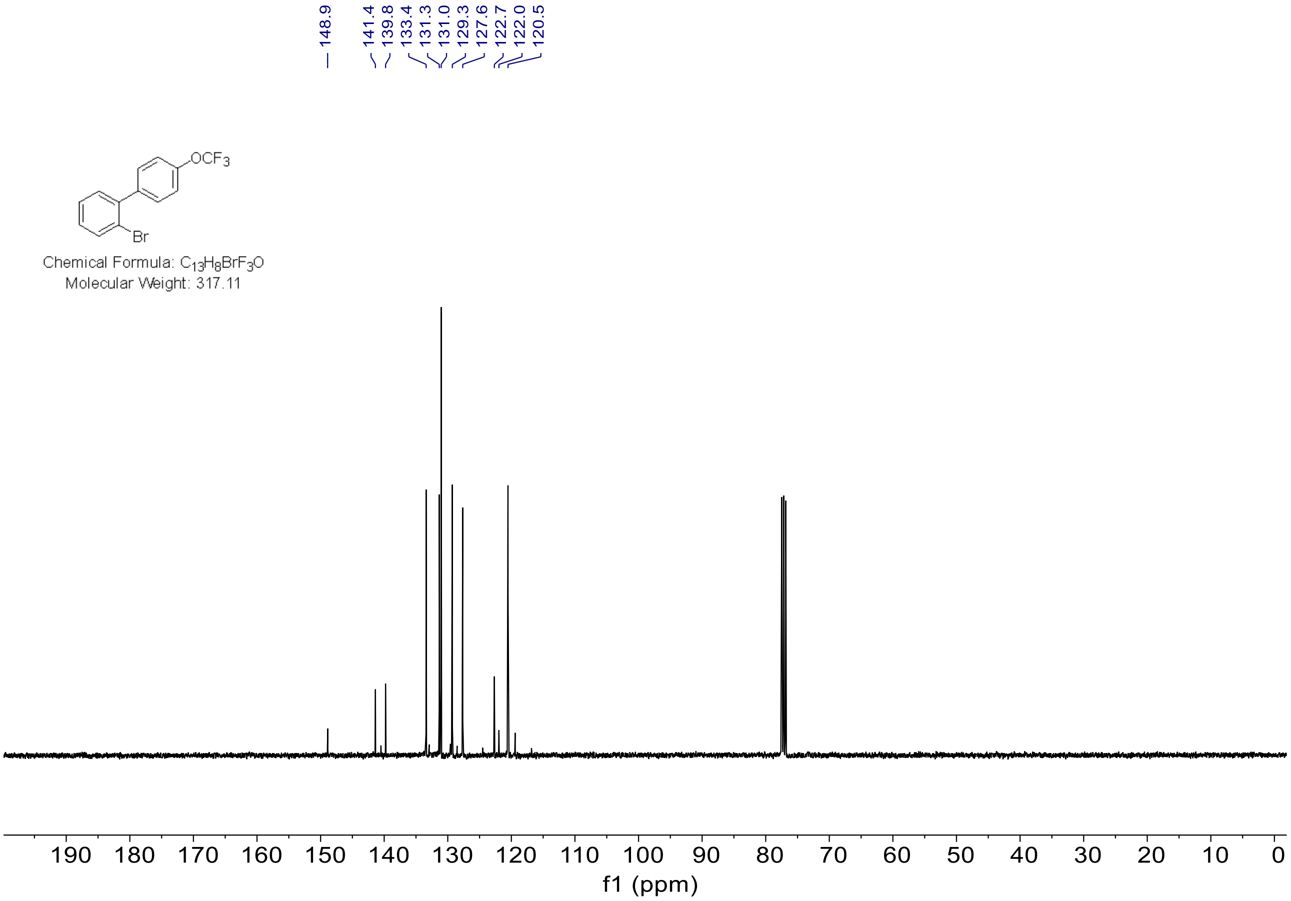

Bromo-2′-methyl-1,1′-biphenyl

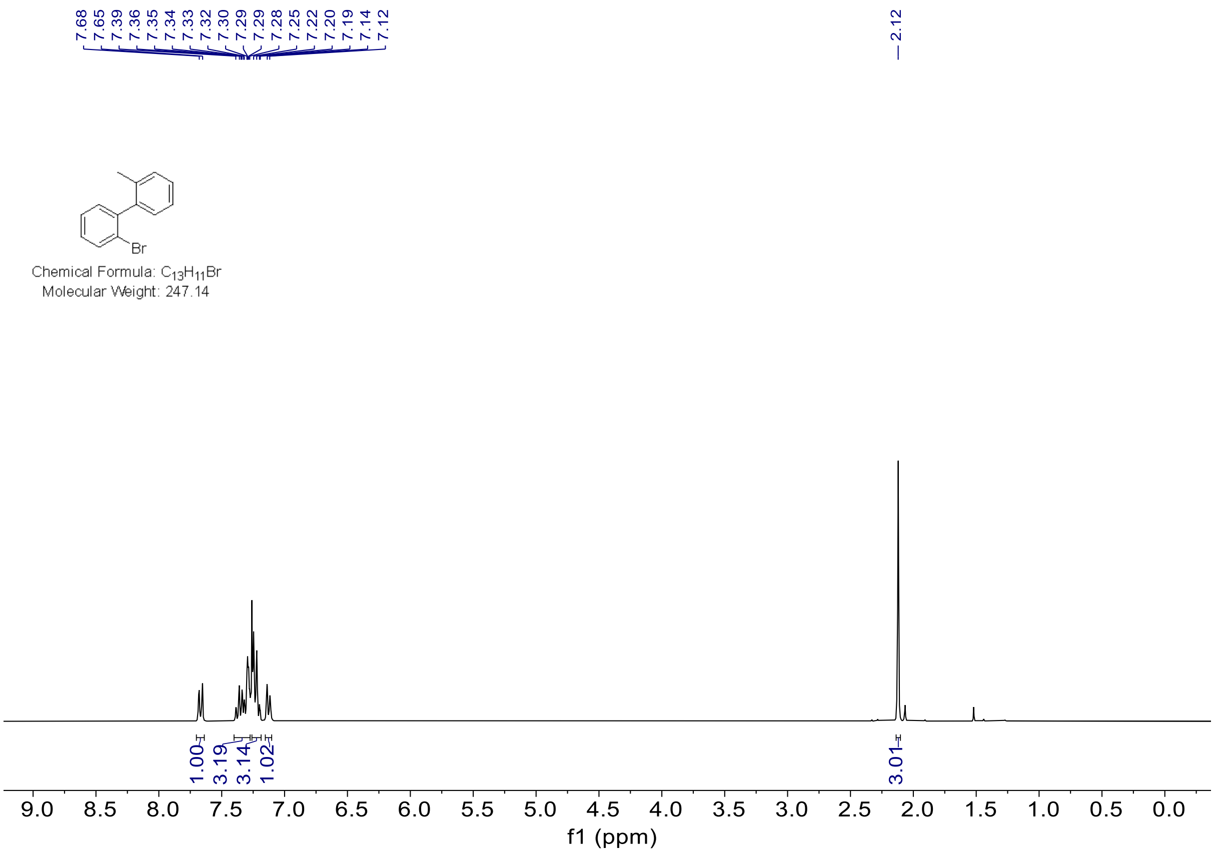

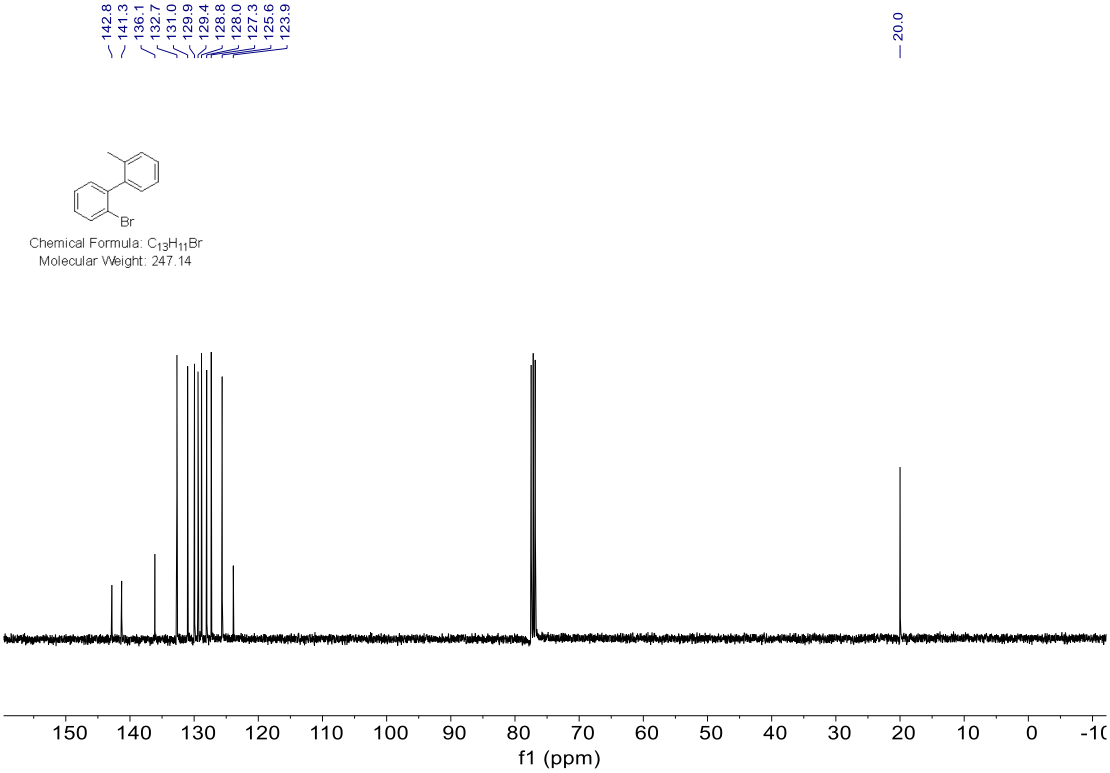

2-Bromo-2'-methoxyl-1,1'-biphenyl

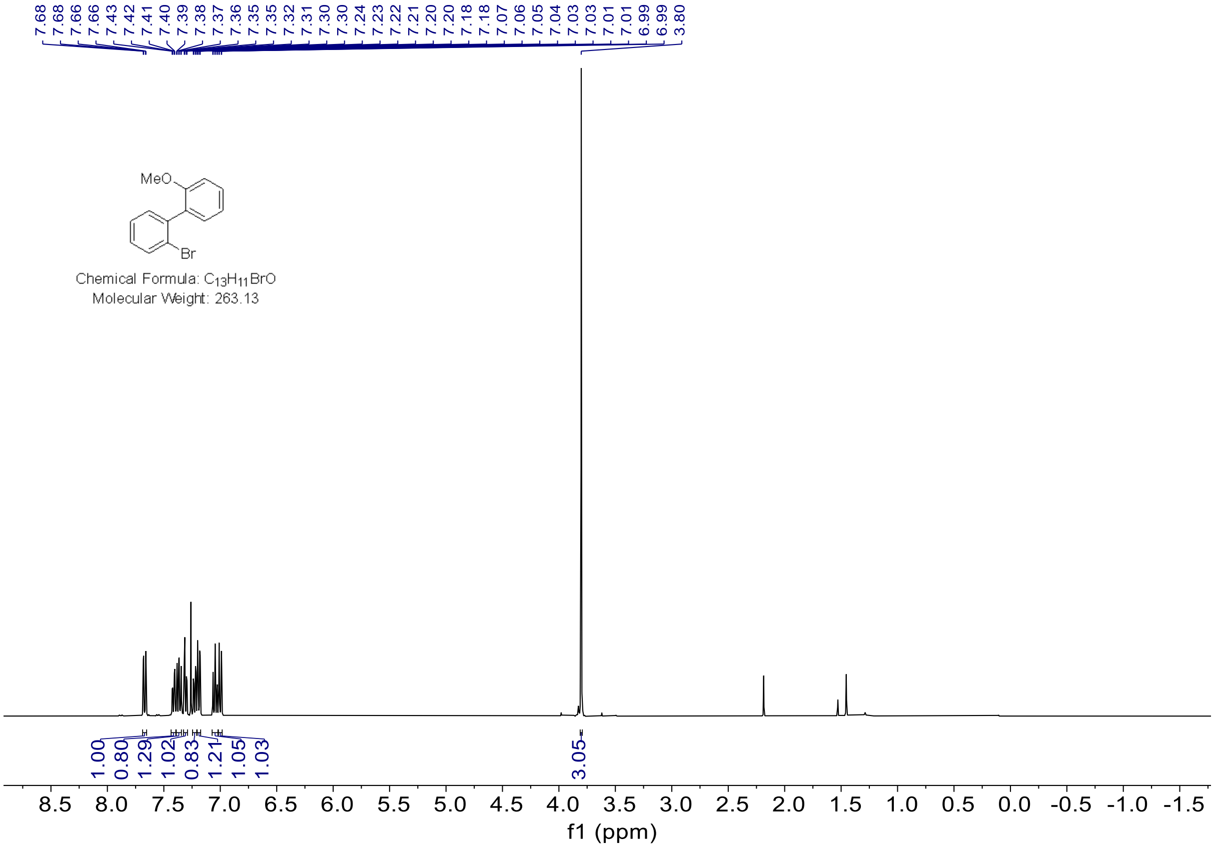

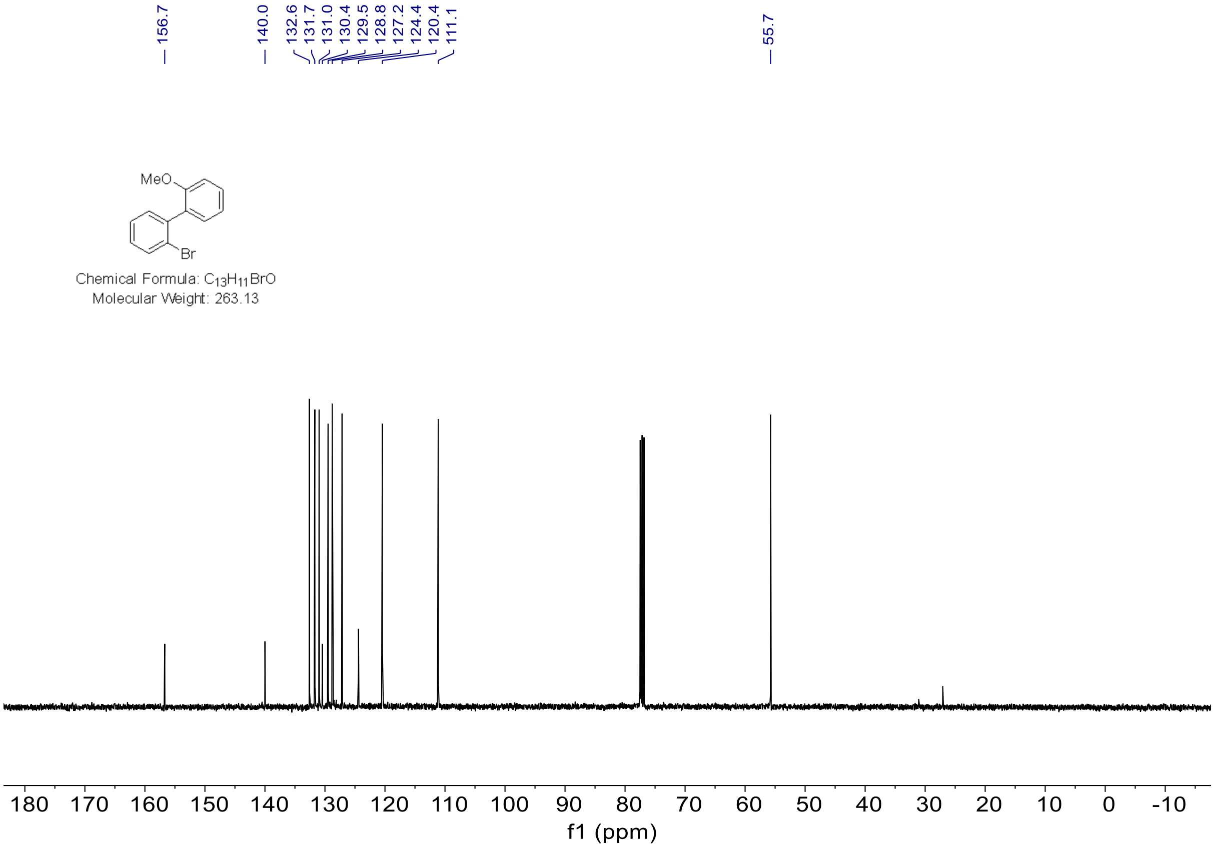

2-Bromo-2'-ethyl-1,1'-biphenyl

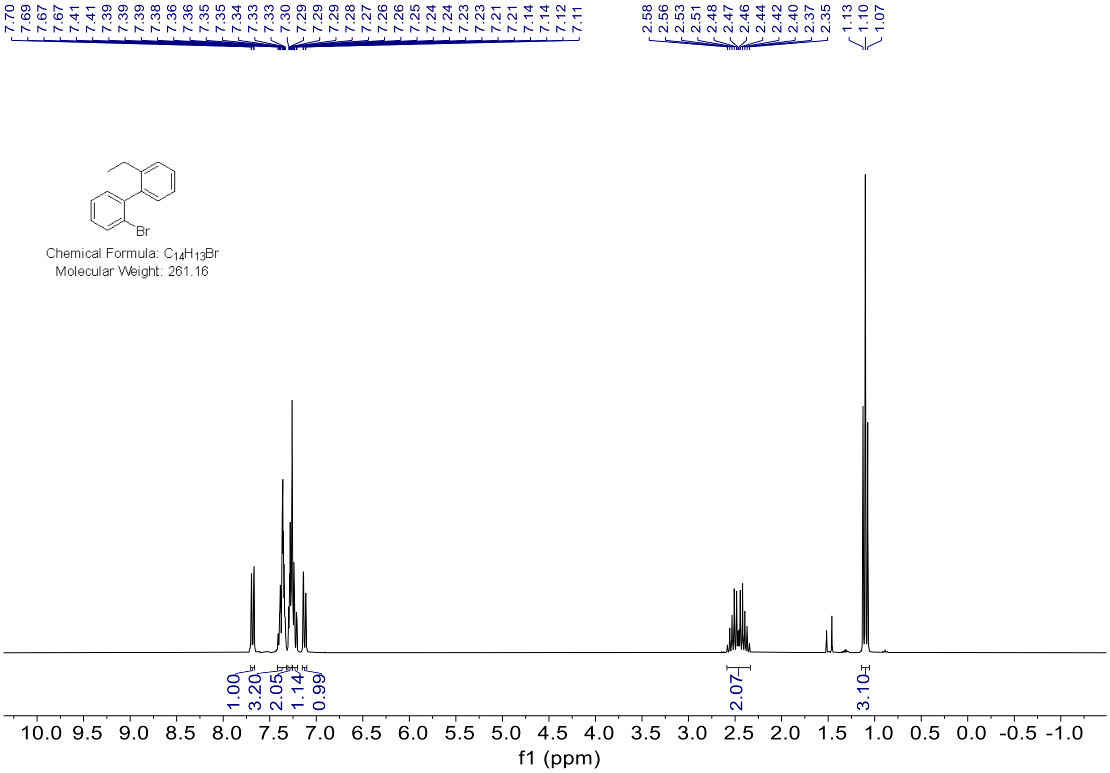

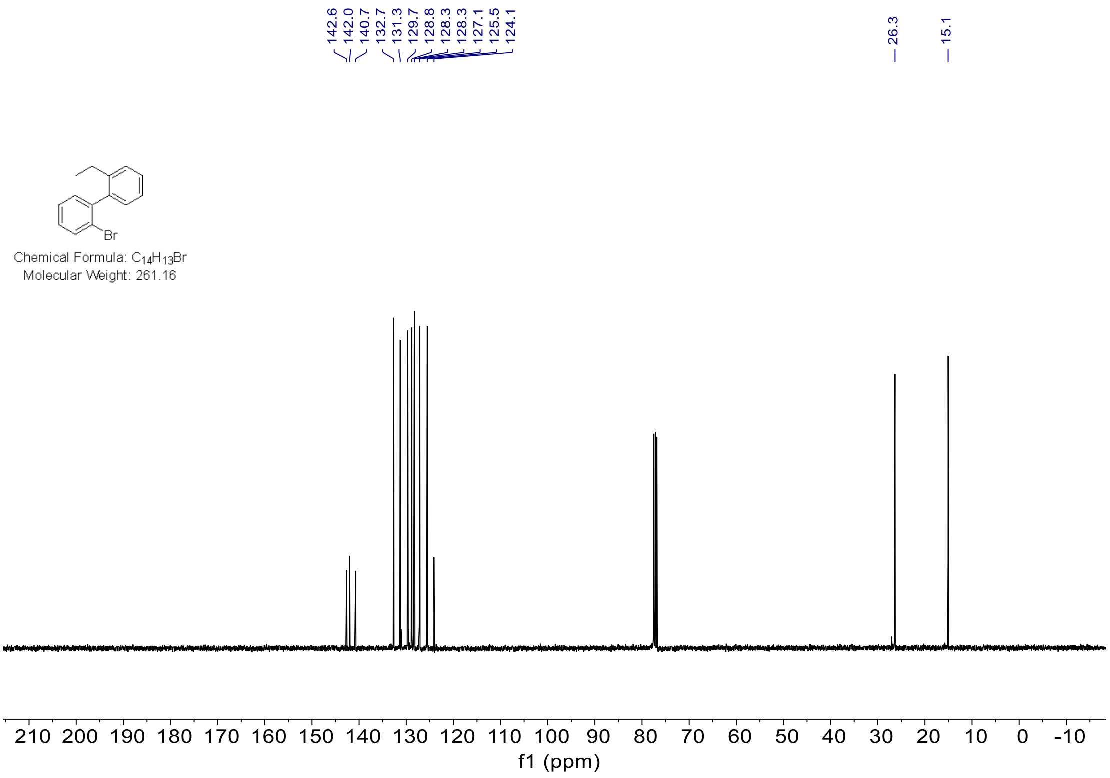

2-Bromo-3′-methyl-1,1′-biphenyl

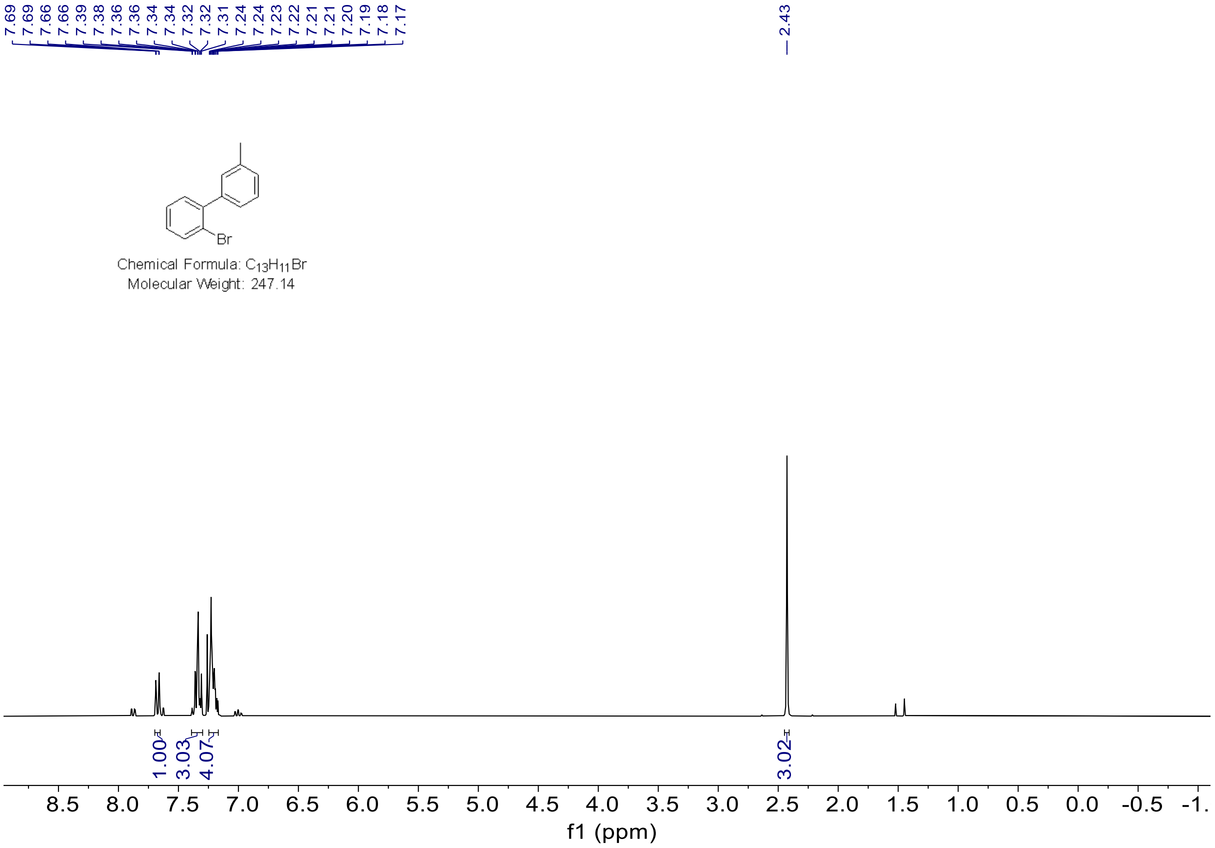

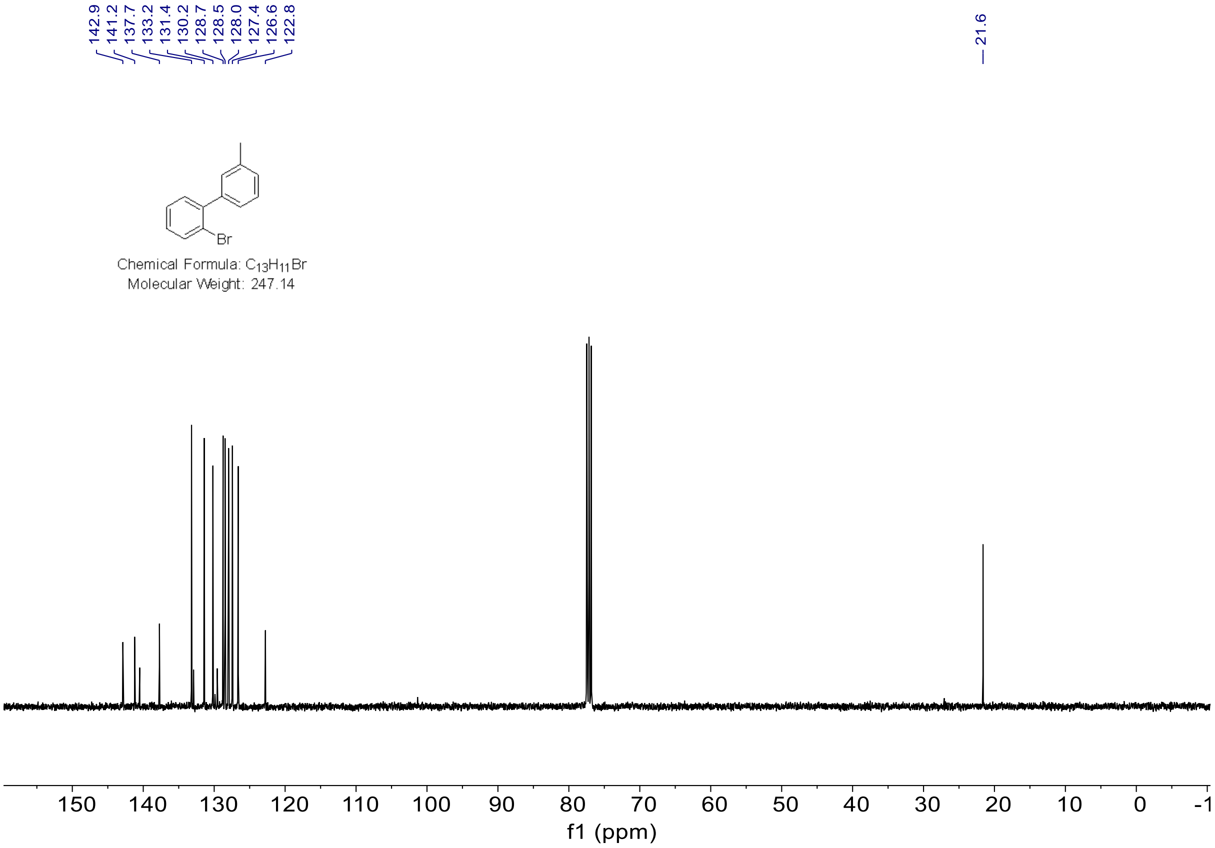

2-Bromo-3′,5′-bis(trifluoromethyl)-1,1′-biphenyl

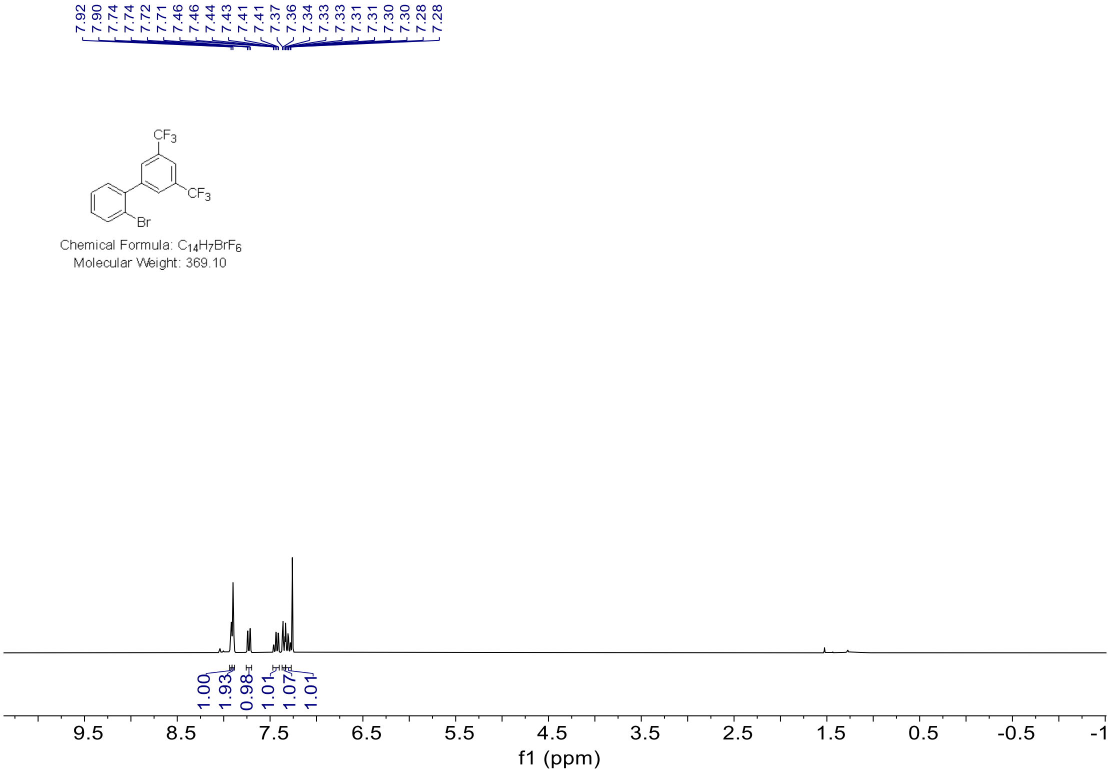

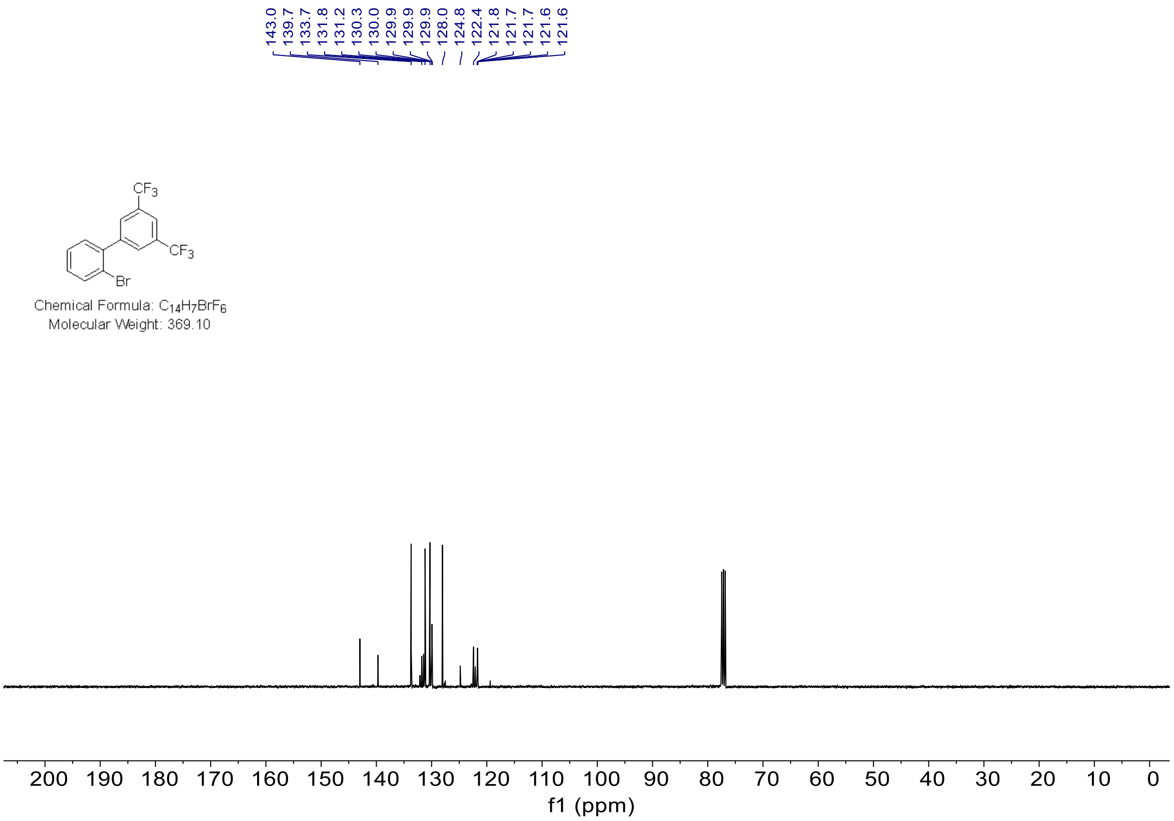

2-(2'-Bromophenyl) naphthalene

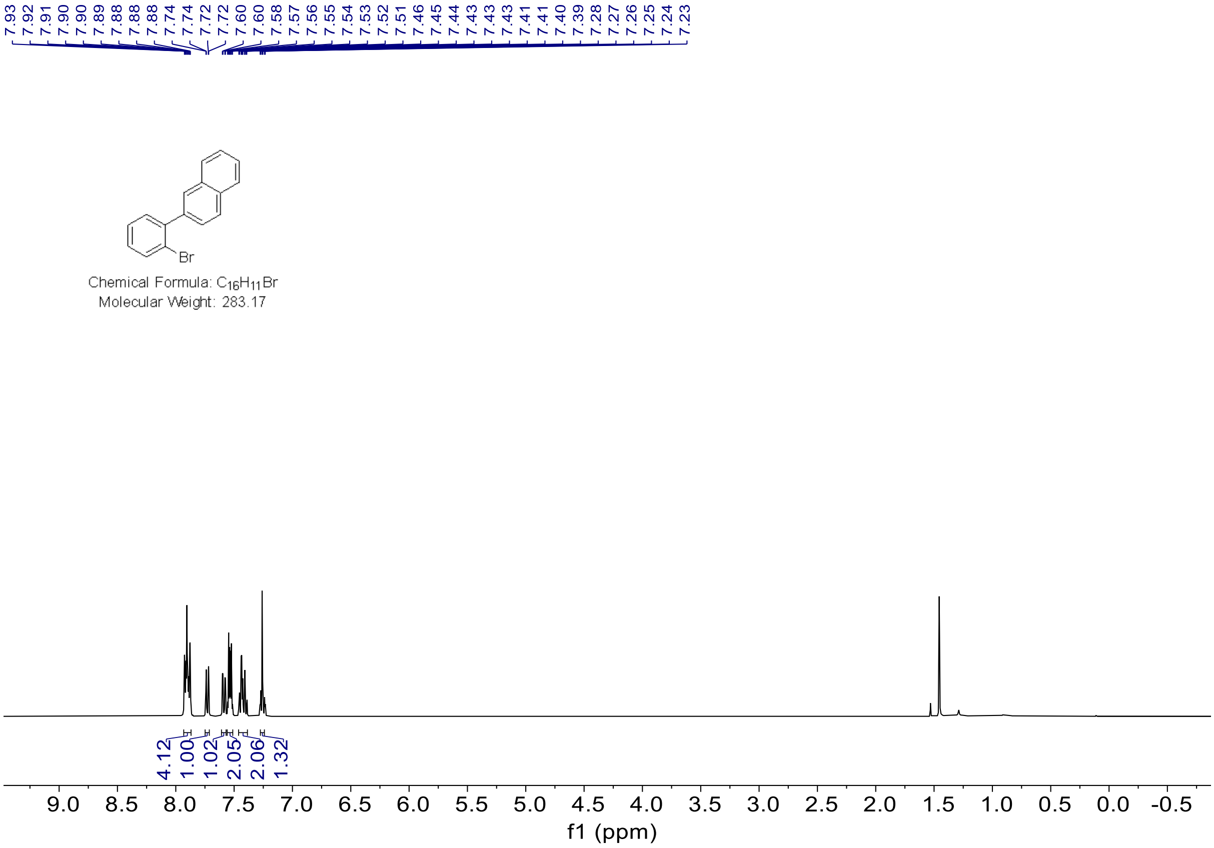

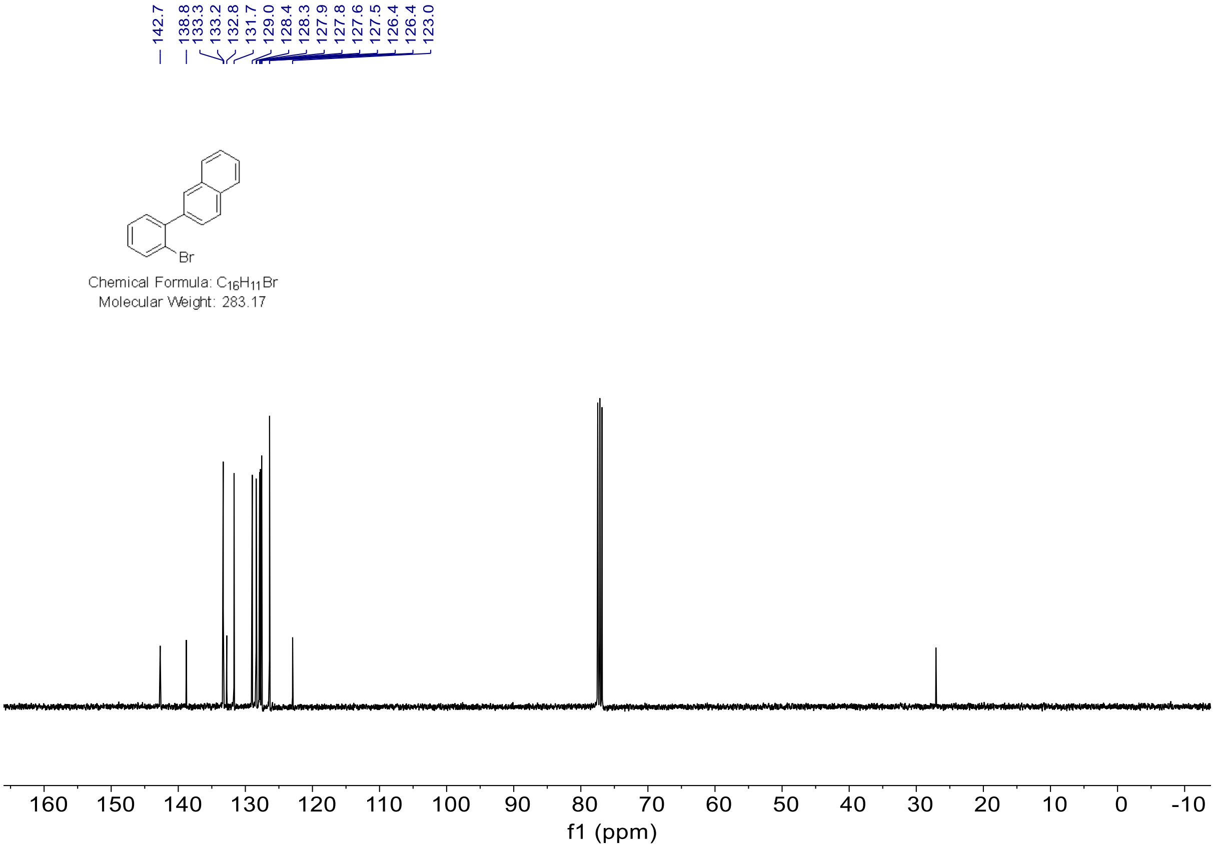

2-(2-Bromophenyl)-6-methoxynaphthalene

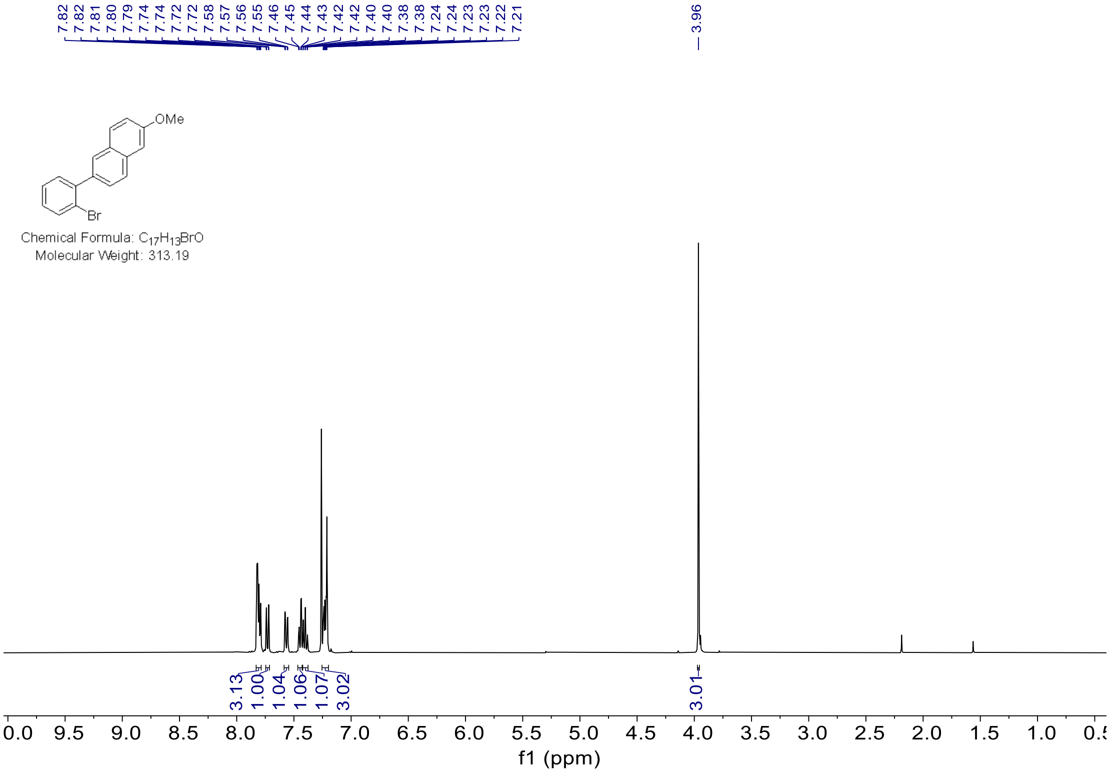

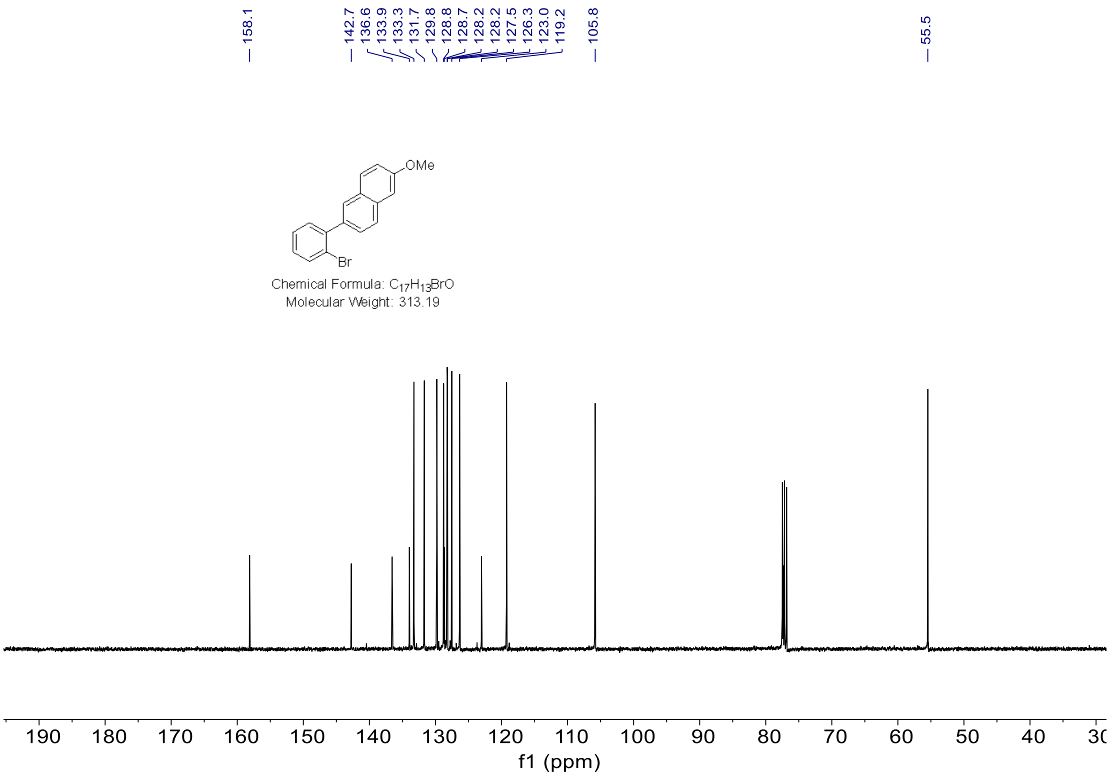

(2-Bromophenyl)ferrocene.

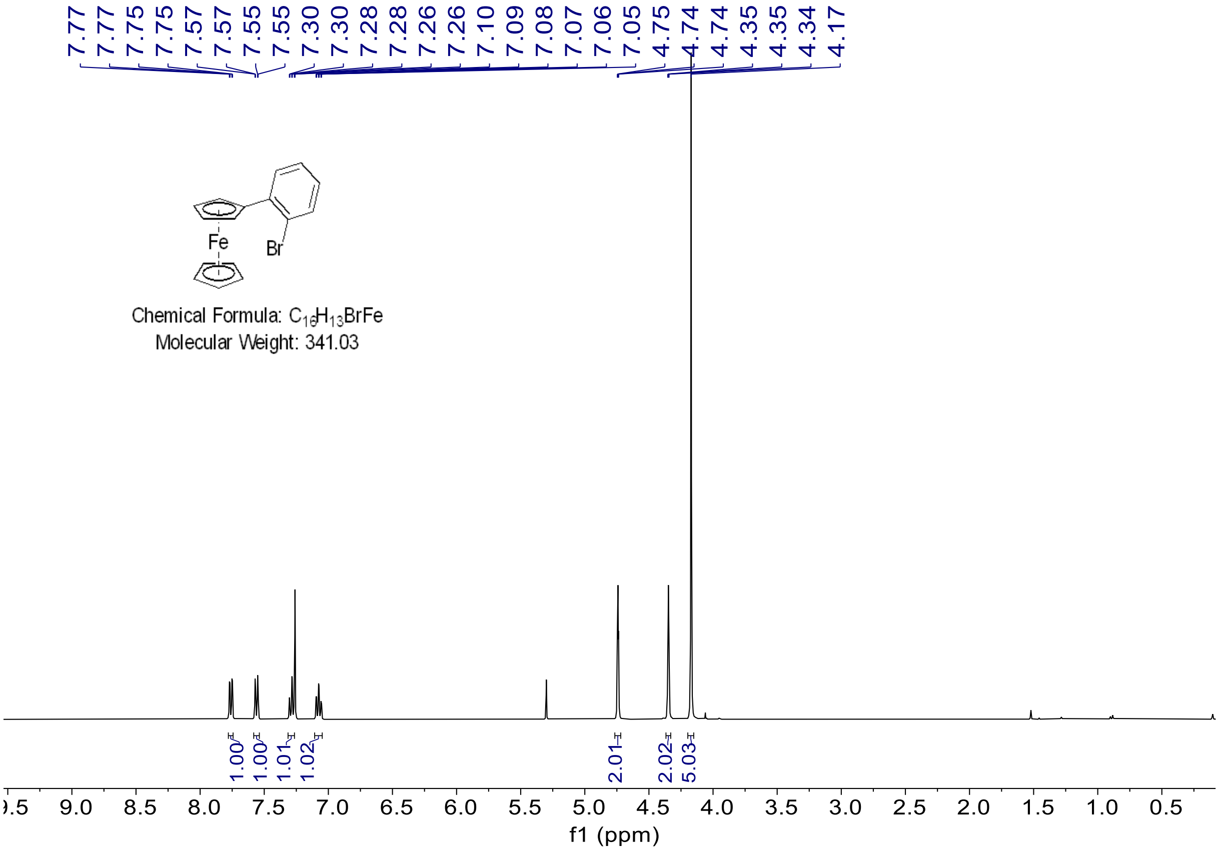

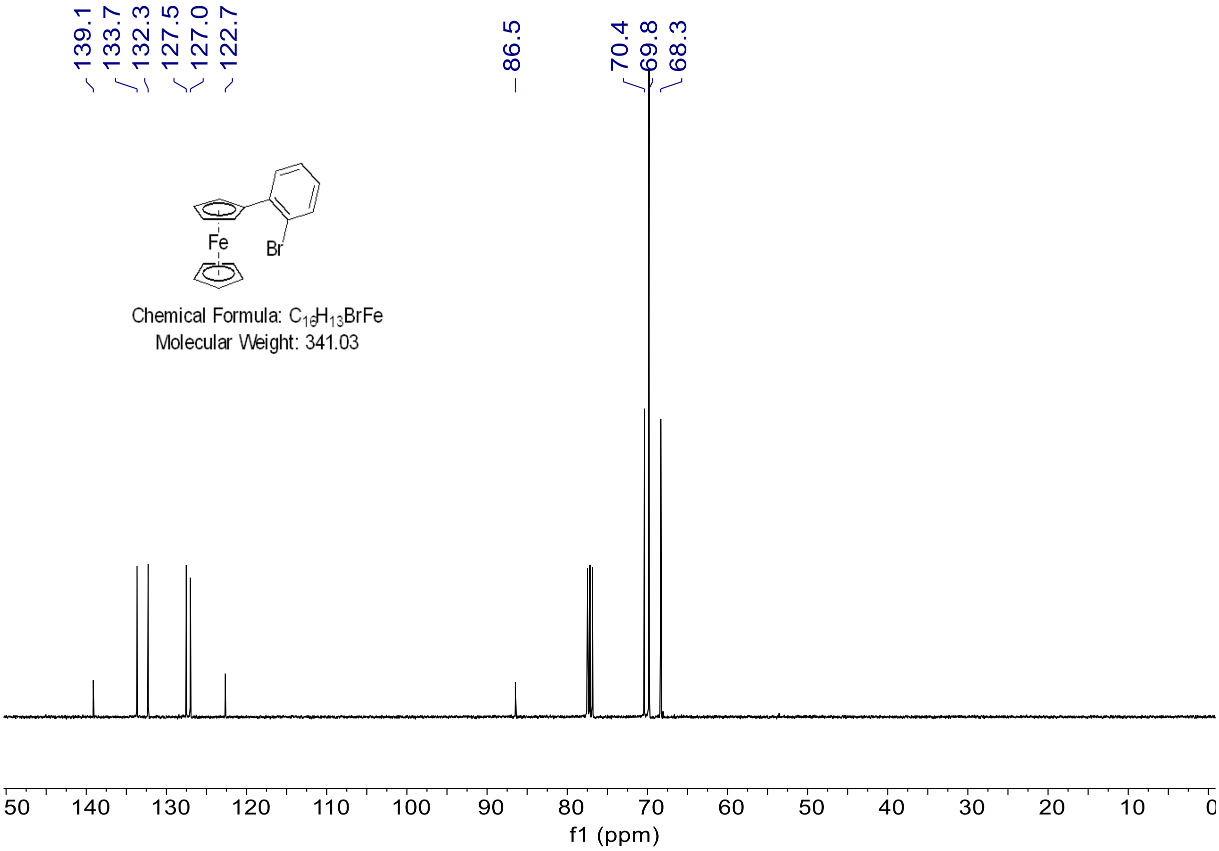

2,2'''-Dibromo-1,1':4',1'':4'',1'''-quaterphenyl

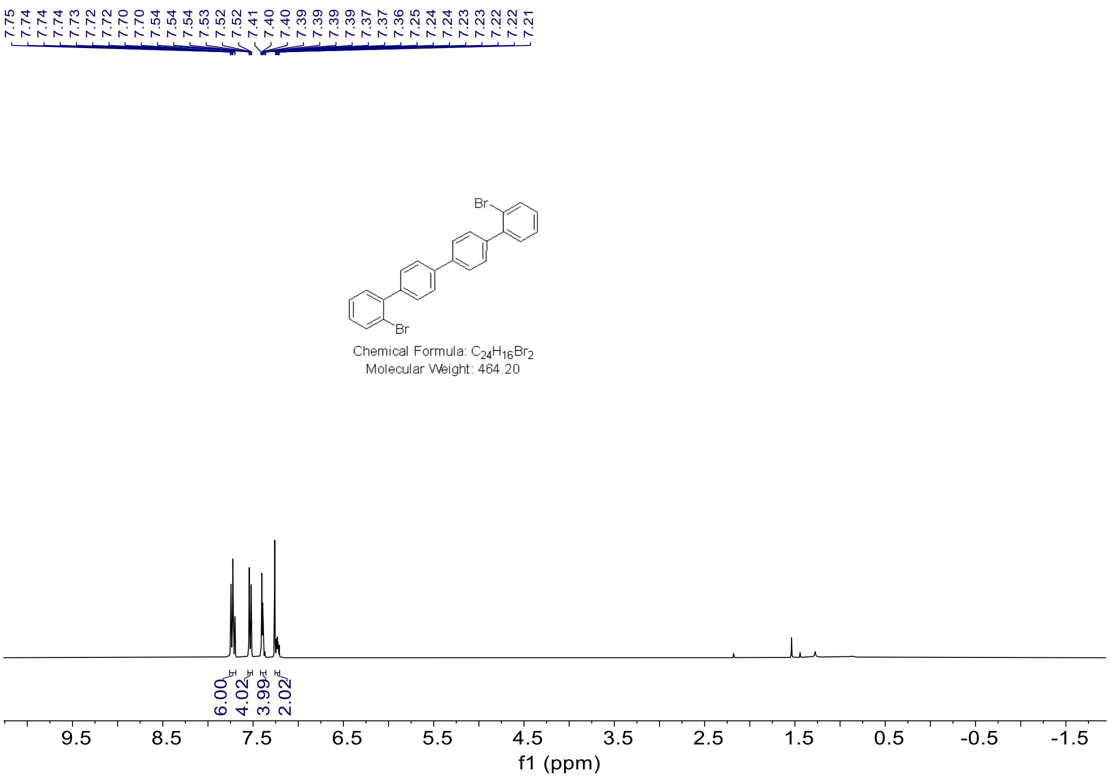

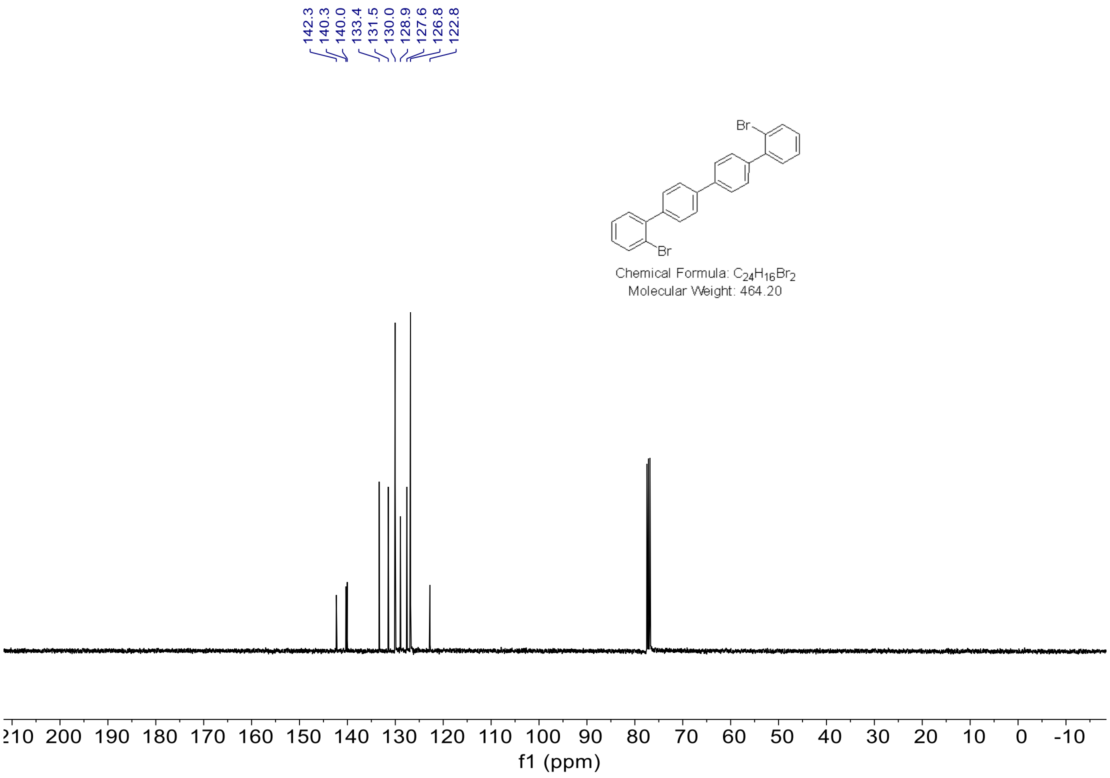

2,2''-Dibromo-1,1':4',1''-terphenyl

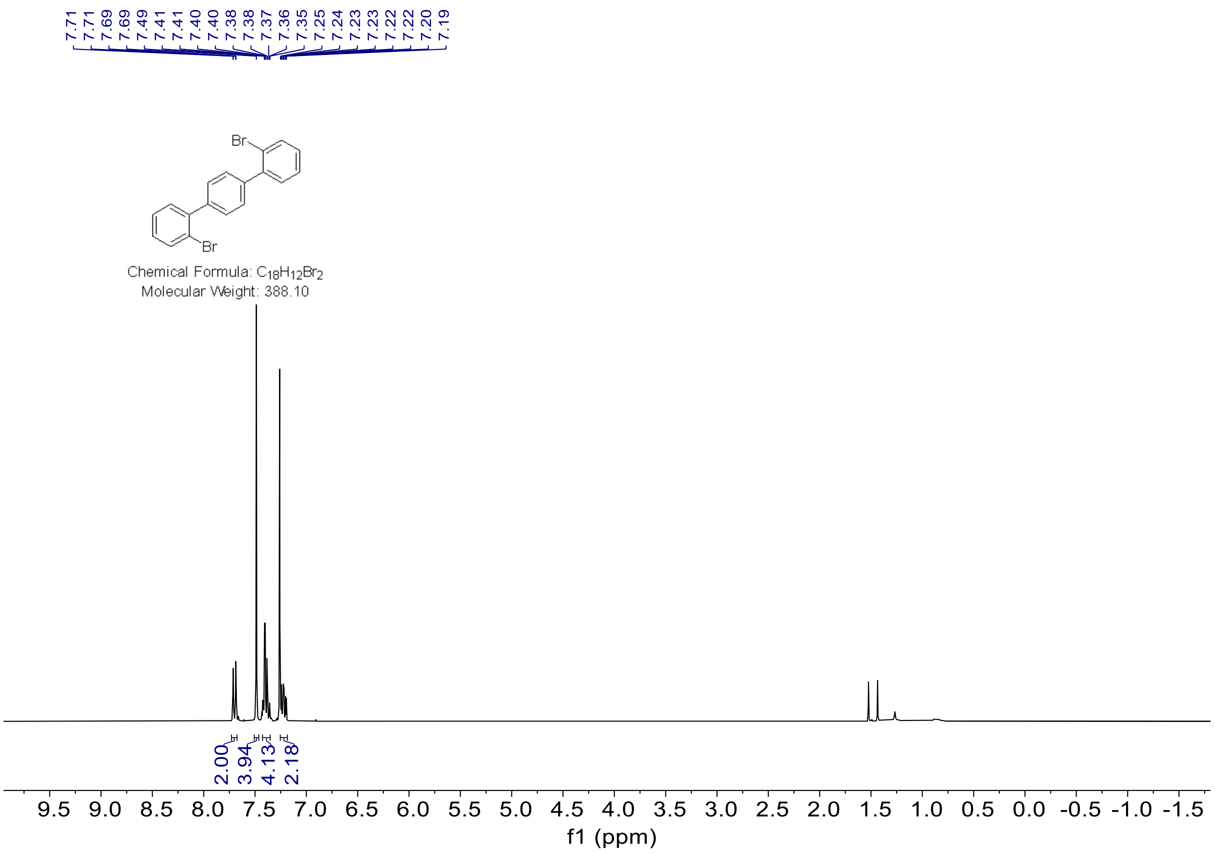

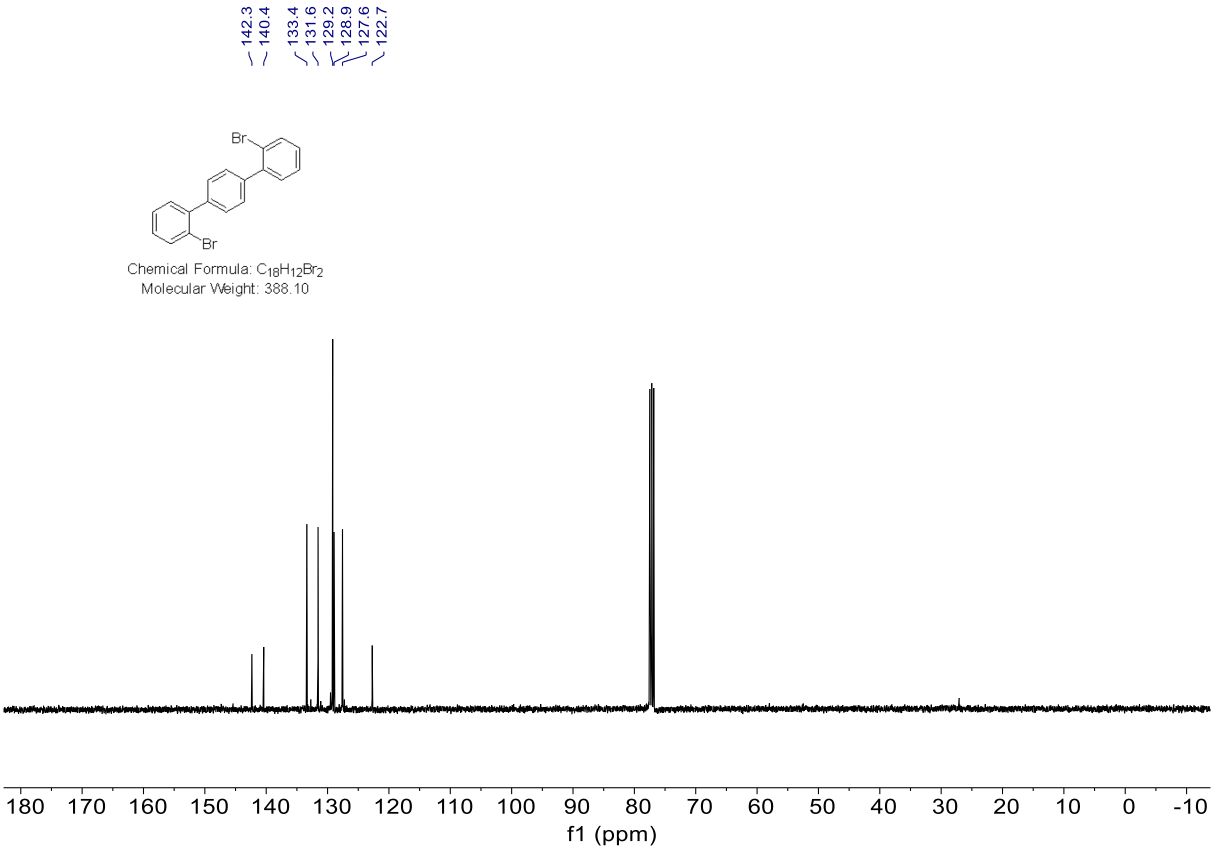

2-(Dimethylsilyl) biphenyl (5a)

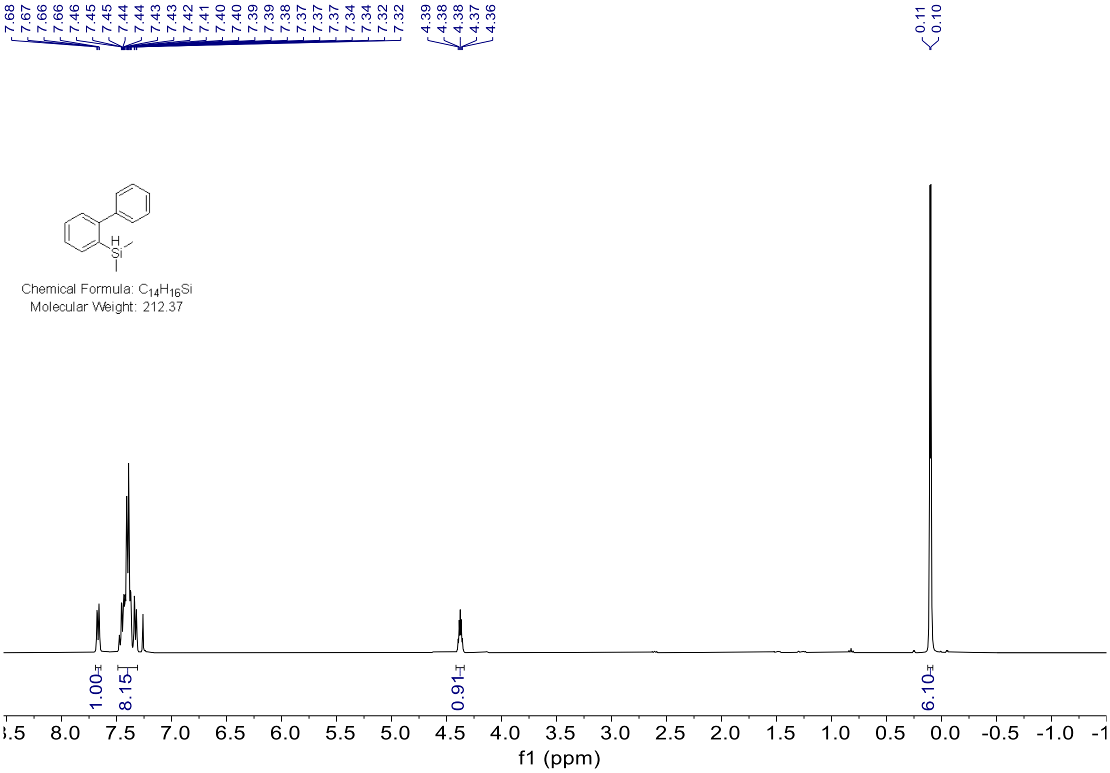

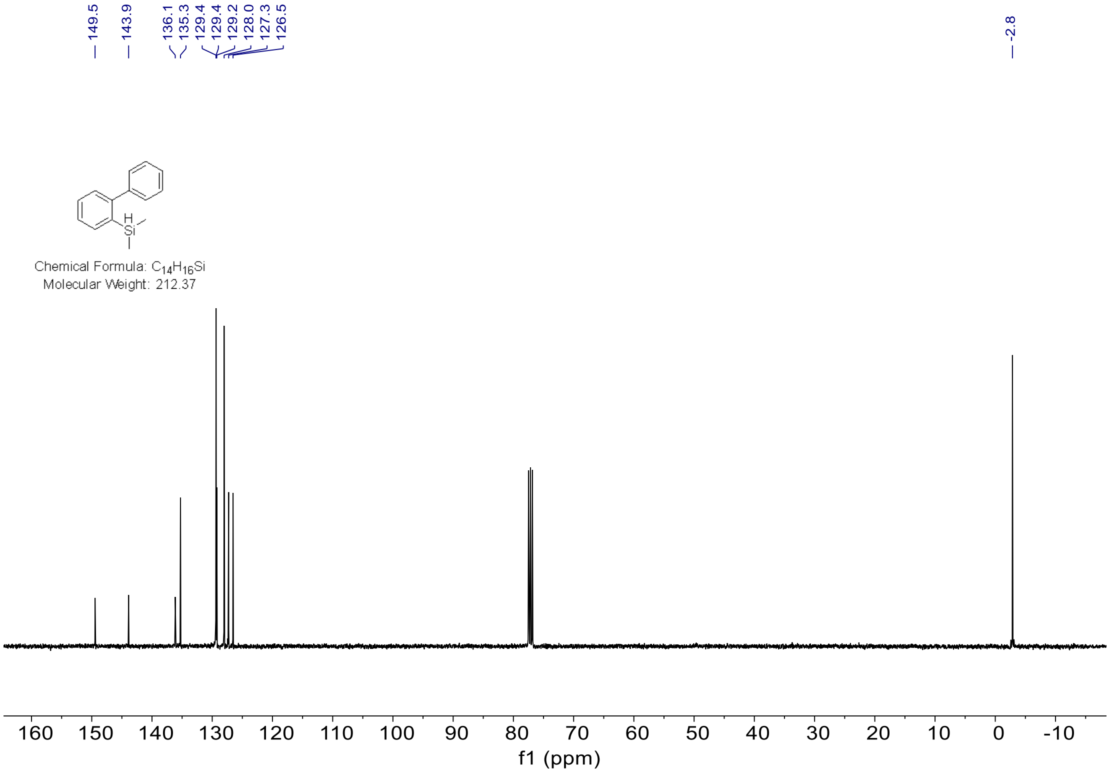

[1,1':4',1''-Terphenyl]-2-yldimethylsilane (5b)

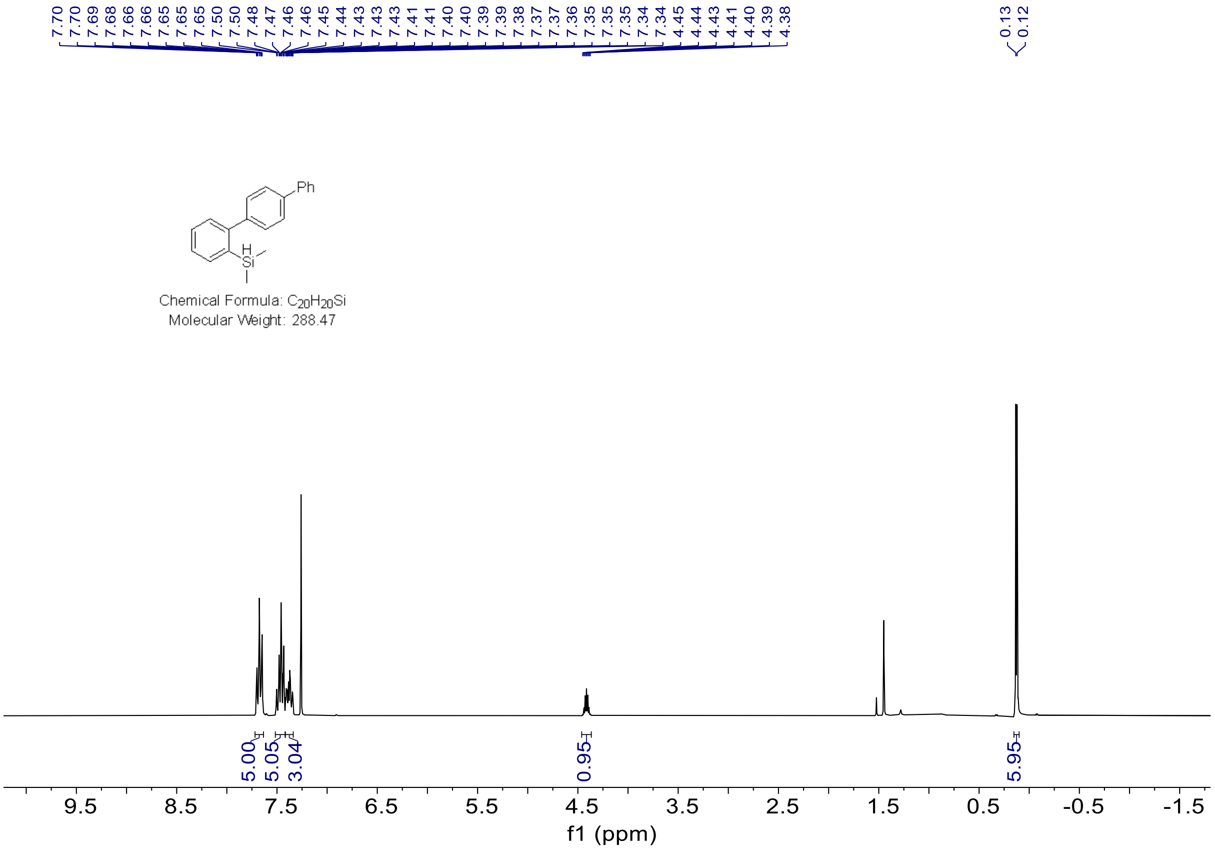

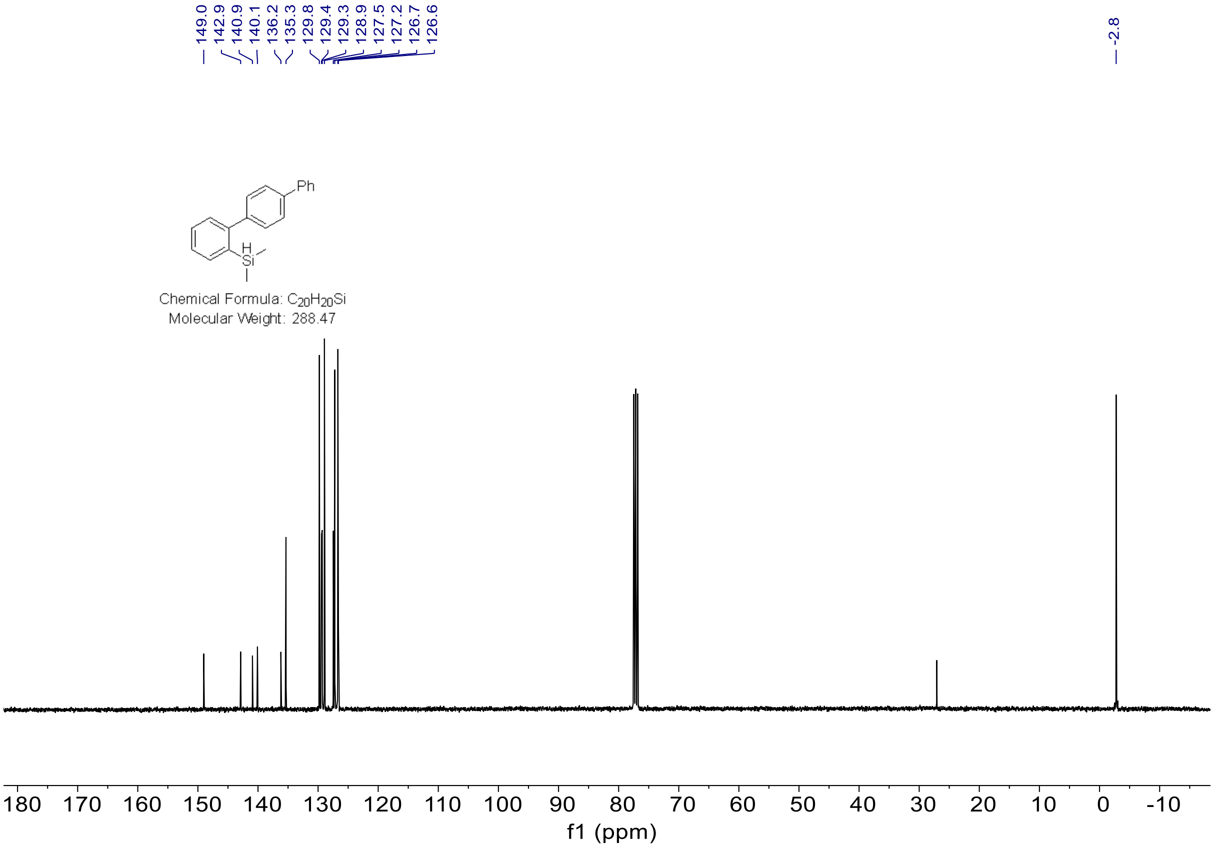

2-(Dimethylsilyl)-4'-methylbiphenyl (5c).

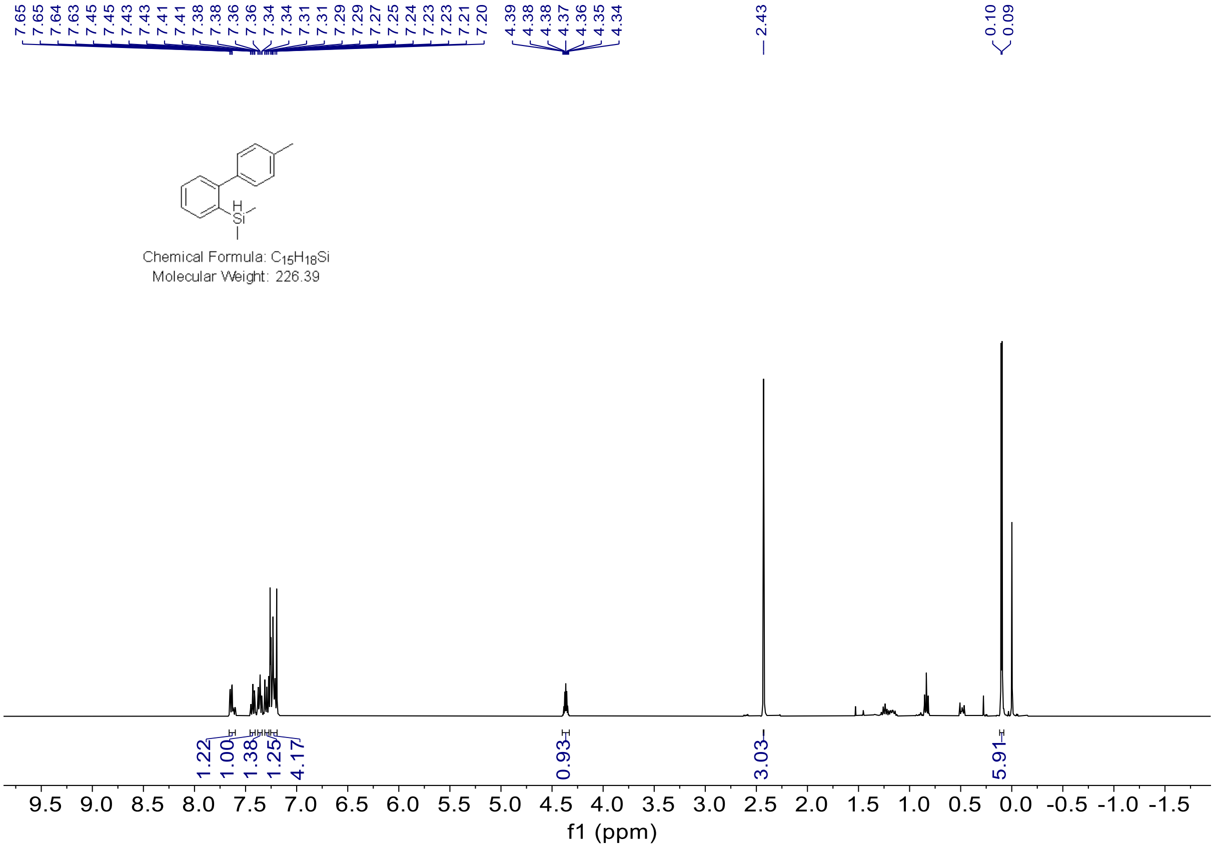

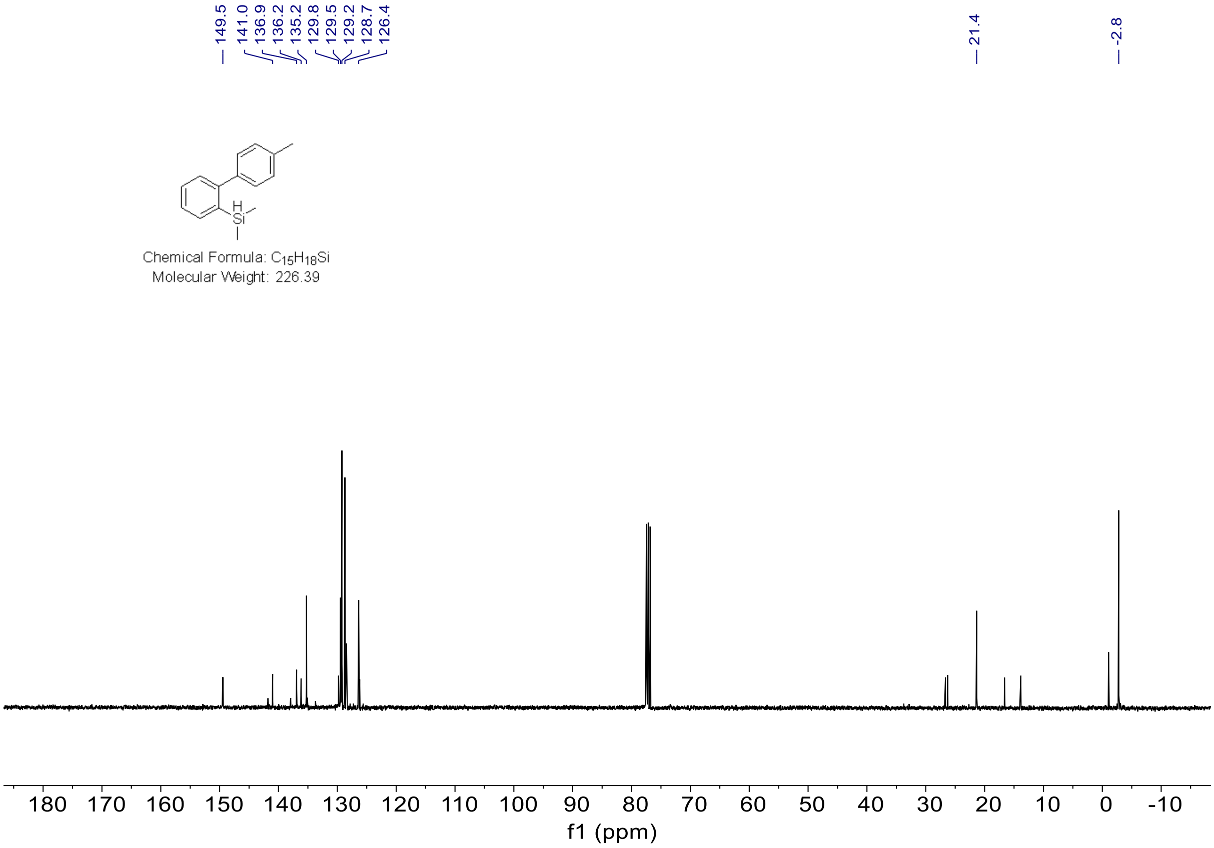

2-(Dimethylsilyl)-4'-isopropylbiphenyl (5d).

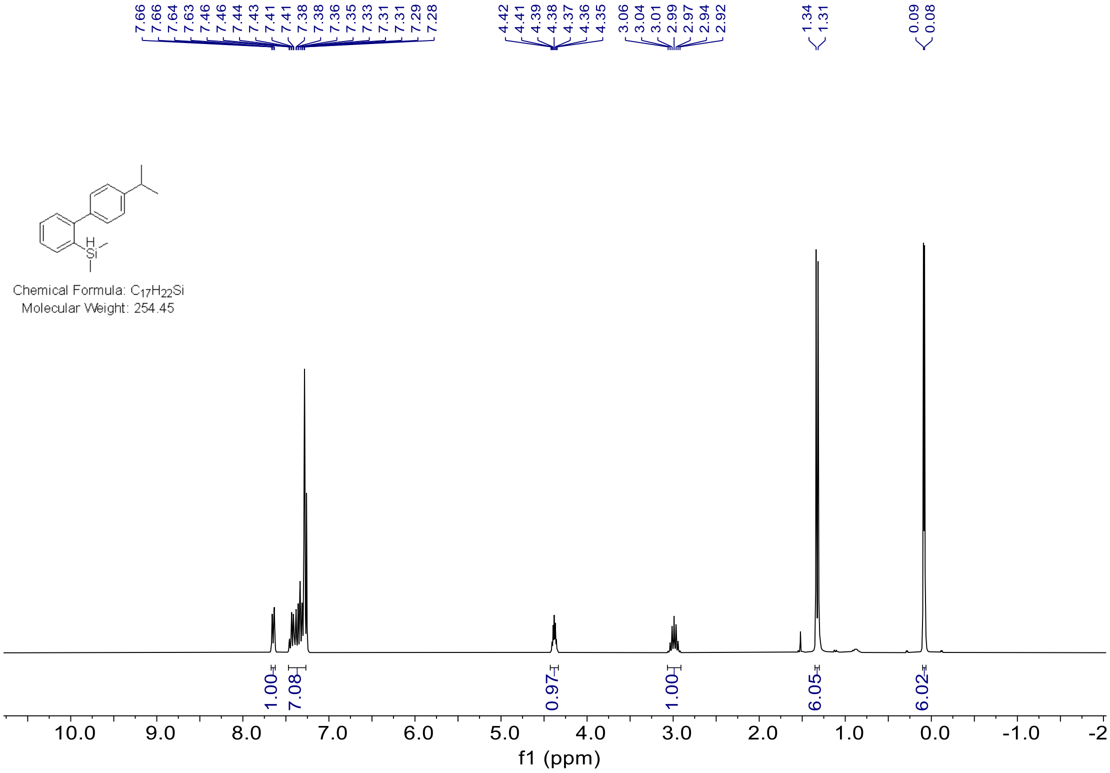

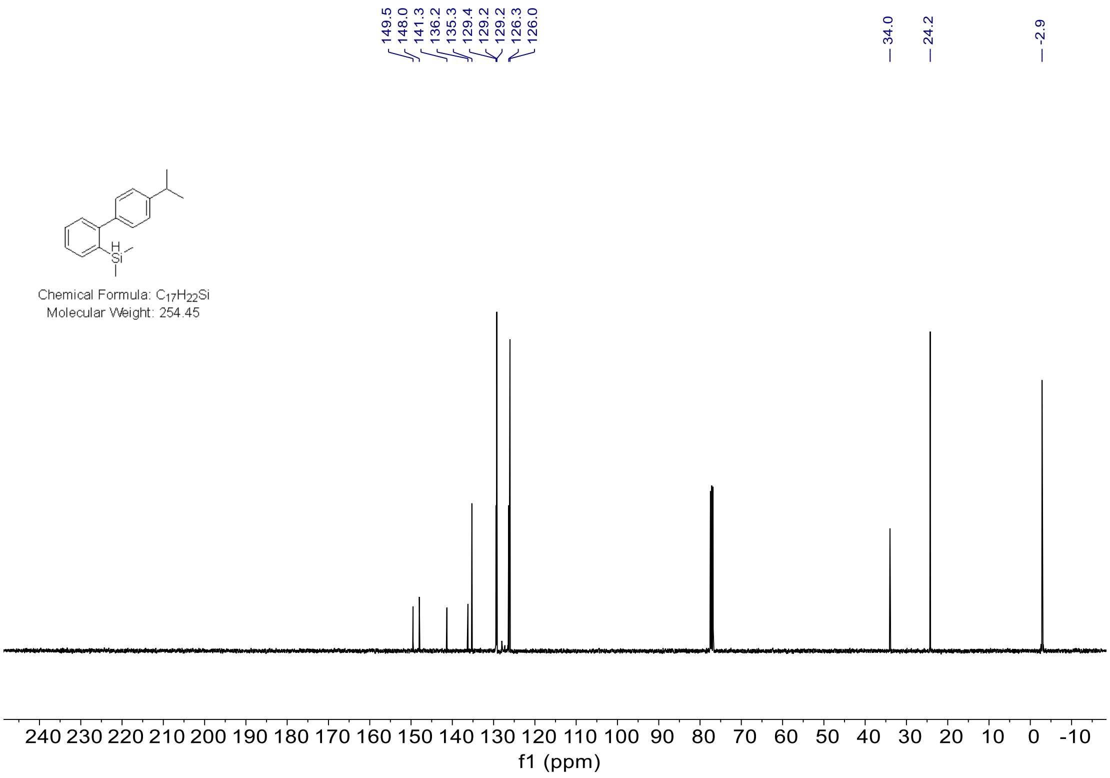

2-(Dimethylsilyl)-4'-*tert*-butylbiphenyl (5e).

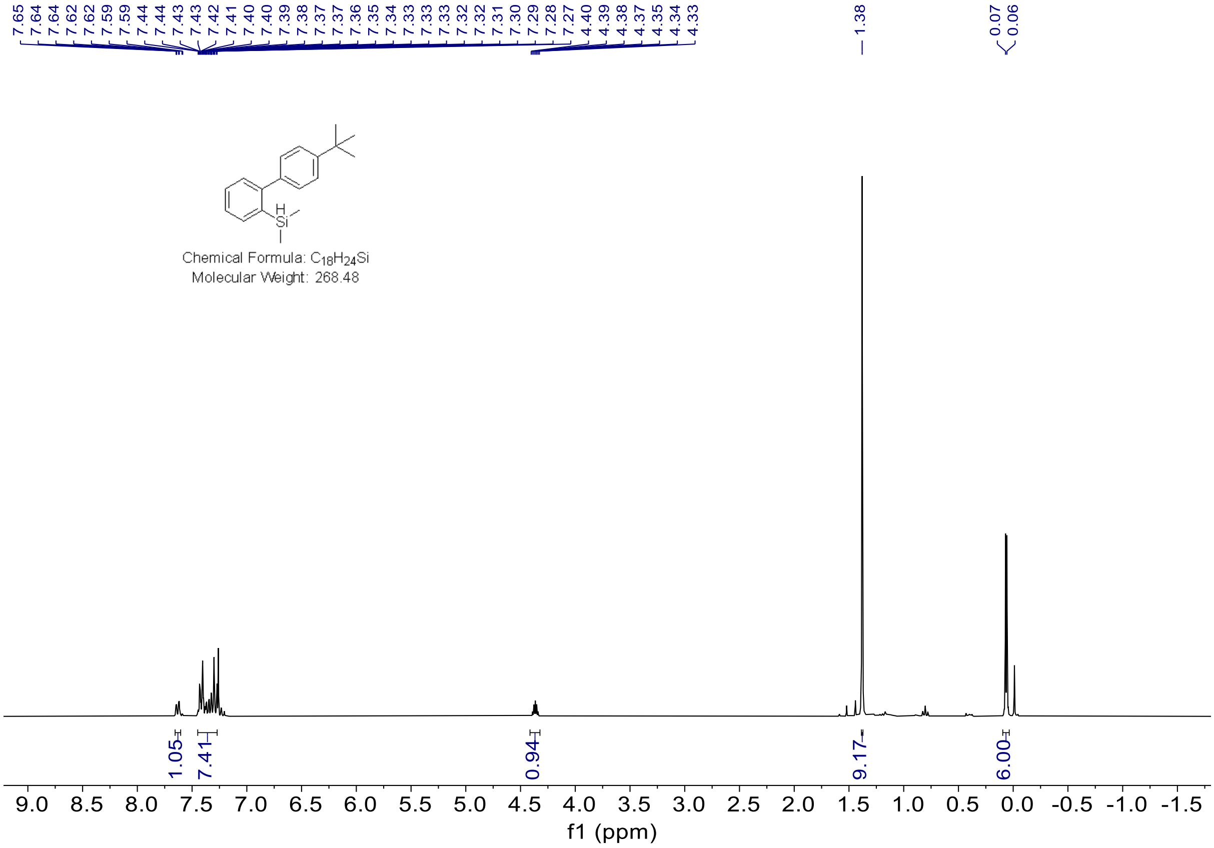

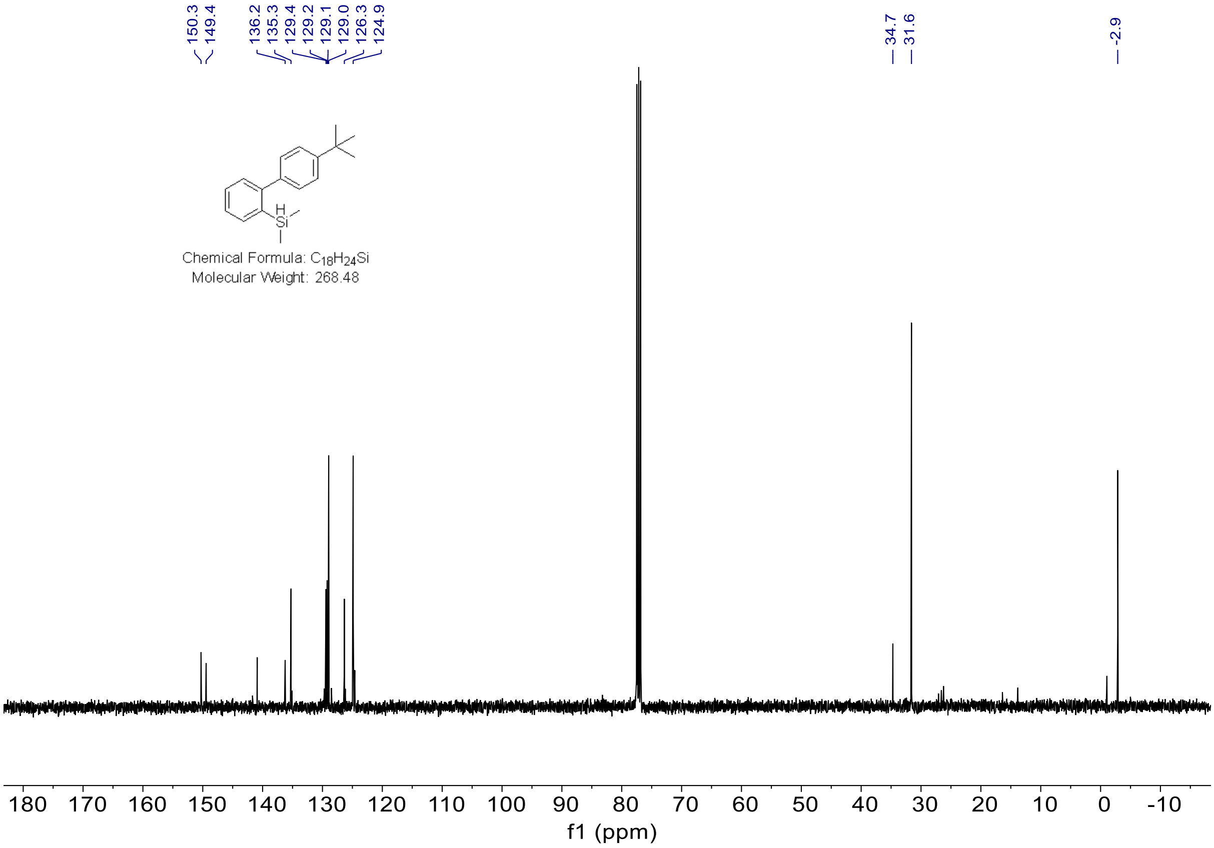

2-(Dimethylsilyl)-4'-trifluoromethylbiphenyl (5f).

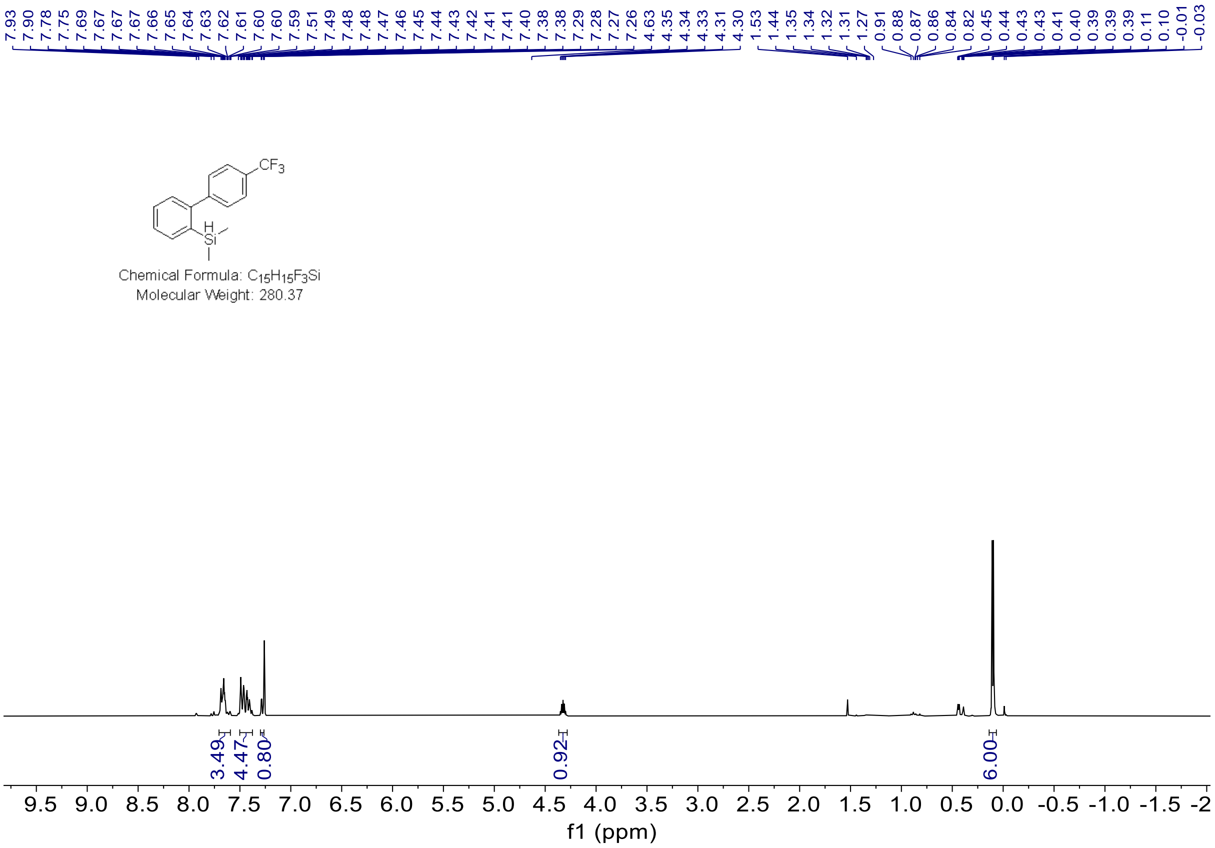

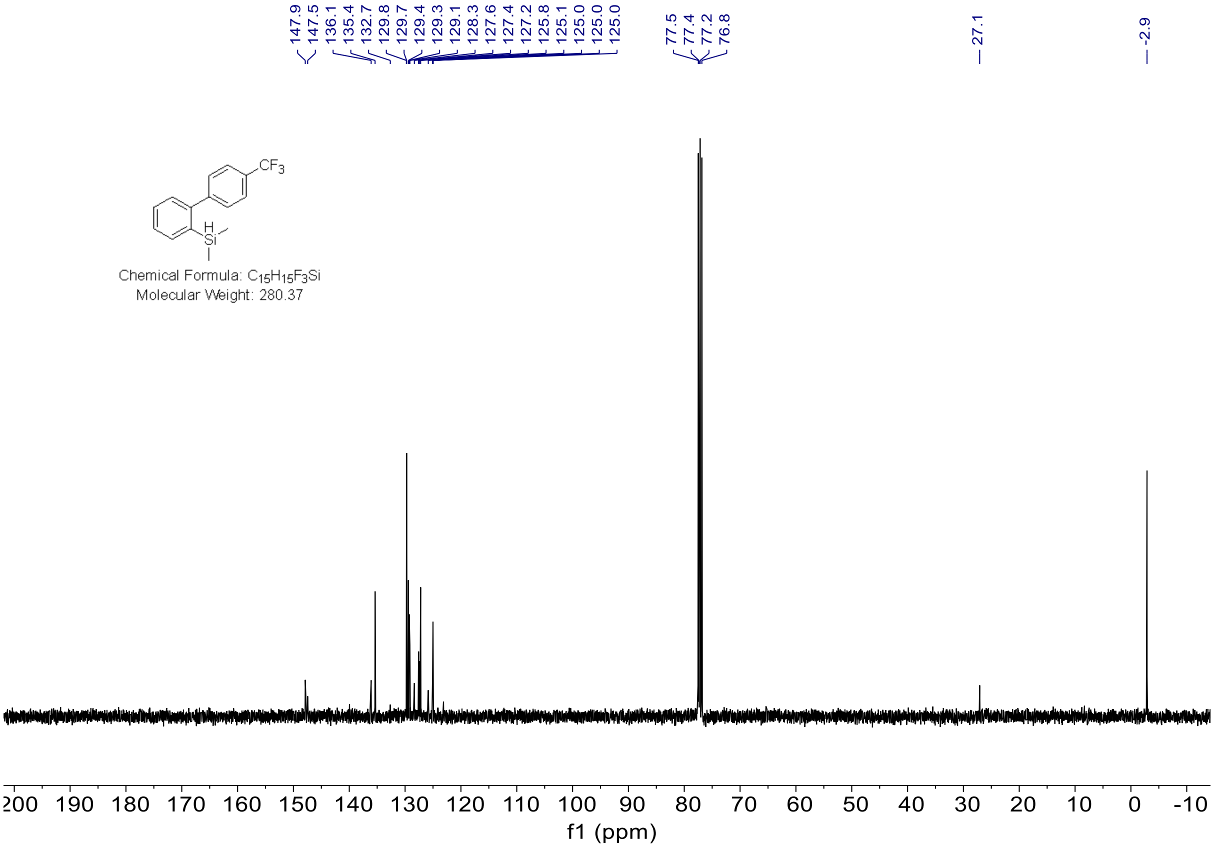

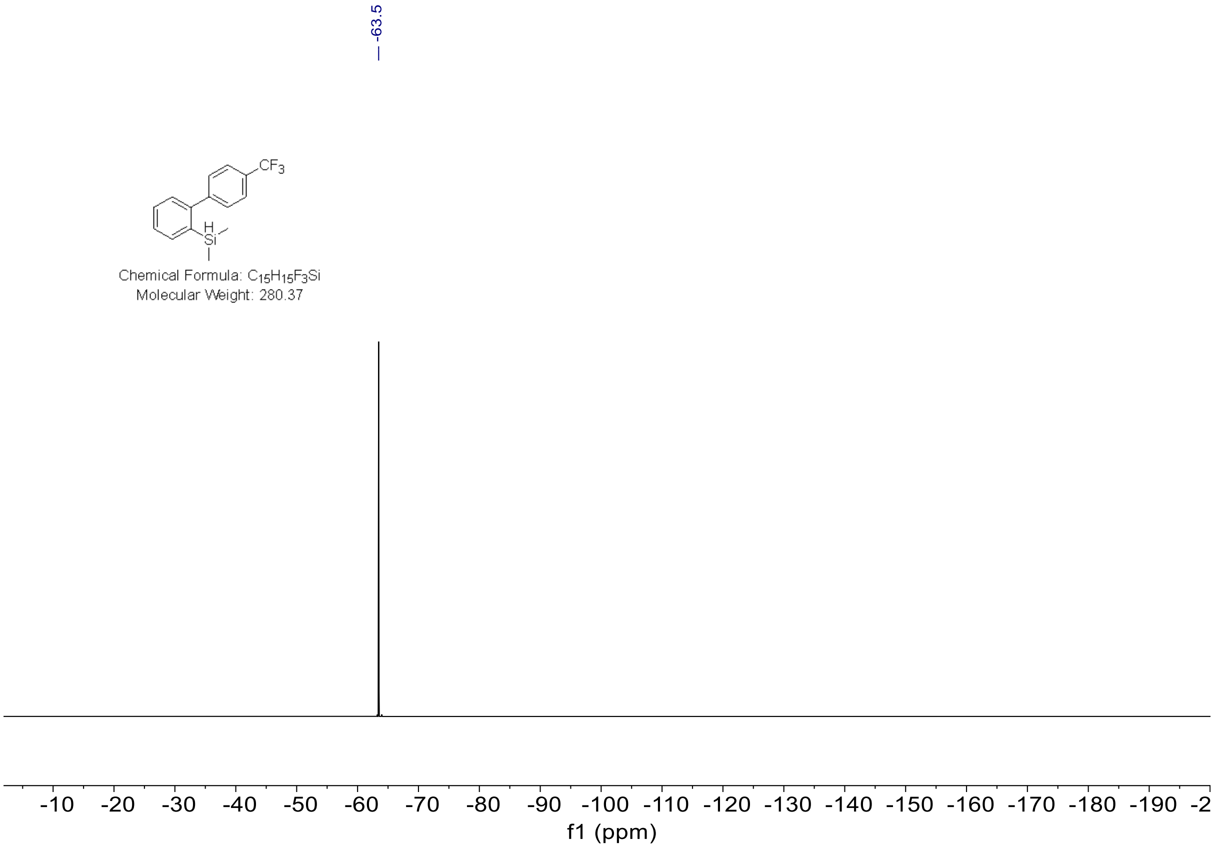

2-(Dimethylsilyl)-4'-fluorobiphenyl (5g)

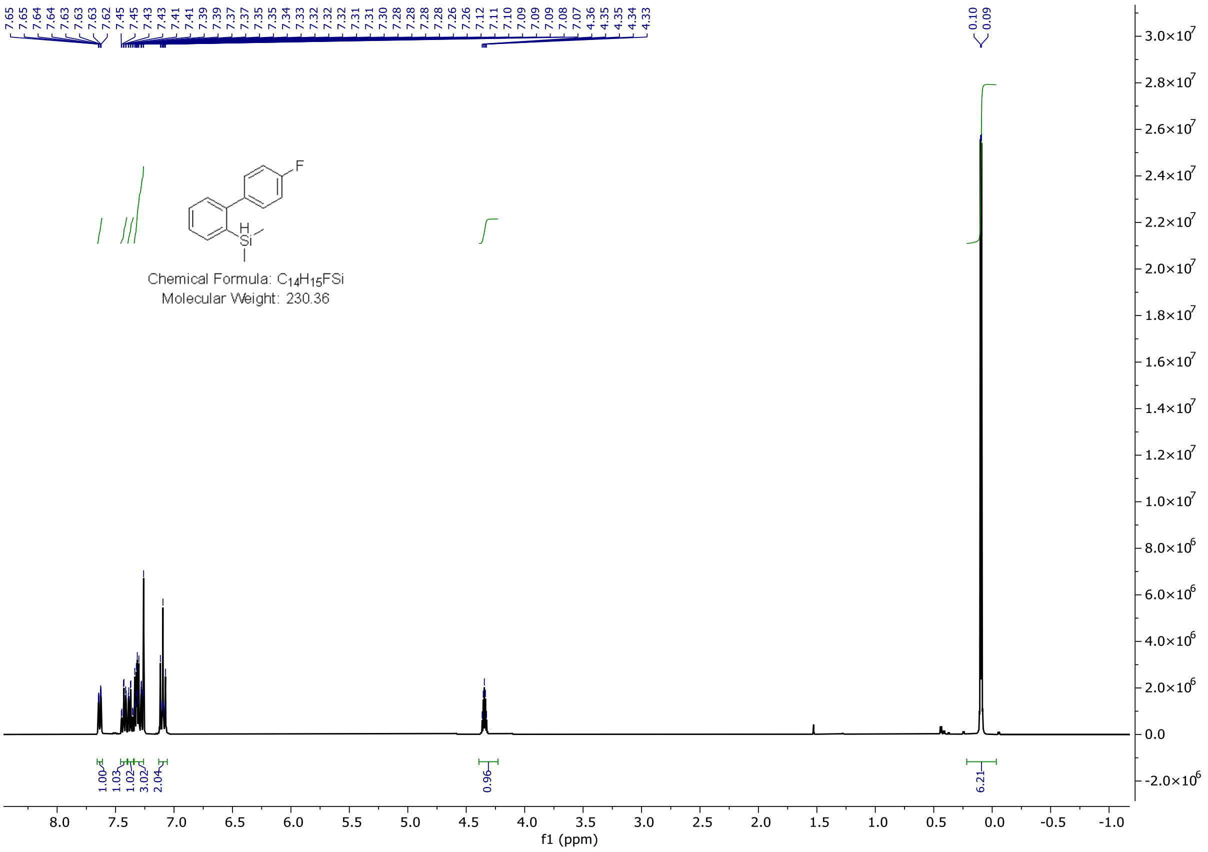

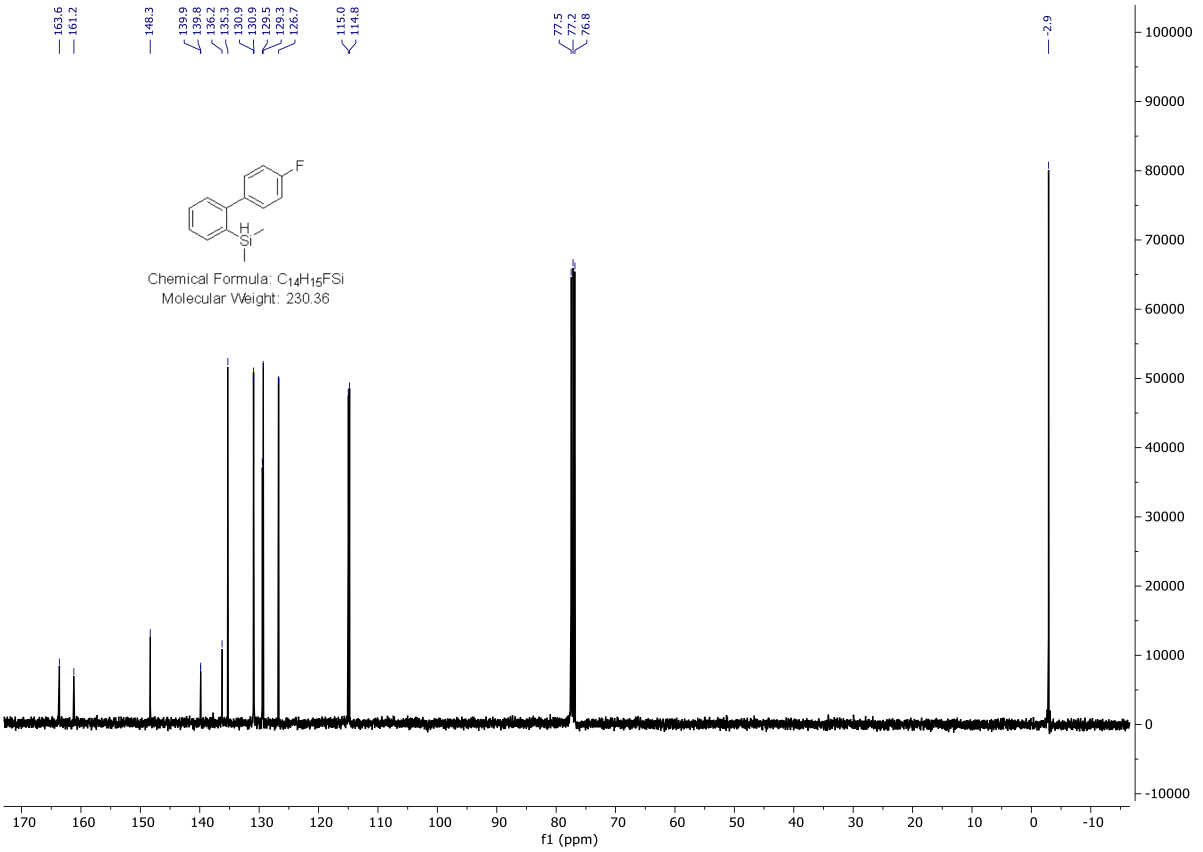
163.6
161.2
148.3
139.9
139.8
136.2
135.3
130.9
130.9
129.5
129.3
126.7
115.0
114.8
77.5
77.2
76.8
-2.9
F
H
Si
Chemical Formula: C14H15FSi
Molecular Weight: 230.36
f1 (ppm)

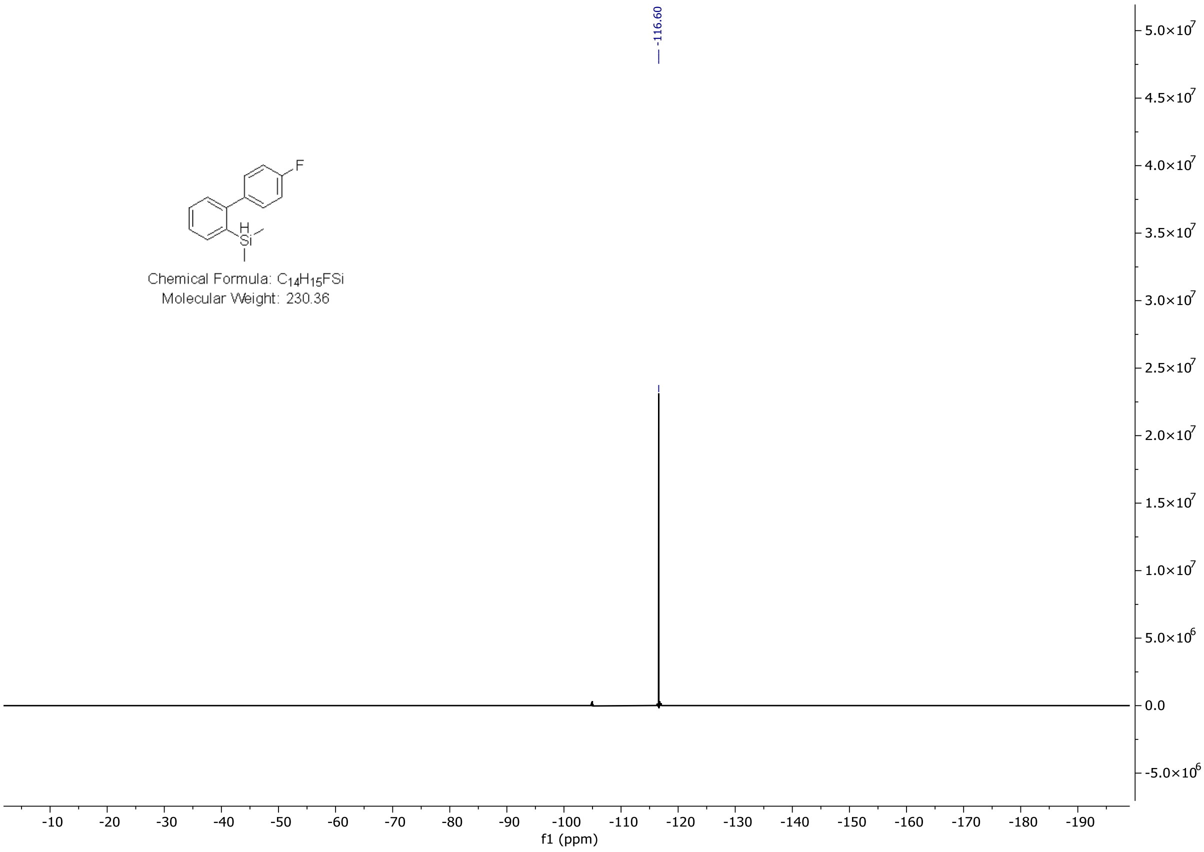
-116.60
F
H
Si
Chemical Formula: C14H15FSi
Molecular Weight: 230.36
f1 (ppm)

Dimethyl(4'-(trifluoromethoxy)-[1,1'-biphenyl]-2-yl)silane (5h).

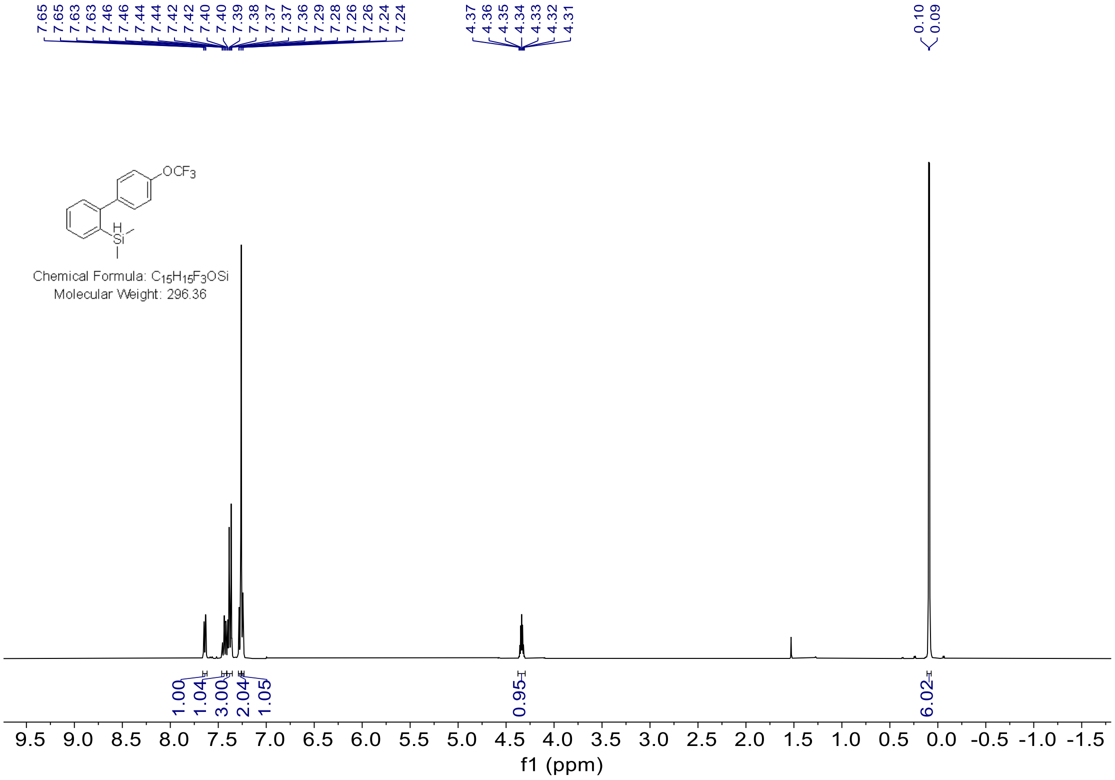

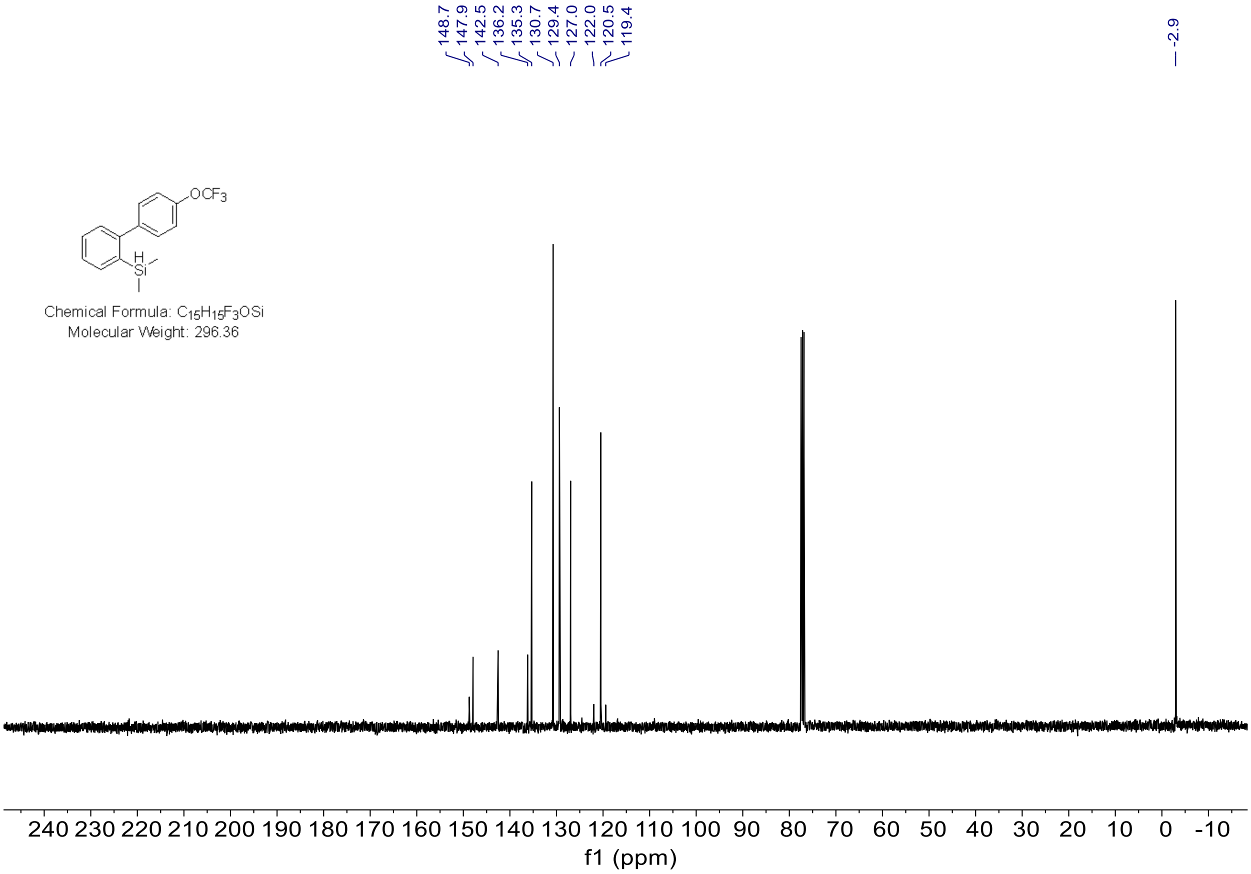

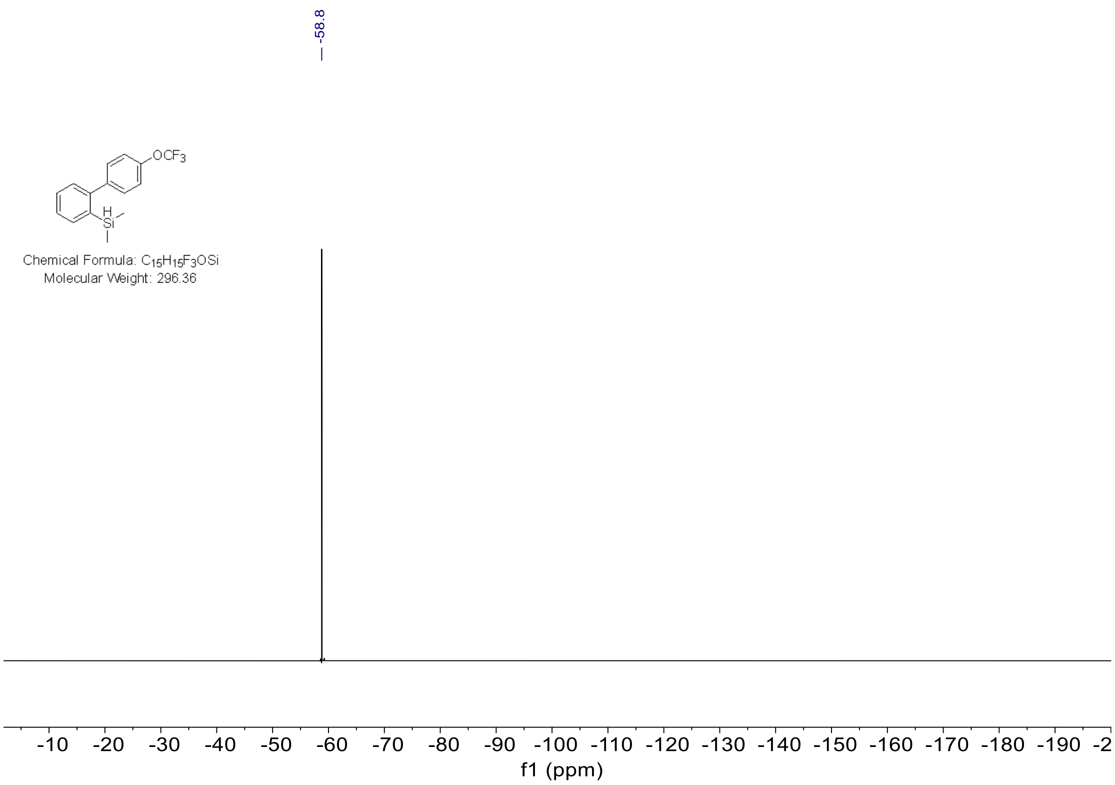

2-(Dimethylsilyl)-2'-methylbiphenyl (5i).

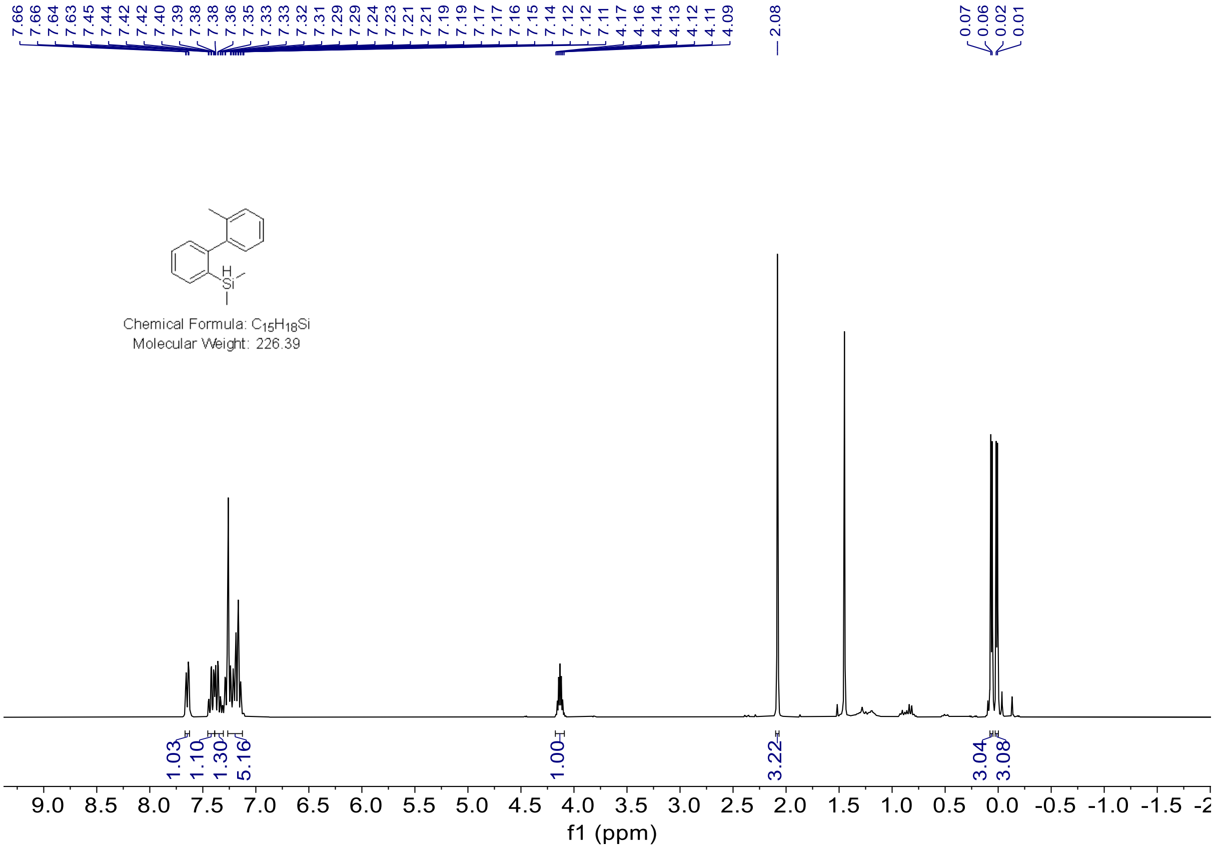

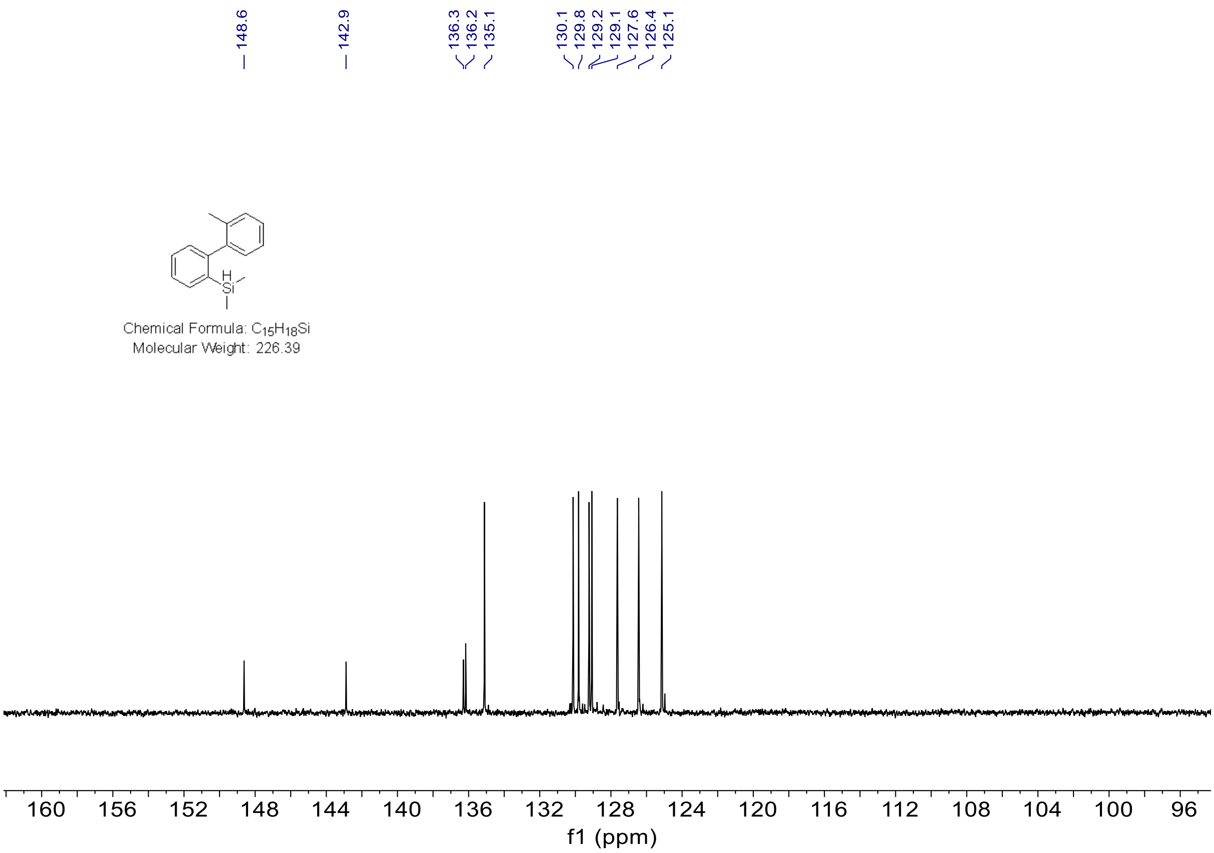

2-(Dimethylsilyl)-2'-methoxylbiphenyl (5j).

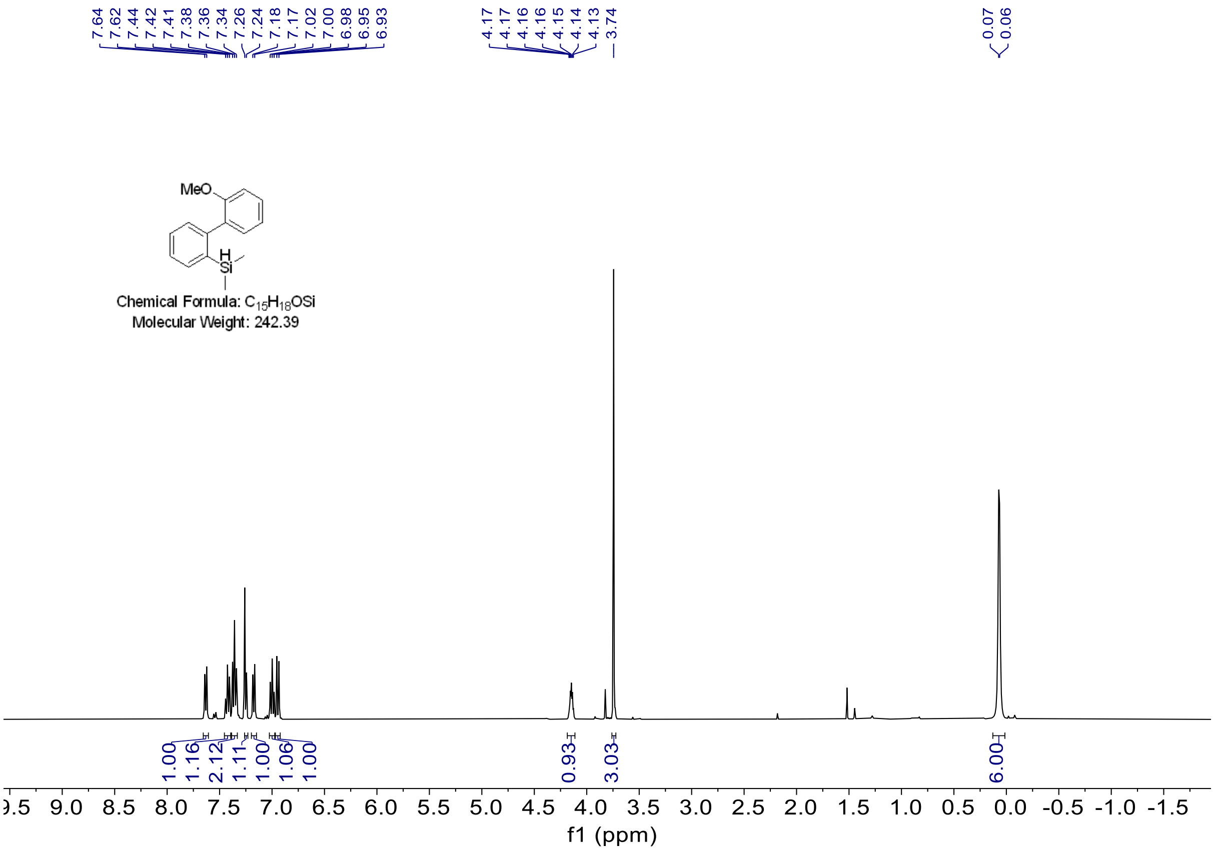

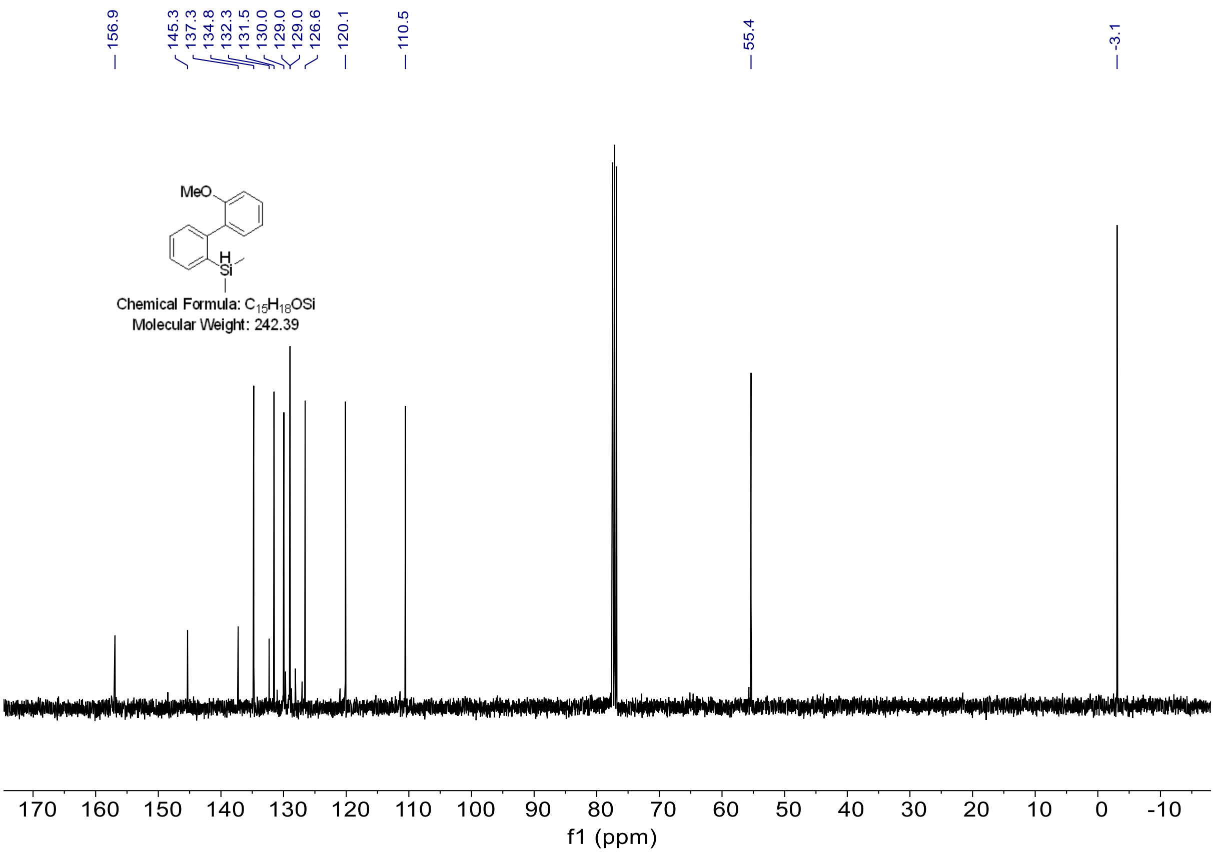

2-(Dimethylsilyl)-2'-methoxylbiphenyl (5k).

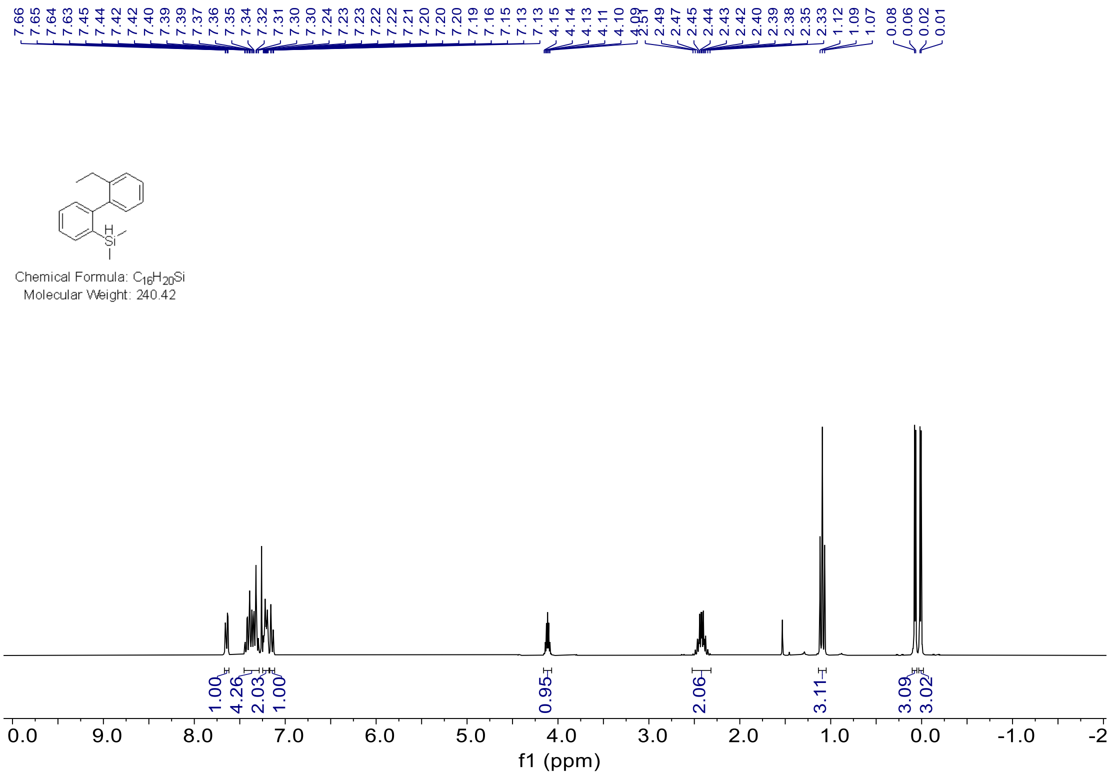

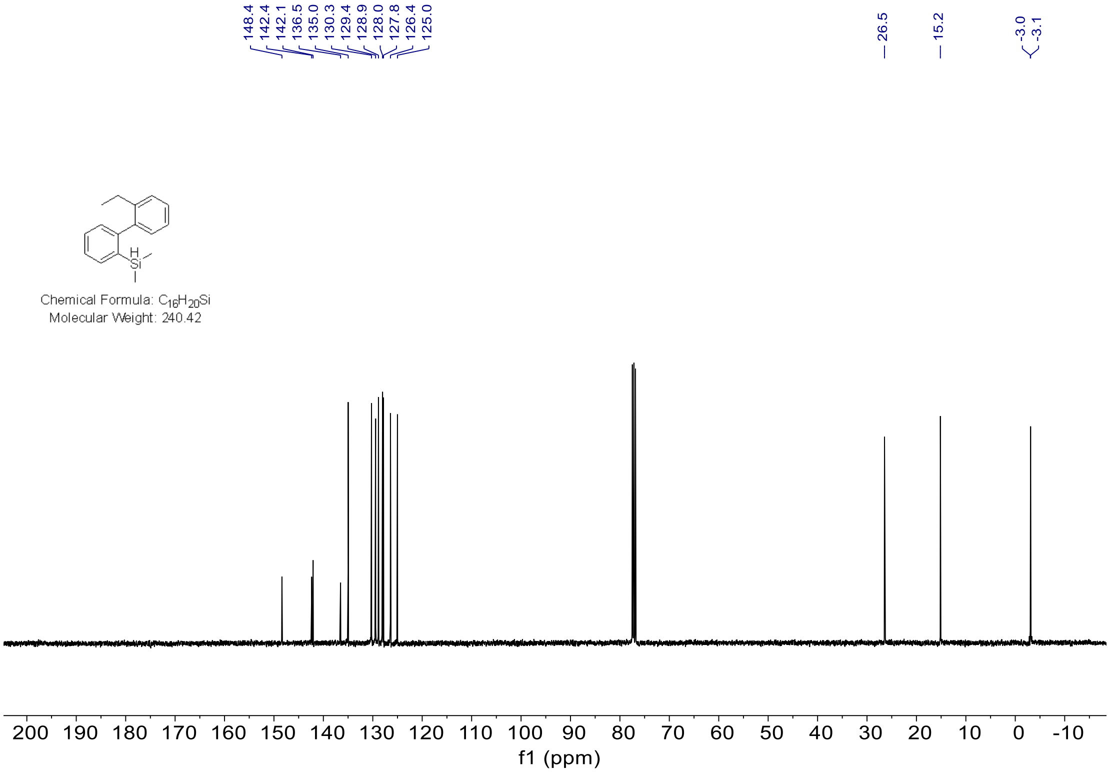

2-(Dimethylsilyl)-3'-methylbiphenyl (5l).

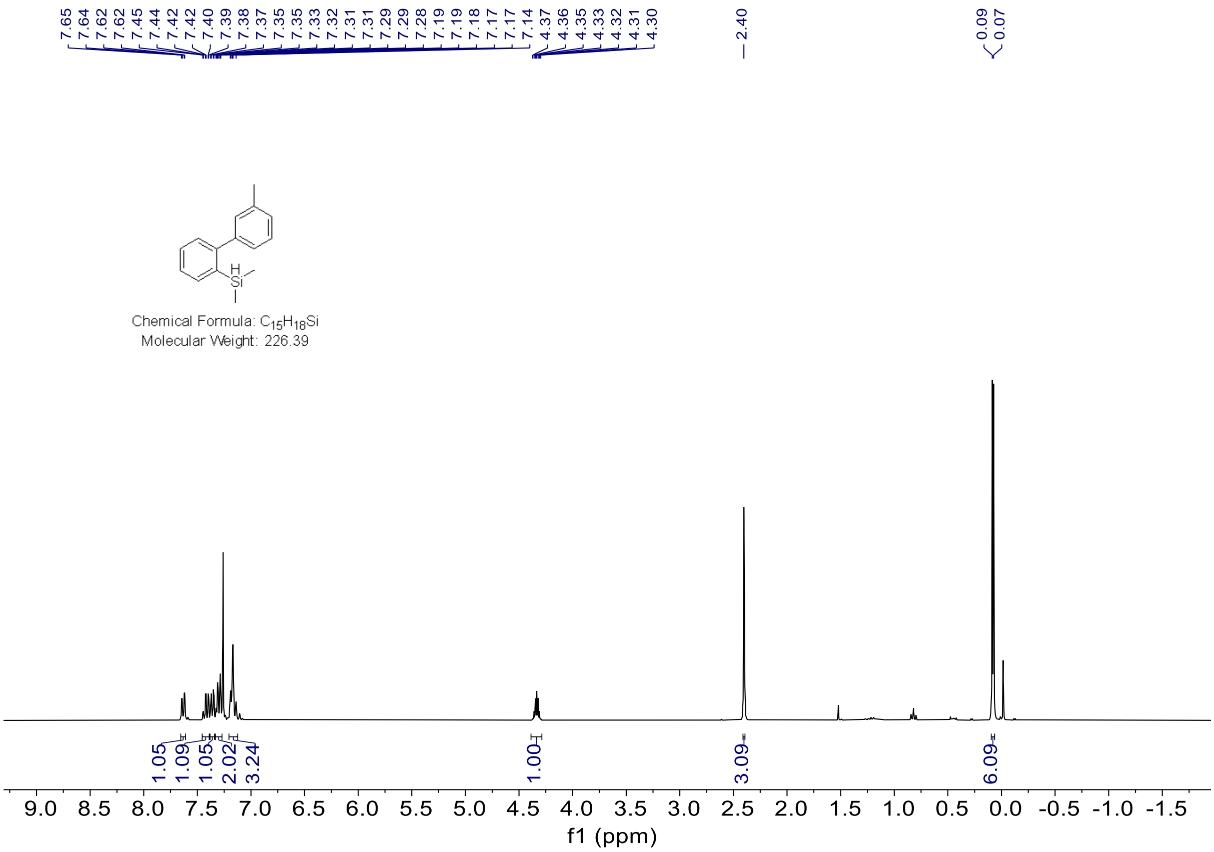

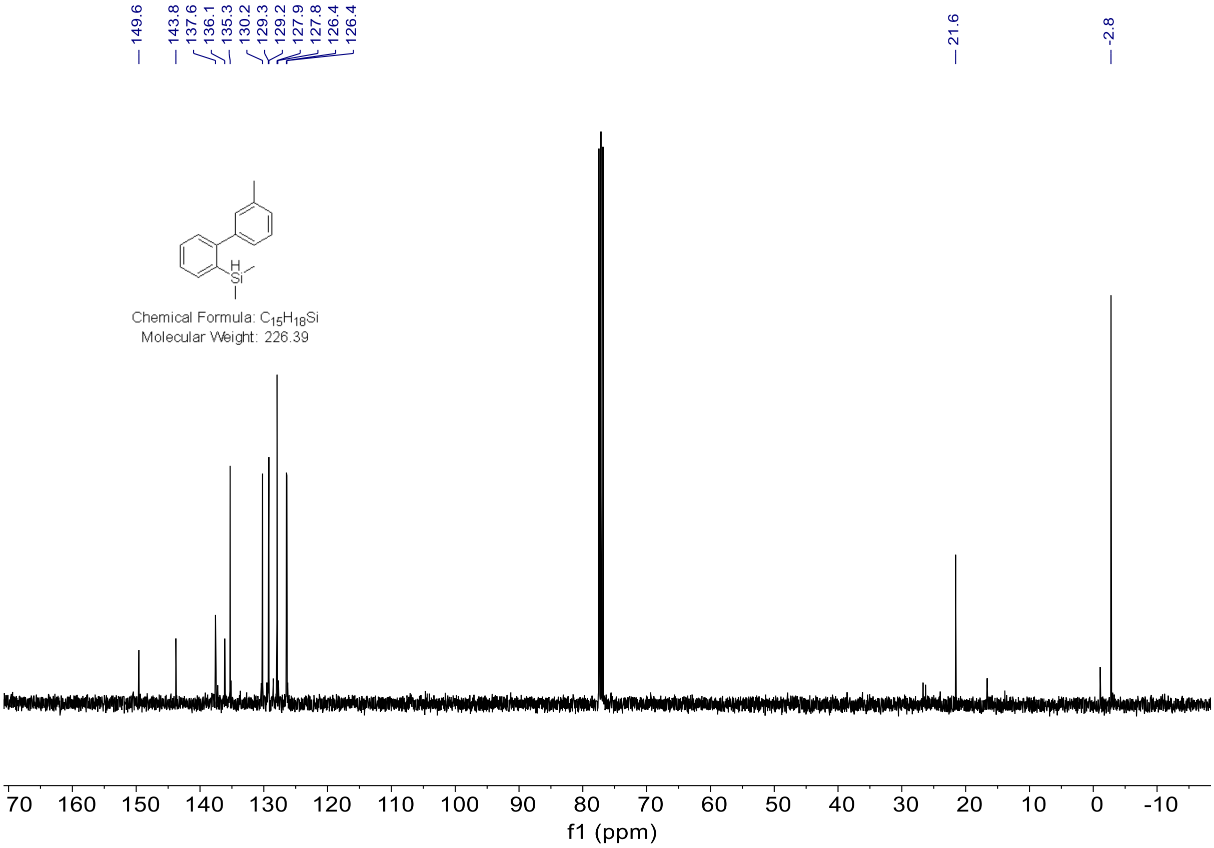

2-(Dimethylsilyl)-3',5'-bis(trifluoromethyl)biphenyl (5m).

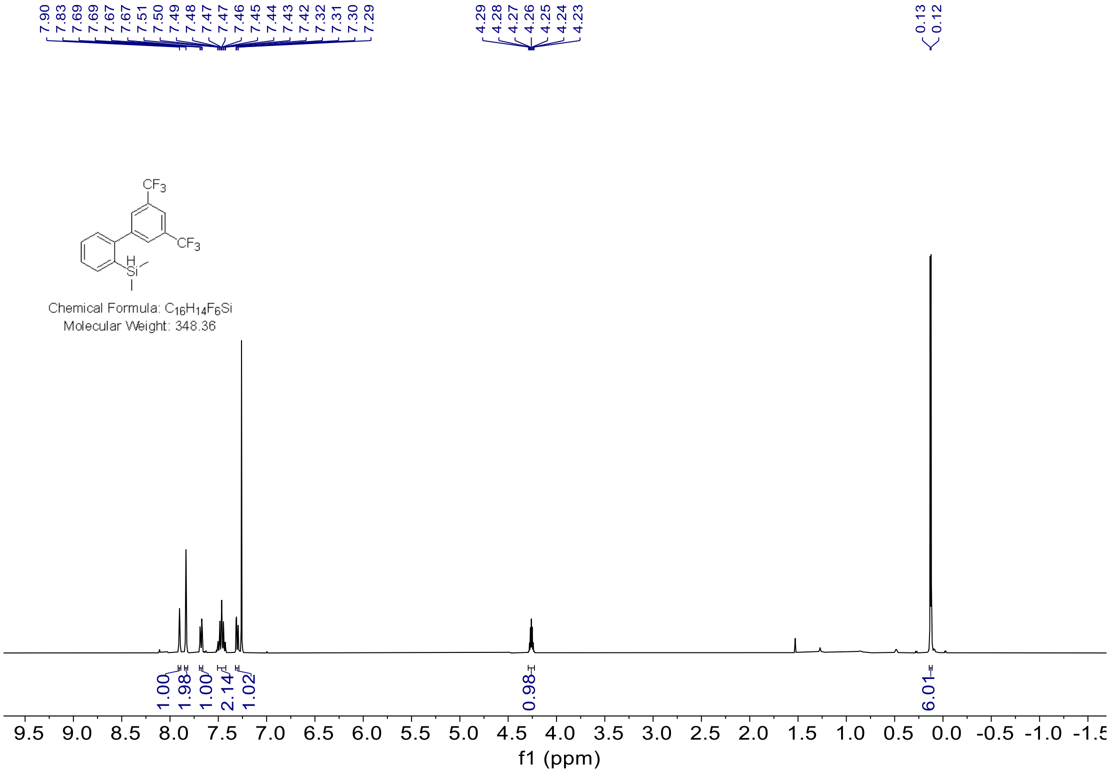

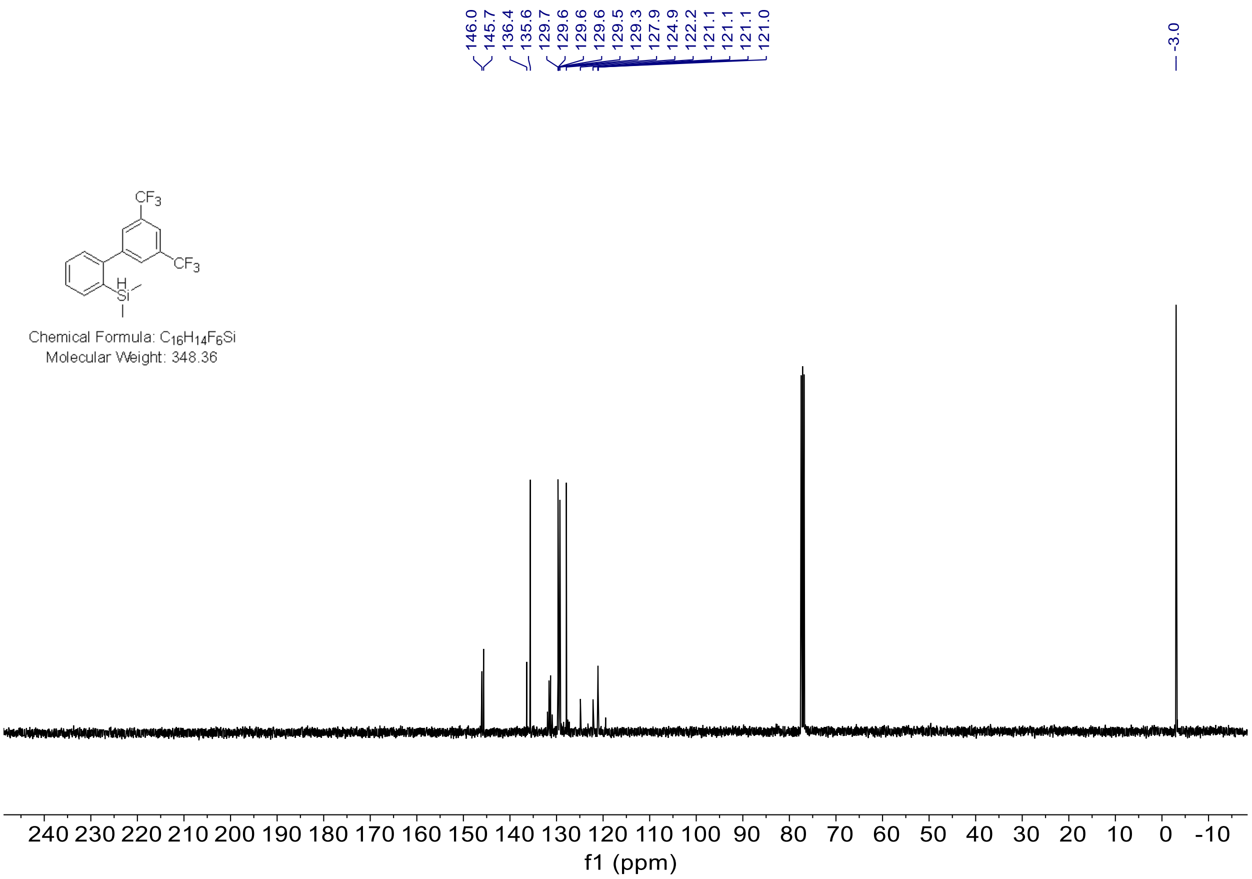
146.0
145.7
136.4
135.6
129.7
129.6
129.6
129.6
129.5
129.3
127.9
124.9
122.2
121.1
121.1
121.1
121.0
-3.0
CF3
CF3
H
Si
Chemical Formula: C16H14F6Si
Molecular Weight: 348.36
240 230 220 210 200 190 180 170 160 150 140 130 120 110 100 90 80 70 60 50 40 30 20 10 0 -10
f1 (ppm)

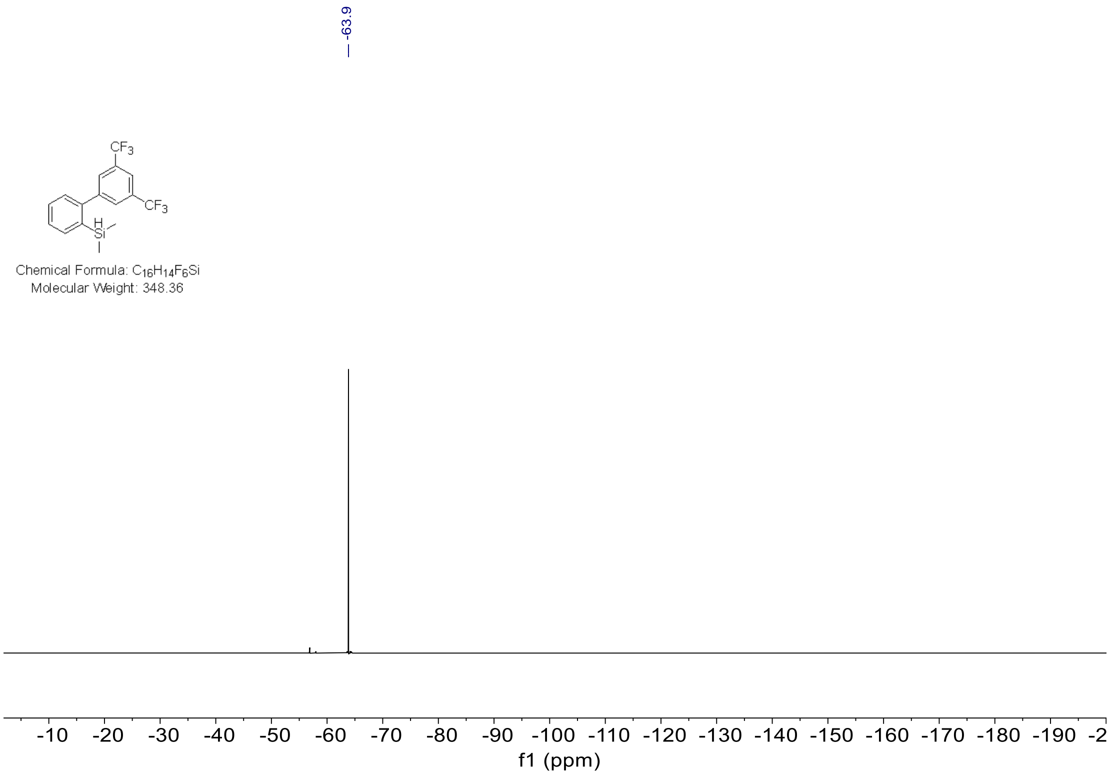
-63.9
CF3
CF3
H
Si
Chemical Formula: C16H14F6Si
Molecular Weight: 348.36
-10 -20 -30 -40 -50 -60 -70 -80 -90 -100 -110 -120 -130 -140 -150 -160 -170 -180 -190 -2
f1 (ppm)

2-(2-Dimethylsilylphenyl)naphthalene (5n).

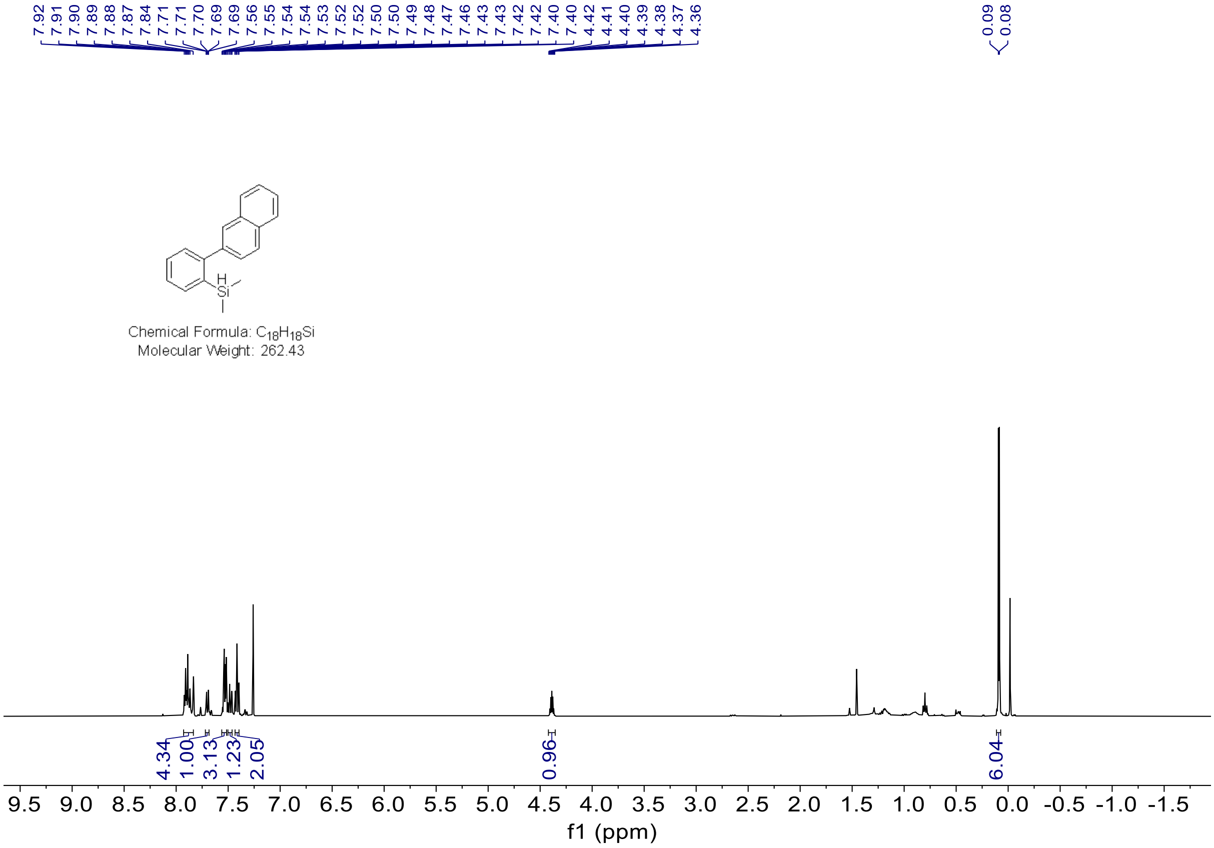

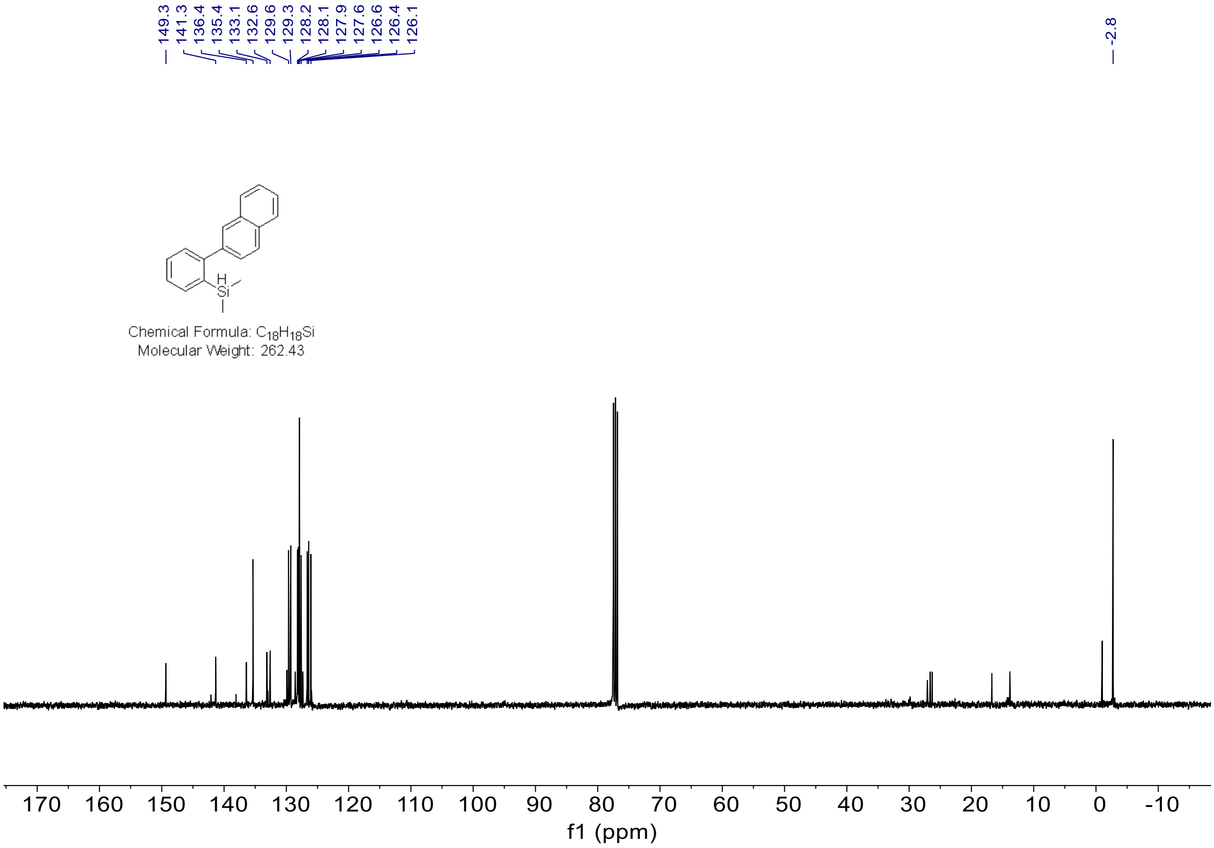

(2-(6-Methoxynaphthalen-2-yl)phenyl)dimethylsilane (5o).

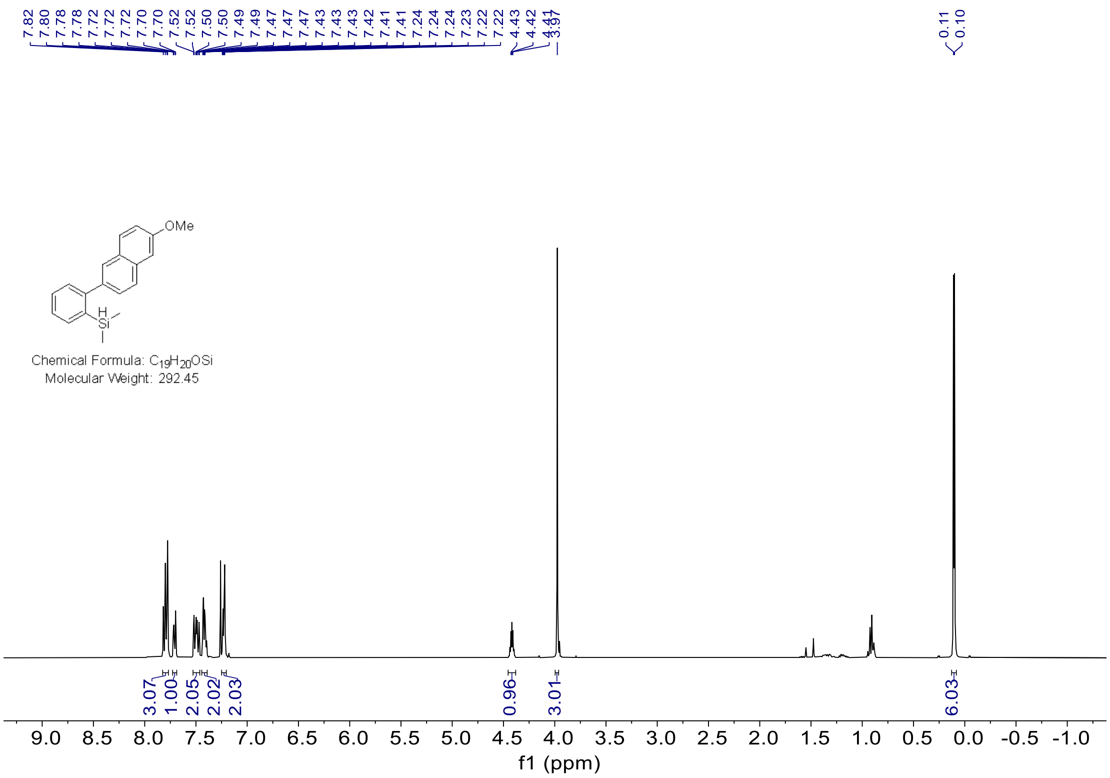

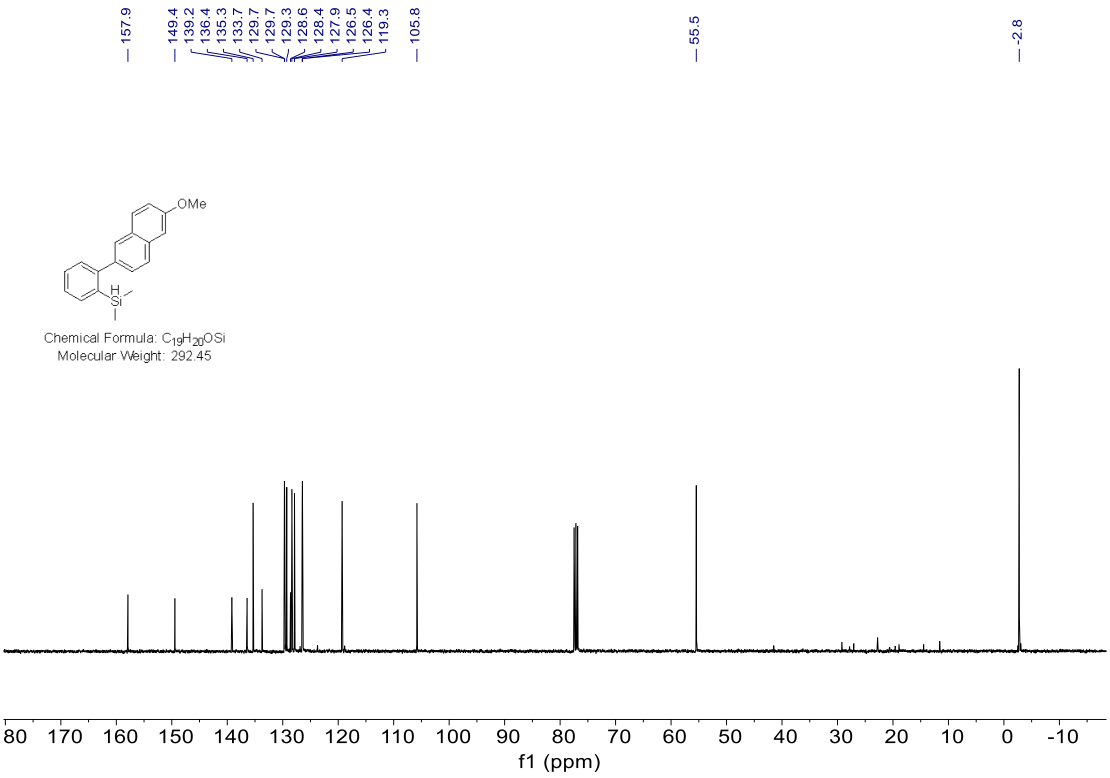

2-(Dimethylsilyl)phenyl]ferrocene (5p).

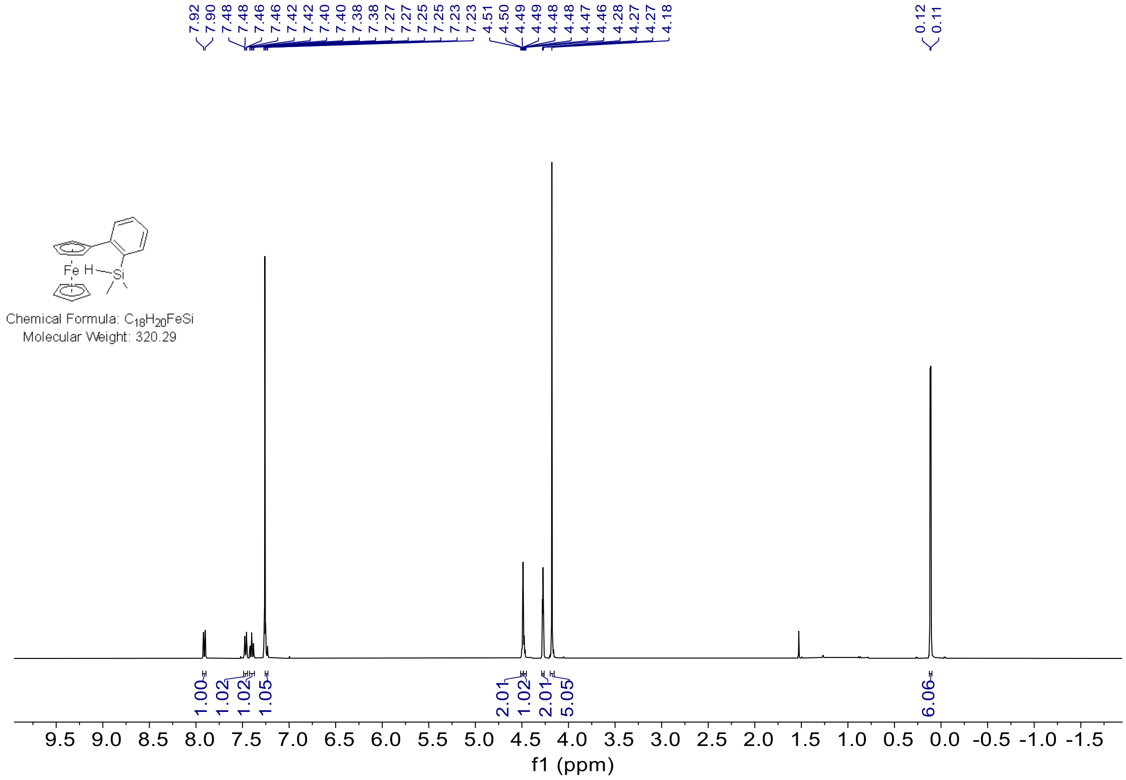

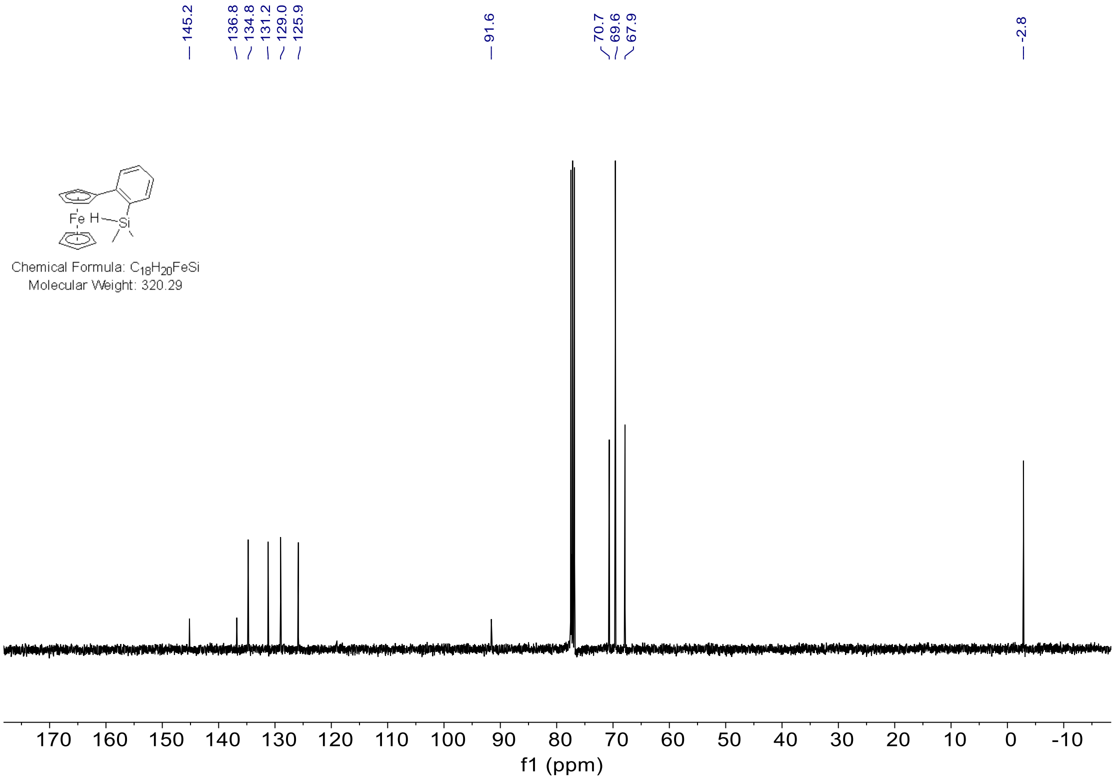

Dimethyl(1-phenylnaphthalen-2-yl)silane (5q).

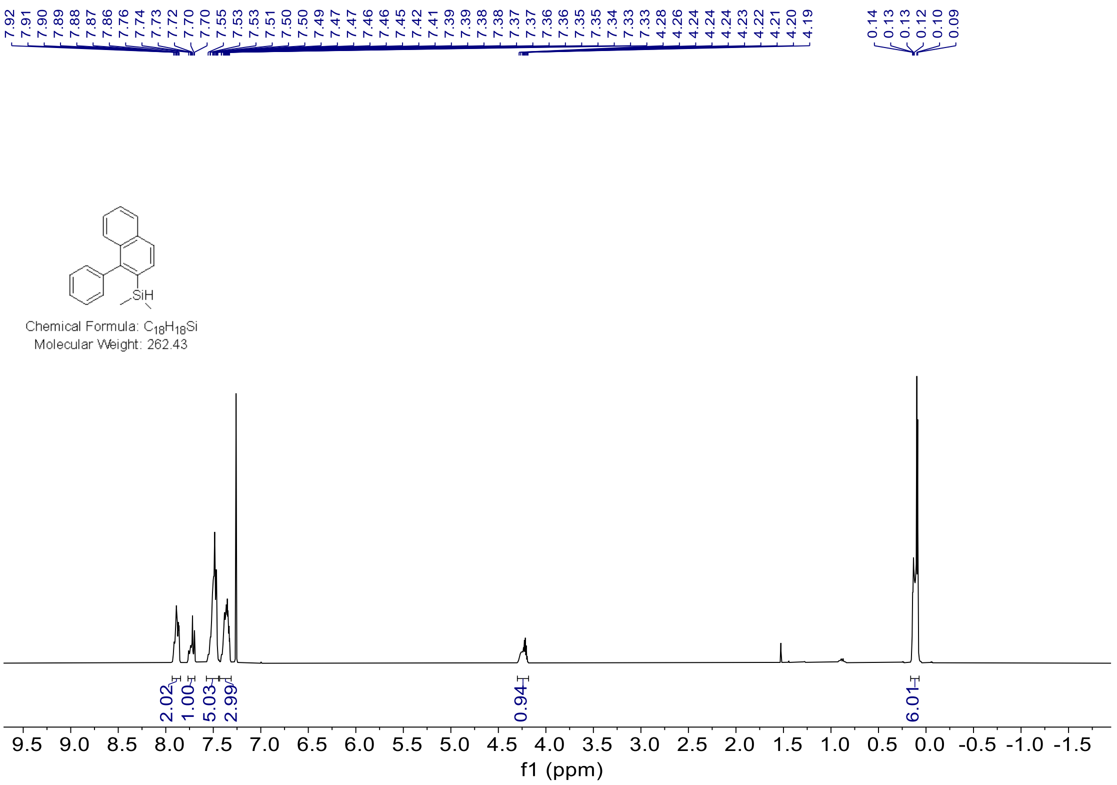

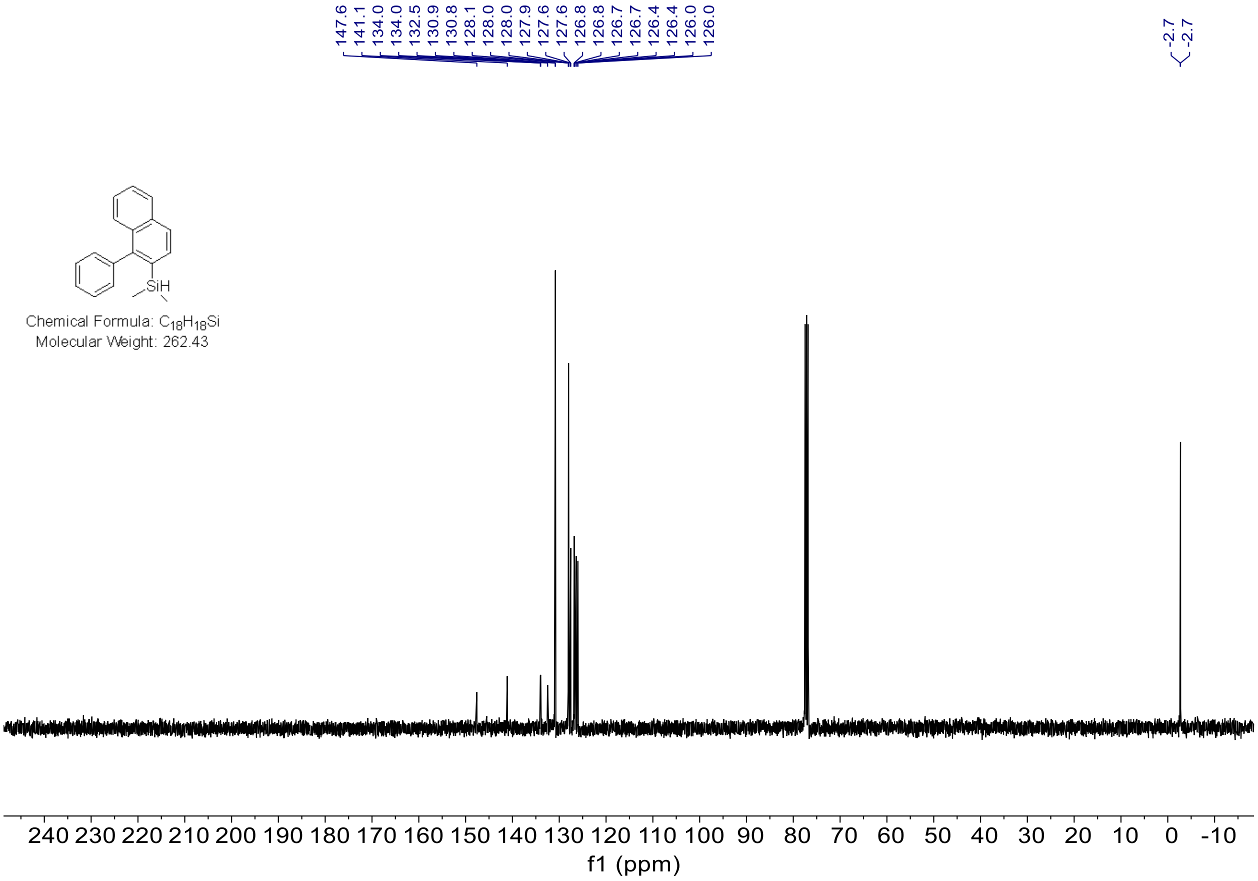

2,2'''-Bis(dimethylsilyl)-1,1':4',1'':4'',1'''-quaterphenyl (5r).

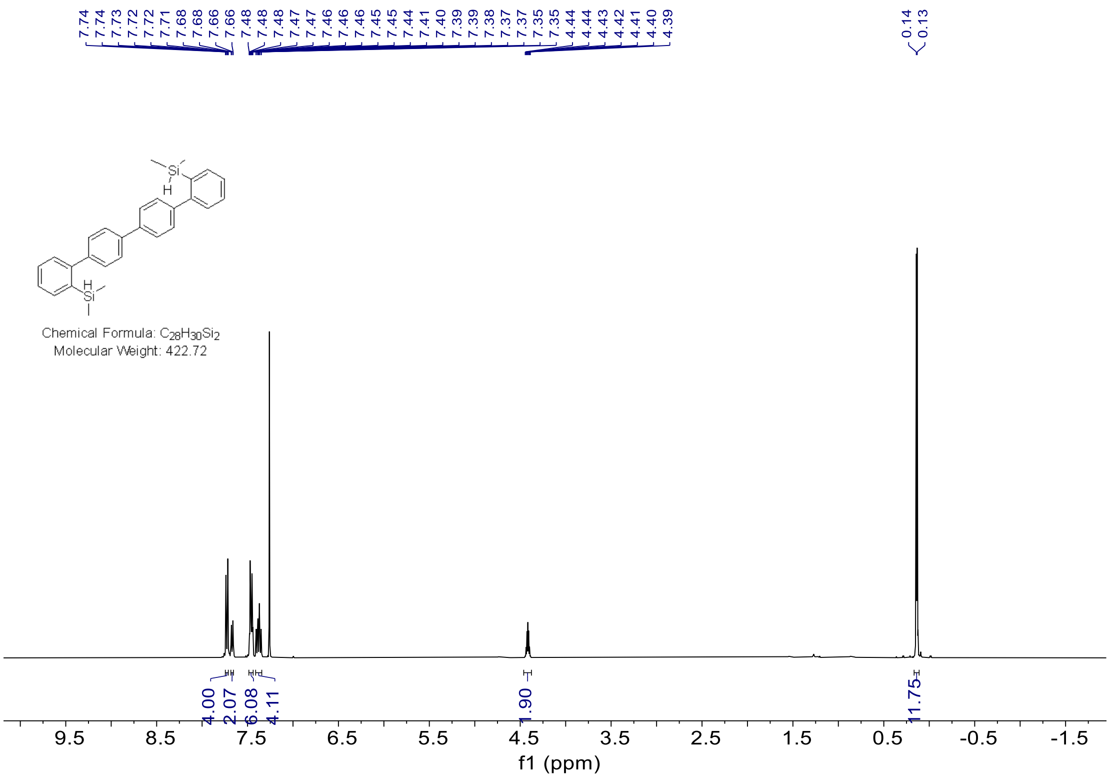

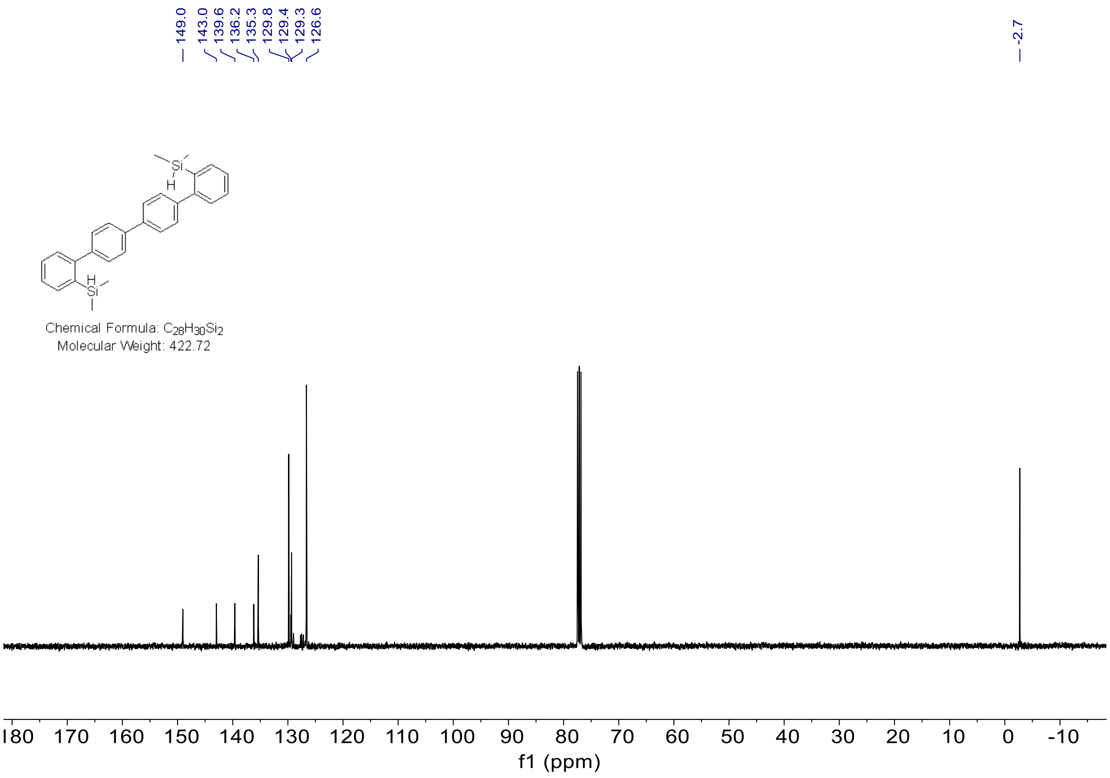

2,2''-Bis(dimethylsilyl)-*p*-terphenyl (5s).

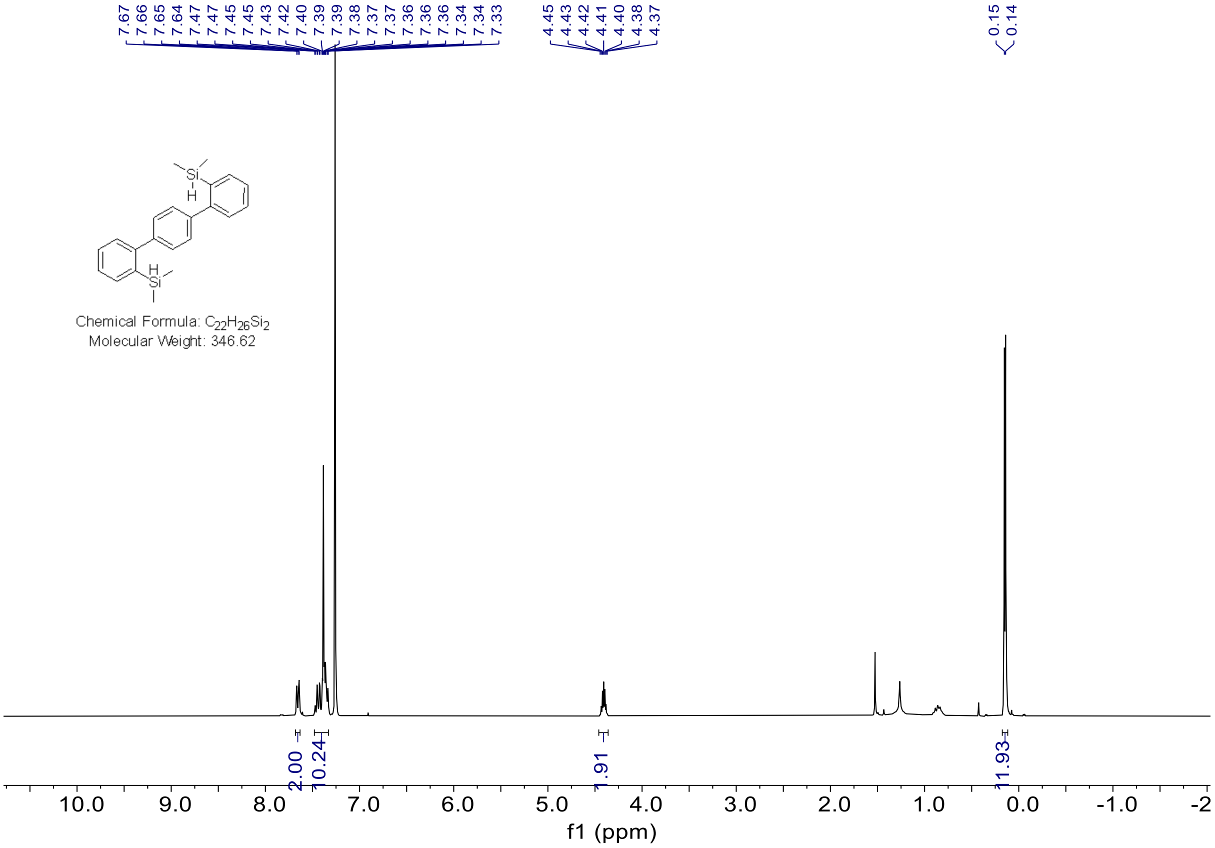

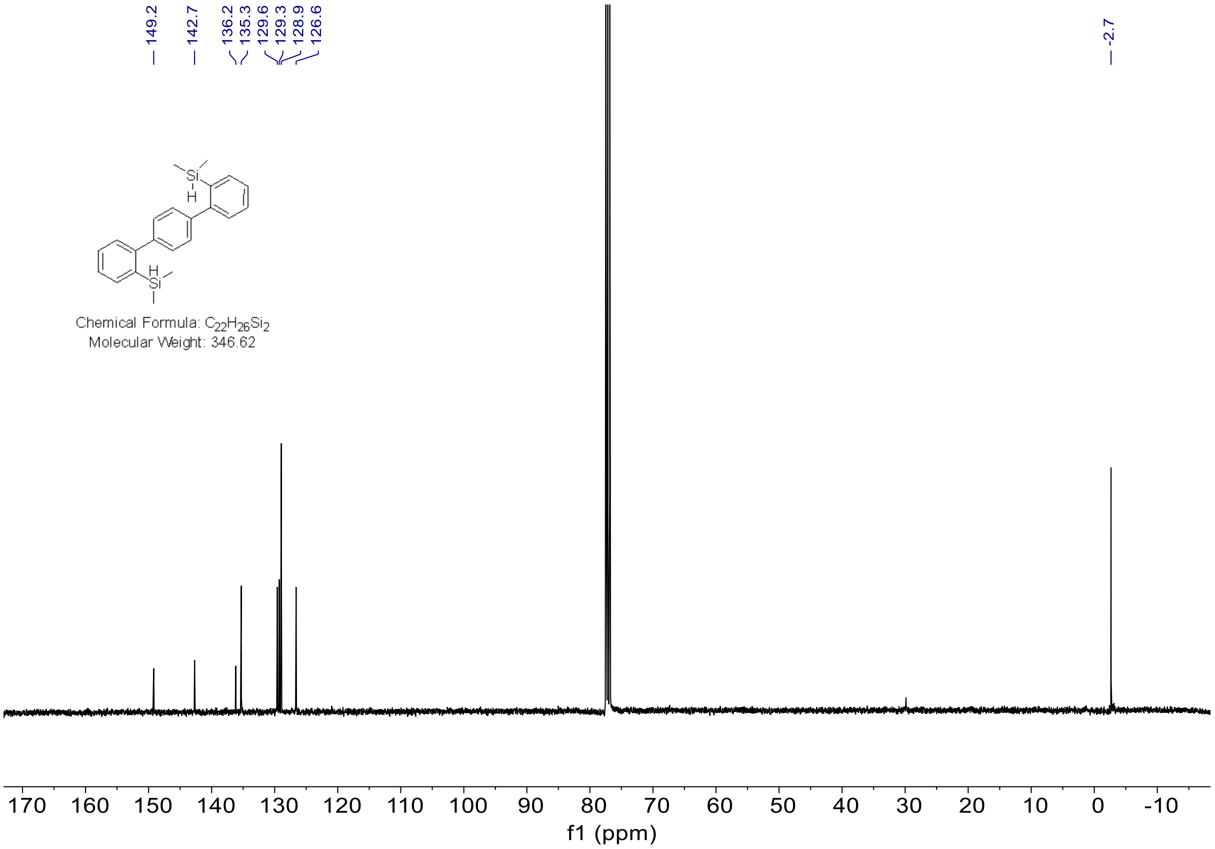

Bis(biphenyl-2-yl)silane (5t).

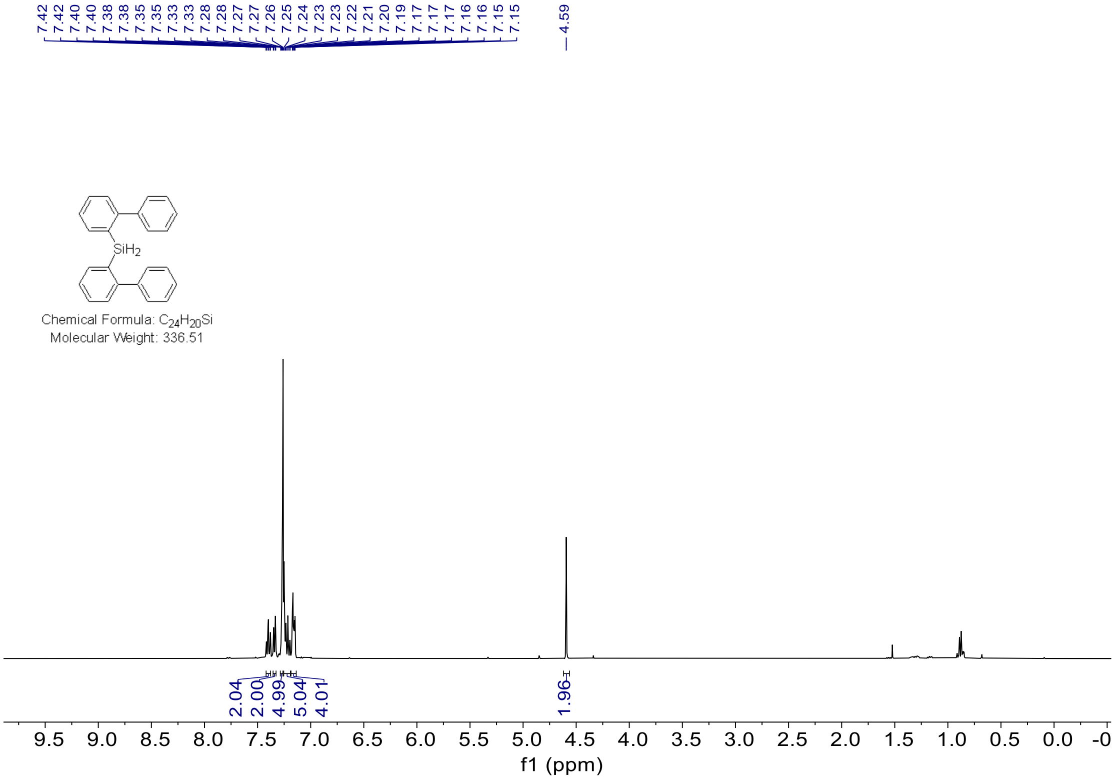

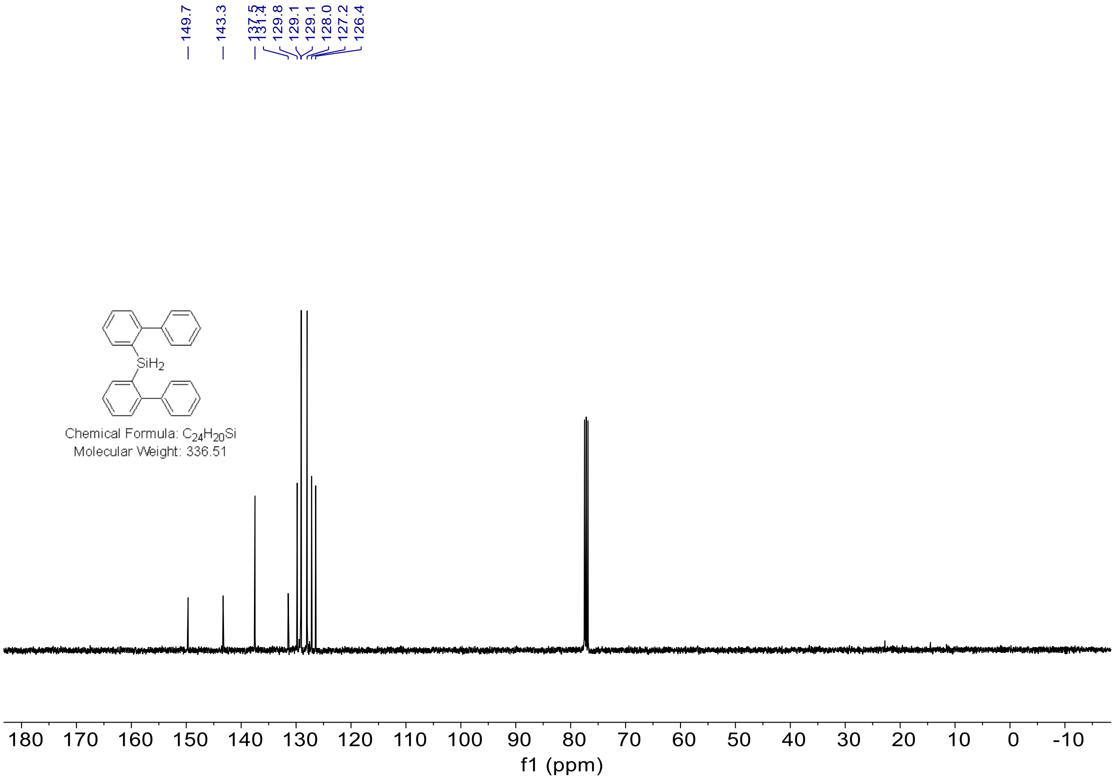

1,1'-Biphenyl-2-yl-diphenylsilane (5u).

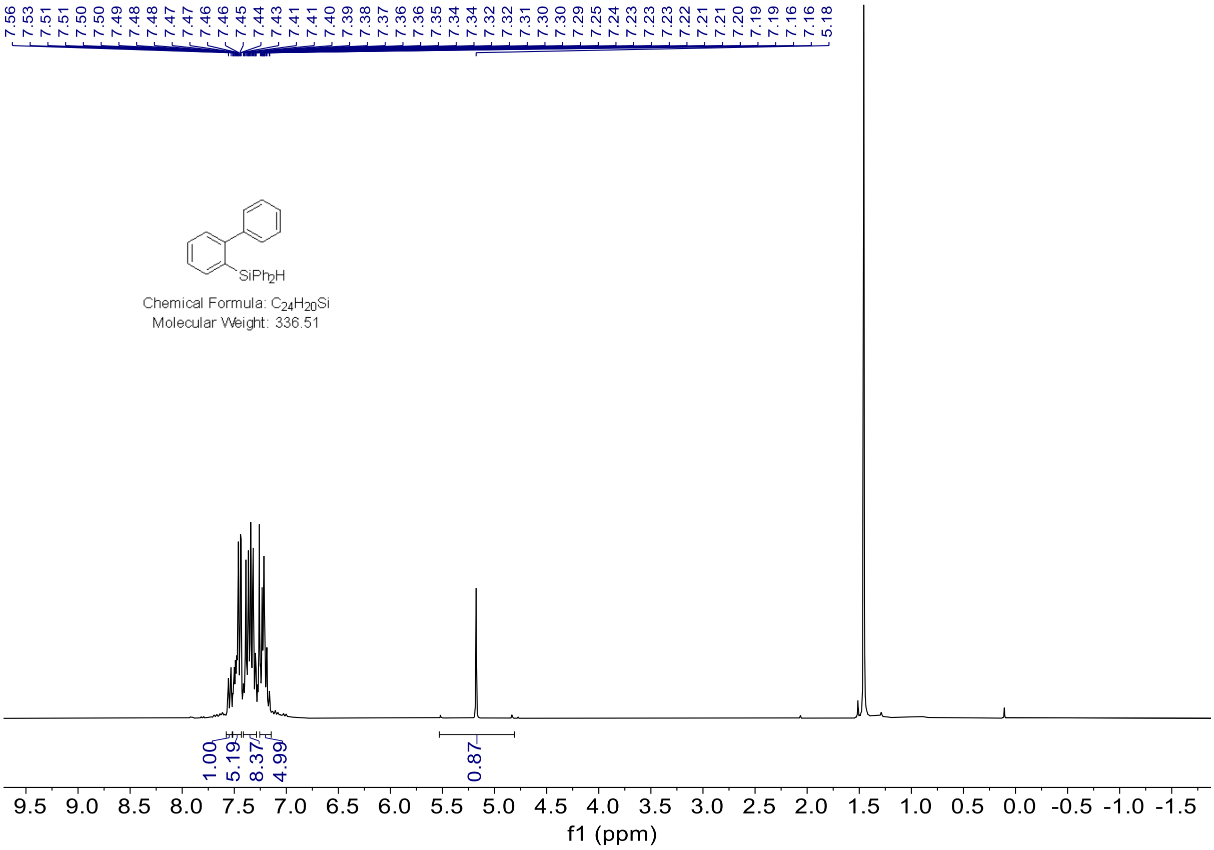

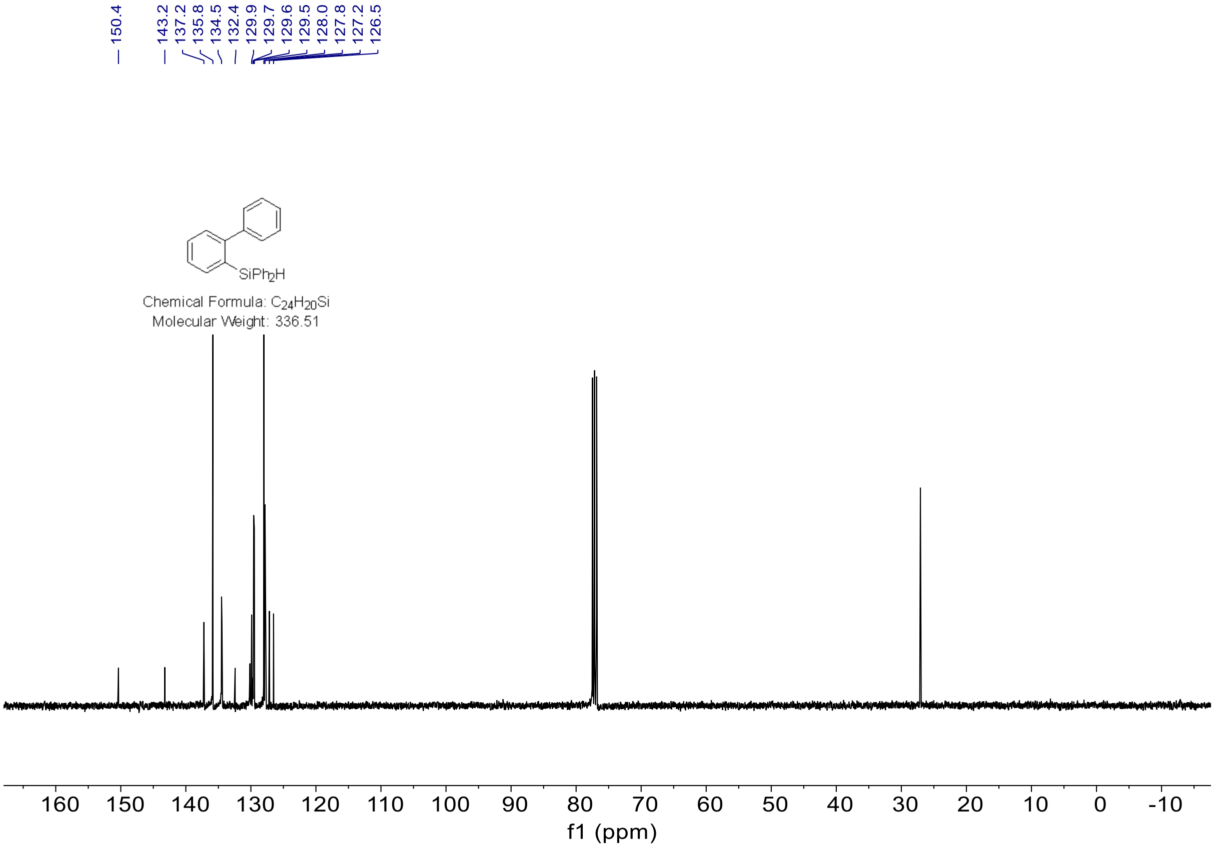

(Biphenyl-2-yl)methylphenylsilane (5v).

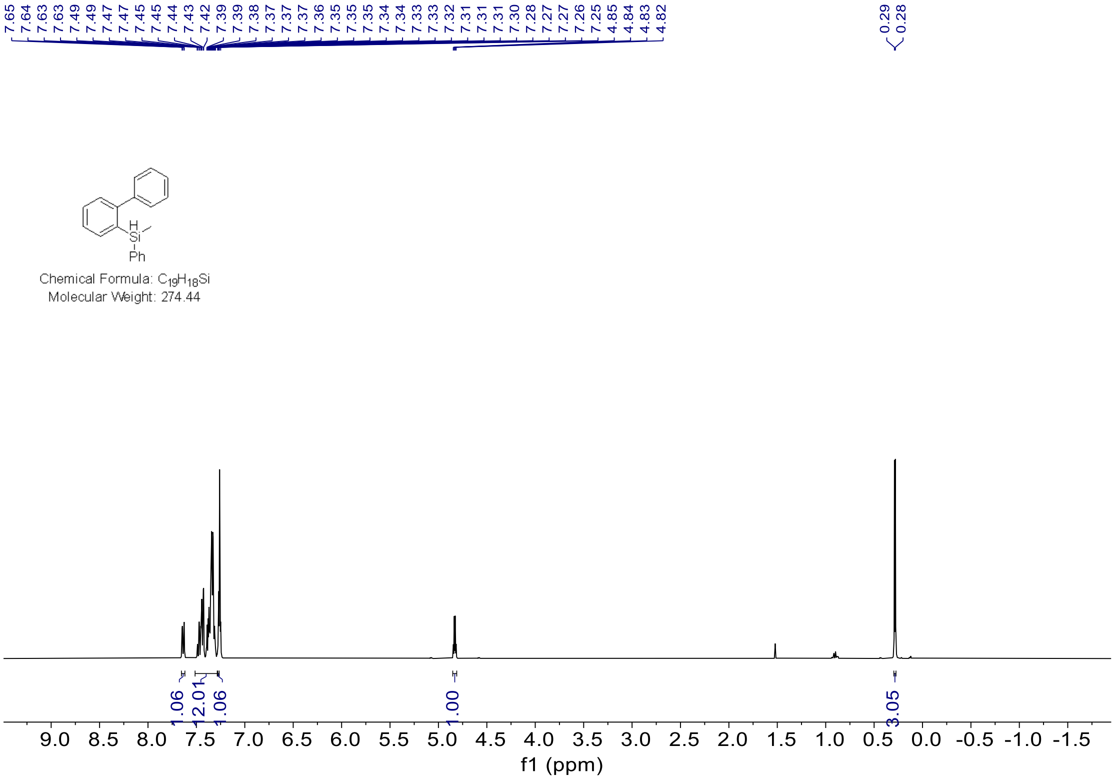

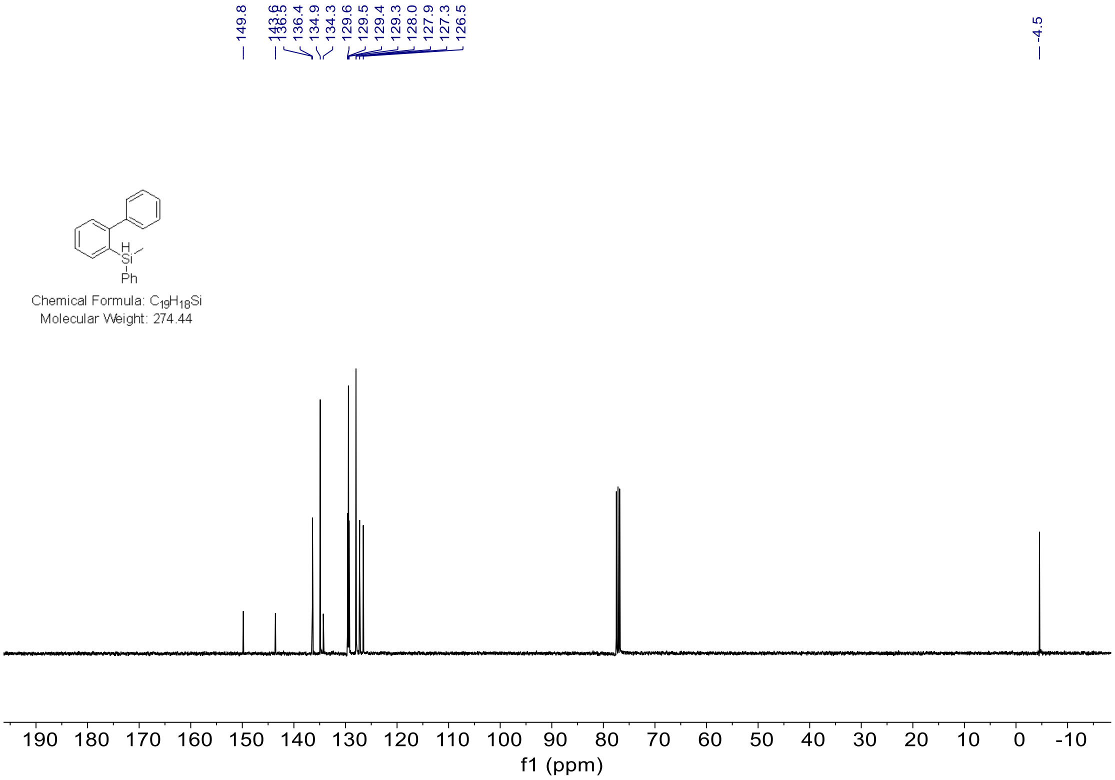

2-(Diethylsilyl)biphenyl (5w).

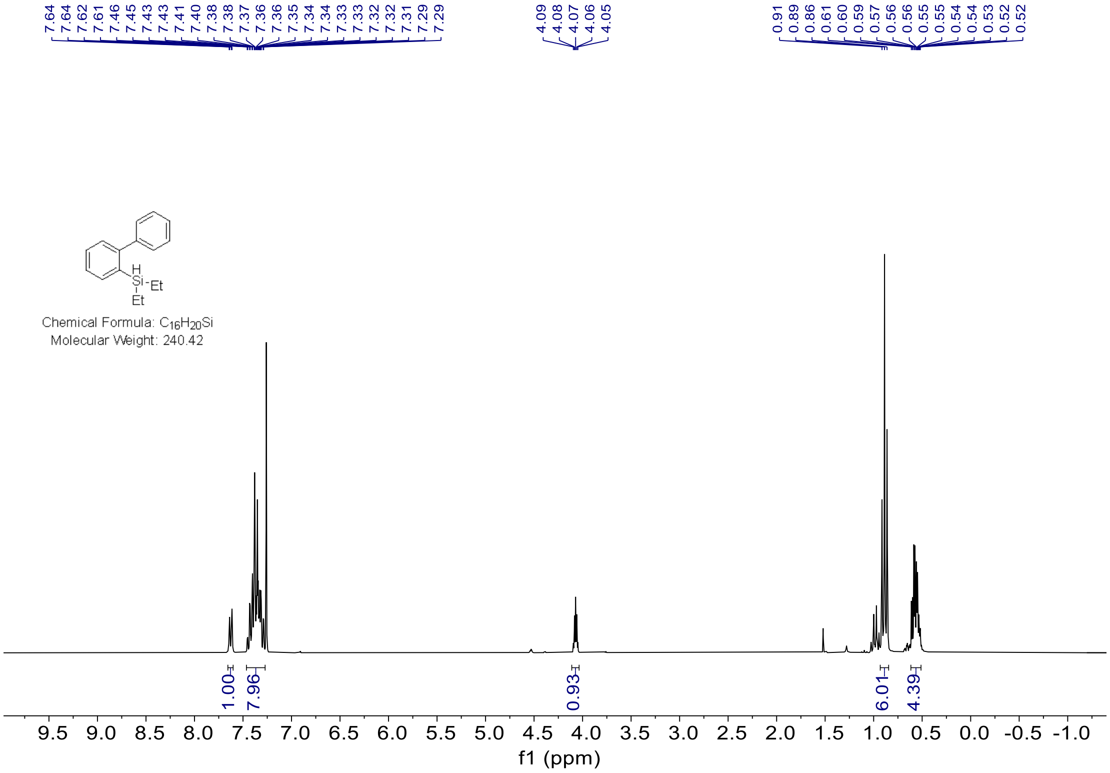

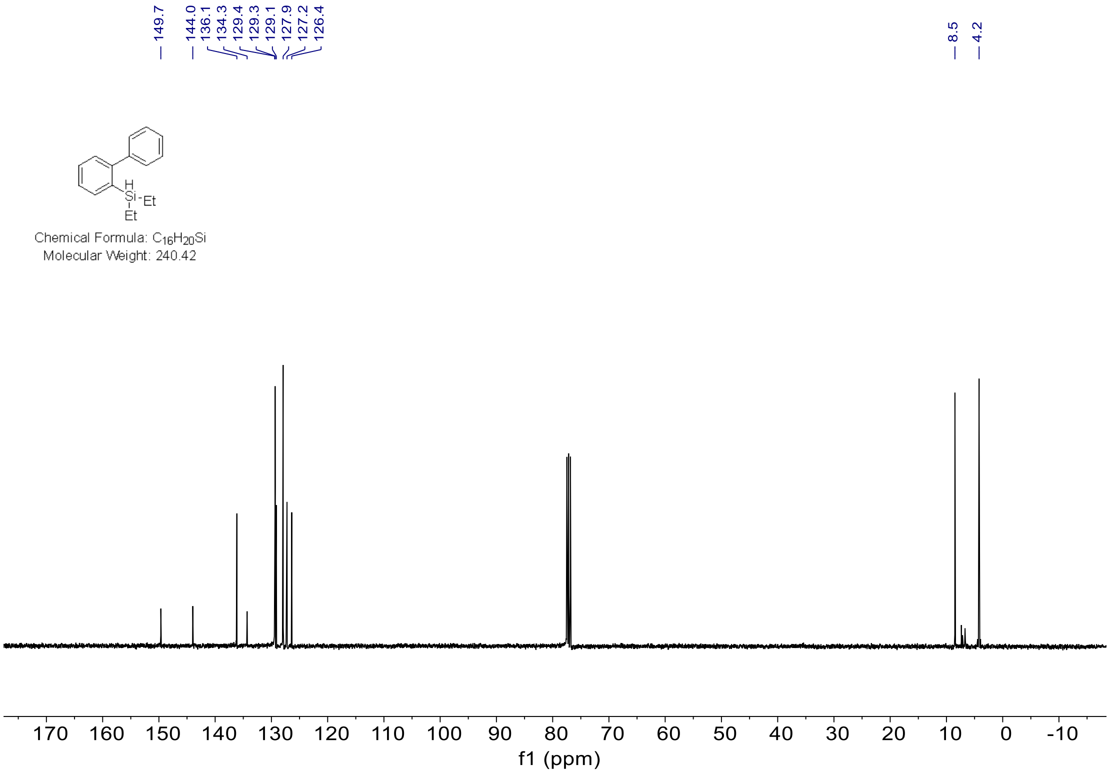

2-(Dimethylgermyl)biphenyl (5x).

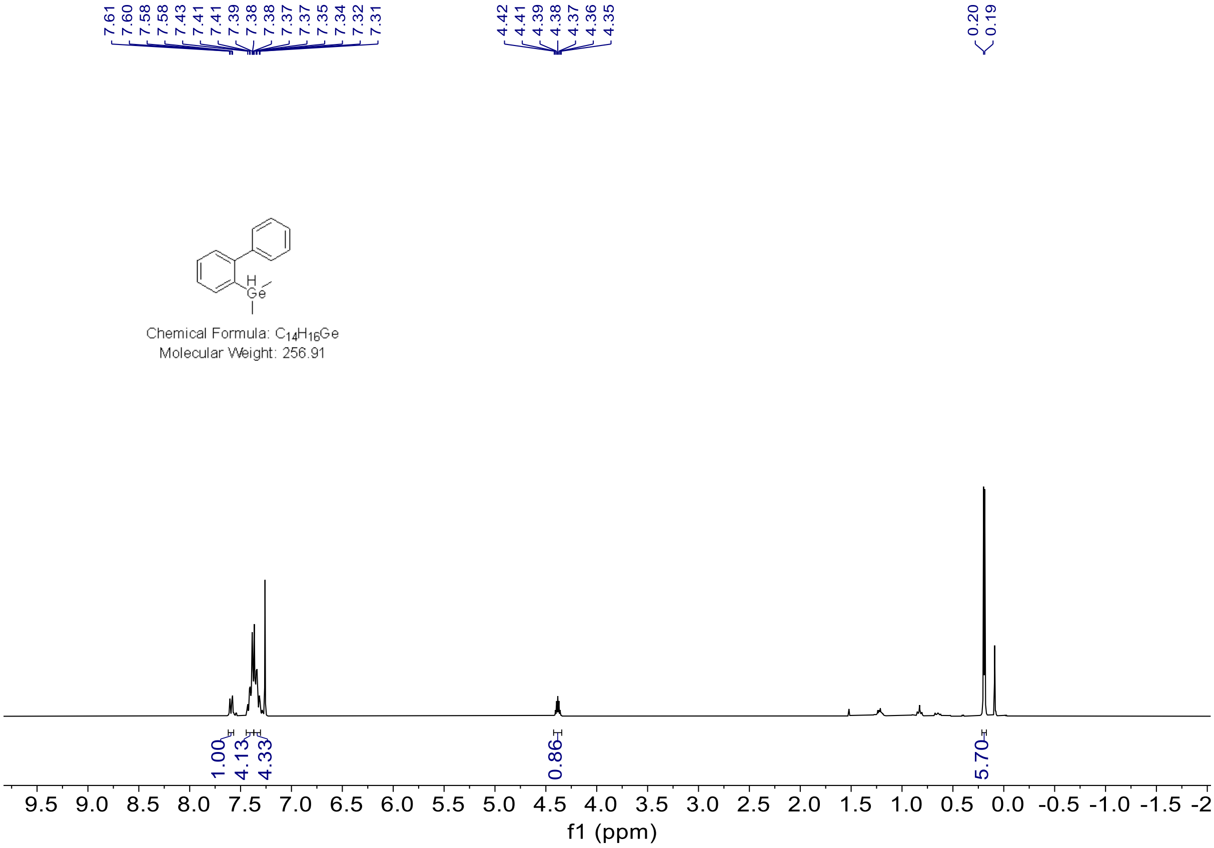

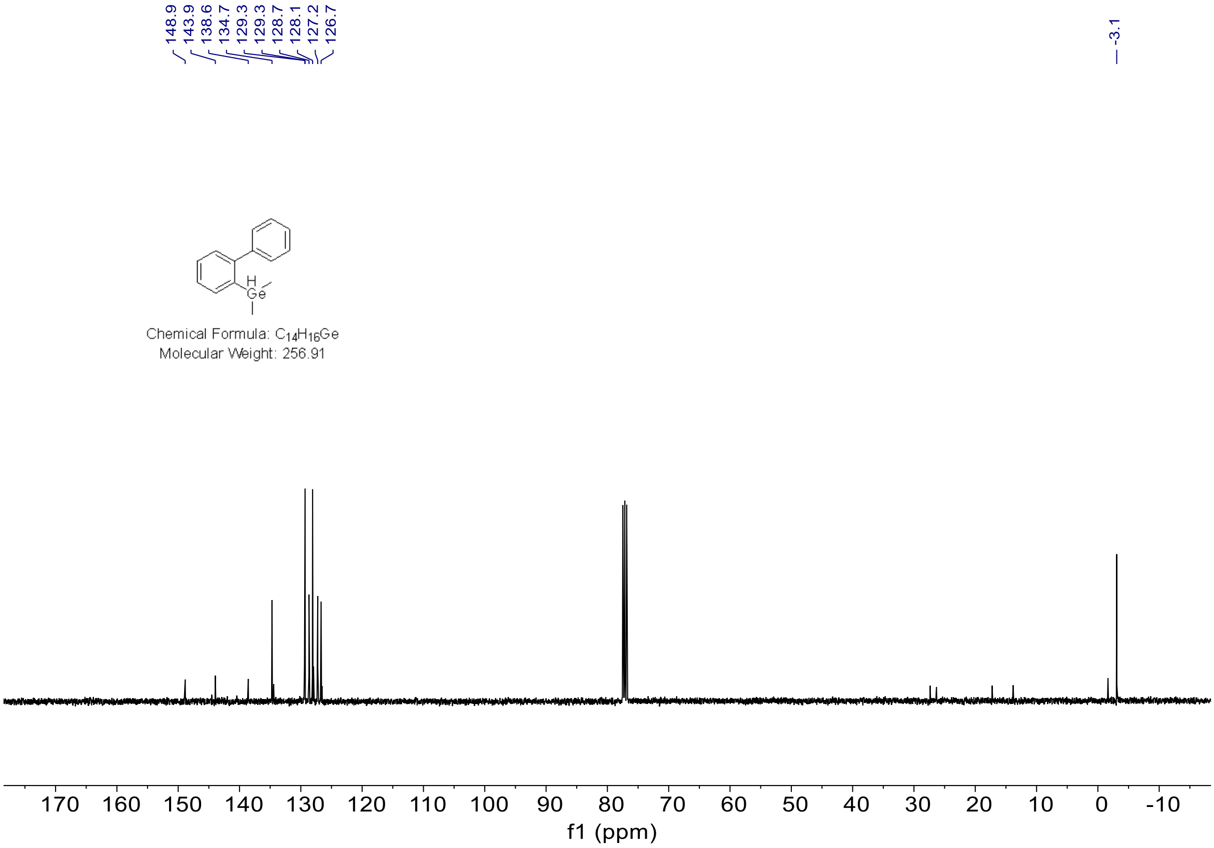

9,9-Dimethyl-9-silafluorene (6a).

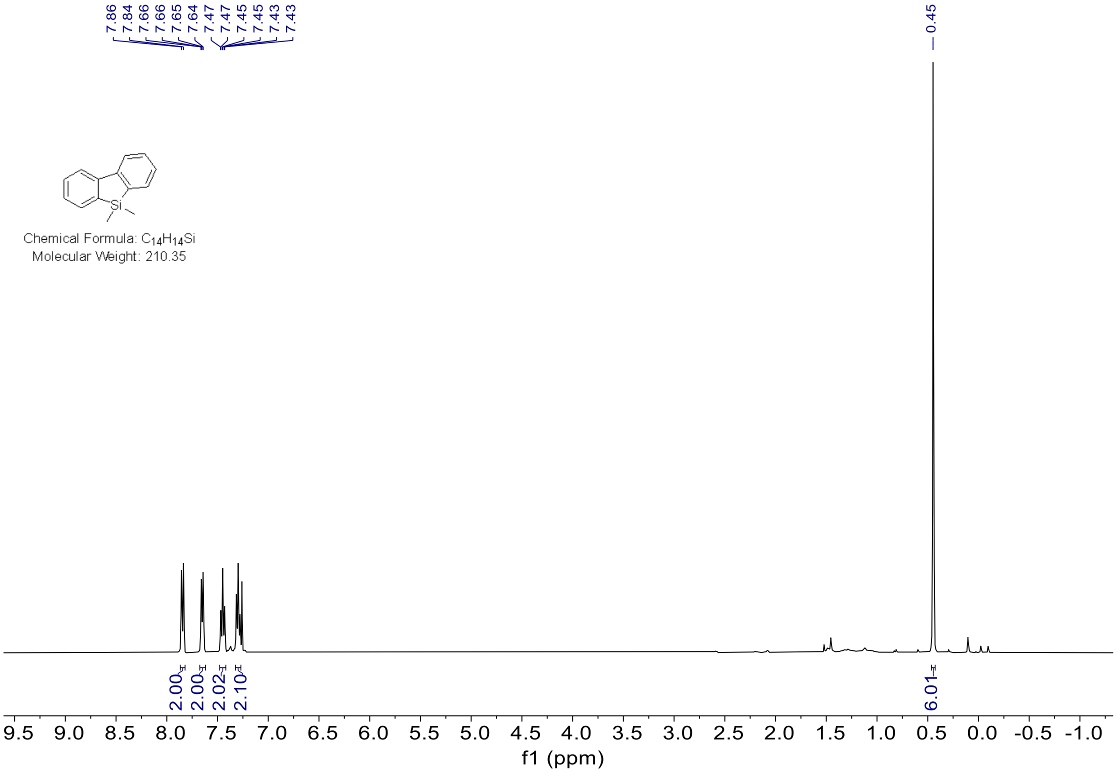

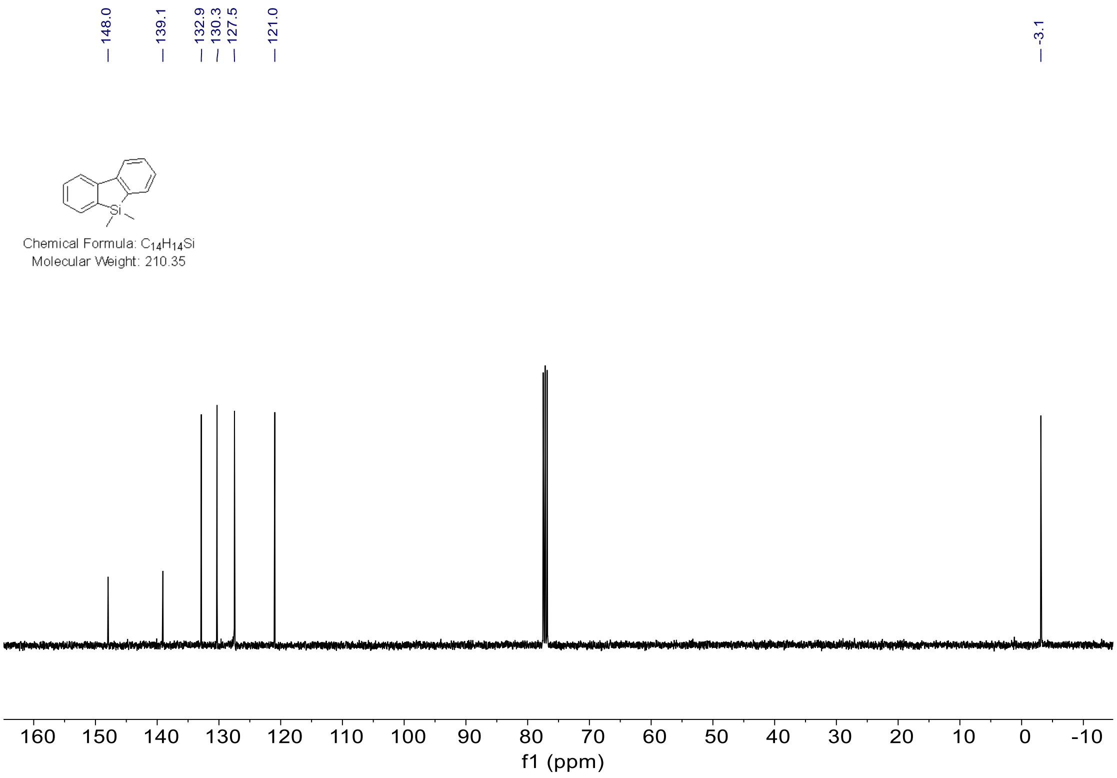

5,5-Dimethyl-3-phenyl-5*H*-dibenzo[*b,d*]silole (6b).

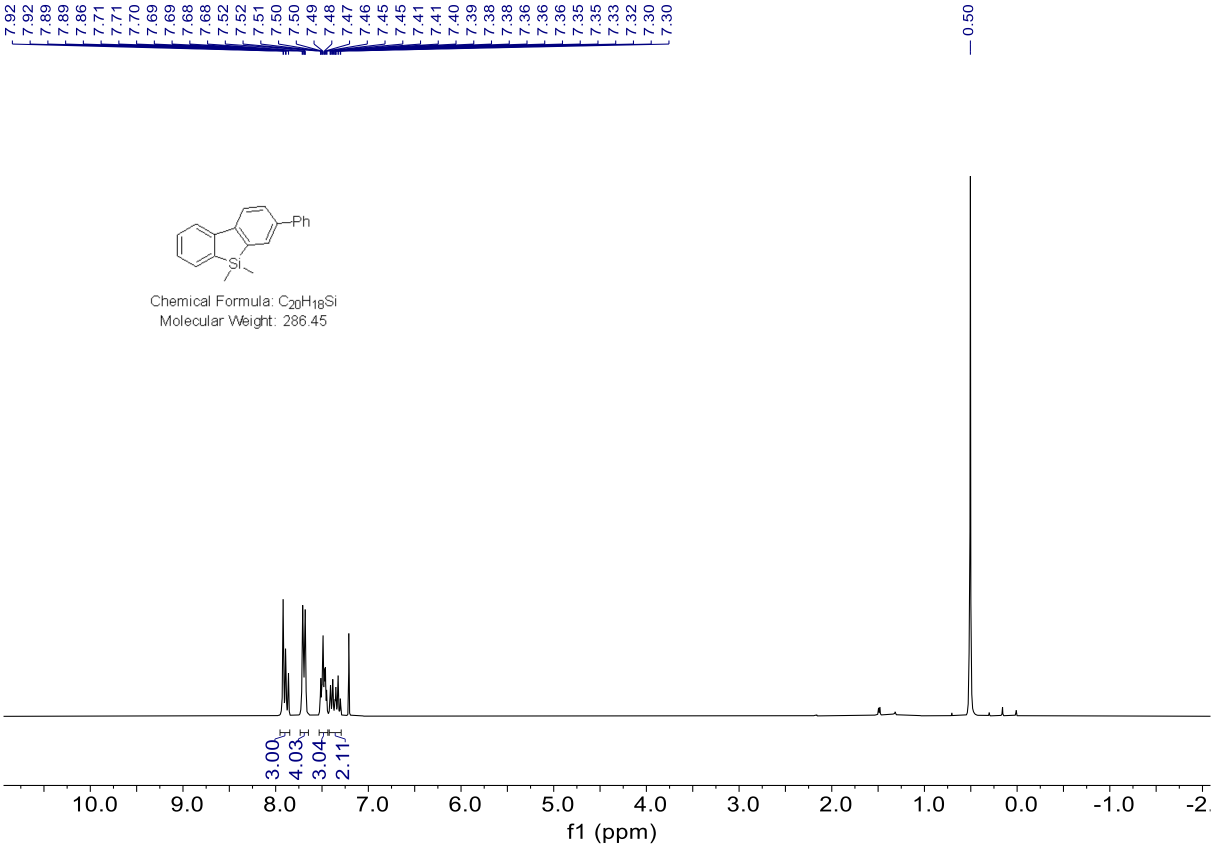

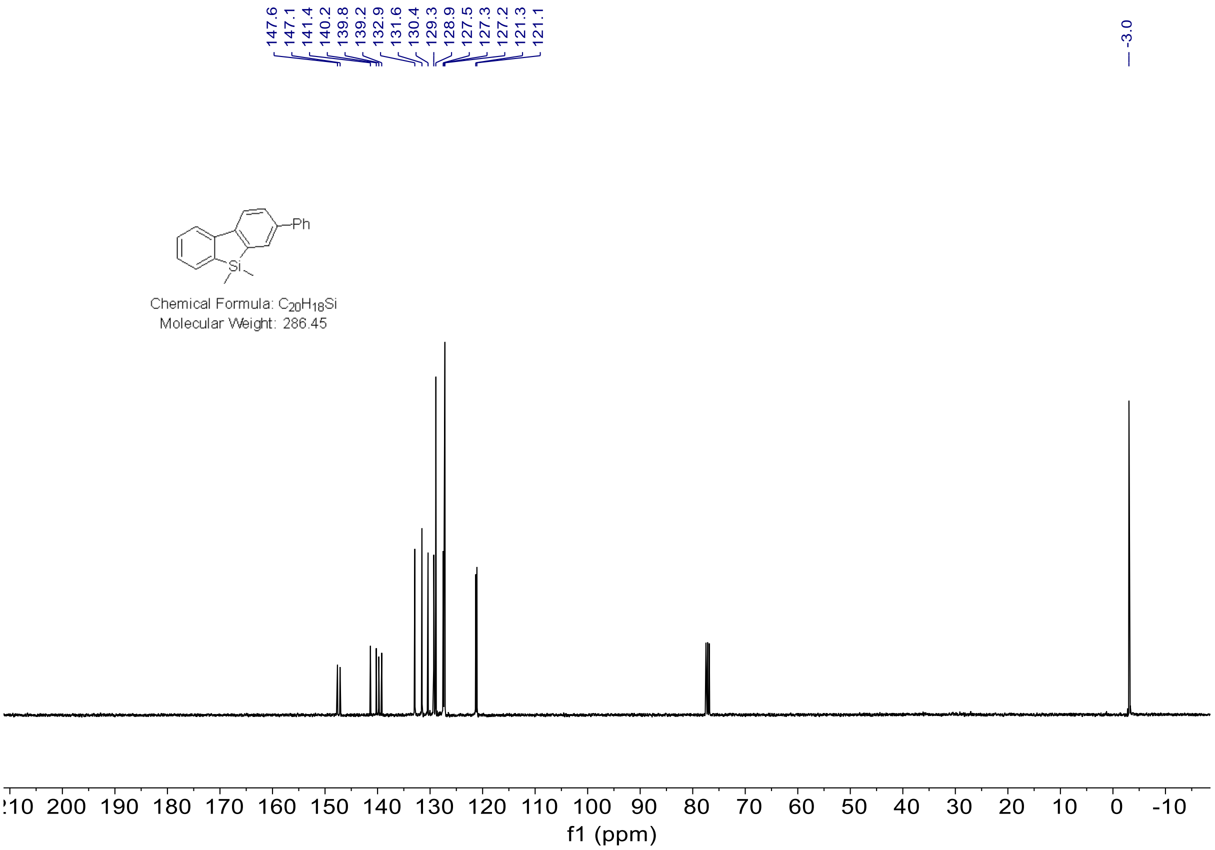

9,9-Dimethyl-2-methyl-9-silafluorene (6c).

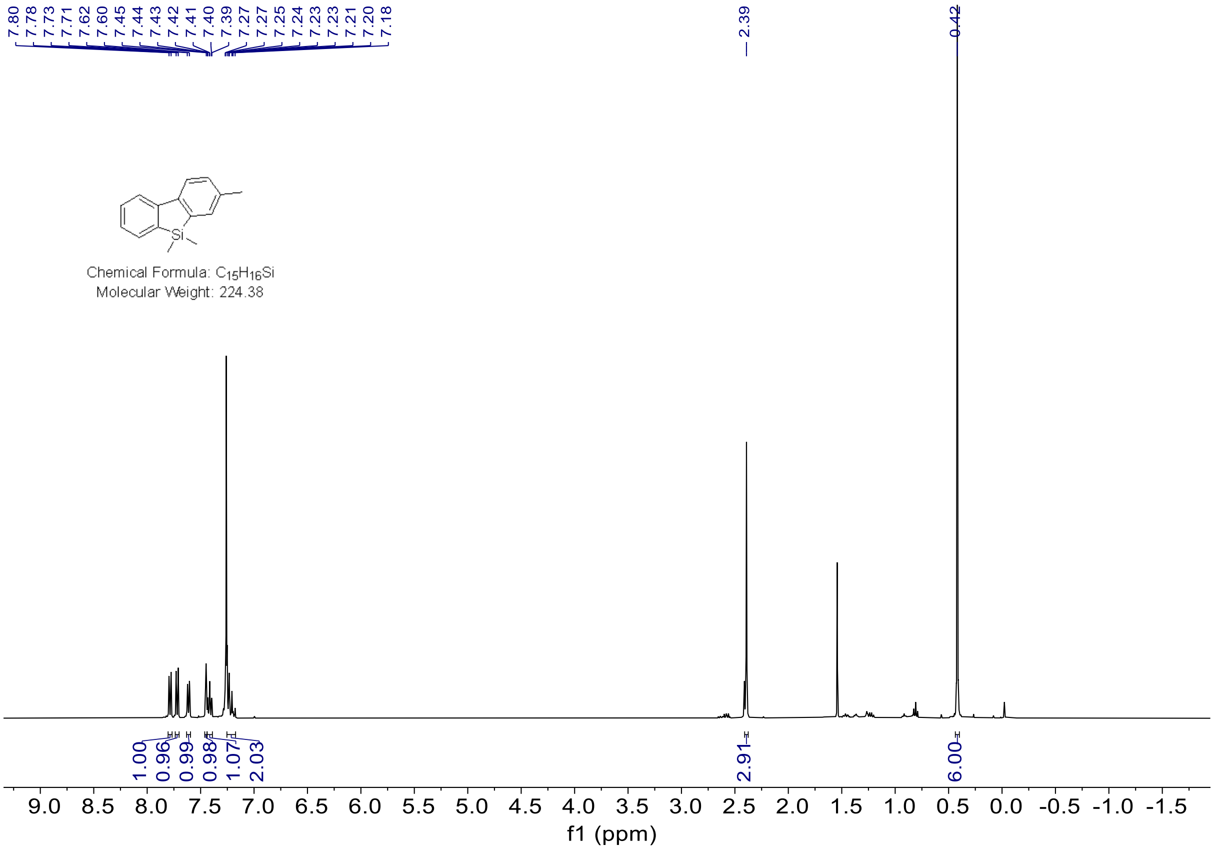

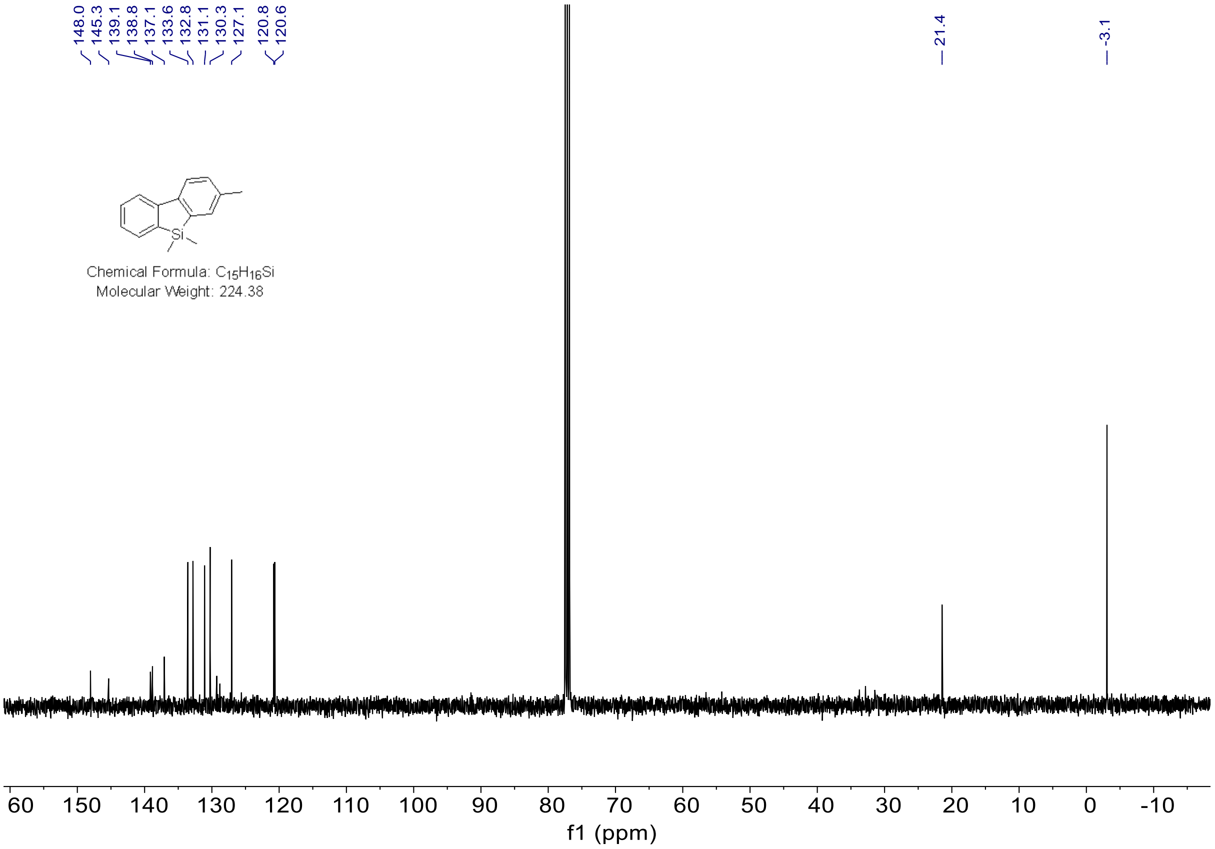

9,9-Dimethyl-2- isopropyl -9-silafluorene (6d).

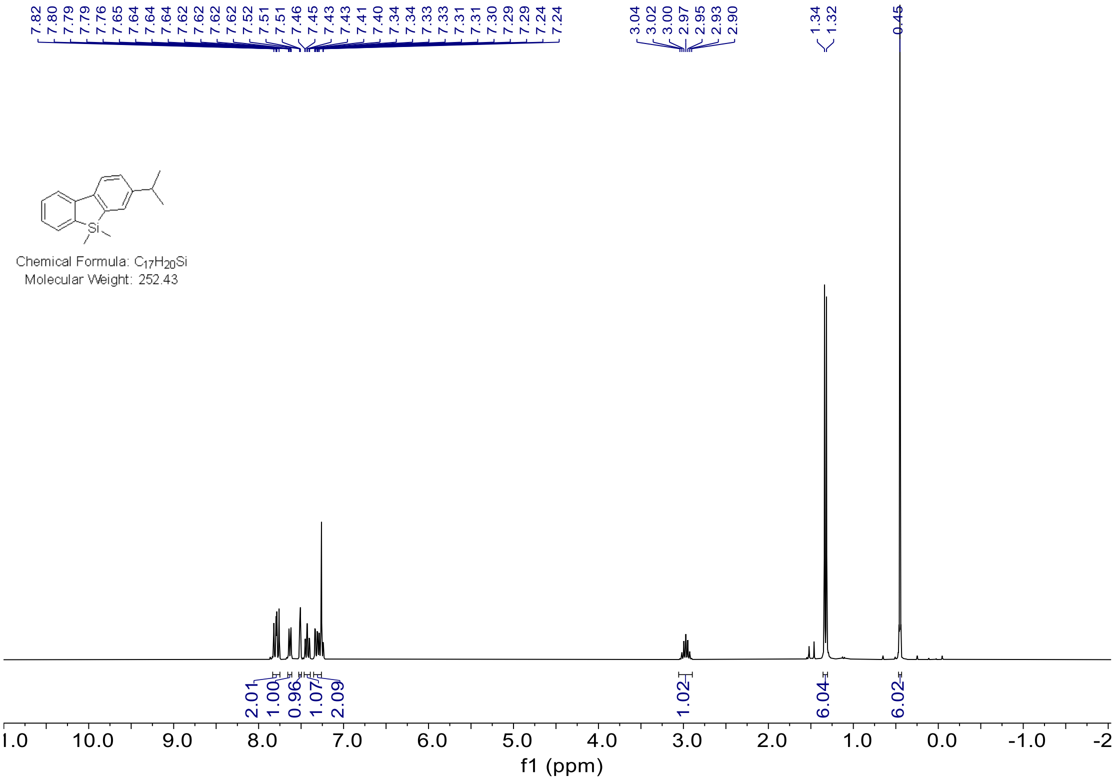

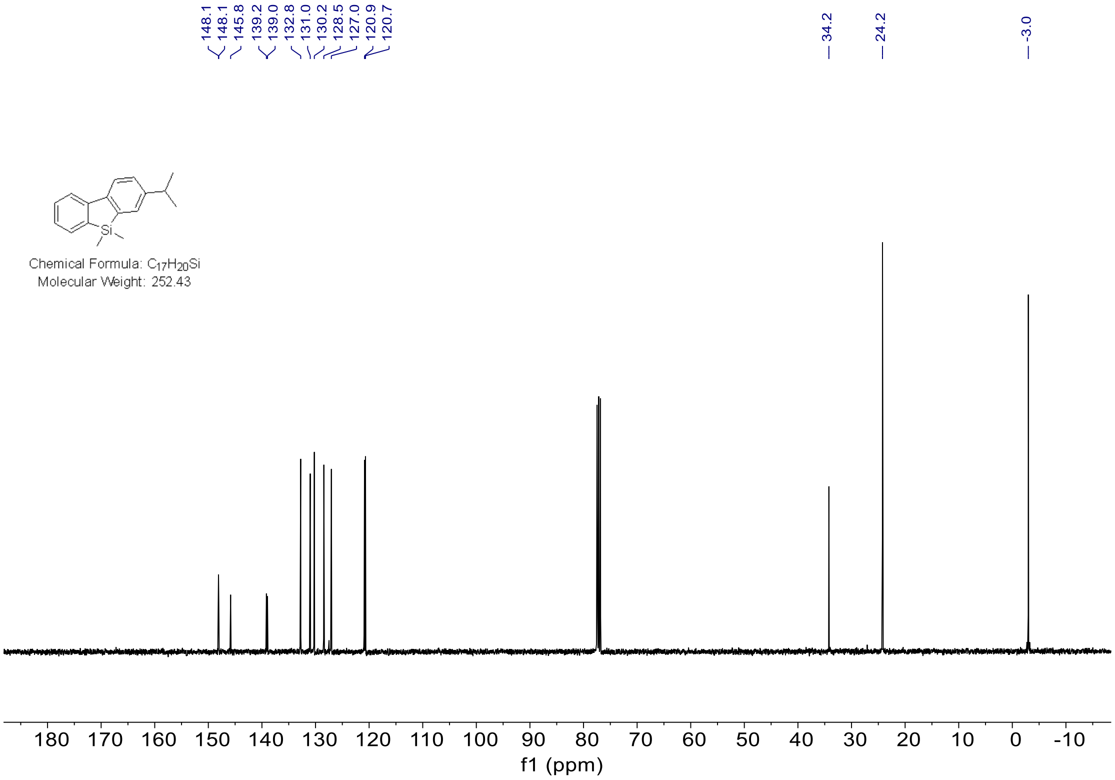

9,9-Dimethyl-2-*tert*-butyl-9-silafluorene (6e).

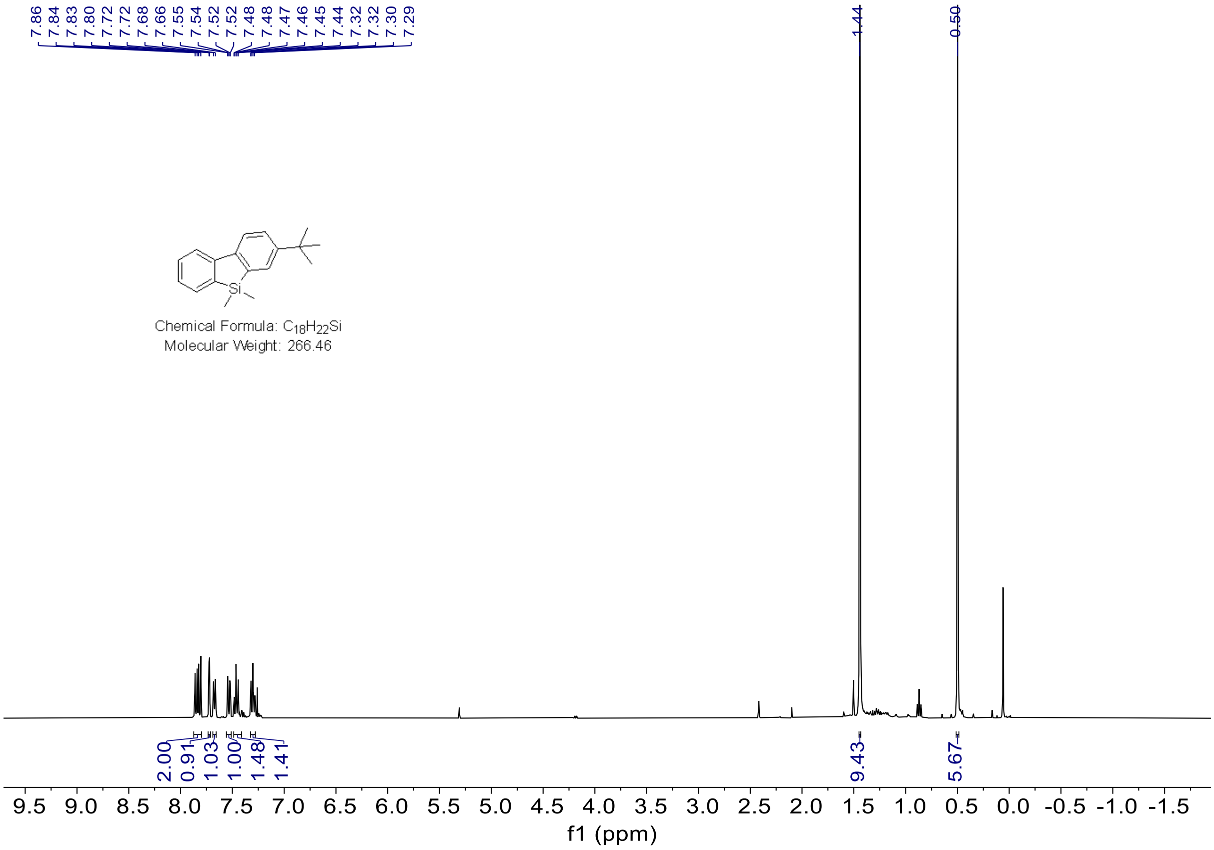

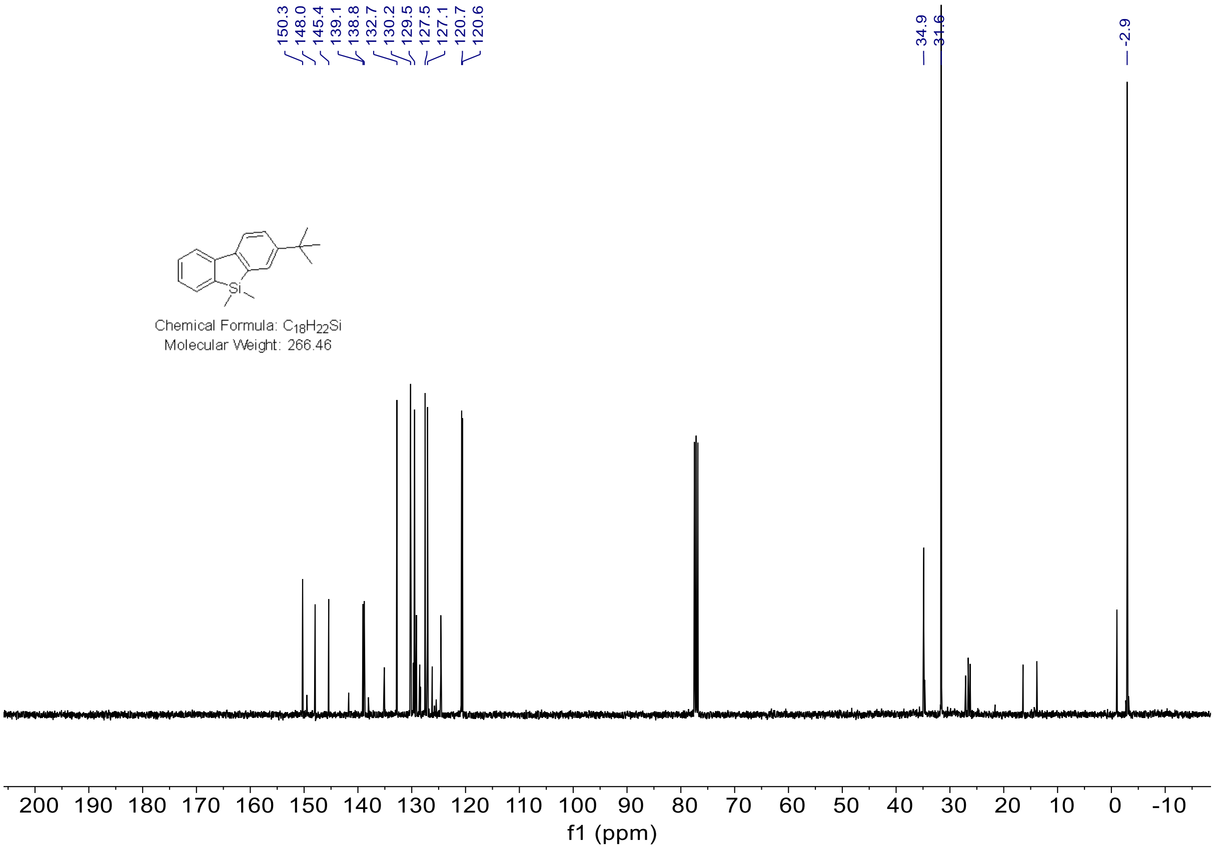

9,9-Dimethyl-2-trifluoromethyl-9-silafluorene (6f).

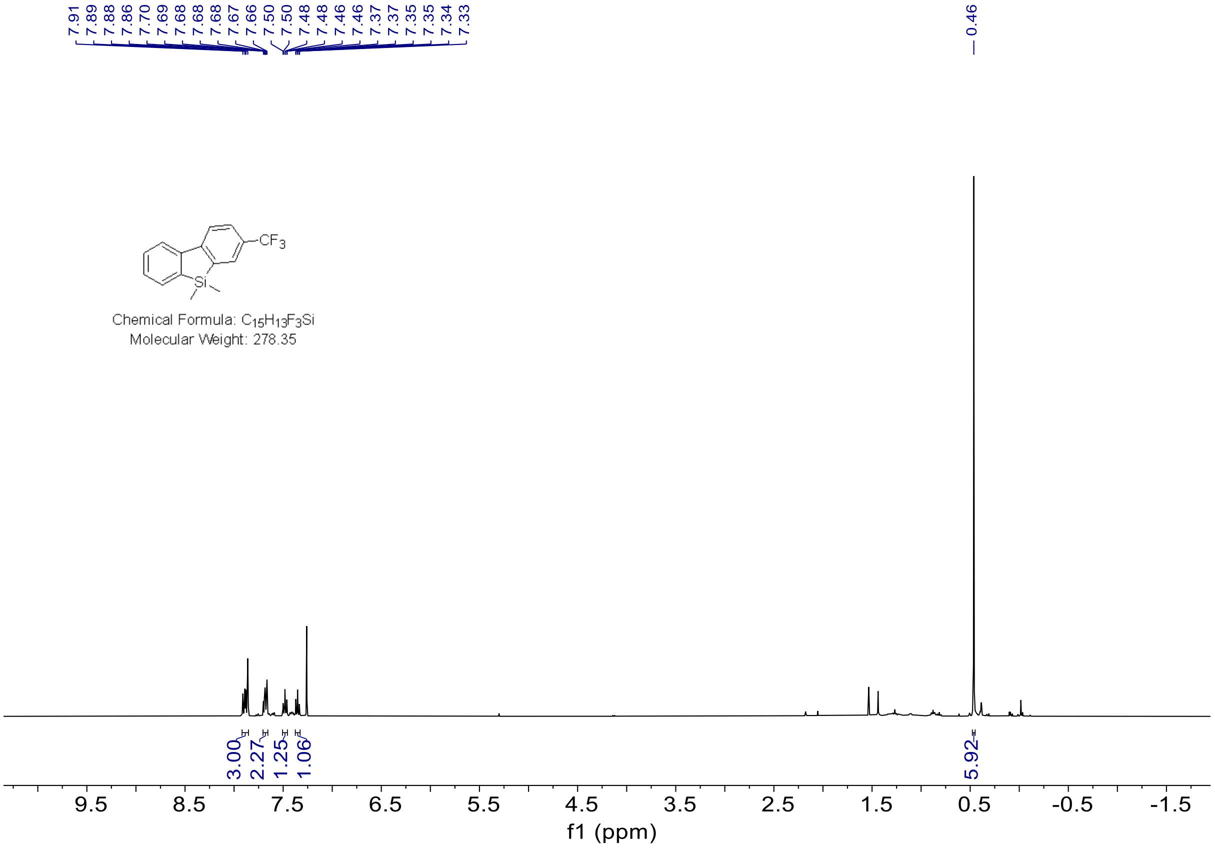

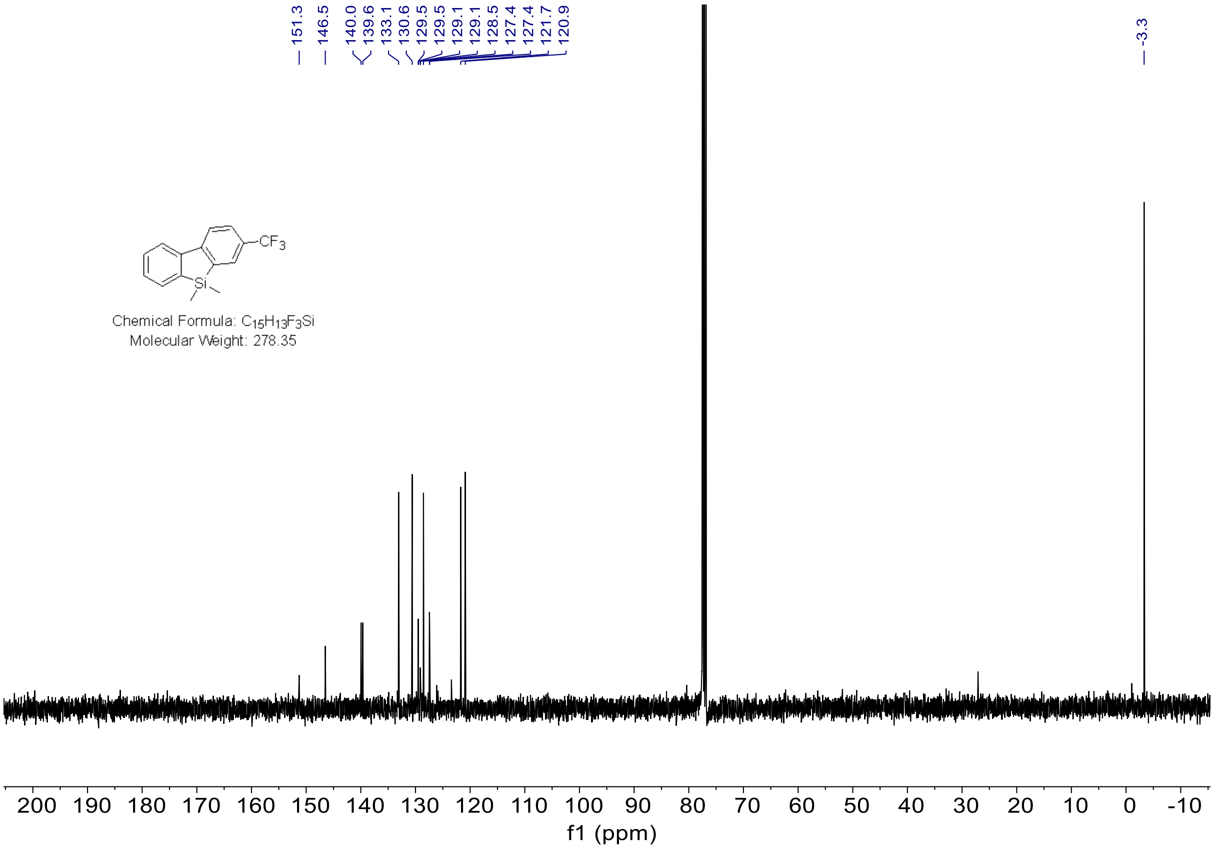

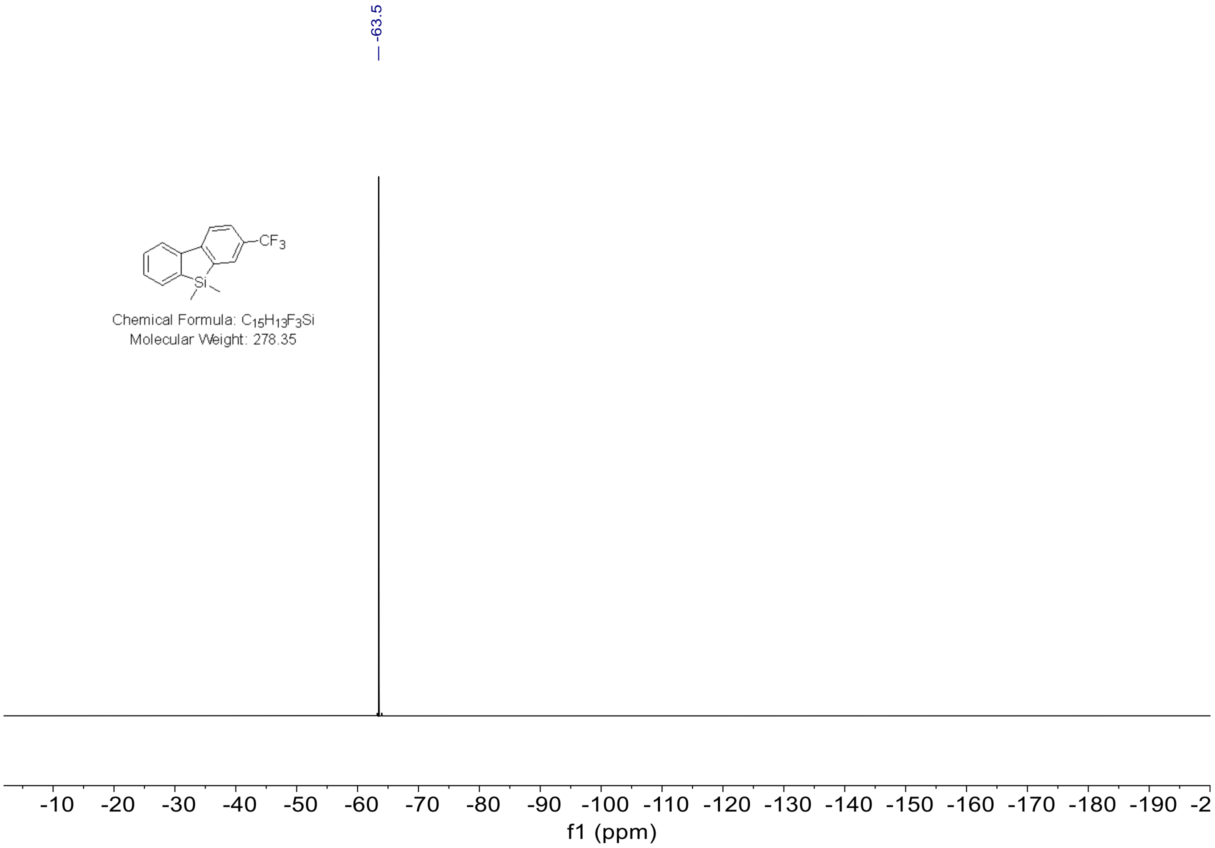

9,9-Dimethyl-2-fluoro-9-silafluorene (6g).

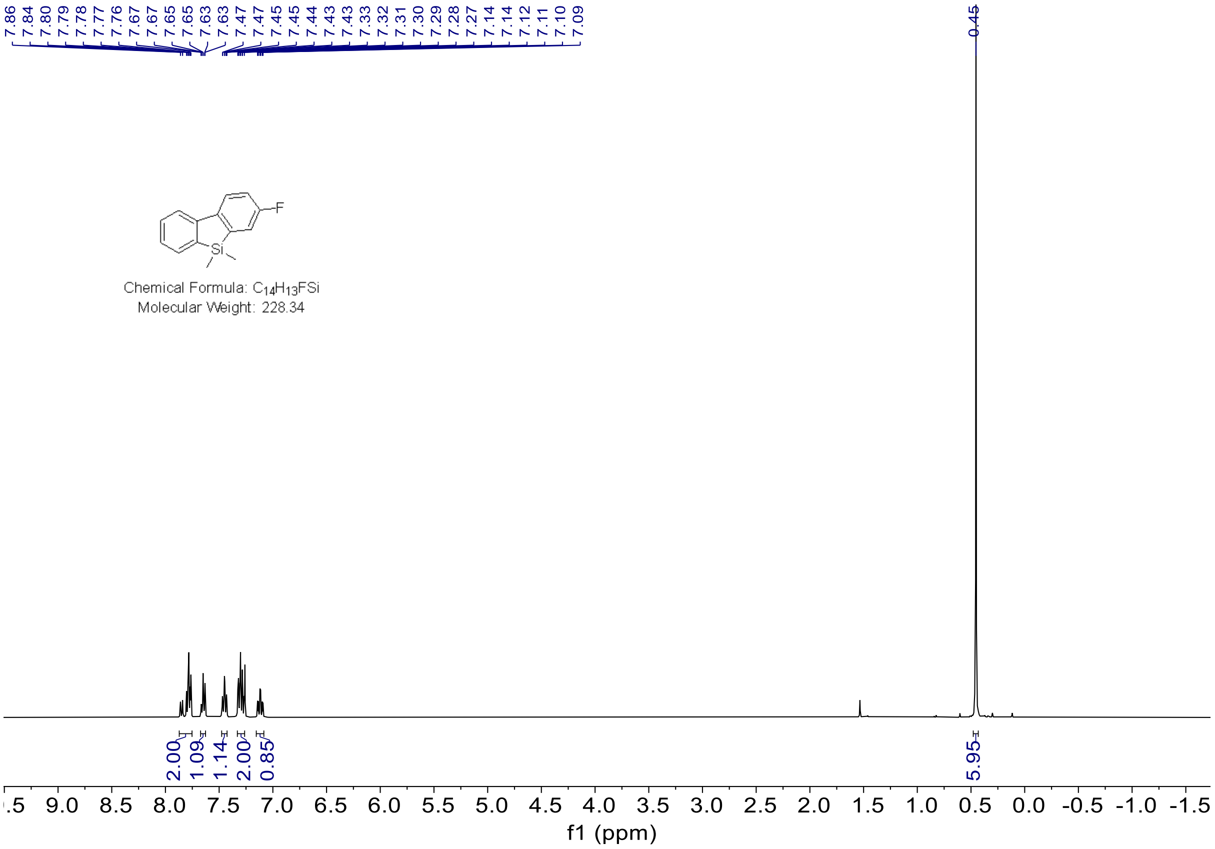

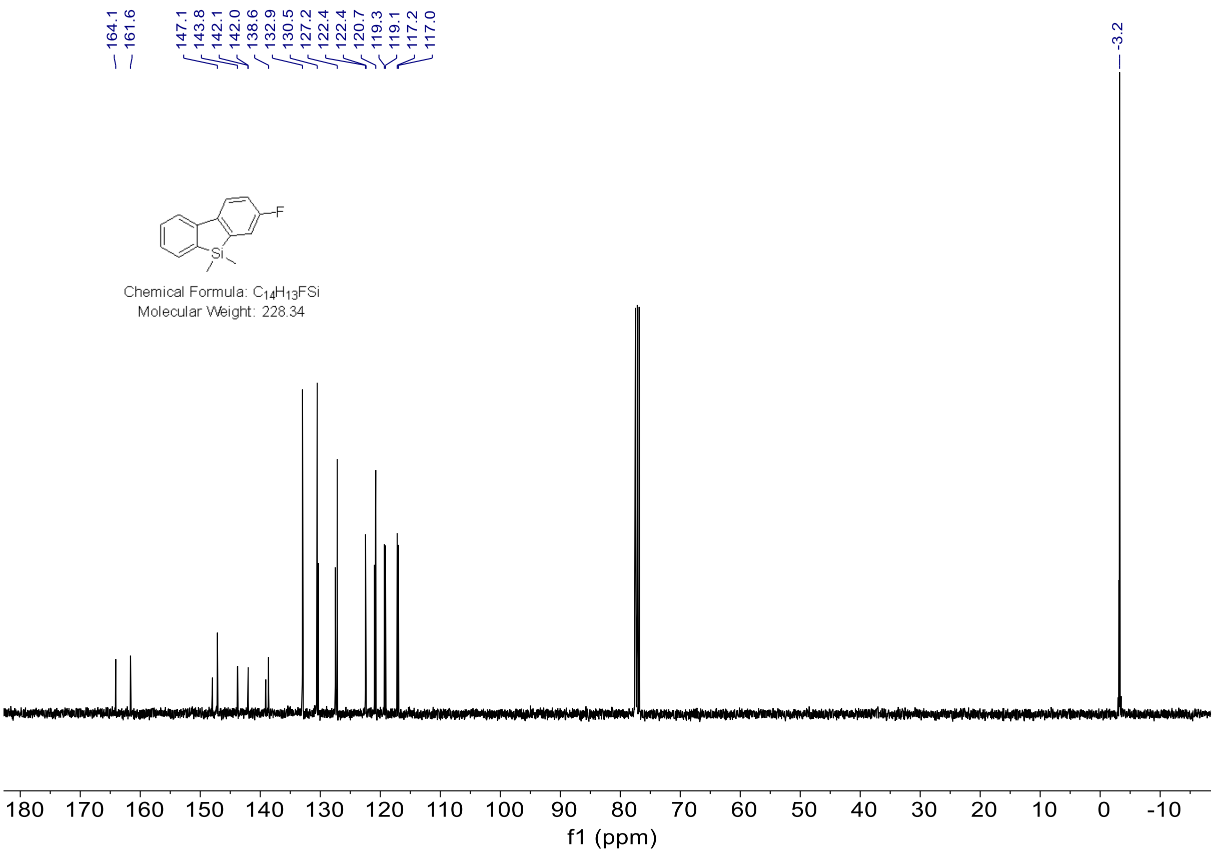
164.1
161.6
147.1
143.8
142.1
142.0
138.6
132.9
130.5
127.2
122.4
122.4
120.7
119.3
119.1
117.2
117.0
-3.2
F
Si
Chemical Formula: $C_{14}H_{13}FSi$
Molecular Weight: 228.34
180 170 160 150 140 130 120 110 100 90 80 70 60 50 40 30 20 10 0 -10
f1 (ppm)

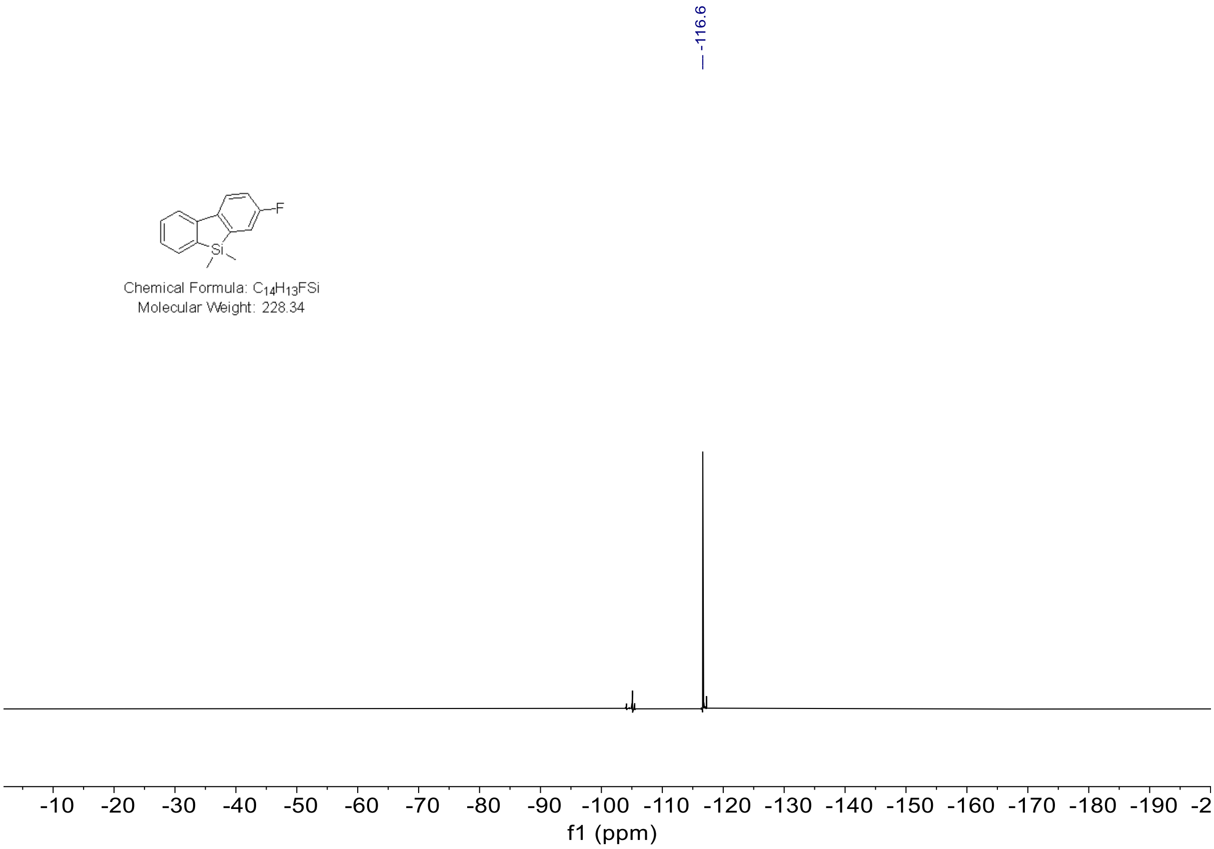
-116.6
F
Si
Chemical Formula: $C_{14}H_{13}FSi$
Molecular Weight: 228.34
-10 -20 -30 -40 -50 -60 -70 -80 -90 -100 -110 -120 -130 -140 -150 -160 -170 -180 -190 -2
f1 (ppm)

5,5-Dimethyl-3-(trifluoromethoxy)-*5H*-dibenzo[*b,d*]silole (6h).

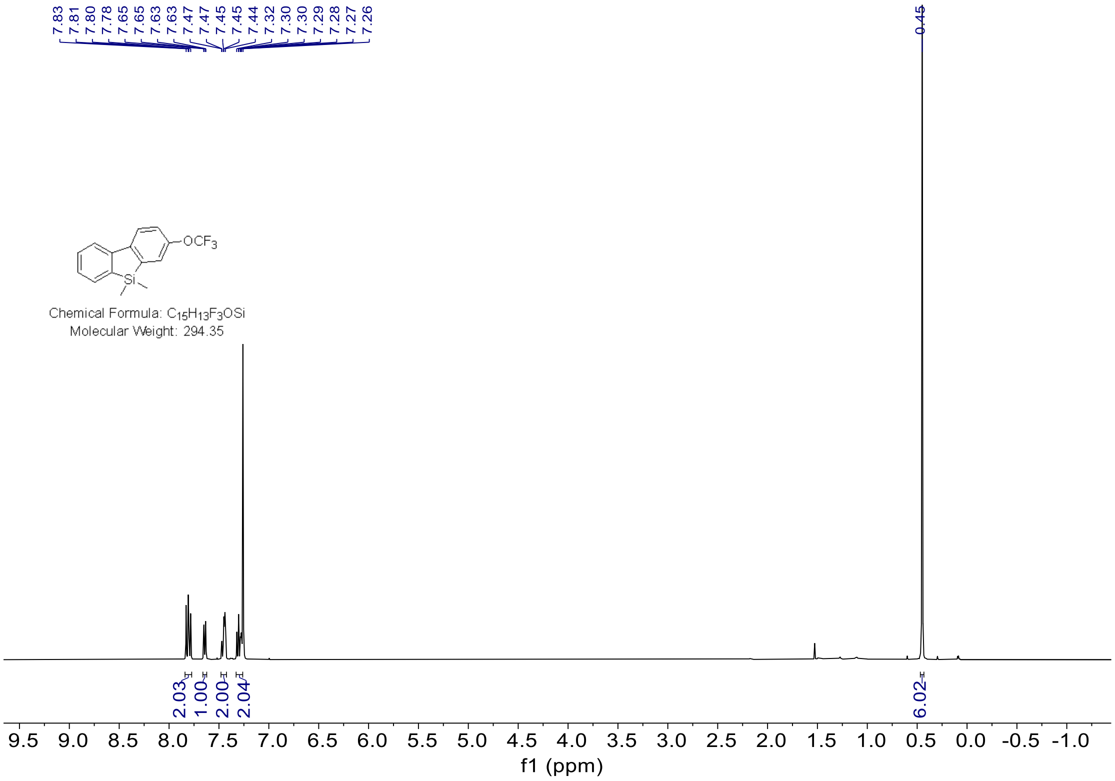

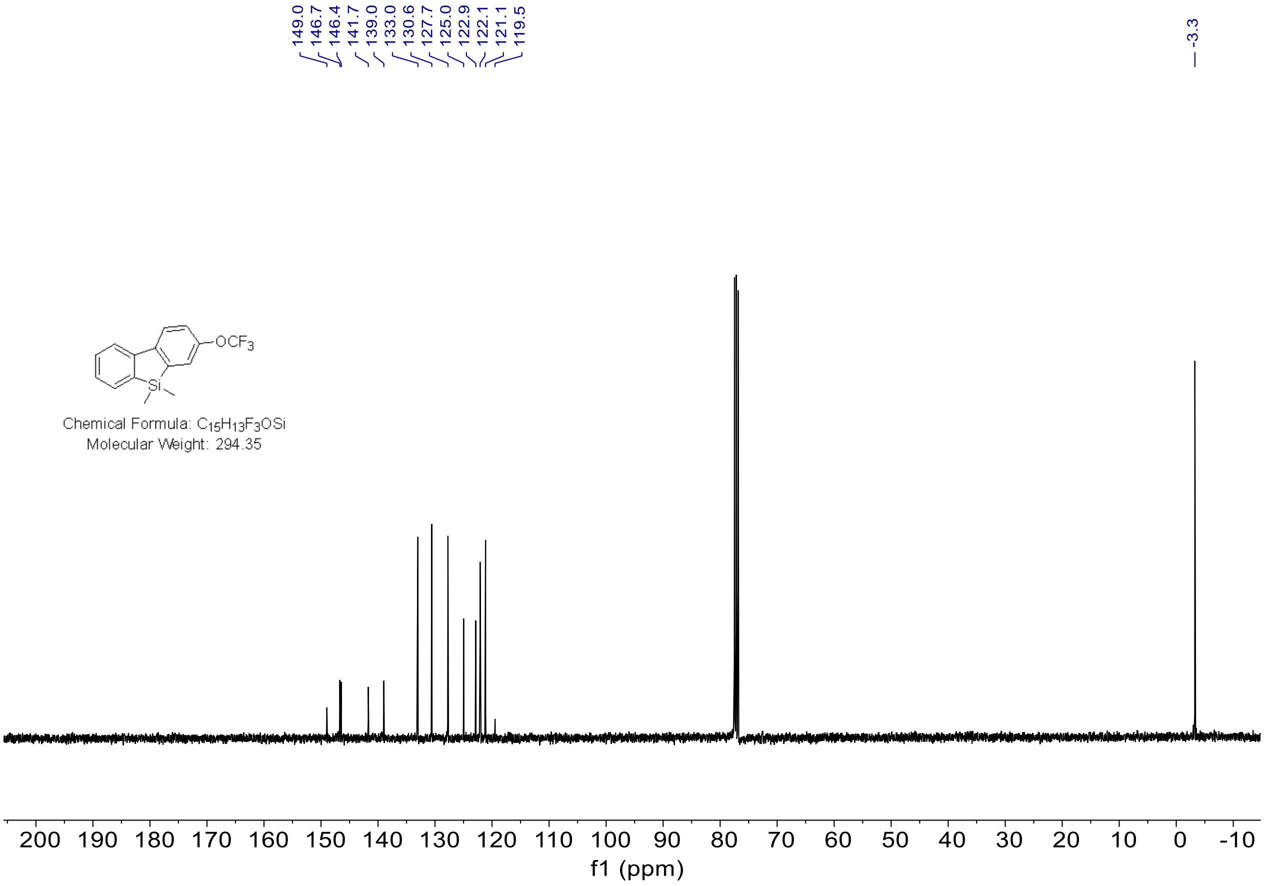

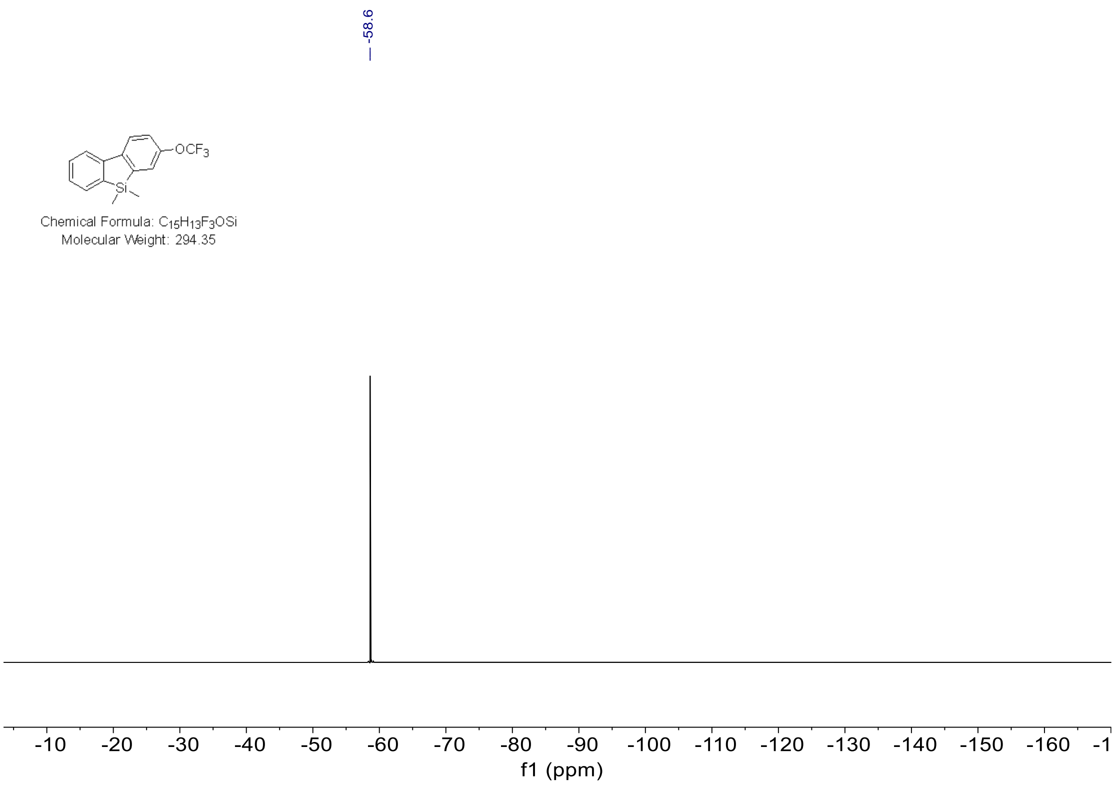

9,9-Dimethyl-4-methyl-9-silafluorene (6i).

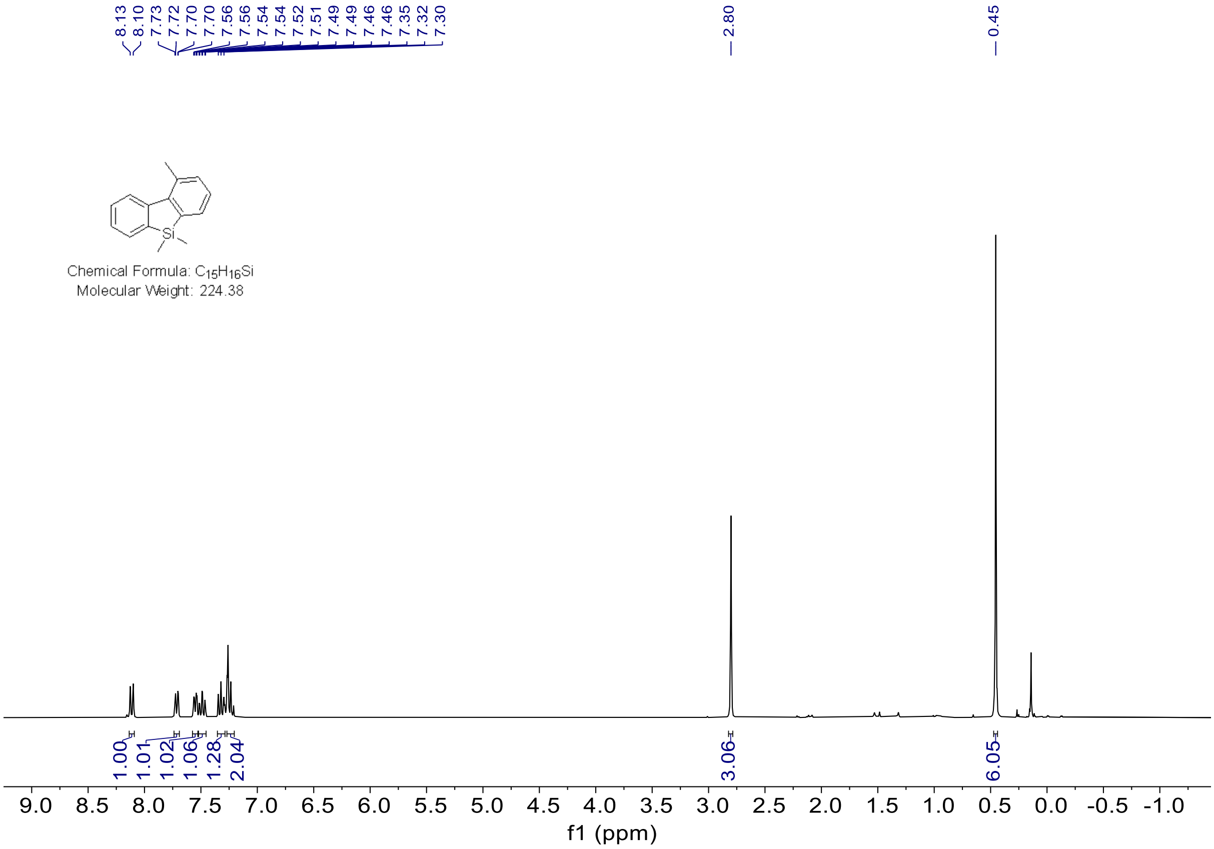

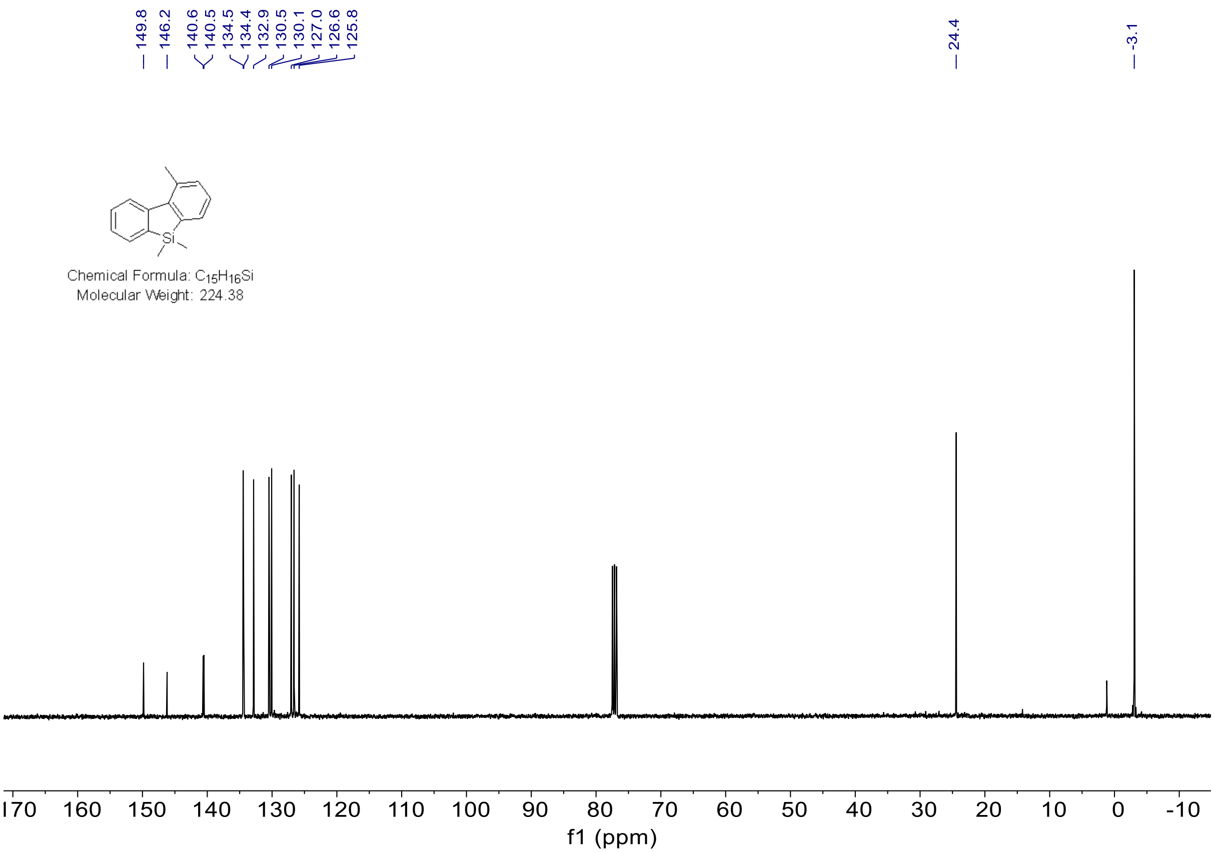

9,9-Dimethyl-4-methoxyl-9-silafluorene (6j).

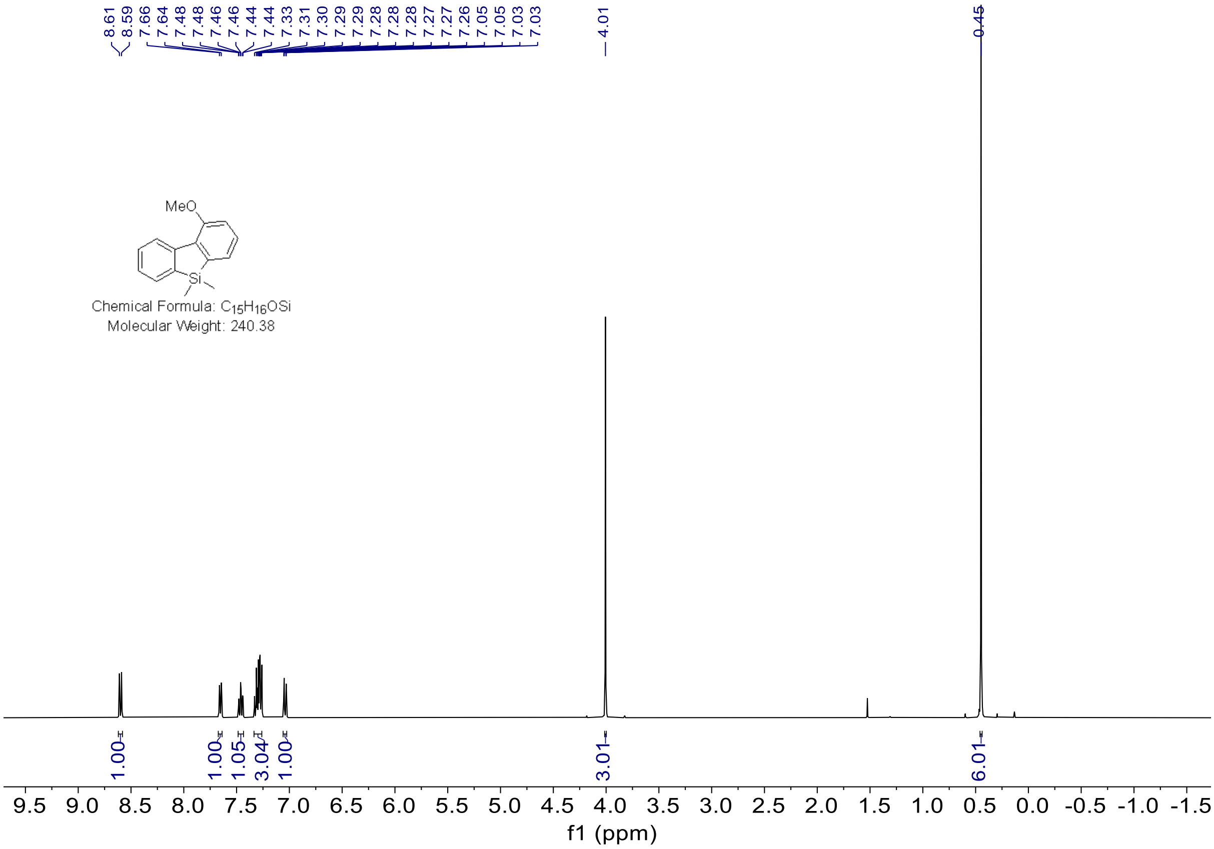

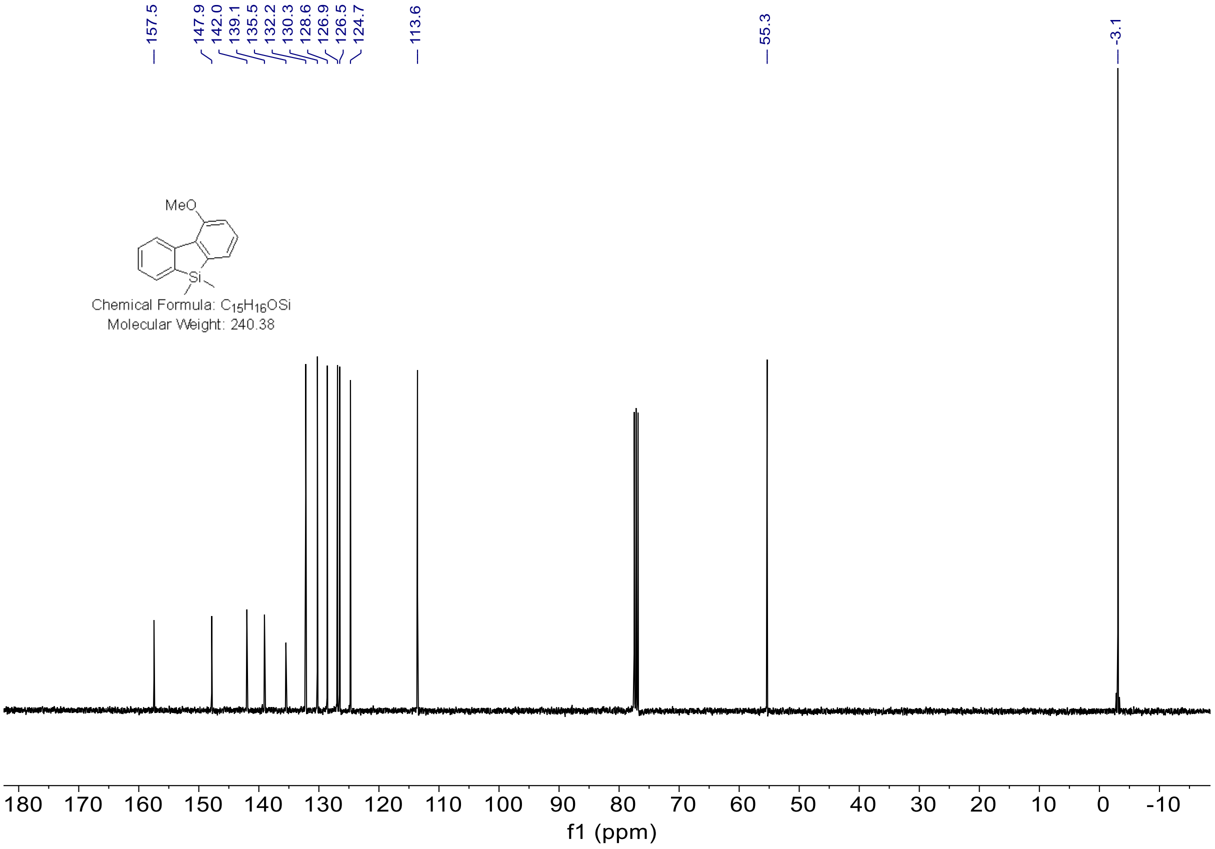

9,9-Dimethyl-4- ethyl -9-silafluorene (6k).

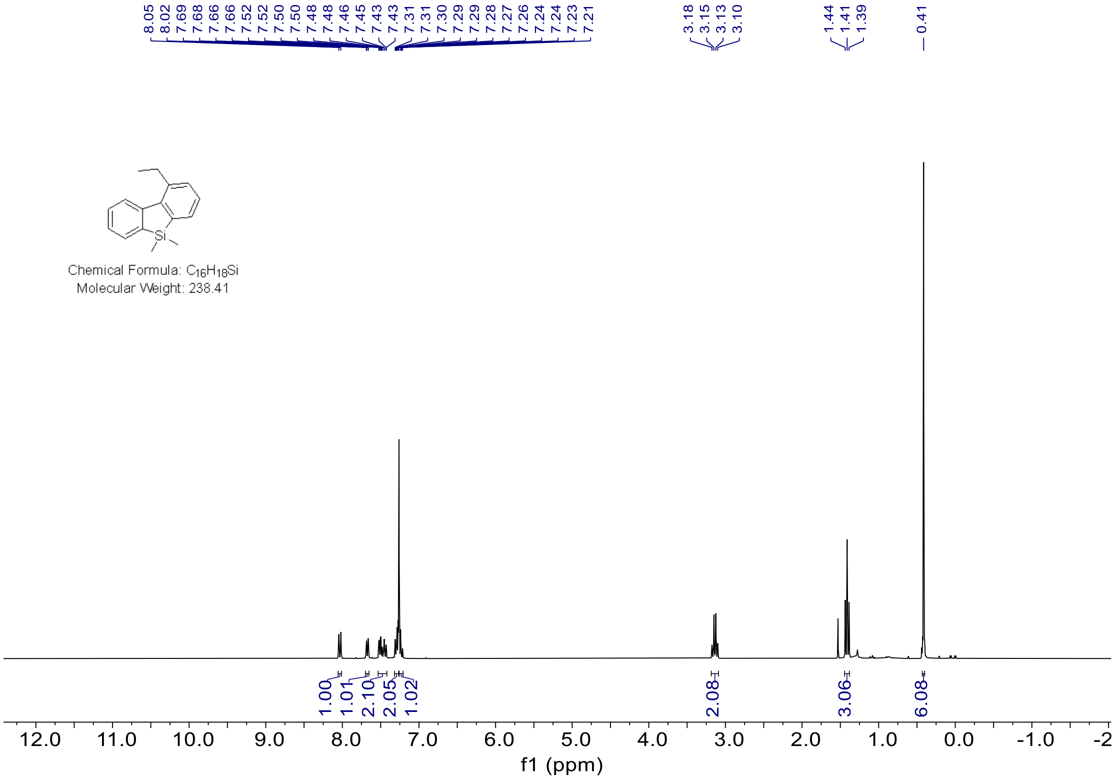

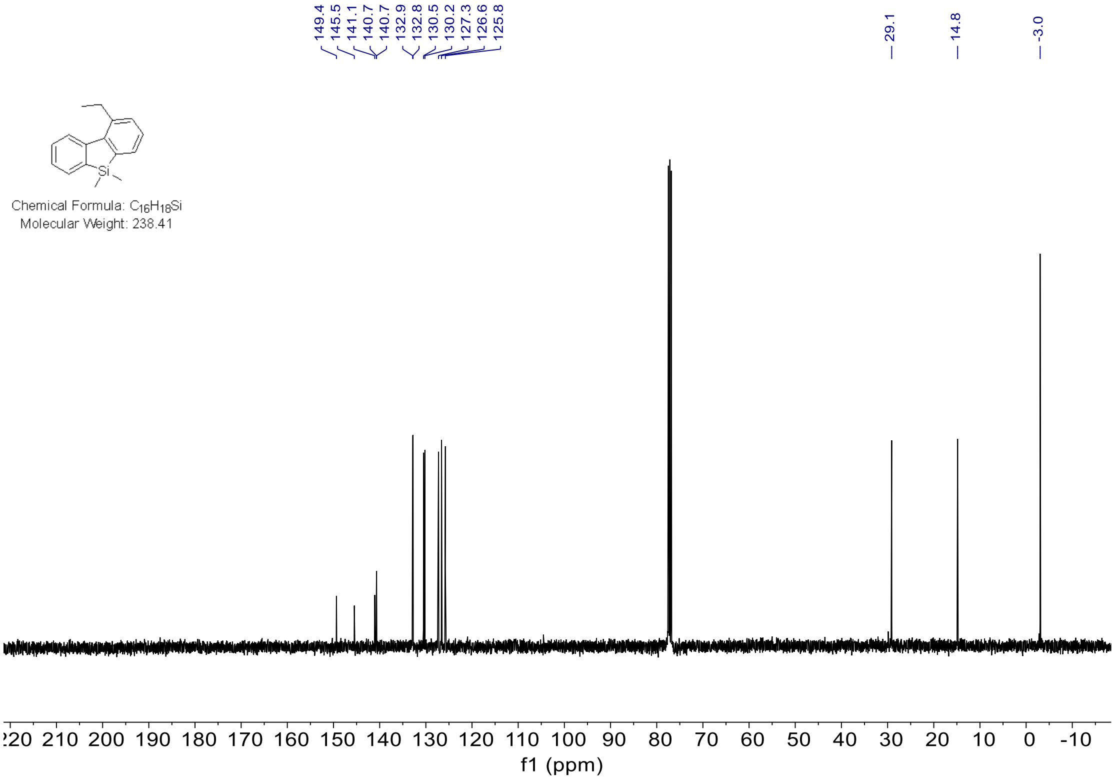

9,9-Dimethyl-3-methyl-9-silafluorene (6l).

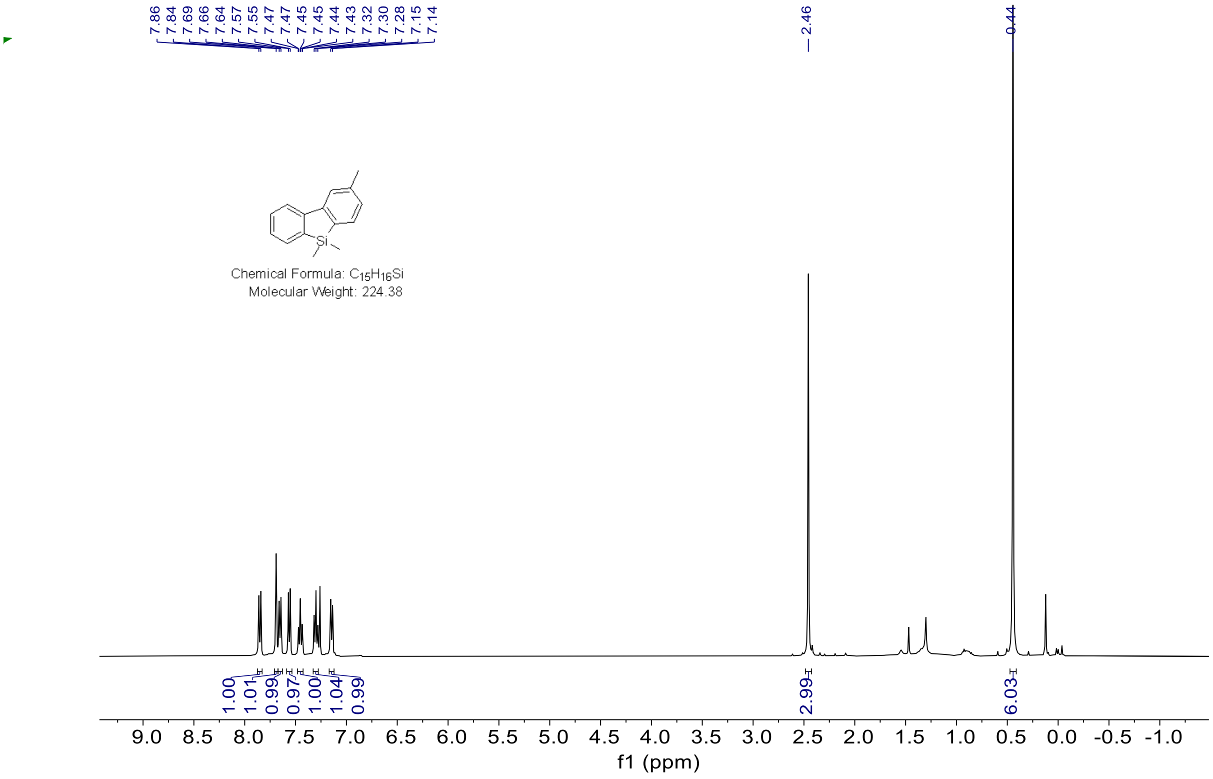

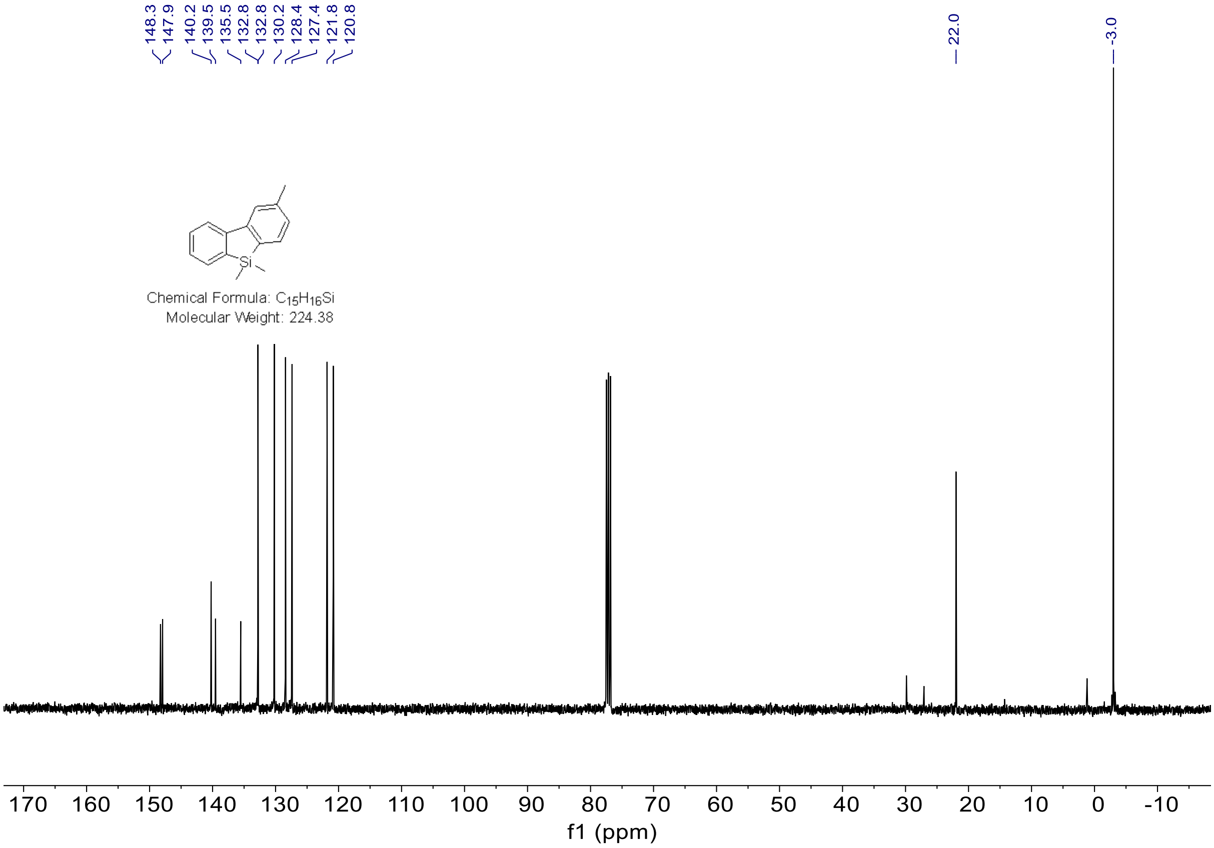

5,5-Dimethyl-2,4-bis(trifluoromethyl)-*5H*-dibenzo[*b,d*]silole (6m).

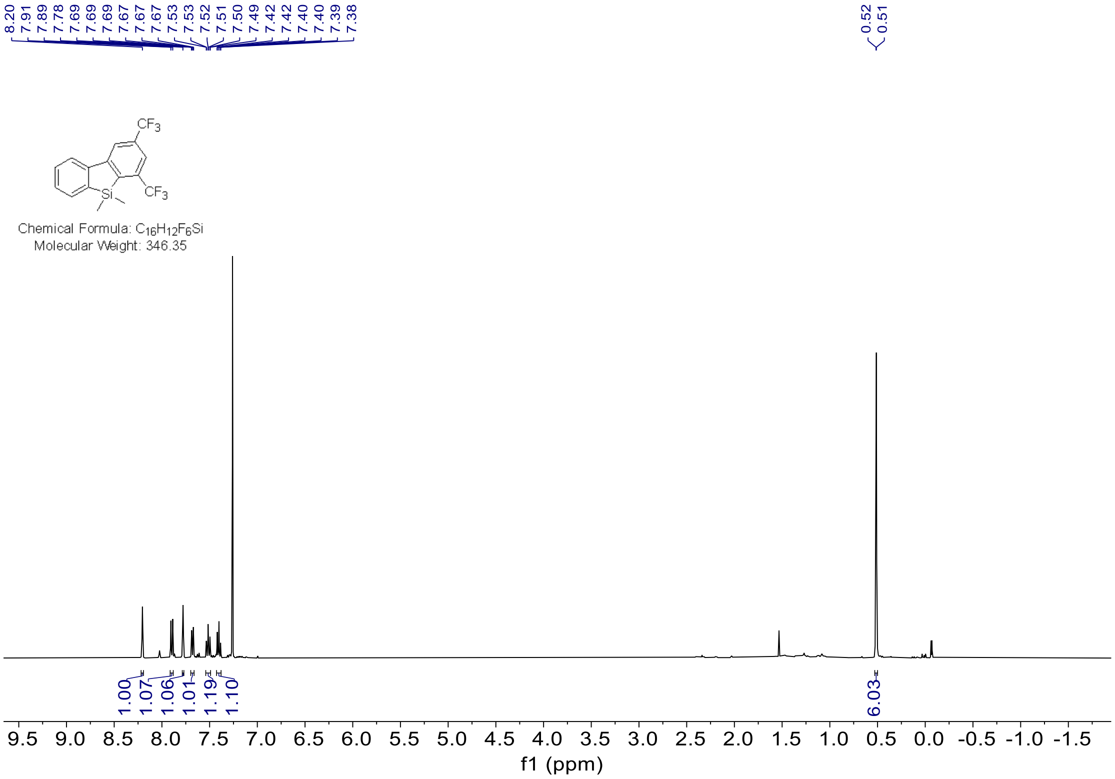

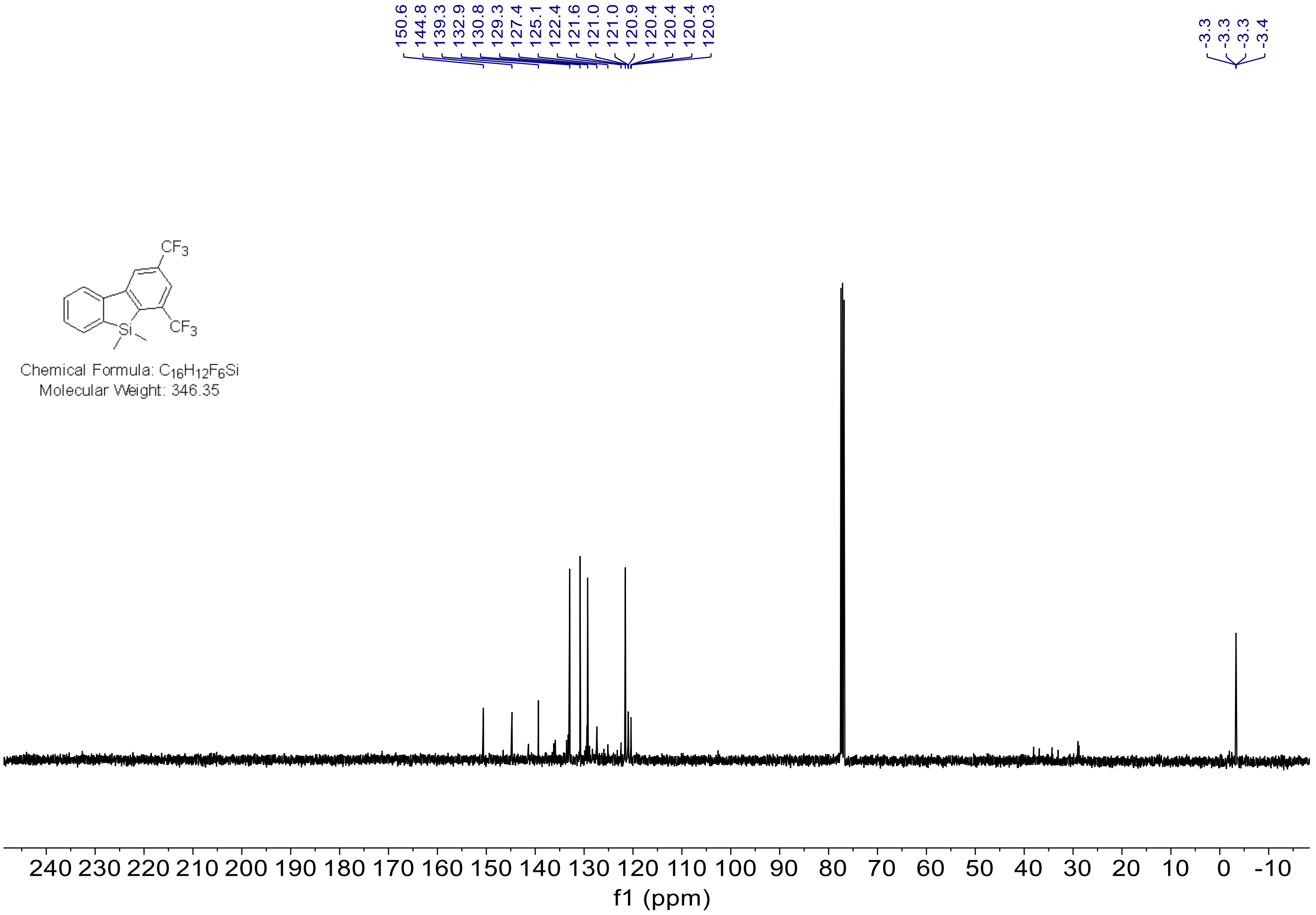
150.6
144.8
139.3
132.9
130.8
129.3
127.4
125.1
122.4
121.6
121.0
120.9
120.4
120.4
120.4
120.3
-3.3
-3.3
-3.3
-3.4
CF3
CF3
Si
Chemical Formula: C16H12F6Si
Molecular Weight: 346.35
240 230 220 210 200 190 180 170 160 150 140 130 120 110 100 90 80 70 60 50 40 30 20 10 0 -10
f1 (ppm)

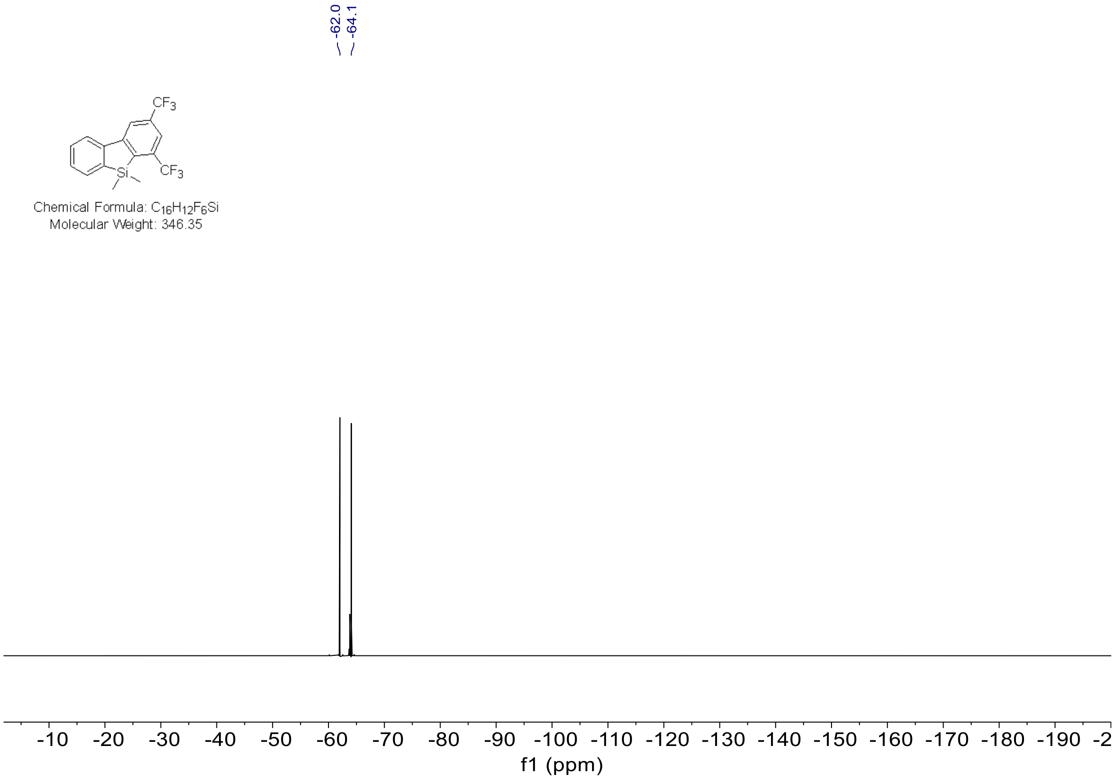
-62.0
-64.1
CF3
CF3
Si
Chemical Formula: C16H12F6Si
Molecular Weight: 346.35
-10 -20 -30 -40 -50 -60 -70 -80 -90 -100 -110 -120 -130 -140 -150 -160 -170 -180 -190 -2
f1 (ppm)

11,11-Dimethyl-*11H*-benzo[*b*]silafluorene (6n).

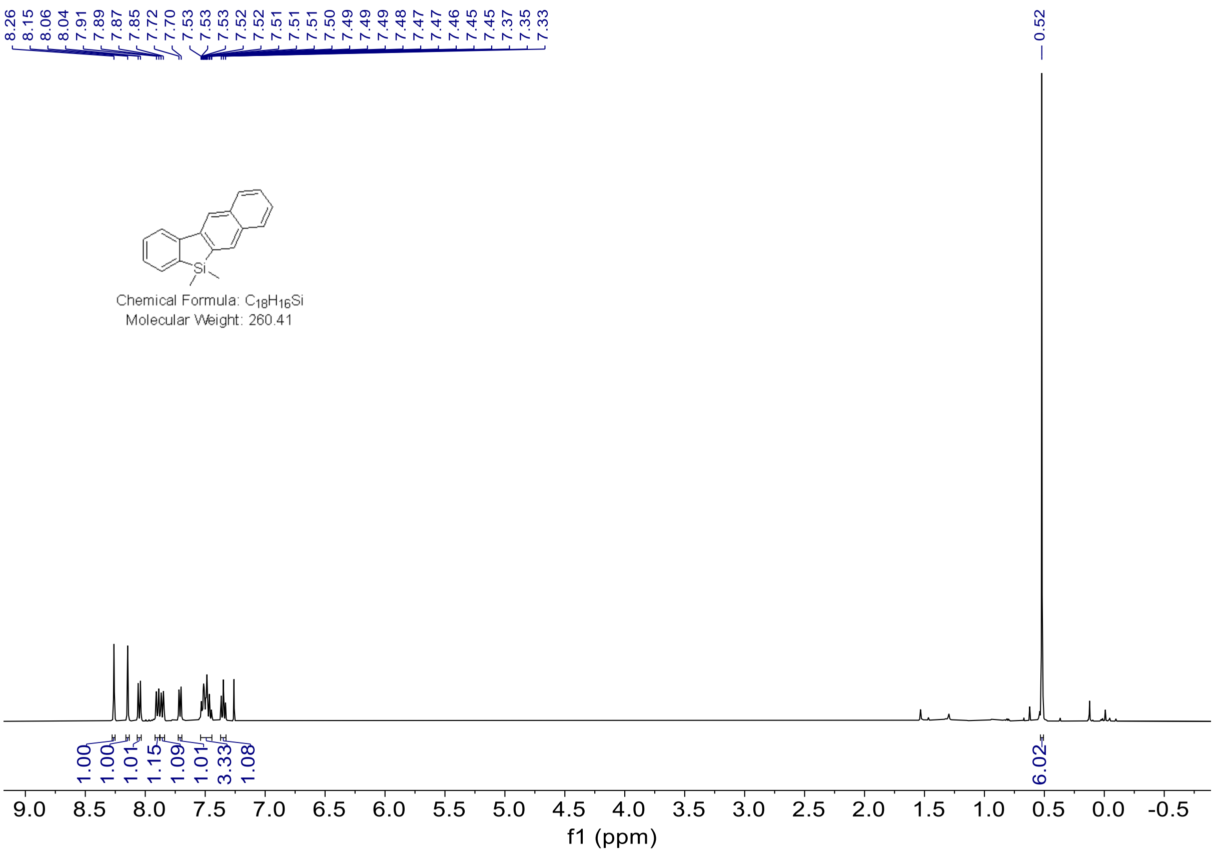

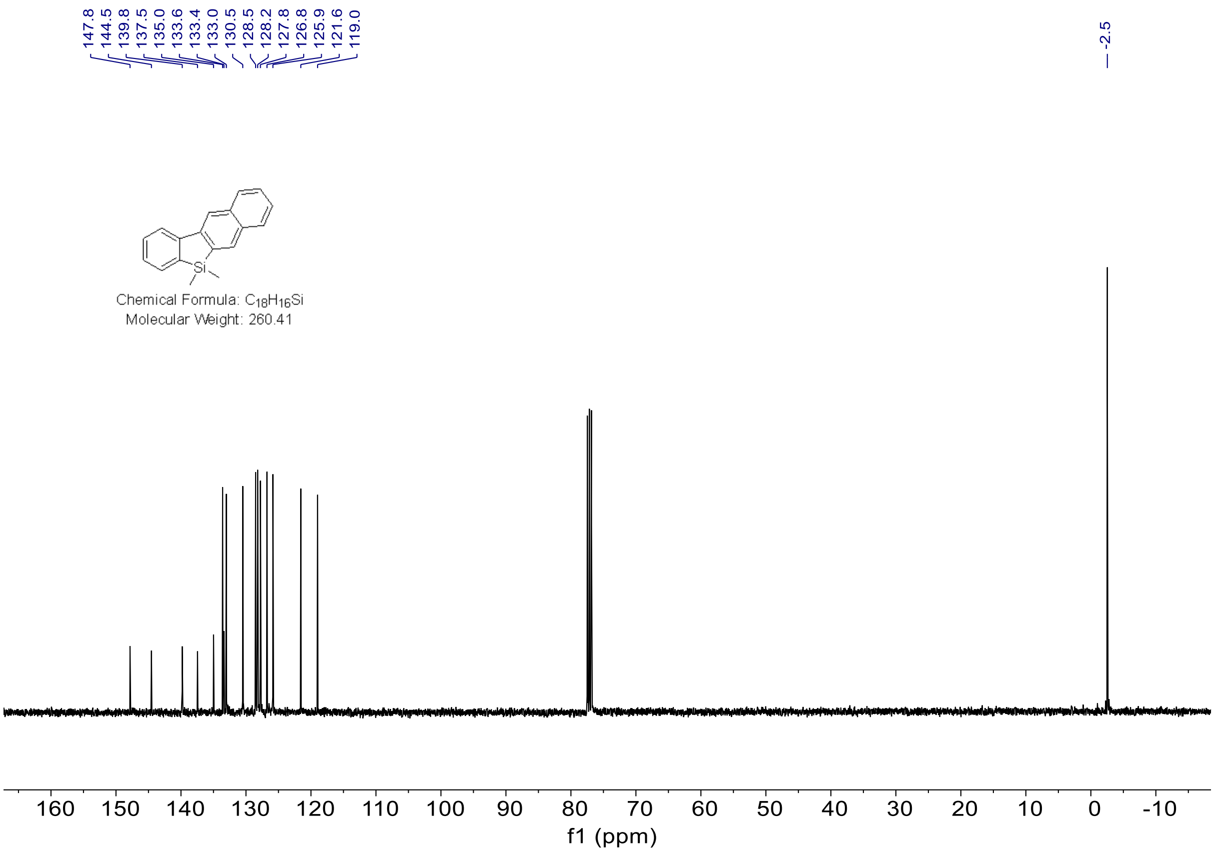

8-Methoxy-5,5-dimethyl-*5H*-benzo[*b*]naphtho[*2,3-d*]silole (6o).

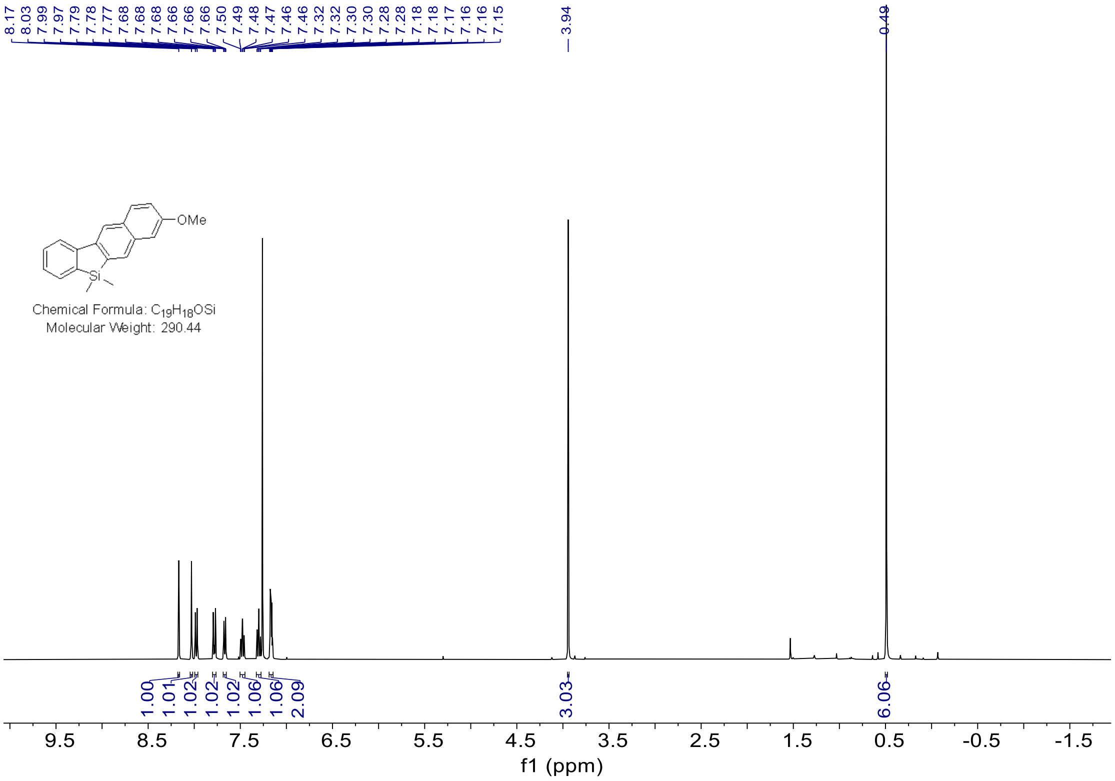

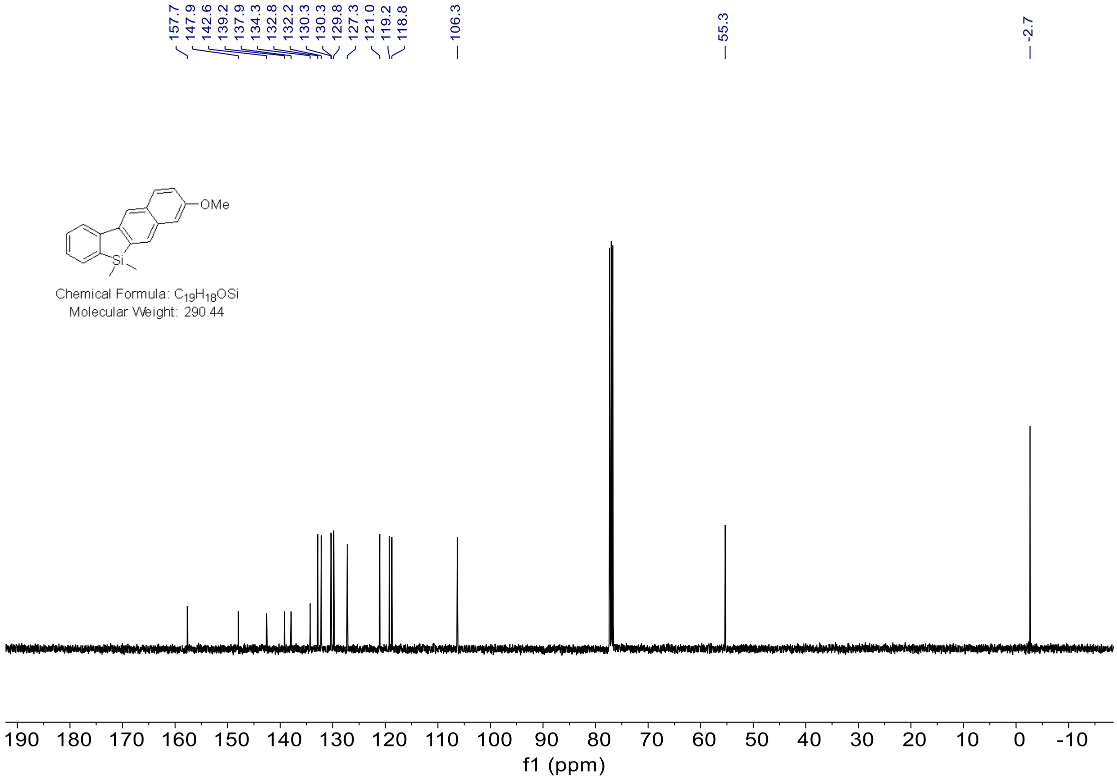

8,8-Dimethylbenzosilolo[*2,3-a*]ferrocene (6p).

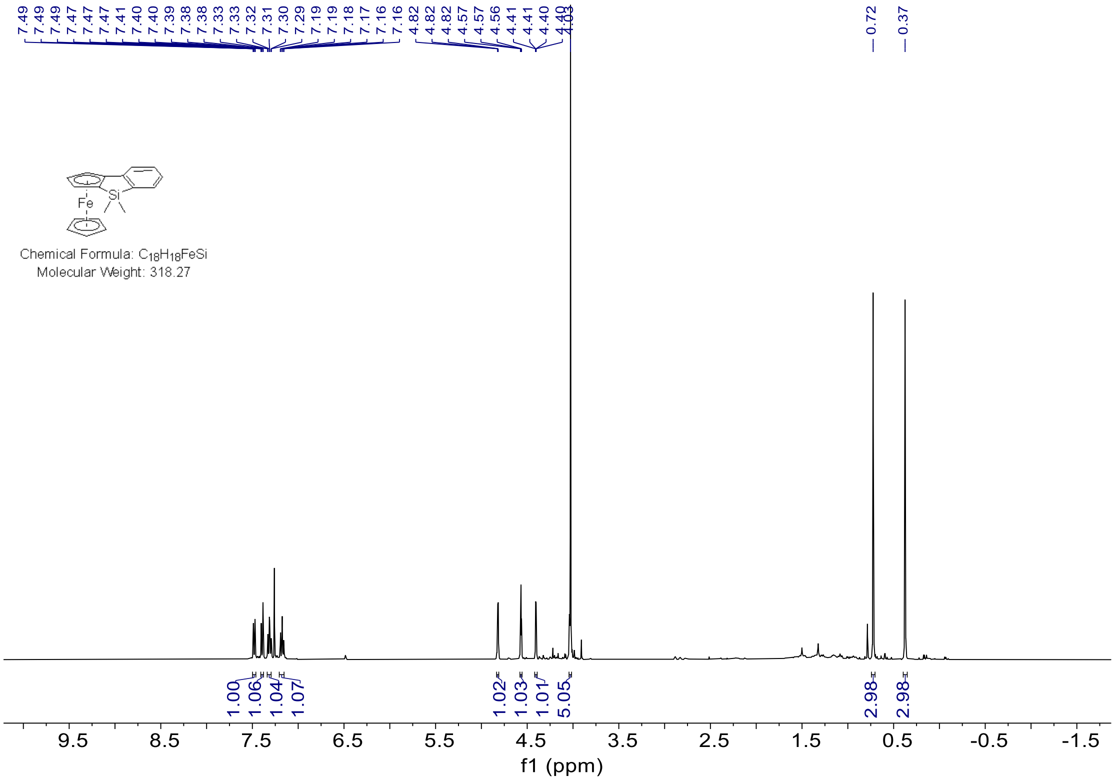

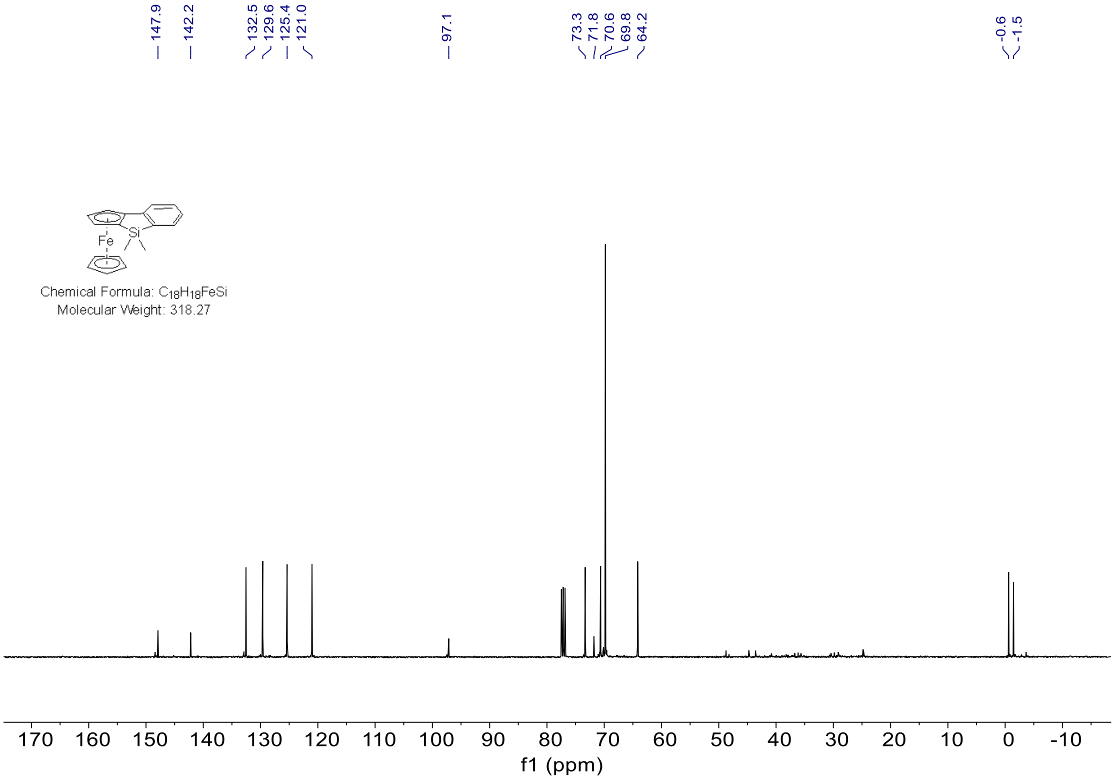

7,7-Dimethyl- *7H*-benzo[*b*]naphtho[*1,2-d*]silole (6q).

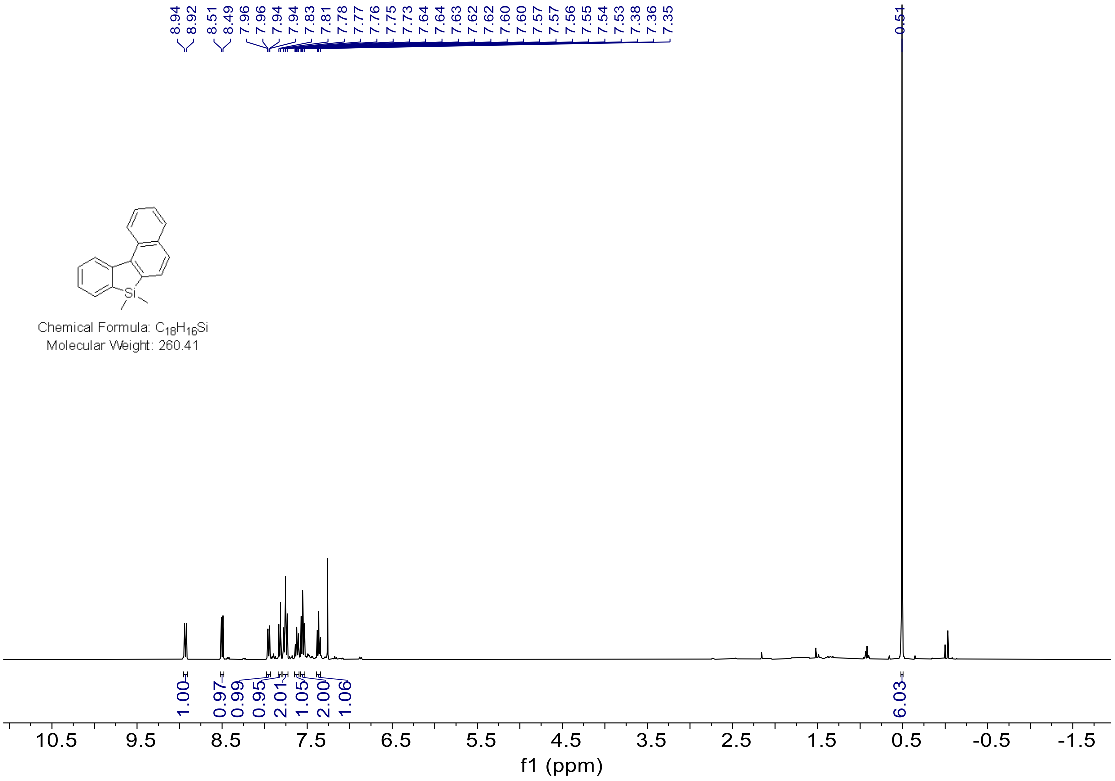

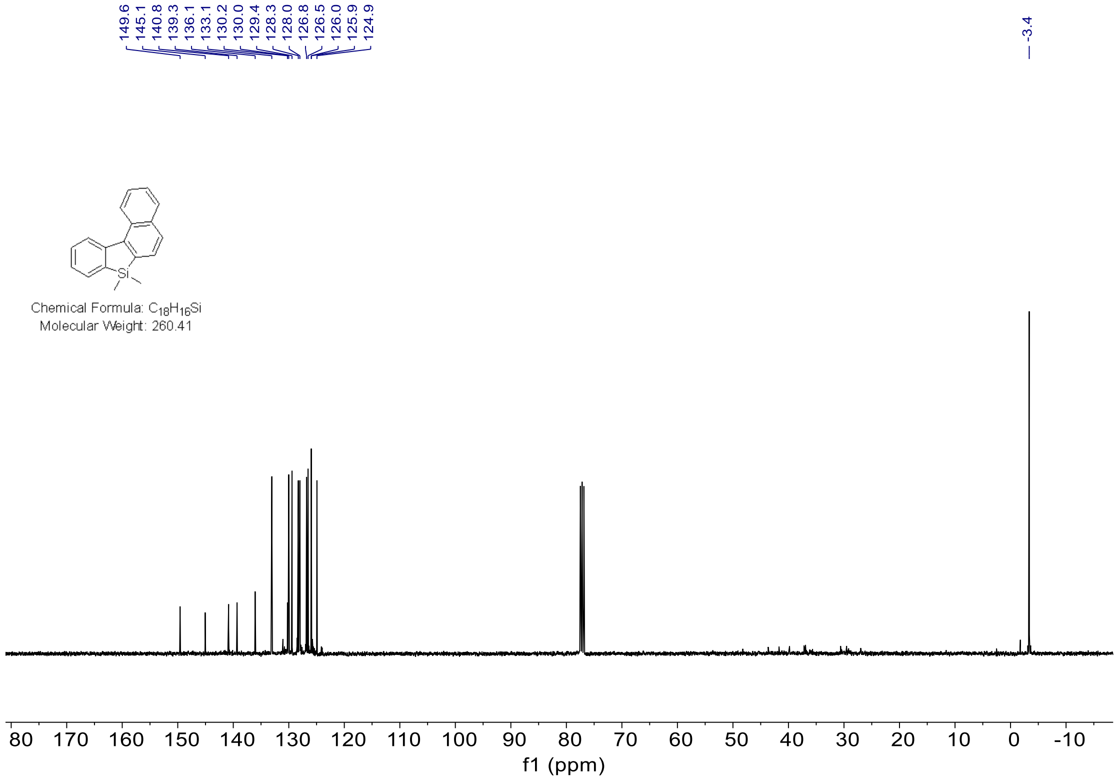

5,5,5',5'-Tetramethyl-*5H,5'H*-3,3'-bidibenzo[*b,d*]silole (6r).

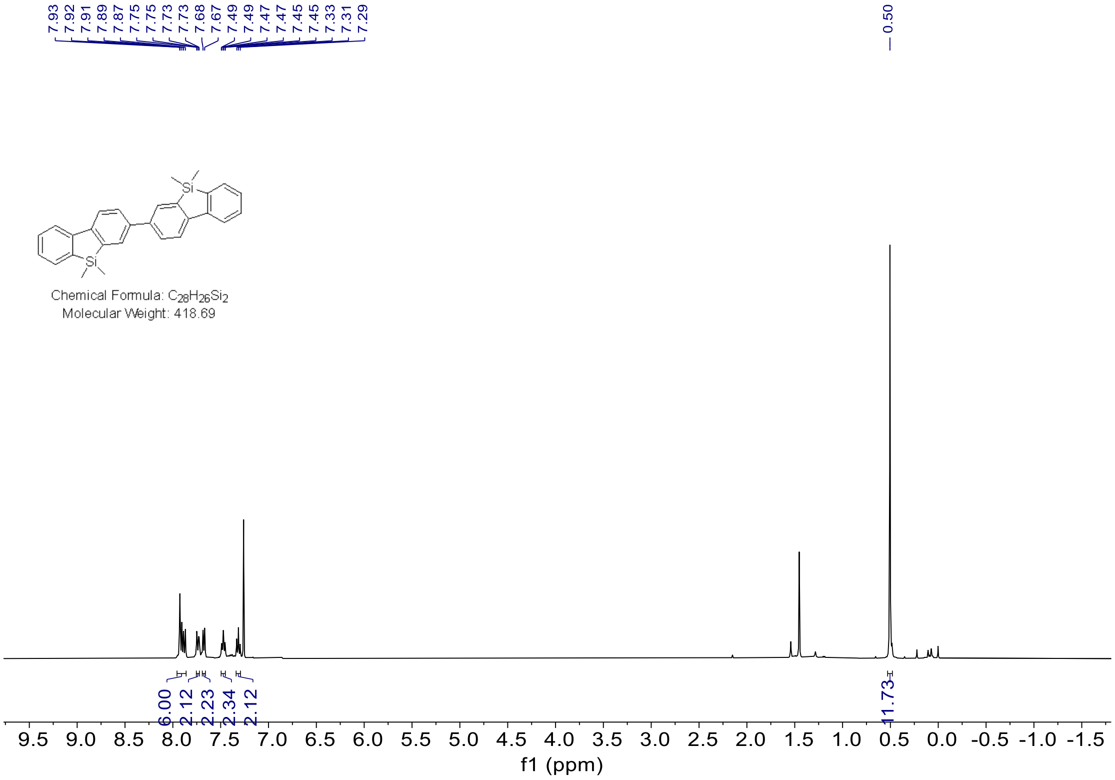

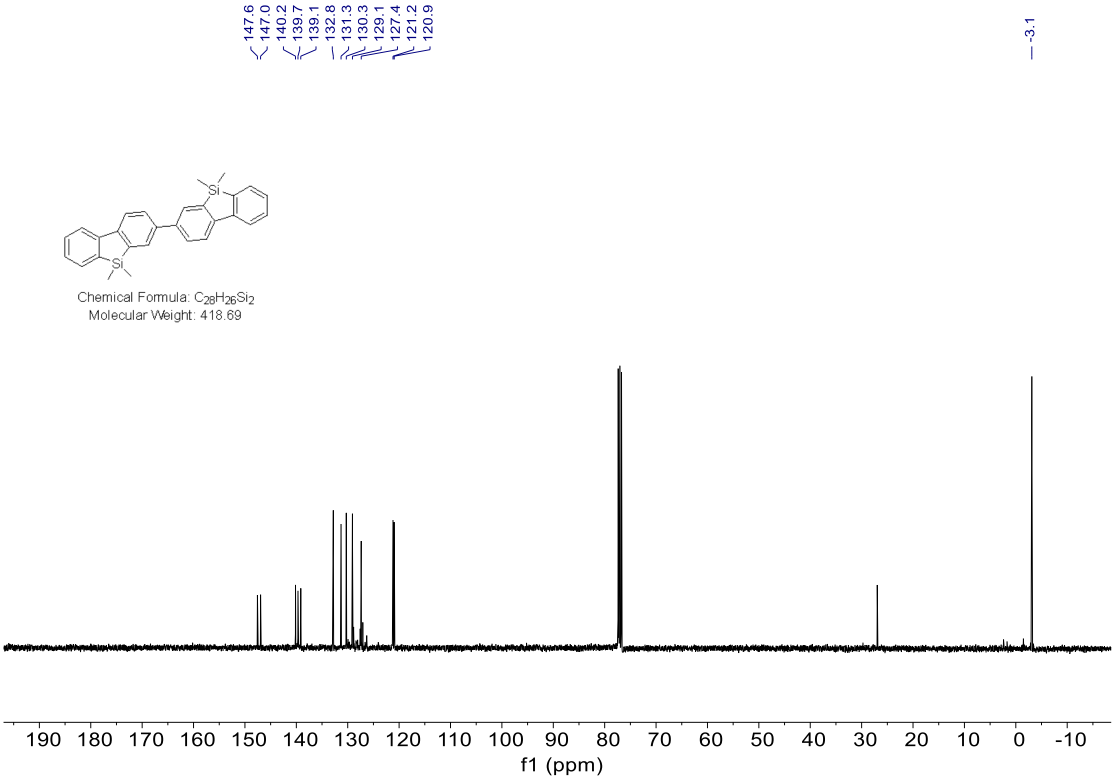

6,6,12,12-Tetramethyl-6,12-disilaindeno[*1,2-b*]fluorene (6s).

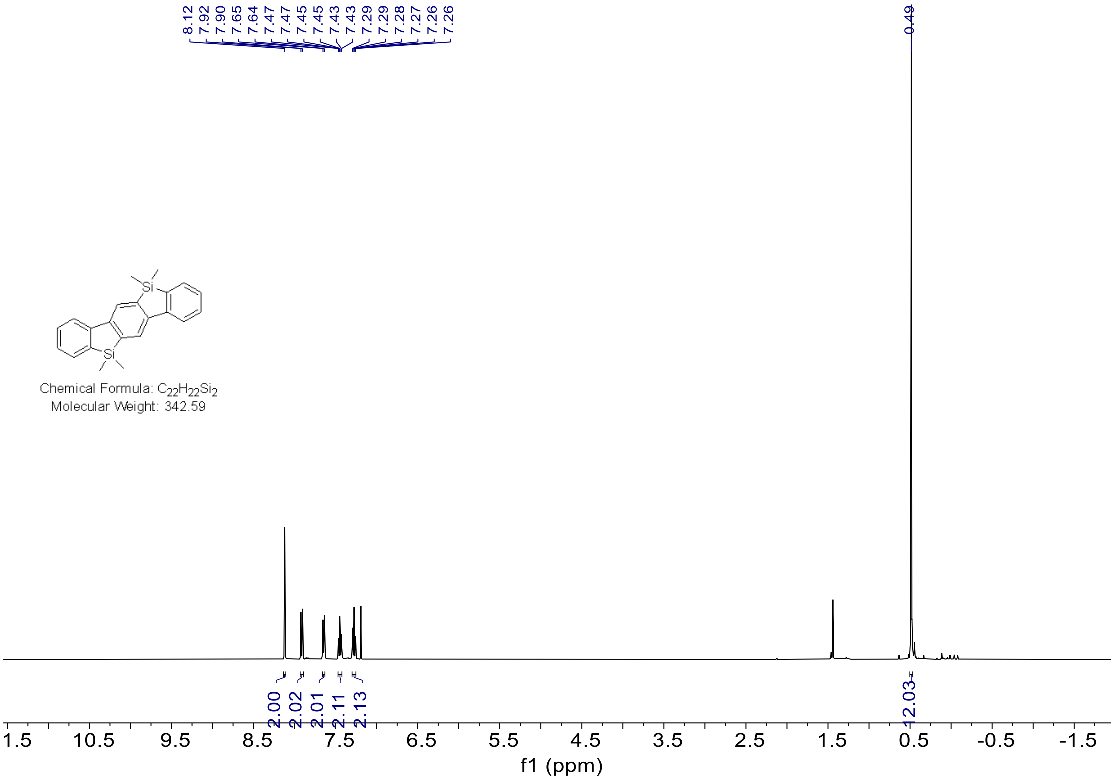

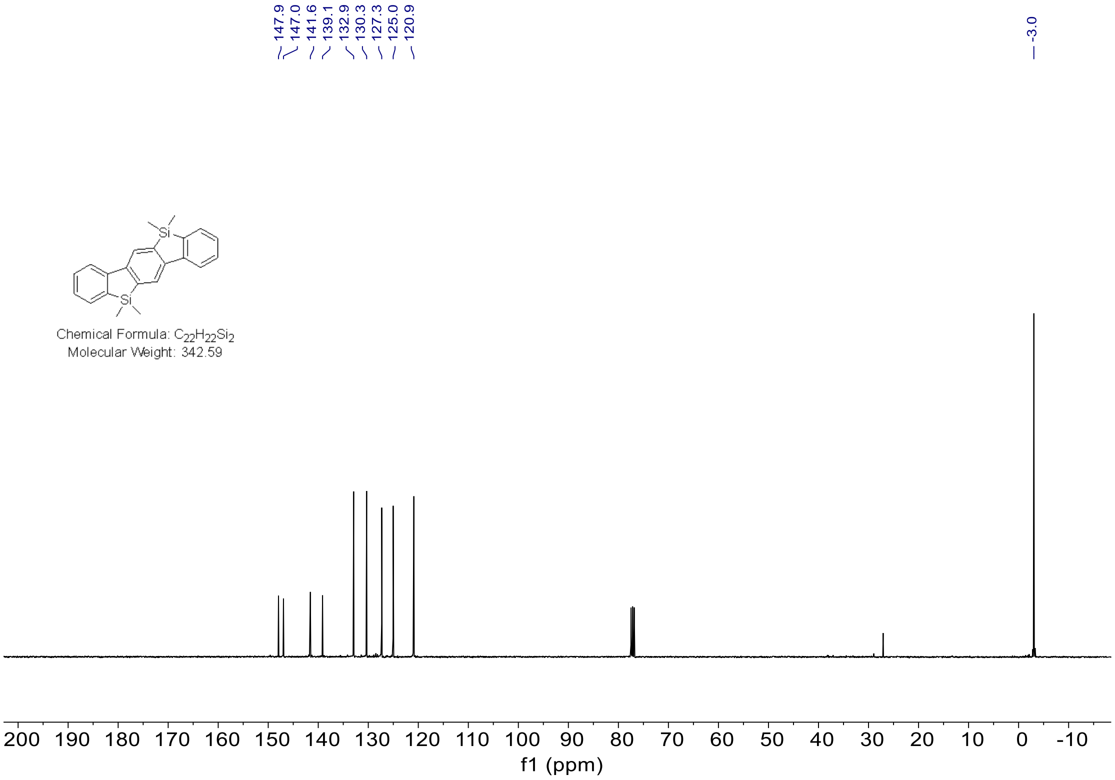

9,9′-Spiro-9-silabifluorene (6t).

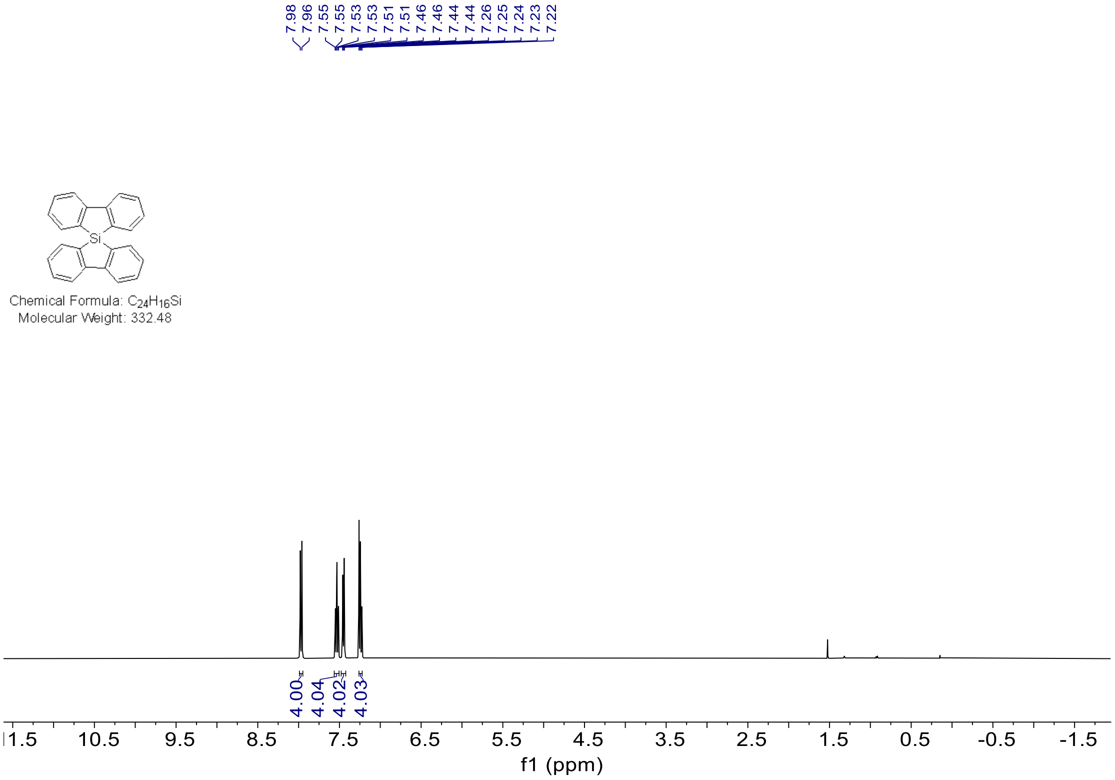

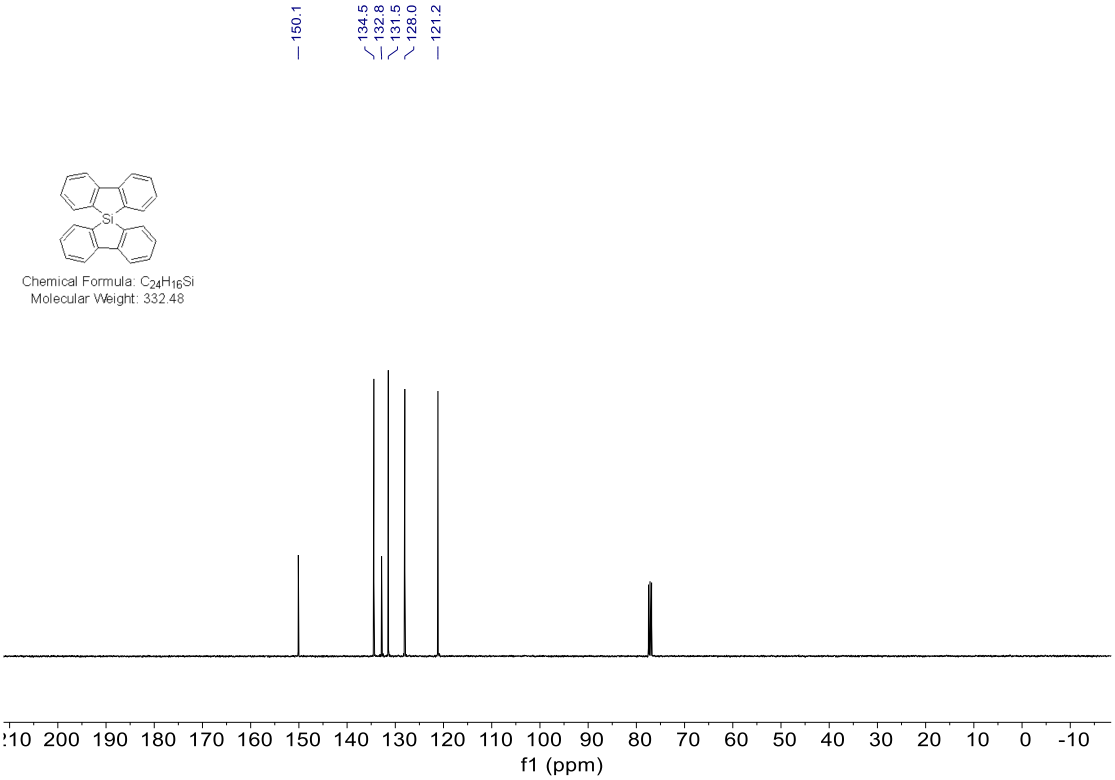

5,5-Diphenyl-*5H*-dibenzo[*b,d*]silole (6u).

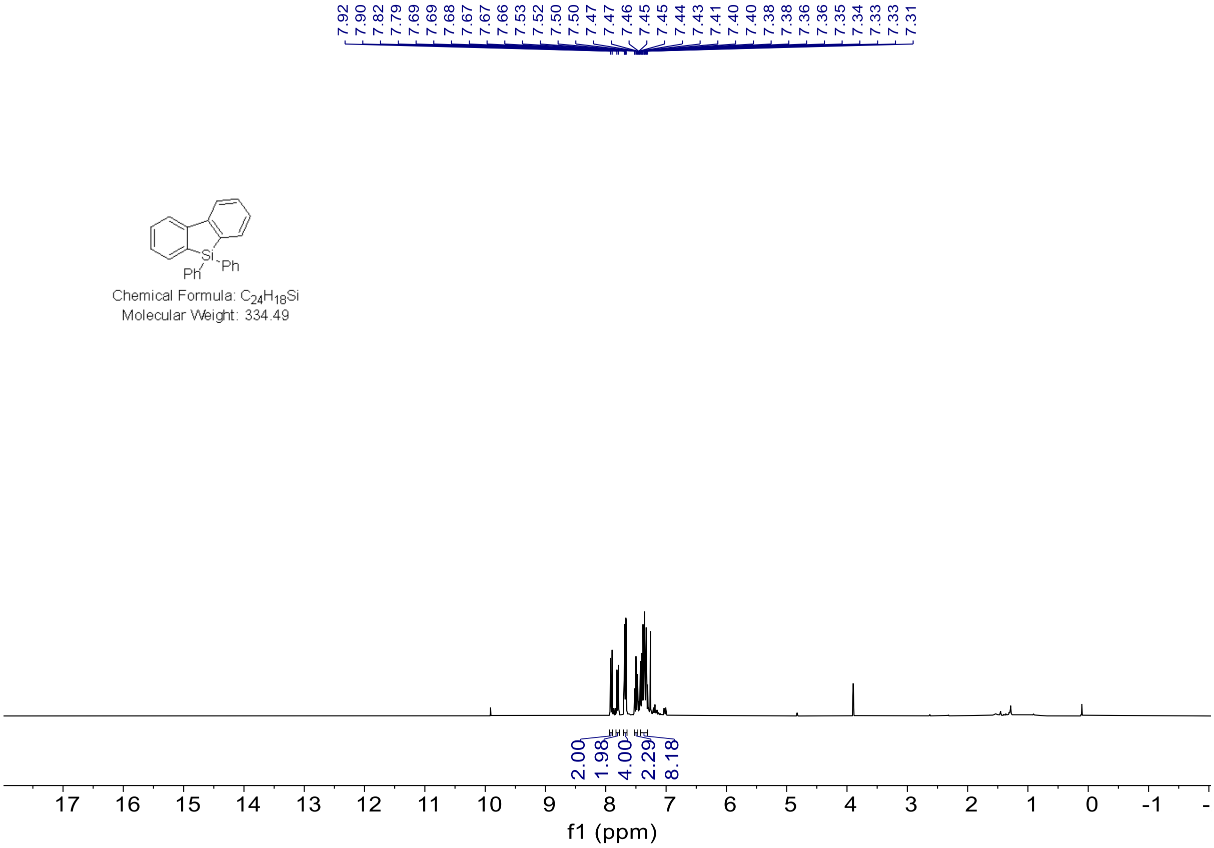

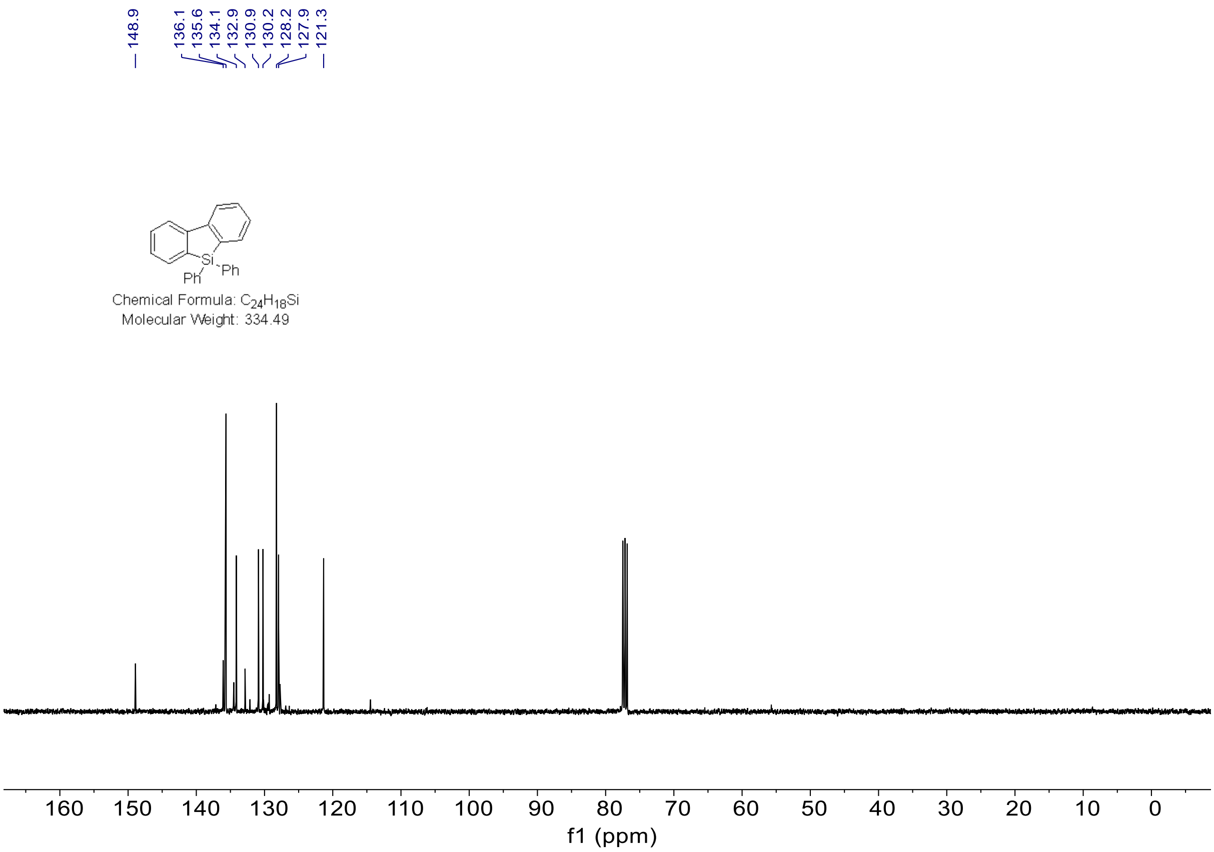

9-Methyl-9-phenyl-9-silafluorene (6v).

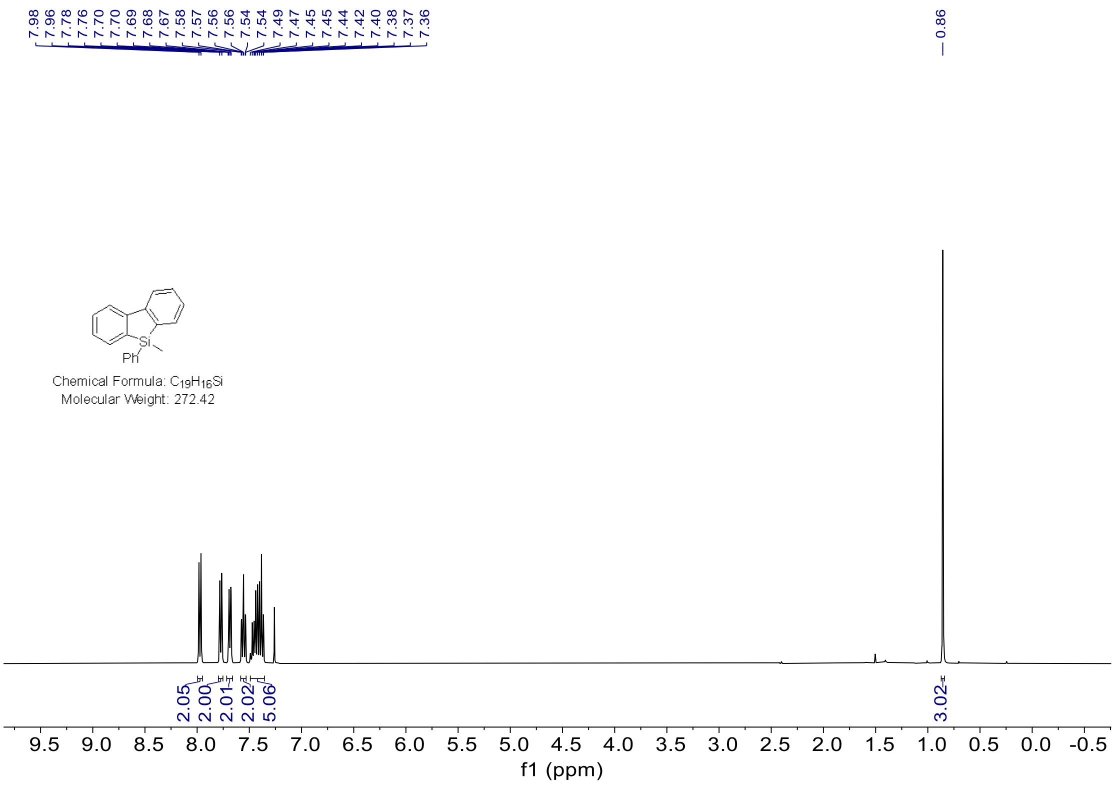

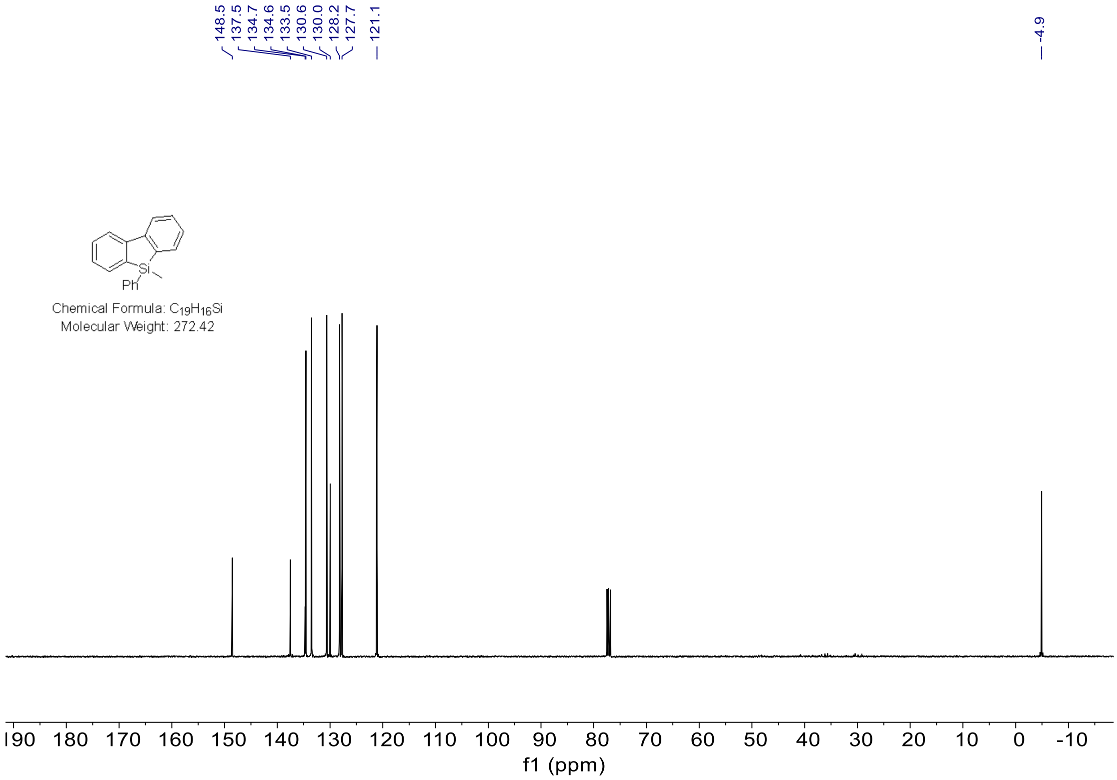

9,9-Diethyl-9-silafluorene (6w)

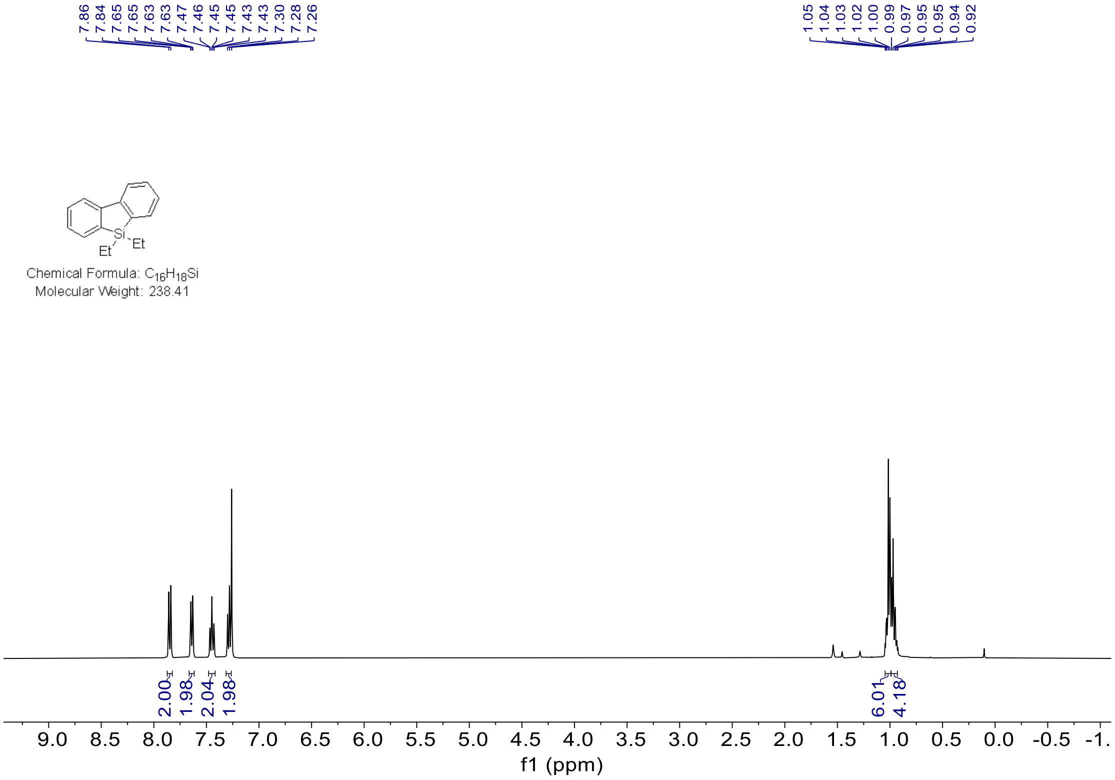

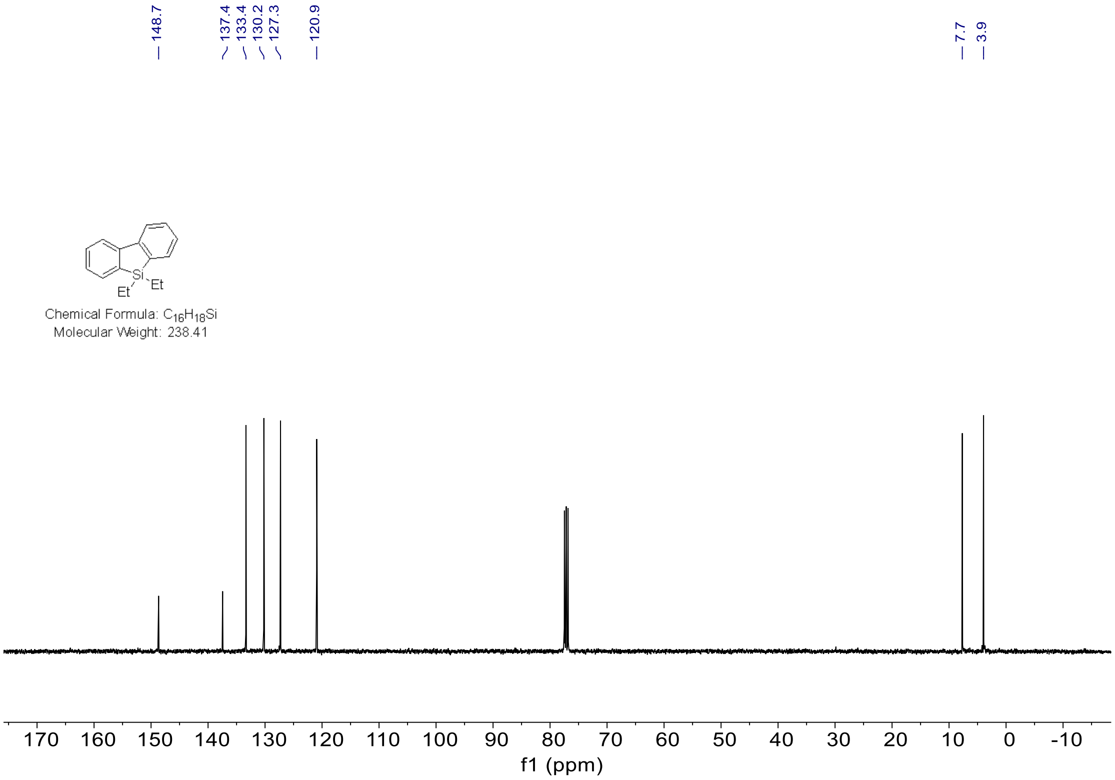

9,9-Dimethyl-9-germafluorene (6x).

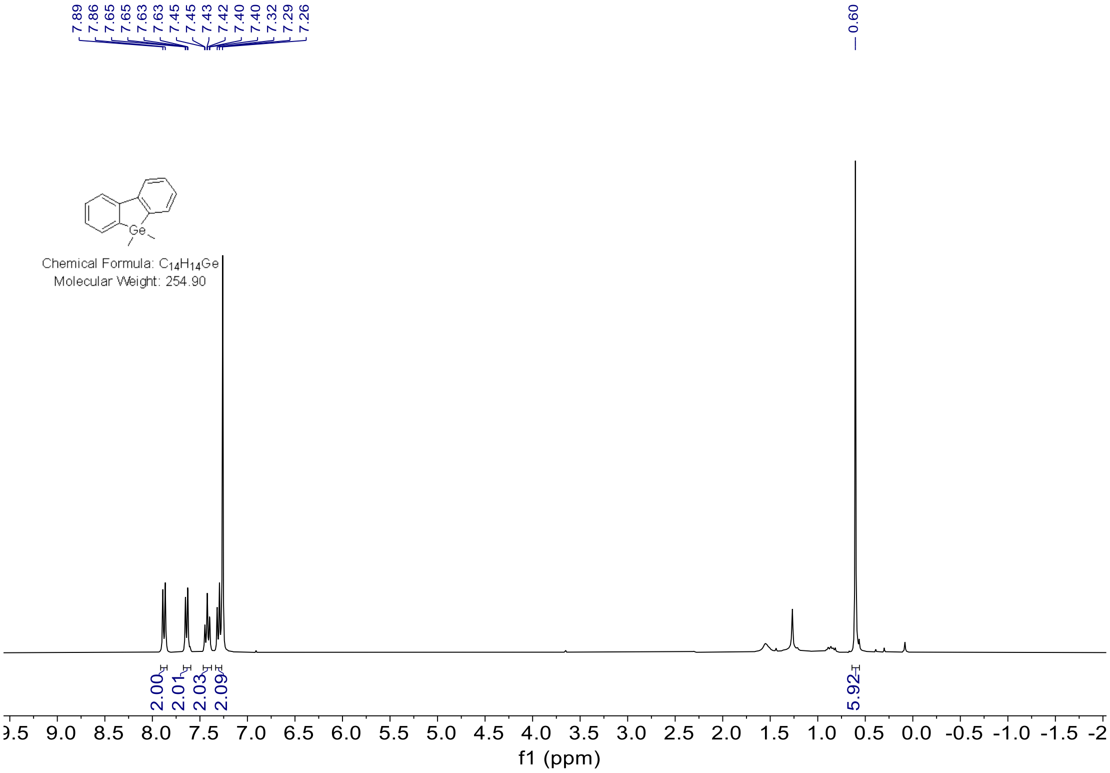

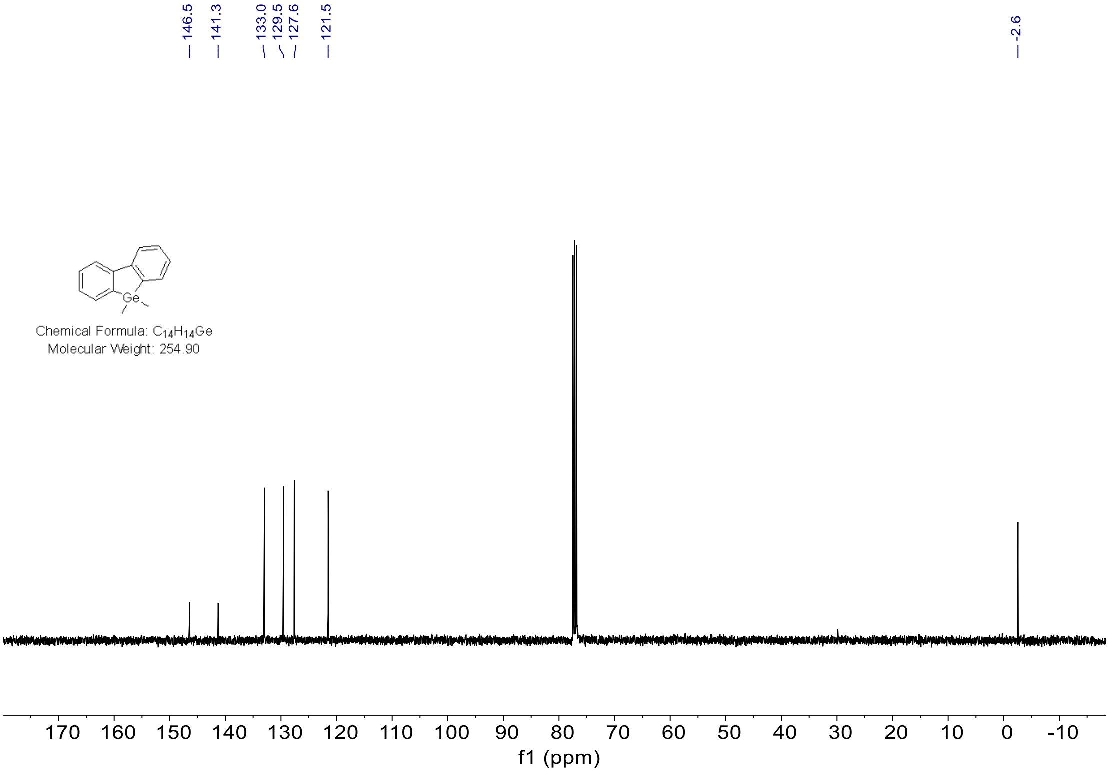

Supplement: Supplementary file 1 — The authors have cited additional references within the Supporting Information [43, 44, 45, 46, 47, 48, 49, 50, 51, 52, 53, 54, 55, 56, 57, 58]. Supporting File: anie72205‐sup‐0001‐SuppMat.pdf. [file ANIE-65-e5584122-s001.pdf]
